# A systematic review and meta-analysis on the association between CD36 rs1761667 polymorphism and cardiometabolic risk factors in adults

**DOI:** 10.1038/s41598-022-09908-0

**Published:** 2022-04-08

**Authors:** Zeinab Yazdanpanah, Hassan Mozaffari‐Khosravi, Masoud Mirzaei, Mohammad Hasan Sheikhha, Amin Salehi-Abargouei

**Affiliations:** 1 Department of Nutrition, School of Public Health, Shahid Sadoughi University of Medical Sciences, Yazd, PO Code 8915173160 Iran; 2 Nutrition and Food Security Research Center, Shahid Sadoughi University of Medical Sciences, Yazd, Iran; 3 Yazd Diabetic Research Center, Shahid Sadoughi University of Medical Sciences, Yazd, Iran; 4 Yazd Cardiovascular Research Centre, Non-Communicable Research Institute, Shahid Sadoughi University of Medical Sciences, Yazd, Iran; 5 Department of Medical Genetics, Shahid Sadoughi University of Medical Sciences, Yazd, Iran; 6 Abortion Research Center, Yazd Reproductive Sciences Institute, Shahid Sadoughi University of Medical Sciences, Yazd, Iran

**Keywords:** Genetics, Cardiology

## Abstract

The cluster of differentiation 36 (CD36) is one of the main receptors implicated in the pathogenesis of the cardiovascular disease. This study aimed to assess the association between CD36 rs1761667 polymorphism and cardiometabolic risk factors including body mass index (BMI), waist circumference (WC), total cholesterol (TC), triglyceride, HDL-C, LDL-C, blood pressure and fasting blood glucose (FBG). PubMed, EMBASE, Scopus, web of science, and Google Scholar were searched up to December 2021. Subgroup and meta-regression analyses were conducted to explore sources of heterogeneity. Eighteen eligible studies (6317 participants) were included in the study. In the overall analysis, a significant association was found between rs1761667 polymorphism of CD36 and TG in allelic (*p* < 0.001), recessive (*p* = 0.001) and homozygous (*p* = 0.006) models. A relationship between this polymorphism and HDL-C and FBG level was observed in the recessive genetic model. In the subgroup analysis, the A allele was associated with impaired lipid profiles (TC, LDL-C and HDL-C) in the Asian population. The influences of health status, design of the study, confounders, and other sources of heterogeneity should be considered when interpreting present findings. Cohort studies with large sample size and in different ethnicities are needed to confirm the relationship between rs1761667 SNP and cardiometabolic risk factors.

## Introduction

Cardiovascular diseases (CVD) are the most life-threatening conditions which have negative impacts on development and economic growth. Cardiometabolic risk factors including obesity, dyslipidemia, dysglycemia, and elevated blood pressure, increase the risk of cardiometabolic diseases. Impaired cardiometabolic risk factors can be present for years before the clinical symptoms become apparent; therefore, their management is difficult for physicians^1^. It has been demonstrated that genetic factors play a role in the development of cardiometabolic risk independent of environmental factors^2^. The cluster of differentiation 36 (CD36), also known as platelet glycoprotein IV or IIIb, is one of the most important membrane proteins presents on the surface of a wide variety of cell types including adipocytes, macrophages, skeletal and cardiac myocytes, hepatocytes, microvascular endothelial cells, breast, kidney, platelets, microvascular endothelial cells, and epithelial cells in the gut^3^. It has been shown that CD36 plays a role in inflammatory reactions, angiogenesis, orosensory perception of dietary lipid and fat preference, regulating the metabolic pathways of insulin-resistance, transporting long-chain fatty acids (LCFA) into adipose and muscle tissues, chylomicron synthesis, and energy metabolism^4^. As a consequence of its functions, CD36 might be associated with a wide range of disorders such as CVD, dyslipidemia, hypertension, diabetes, metabolic syndrome, and cancer^5,6^. A genome-wide association study (GWAS) of the four large cohorts (19,602 white people in whom 1544 cases of stroke) showed that CD36 rs3211928 was significantly associated with stroke. Several studies have been conducted on the association between single-nucleotide polymorphisms (SNPs) in the CD36 gene including rs1761667 (A/G substitution) with CVD^7,8^, type 2 diabetes mellitus (T2DM)^9^, consumption of total fat and fat taste perception, obesity^10^, and metabolic syndrome^11^. These studies have been performed in various ethnic populations around the world. However, ambivalent results were obtained regarding the association between genotype distribution of rs1761667 and cardiometabolic risk factors. For instance, Bayoumy et al.^11^ demonstrated that individuals with AA genotype of the CD36 rs1761667 had a significantly lower degree of dyslipidemia, systolic blood pressure (SBP), and waist circumstance (WC) compared to individuals with AG and GG genotype. Boghdady et al.^8^ also showed that the AG genotype may be involved in the pathogenesis of coronary artery disease, raised body mass index (BMI), metabolic syndrome, and T2DM. On the other hand, Pioltine et al.^12^ reported that the SNP rs1761667 in the CD36 gene was not associated with obesity risk.

To the best of our knowledge, there is no systematic review and meta-analysis trying to examine the possible relationship between CD36 rs1761667 polymorphism and cardiometabolic risk factors. Thus, the current study aimed to examine the association between this polymorphism and cardiometabolic risk factors including BMI, WC, total cholesterol (TC), triglyceride (TG), high-density lipoprotein cholesterol (HDL-C), low-density lipoprotein cholesterol (LDL-C), systolic and diastolic blood pressure, and fasting blood glucose (FBG).

## Results

### Literature search and study characteristics

**Figure 1:**
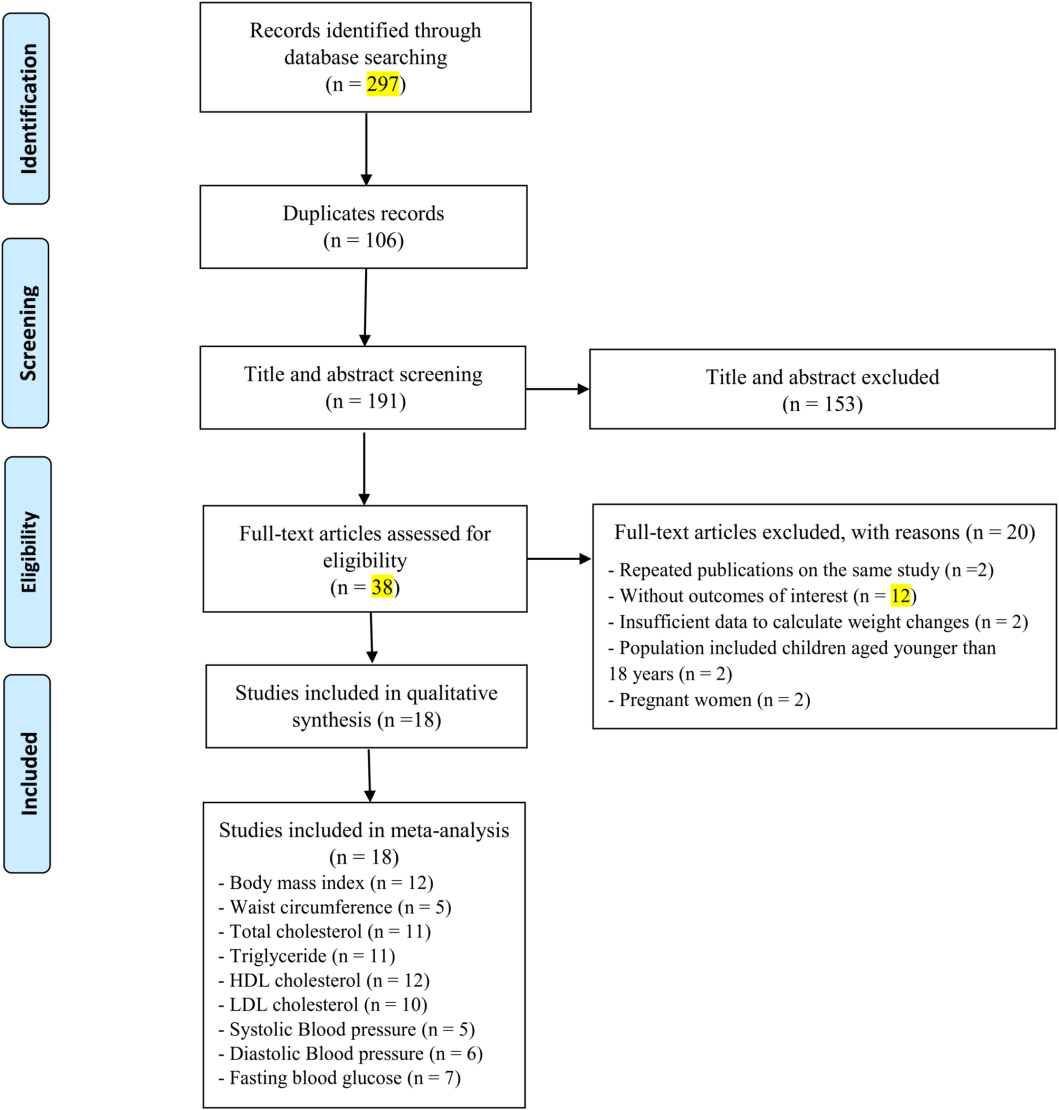
Flow diagram for the study selection process.

In total, 297 publications were identified in the initial search; from which 106 studies were duplicates, and 153 articles did not meet the eligibility criteria after screening titles/abstracts. The full texts of thirty-eight articles were assessed for further consideration and twenty articles were excluded for the following reasons: reported duplicate data (n = 2), had no data on the outcome variables (n = 12), conducted on pregnant women (n = 2), children and adolescents aged below 18 years (n = 2) and did not provide the sufficient data (n = 2) (Supplementary Table S1). The article selection procedure is illustrated in Fig. 1. Eventually, 18 studies with 6317 participants were included in the systematic review^7,8,11,13–26^. One study reported BMI before pregnancy; thus, it was included for this variable and this article was not used for other risk factors^27^.

General characteristics of the 18 eligible studies are provided in Table 1. Six studies were from Asia^7,15,19,25–27^, five from African^8,11,20–22^, and six from Europe^13,14,17,18,23,24^,and one study was from the USA/Italy^16^. The majority of eligible studies included both sexes and only two^20,27^ and one^17^ studies were performed on women and men, respectively. The participants were aged 18–75.73 years and the mean body mass index was ranged from 21.66 to 34.60 kg/m^2^. Seven studies were conducted on healthy individuals^16–18,20,21,23,27^, three studies targeted patients with heart diseases^7,8,19^, one study included individuals with T2DM^25^, metabolic syndrome^11^, and the six remaining studies included adults with other health status^13–15,22,24,26^. In the majority of studies, genotype distributions were in HWE^7,11,13,15,16,21,22,24–27^. HWE was not reported in six studies^8,14,17,18,20,23^ and in one publication which reported the results in four different groups, HWE were not in equilibrium in three groups^19^. Of the total 18 studies that assessed the relationship between CD36 rs1761667 genotypes and cardiovascular risk factors, nine studies were case–control (five studies reported only the data of the case group^7,8,11,25,27^, two studies combined the data of two groups^24,26^, the rest of the studies revealed the result separately in the case and control groups^18,19^), nine studies were cross-sectional, ^13–17,20–23^ of which 2 were baseline assessment of clinical trials^14,17^ (Table 1).

**Table 1:** Characteristics of studies that were included in this systematic review and meta-analysis.

Author (year)	Country	Study design	Number and sex (F/M)	BMI (Kg/cm^2^)	Genotypes frequencies	Mean age (years)	Notes about participants	Ethnicity	Method of genotype measured	Reported data	Hardy–Weinberg
Bayoumy et al.^11^	Egypt	Case–control, case assessment included	33 F/67 M	33.40 ± 3.20	GG: 5 AG: 70 AA: 25	GG: 45.00 AG: 44.00 AA: 47.00	Metabolic syndrome	Arabs	Real-time PCR	WC, TC, TG, HDL-C, LDL-C, SBP, DBP	Equilibrium
Boghdady et al.^8^	Egypt	Case–control, case assessment included	16 F/31 M	29.40 ± 3.40	GG: 9 AG: 30 AA: 8	GG: 54.80 AG: 53.80 AA: 54.60	Coronary artery disease (CAD)	African Americans	Real-time PCR	BMI, WC, TC, TG, HDL-C, LDL-C	NM
Dalton et al.^13^	UK	Cross-sectional	85 F&M	GG: 23.51 ± 4.18 AG: 25.02 ± 4.30 AA: 24.31 ± 3.69	GG: 28 AG: 39 AA: 18	26.10	Individuals susceptible to overeating	Caucasian	Sequenom Mass Array system	BMI	Equilibrium
Dawczynski et al.^14^	Germany	Clinical trial, baseline assessment included	45 F&M	26.31 ± 3.77	GG: 8 AG: 24 AA: 13	60.00	Mildly hypertriacylglycerolemia	NM	Sequencing	TG, HDL-C	NM
Fujii et al.^15^	Japan	Cross-sectional	267 F/228 M	GG: 23.60 ± 3.40 AG: 23.70 ± 3.40 AA: 23.40 ± 2.80	GG: 268 AG: 190 AA: 37	GG: 62.90 AG: 64.00 AA: 64.90	Community-dwelling individuals (hypertension, dyslipidemia, diabetes, obesity)	Asians	PCR-CTPP	BMI, WC, TG, HDL-C, SBP, DBP, FBG	Equilibrium
Ma et al.^16^	USA/ Italy	Cross-sectional	328 F/214 M	30.67 ± 5.54	GG: 115 AG: 273 AA: 154	GG: 35.00 AG: 36.00 AA: 38.00	Healthy individuals of Caucasian origin	Caucasian	AcycloPrime-FP SNP Detection System	TC, TG, HDL-C, SBP, DBP, FBG	Equilibrium
Madden et al.^17^	UK	Clinical trial, baseline assessment included	108 M	GG: 27.54 ± 4.20 AG: 27.75 ± 3.91 AA:26.90 ± 4.02	GG: 27 AG: 51 AA: 30	GG: 57.00 AG: 55.80 AA: 54.00	Healthy middle-aged men	Caucasian	Real-Time PCR	BMI, TG, HDL-C, LDL-C, FBG	NM
Melis et al.^18^	Italy	Case–control	37 F/25 M	28.56 ± 7.52	Case GG: 16/ AG: 31/ AA: 15	NM	Healthy obesity	Caucasian	PCR–RFLP	BMI	NM
			43 F/21 M		Control GG: 18/ AG: 39/ AA:7		Healthy normal weight				
Momeni-Moghaddam et al.^19^	Iran	Case–control	25 F&27 M	GG: 25.54 ± 5.26 AG: 25.35 ± 4.88 AA: 24.10 ± 4.35	Case GG: 6/ AG: 40/ AA: 6	GG: 55.50 AG: 54.90 AA: 52.50	HTN without CAD	Asians	PCR–RFLP	BMI, TC, TG, HDL-C, LDL-C, SBP, DBP, FBG	Disequilibrium
			13 F&44 M		Case GG: 17/ AG: 38/ AA: 2	GG: 57.47 AG: 56.43 AA: 50.00	CAD&HTN				Disequilibrium
			11 F&54 M		Case GG: 12/ AG: 45/ AA: 8	GG: 56.18 AG: 56.69 AA: 54.13	CAD				Disequilibrium
			26 F&39 M		Control GG: 26/ AG: 30/ AA: 9	GG: 52.65 AG: 55.93 AA: 50.71	Coronary angiography (without HTN and CAD)				Equilibrium
Mrizak et al.^20^	Tunisia	Cross-sectional	203 F	34.60 ± 4.20	GG: 42/ AG: 102/ AA: 59	38–43	Healthy obesity women	African	PCR–RFLP	TC, LDL-C	NM
Ramos-Lopez et al.^21^	Mexico	Cross-sectional	157 F/75	21.66 ± 2.26	GG: 46 AG: 104 AA: 82	18–25	Healthy normal-weight	Admixed population	PCR–RFLP	TC, TG, HDL-C, LDL-C	Equilibrium
Ramos-Lopez et al.^22^	Mexico	Cross-sectional	40 F/33 M	GG: 24.40 ± 3.10 AG: 24.90 ± 4.20 AA: 26.60 ± 4.10	GG: 11 AG: 40 AA: 22	GG: 53.70 AG: 51.40 AA: 48.10	Chronic hepatitis C virus infection	Amerindian, Caucasian and African	Real-Time PCR	BMI, TC, TG, HDL-C, LDL-C, FBG	Equilibrium
Shen et al.^23^	UK	Cross-sectional	95 F/41 M	22.9 ± 3.96	GG: 37 AG: 66 AA: 33	18–55	Healthy adults	Caucasian, African, West Asian and East Asian	Fluorogenic 5' nuclease assay (Taq-Man)	BMI	NM
Solakivi et al.^24^	Finland	Case–control	736 F&M (Case: 314 + Control: 422)	Case: 28.80 ± 5.20 Control: 25.50 ± 3.60	GG: 158 AG: 376 AA: 202	50.00	Hypertension & non-hypertensive healthy subjects	Caucasian	Competitive Allele Specific PCR (KASP) technique	BMI, TC, SBP, DBP, FBG	Equilibrium
Yang et al.^27^	China	Case–control, case assessment included	209 F	21.95 ± 2.76	GG: 88 AG: 99 AA: 22	32.99	Healthy women with GDM^a^	Asians	Taq-man allelic discrimination assay	BMI	Equilibrium
Yuan et al.**^25^	China	Case–control, case assessment included	42 F/70 M	GG: 23.80 ± 2.70 AG: 24.40 ± 3.20 AA: 23.50 ± 2.60	GG: 41 AG: 49 AA: 22	57.10	T2DM	Asians	PCR–RFLP	BMI, TC, TG, HDL-C, LDL-C, DBP, FBG	Equilibrium
Zhang et al.^7^	China	Case–control, case assessment included	43 F/69 M	23.78 ± 2.85	GG: 43 AG: 60 AA: 9	64.04	Coronary artery heart disease	Asians	PCR–RFLP	BMI, TC, TG, HDL-C, LDL-C	Equilibrium
Zhang et al.^26^	China	Case–control	1359 F&M (Case: 367 + Control: 992)	Case: 25.52 ± 3.42 Control: 25.29 ± 3.48	GG: 543 AG: 631 AA: 185	75.73	Atherothrombotic stroke & without stroke	Asians	ligase detection reaction (LDR) probe sequences	TC, HDL-C, LDL-C	Equilibrium

*F* female, *M* male, *NM* HNW, Healthy normal weight, *HO* Healthy obesity, not mention, *HTN* hypertension, *T2DM* type 2 diabetes mellitus, *GDM* gestational diabetes mellitus, *PCR* polymerase chain reaction, *PCR-CTPP* Polymerase chain reaction with confronting two-pair primers, *PCR–RFLP* polymerase chain reaction-restriction fragment length polymorphism, *BMI* body mass index, *WC* waist circumference, *TC* total cholesterol, *TG* triglyceride, *HDL-C* high-density lipoproteins cholesterol, *LDL-C* low-density lipoprotein cholesterol, *SBP* systolic blood pressure, *DBP* diastolic blood pressure, *FBG* fasting blood glucose.

^a^BMI was calculated before gestation.

^b^Insufficient data to calculate changes in SBP.

### Study quality assessment

Based on the Newcastle–Ottawa Scale, four^8,18,24,26^, and five^7,11,19,25,27^ case–control studies had a high and moderate methodological quality, respectively. All cross-sectional studies were categorized to be very good or good regarding their quality^13–16,20,22,23^, except for two studies^17,21^ (Supplementary Table S2).

### The results of quantitative analysis

In this meta-analysis, five common genotype models of CD36 (rs1761667) were considered: allelic model (A vs. G), dominant model (AA + GA vs. GG), recessive model (AA vs. GA + GG), homozygous model (AA vs. GG) and heterozygous model (GA vs. GG).

### Anthropometric measures

Twelve articles^7,8,13,15,17–19,22–25,27^ (n = 2478) reported the data on BMI and five articles^8,11,15,16,24^ with 1859 subjects were included in quantitative analysis for WC. As shown in Table 2, there was no significant association between CD36 rs1761667 genotypes and BMI in allelic (WMD = 0.029 kg/m^2^, 95%, CI: − 0.25, 0.31, *p* = 0.84, I^2^ = 31.20%), dominant (WMD = 0.23 kg/m^2^, 95%, CI: − 0.14, 0.61, *p* = 0.23, I^2^ = 17.90%), recessive (WMD = − 0.29 kg/m^2^, 95%, CI: − 0.84, 0.25, *p* = 0.29, I^2^ = 41.20%), homozygous (WMD = − 0.10 kg/m^2^, 95%, CI: − 0.72, 0.51, *p* = 0.73, I^2^ = 35.00%), and heterozygous (WMD = 0.35 kg/m^2^, 95%, CI: − 0.04, 0.75, *p* = 0.07, I^2^ = 15.70%) models (Supplementary Figure S5). According to the subgroup analysis, the association was significant for BMI in patients with heart disease (WMD = − 1.36 kg/m^2^, 95% CI: − 2.53, − 0.20, *p* = 0.02, I^2^ = 0.00%) and in the heterozygous model, in studies with medium quality (WMD = 0.58 kg/m^2^, 95% CI: 0.07, 1.09, *p* = 0.02, I^2^ = 0.00%) (Supplementary Table S3).

**Table 2:** The association between *CD36* rs1761667 polymorphism and anthropometric indices. All analyses were conducted using a random-effects model^a^.

	No. of data-sets	No. of subjects	Meta-analysis		Heterogeneity			Publication bias	
			WMD^2^ (95%CI)	P _effect_	*Q* statistic	P _within_	I^2^ (%)	Begg's tests	Egger's tests
**Body mass index (kg/m**^**2**^**)**									
Allelic model (A vs. G)	12	4956	0.029 (− 0.25, 0.31)	0.84	15.99	0.14	31.20	0.11	0.07
Dominant model (AA + GA vs. GG)		2478	0.23 (− 0.14, 0.61)	0.23	13.40	0.26	17.90	0.15	0.18
Recessive model (AA vs. GA + GG)		2478	− 0.29 (− 0.84, 0.25)	0.29	18.71	0.06	41.20	0.53	0.26
Homozygous model (AA vs. GG)		1255	− 0.10 (− 0.72, 0.51)	0.73	16.93	0.11	35.00	0.15	0.09
Heterozygous model (GA vs. GG)		2028	0.35 (− 0.04, 0.75)	0.07	13.04	0.29	15.70	0.45	0.30
**Waist circumference (cm) **^**b**^									
Allelic model (A vs. G)	5	3718	− 0.92 (− 2.20, 0.35)	0.15	9.45	0.05	57.70	–	–
Dominant model (AA + GA vs. GG)		1859	− 0.70 (− 2.76,1.36)	0.50	8.38	0.07	52.30	–	–
Recessive model (AA vs. GA + GG)		1859	− 2.32 (− 4.71, 0.06)	0.056	11.87	0.01	66.30	–	–
Homozygous model (AA vs. GG)		949	− 2.25 (− 5.48, 0.98)	0.17	11.66	0.02	65.70	–	–
Heterozygous model (GA vs. GG)		1452	− 0.29 (− 2.04, 1.45)	0.74	5.92	0.20	32.40	–	–

^a^All analyses were done using the random-effects model.

^b^There was no evidence of publication bias by observing the funnel plots.

WMD, weighted mean difference; 95% CI, 95% confidence interval.

Also, no association was found for WC in allelic (WMD = − 0.92 cm, 95%, CI: − 2.20, 0.35, *p* = 0.15, I^2^ = 57.70%), dominant (WMD = -0.70 cm, 95%, CI: − 2.76,1.36, *p* = 0.50, I^2^ = 52.30%), recessive (WMD = − 2.32 cm, 95%, CI: − 4.71, 0.06, p = 0.056, I^2^ = 66.30%), homozygous (WMD = − 2.25 cm, 95%, CI: − 5.48, 0.98, *p* = 0.17, I^2^ = 65.70%), heterozygous (WMD = − 0.29 cm, 95%, CI: − 2.04, 1.45, *p* = 0.74, I^2^ = 32.40%) models (Table 2 and Supplementary Figure S6).

### Lipid profile

#### Total cholesterol (TC)

The overall meta-analysis of eleven studies with 3755 participants^7,8,11,16,19–22,24–26^ showed no significant association between CD36 rs1761667 polymorphism and serum TC levels (allelic: WMD = 0.41 mg/dl, 95% CI: − 1.60, 2.44, *p* = 0.68; dominant: WMD = 0.66 mg/dl, 95% CI: − 2.96, 4.30, *p* = 0.71; recessive: WMD = − 0.83 mg/dl, 95% CI: − 5.61, 3.95, *p* = 0.73; homozygous: WMD = 2.18 mg/dl, 95% CI: − 1.24, 5.61, *p* = 0.21; heterozygous: WMD = − 1.37 mg/dl, 95% CI: − 6.53, 3.79, *p* = 0.60) models and heterogeneity between included studies was moderate to high (Table 3 and Supplementary Figure S7). In the subgroup analysis, the association was significant in studies conducted in the Asian population in allelic (WMD = 1.04 mg/dl, 95% CI: 0.88, 1.20, *p* < 0.001, I^2^ = 0.00%) and homozygous (WMD = 1.17 mg/dl, 95% CI: 0.76, 1.58, *p* < 0.001, I^2^ = 0.00%) models and healthy individuals (AA vs. GG: WMD = 5.52 mg/dl, 95% CI: 1.60, 9.43, *p* = 0.006, I^2^ = 18.50%). Furthermore, serum TC level was significantly higher in adjusted studies under allelic, dominant and heterozygous models (Supplementary Table S4).

**Table 3:** The association between *CD36* rs1761667 polymorphism and lipid profile. All analyses were conducted using a random-effects model^a^.

	No. of data-sets	No. of subjects	Meta-analysis		Heterogeneity			Publication bias	
			WMD^2^ (95%CI)	P_effect_	*Q* statistic	P_within_	I^2^ (%)	Begg's tests	Egger's tests
**Total cholesterol (mg/dl)**									
Allelic model (A vs. G)	11	7510	0.41 (− 1.60, 2.44)	0.68	28.23	0.002	64.60	0.35	0.33
Dominant model (AA + GA vs. GG)		3755	0.66 (− 2.96, 4.30)	0.71	29.09	0.001	65.60	0.87	0.46
Recessive model (AA vs. GA + GG)		3755	− 0.83 (− 5.61, 3.95)	0.73	91.91	< 0.001	89.10	0.35	0.77
Homozygous model (AA vs. GG)		1867	2.18 (− 1.24, 5.61)	0.21	20.59	0.02	51.40	0.21	0.82
Heterozygous model (GA vs. GG)		2962	− 1.37 (− 6.53, 3.79)	0.60	59.76	< 0.001	83.30	0.53	0.57
**Triglyceride (mg/dl)**									
Allelic model (A vs. G)	11	4210	− 7.11 (− 11.06, − 3.16)	** < 0.001**	18.97	0.04	47.30	0.062	0.069
Dominant model (AA + GA vs. GG)		2105	− 7.25 (− 14.64, 0.12)	0.054	21.96	0.01	54.50	0.35	0.002 ^b^
Recessive model (AA vs. GA + GG)		2105	− 14.54 (− 22.74, − 6.35)	**0.001**	32.33	< 0.001	69.10	0.75	0.55
Homozygous model (AA vs. GG)		1061	− 13.94 (− 23.82, − 4.06)	**0.006**	24.12	0.007	58.50	0.08	0.051
Heterozygous model (GA vs. GG)		1678	− 6.22 (− 15.40, 2.96)	0.18	30.66	0.001	67.40	0.75	0.004 ^b^
**HDL-C(mg/dl)**									
Allelic model (A vs. G)	12	6928	0.58 (− 0.29, 1.45)	0.19	39.64	< 0.001	72.20	0.73	0.29
Dominant model (AA + GA vs. GG)		3464	0.21 (− 1.16, 1.58)	0.76	28.30	0.003	61.10	1.00	0.54
Recessive model (AA vs. GA + GG)		3464	1.36 (0.08, 2.64)	**0.03**	29.03	0.002	62.10	0.73	0.14
Homozygous model (AA vs. GG)		1789	1.64 (− 0.41, 3.70)	0.11	36.79	< 0.001	70.10	0.94	0.26
Heterozygous model (GA vs. GG)		2852	− 0.31 (− 1.62, 0.99)	0.64	24.45	0.01	55.00	0.73	0.87
**LDL-C (mg/dl)**									
Allelic model (A vs. G)	10	5170	− 0.62 (− 4.40, 3.14)	0.74	55.82	< 0.001	83.90	0.15	0.75
Dominant model (AA + GA vs. GG)		2585	− 0.36 (− 3.80, 3.07)	0.83	15.54	0.07	42.10	0.15	0.02 ^b^
Recessive model (AA vs. GA + GG)		2585	− 2.68 (− 11.53, 6.15)	0.55	138.25	< 0.001	93.50	0.72	0.90
Homozygous model (AA vs. GG)		1295	− 2.07 (− 9.79, 5.64)	0.59	51.61	< 0.001	82.60	0.10	0.64
Heterozygous model (GA vs. GG)		2118	− 3.98 (− 8.77, 0.80)	0.10	27.70	0.001	67.50	0.37	0.01 ^b^

Significant values are in bold.

^a^ All analyses were done using the random-effects model.

^b^ These values were unchanged using the trim and fill method.

WMD, weighted mean difference; 95% CI, 95% confidence interval.

#### Triglyceride (TG)

The analysis of eleven studies^7,8,11,14–17,19,21,22,25^ (n = 2105) revealed that in overall, there was a significant association between rs1761667 polymorphism and TG values in allelic (WMD = − 7.11 mg/dl, 95% CI: − 11.06, − 3.16, *p* < 0.001, I2 = 47.30%), recessive (WMD = − 14.54 mg/dl, 95% CI: − 22.74, − 6.35, *p* = 0.001, I^2^ = 69.10%) and homozygous (WMD = − 13.94 mg/dl, 95% CI: − 23.82, − 4.06, *p* = 0.006, I^2^ = 58.50%) models, the TG level in the AA genotype group was significantly lower than that in G allele carriers, and the same difference was also observed in the above-mentioned models (Table 3 and Supplementary Figure S8). Subgroup analysis revealed that rs1761667 polymorphism was associated with decreased TG in the healthy population and HWE studies with AA genotype and A allele group, compared to the GG genotype and G allele group in homozygous (healthy participants: WMD = − 5.46 mg/dl, 95% CI: − 8.92, − 2.00, *p* = 0.002, I^2^ = 0.00%; equilibrium subgroup: WMD = − 11.85 mg/dl, 95% CI: − 23.02, − 0.68, *p* = 0.03, I^2^ = 67.60%) and allelic models (healthy participants: WMD = − 4.26 mg/dl, 95% CI: − 6.12, − 2.40, *p* < 0.001, I^2^ = 0.00%; equilibrium subgroup: WMD = − 6.65 mg/dl, 95% CI: − 11.20, − 2.11, *p* = 0.004, I^2^ = 58.80%), respectively. Further details about the subgroup analysis are provided in Supplementary Table S5.

### High-density lipoprotein cholesterol (HDL-C)

Based on the results of twelve datasets with 3464 individuals^7,8,11,14–17,19,21,22,25,26^, AA genotype population had a significantly higher HDL-C level (WMD = 1.36 mg/dl, 95% CI: 0.08, 2.64, *p* = 0.03, I^2^ = 62.10%) than G allele carriers in the recessive model (Table 3 and Supplementary Figure S9), with regard to subgroup analysis, this relationship was seen in the studies with HWE (WMD = 1.62 mg/dl, 95% CI: 0.23, 3.01, *p* = 0.02, I^2^ = 68.60%). Lower serum HDL-C levels were observed in the studies with the Asian population in allelic (WMD = − 0.09 mg/dl, 95% CI: − 0.18, − 0.01, *p* = 0.02, I^2^ = 0.00%), dominant (WMD = -0.29 mg/dl, 95% CI: − 0.43, − 0.14, *p* < 0.001, I^2^ = 0.00%) and heterozygous (WMD = − 0.38 mg/dl, 95% CI: − 0.52, − 0.23, *p* < 0.001, I^2^ = 0.00%) models (Supplementary Table S6).

### Low-density lipoprotein cholesterol (LDL-C)

Meta-analysis of ten studies^7,8,11,17,19–22,25,26^ (n = 2585) revealed, no significant association between CD36 rs1761667 polymorphism and LDL-C levels in different genetic models (allelic model: WMD = − 0.62 mg/dl, 95% CI: − 4.40, 3.14, *p* = 0.74; dominant model: WMD = -0.36 mg/dl, 95% CI: − 3.80, 3.07, *p* = 0.83; recessive model: WMD = − 2.68 mg/dl, 95% CI: − 11.53, 6.15, *p* = 0.55; homozygous model: WMD = − 2.07 mg/dl, 95% CI: − 9.79, 5.64, *p* = 0.59; heterozygous model: WMD = − 3.98 mg/dl, 95% CI: − 8.77, 0.80, *p* = 0.10) (Table 3 and Supplementary Figure S10). Allelic (WMD = 5.43 mg/dl, 95% CI: 0.16, 10.70, *p* = 0.04, I^2^ = 69.90%), recessive (WMD = 12.77 mg/dl, 95% CI: 1.99, 23.55, *p* = 0.02, I^2^ = 84.30%) and homozygous (WMD = 9.10 mg/dl, 95% CI: 0.29, 17.91, *p* = 0.04, I^2^ = 54.60%) models were associated with an increase in LDL-C levels in healthy participants and there was moderate to high heterogeneity between the included studies. Under the allelic (WMD = 1.32 mg/dl, 95% CI: 1.18, 1.45, *p* < 0.001, I^2^ = 0.00%) and homozygous (WMD = 1.92 mg/dl, 95% CI: 1.57, 2.28, *p* < 0.001, I^2^ = 0.00%) genetic models, rs1761667 had a significant association with the LDL-C levels in Asian population and there was no heterogeneity between the included studies. The results of subgroup analysis based on the study design and quality indicated that, the concentration of LDL in moderate quality and cross-sectional studies was lower in heterozygous model, whereas this variable was significantly higher in studies that adjusted the association for the potential confounders (WMD = 2.33 mg/dl, 95% CI: 2.17, 2.48, *p* < 0.001, I^2^ = 0.00%) than studies with no adjustment (WMD = − 5.14 mg/dl, 95% CI: − 8.43, − 1.84, *p* = 0.002, I^2^ = 1.70%) (Supplementary Table S7).

### Blood pressure

The analysis results for five studies (n = 2112)^11,15,16,19,24^ for systolic (SBP) and six studies with 2224 participants^11,15,16,19,24,25^ for diastolic blood pressure (DBP) are illustrated in Table 4. No significant association was detected between genetic models of CD36 rs1761667 polymorphism and blood pressure (Table 4 and Supplementary Figure S11–S12). In the recessive model, the average DBP in the AA genotype group was significantly lower than that of G allele carriers in studies which did not adjust the association for confounders (Supplementary Table S8). It should be noted that no relationship was observed regarding this factor in other subgroups.

**Table 4:** The association between *CD36* rs1761667 polymorphism and blood pressure and fasting blood glucose. All analyses were conducted using a random-effects model^a^.

	No. of data-sets	No. of subjects	Meta-analysis		Heterogeneity		
			WMD^2^ (95%CI)	P_effect_	*Q* statistic	P_within_	I^2^ (%)
**Systolic Blood pressure (mmHg) **^b^							
Allelic model (A vs. G)	5	4224	− 2.01 (− 4.92, 0.90)	0.17	36.92	< 0.001	89.20
Dominant model (AA + GA vs. GG)		2112	− 4.17 (− 9.22, 0.88)	0.10	38.98	< 0.001	89.70
Recessive model (AA vs. GA + GG)		2112	− 4.83 (− 12.95, 3.28)	0.24	126.85	< 0.001	96.80
Homozygous model (AA vs. GG)		1050	− 8.42 (− 18.09, 1.24)	0.08	85.03	< 0.001	95.30
Heterozygous model (GA vs. GG)		1669	− 3.30 (− 7.84, 1.23)	0.15	28.79	< 0.001	86.10
**Diastolic Blood pressure (mmHg) **^b^							
Allelic model (A vs. G)	6	4448	− 0.14 (− 0.75, 0.46)	0.63	4.89	0.43	0.00
Dominant model (AA + GA vs. GG)		2224	− 0.14 (− 1.20, 0.92)	0.79	0.87	0.97	0.00
Recessive model (AA vs. GA + GG)		2224	− 0.85 (− 2.56, 0.85)	0.32	9.50	0.08	47.70
Homozygous model (AA vs. GG)		1113	− 0.57 (− 2.14, 1.00)	0.47	5.87	0.31	14.80
Heterozygous model (GA vs. GG)		1759	0.001 (− 1.11, 1.11)	0.99	0.33	0.99	0.00
**Fasting blood glucose (mg/dl) **^b^							
Allelic model (A vs. G)	7	4610	2.07 (− 0.11, 4.25)	0.06	23.76	0.001	74.70
Dominant model (AA + GA vs. GG)		2305	3.64 (− 0.89, 8.17)	0.11	49.96	< 0.001	0.88
Recessive model (AA vs. GA + GG)		2305	2.33 (0.92, 3.74)	**0.001**	6.03	0.42	0.40
Homozygous model (AA vs. GG)		1173	4.28 (− 1.02, 9.60)	0.11	30.42	< 0.001	80.30
Heterozygous model (GA vs. GG)		1813	3.07 (− 1.17, 7.33)	0.15	39.95	< 0.001	85.00

Significant values are in bold.

^a^All analyses were done using the random-effects model.

^b^There was no evidence of publication bias by observing the funnel plots.

WMD, weighted mean difference; 95% CI, 95% confidence interval.

### Fasting blood glucose (FBG)

Seven studies^15–17,19,22,24,25^ which included a total of 2305 individuals assessed the serum FBG in different genotypes of CD36 rs1761667. According to the pooled analysis, under the recessive genetic model, the homozygous A-allele carriers had a significantly higher FBG concentration compared with G allele carriers (WMD = 2.33 mg/dl, 95% CI: 0.92, 3.74), *p* = 0.001, I^2^ = 6.03%) and no heterogeneity was observed between included studies (Table 4 and Supplementary Figure S13). No significant association was observed between the rest of the genotypes with this parameter. Consistent with the results of the overall analysis, a significant relationship of rs1761667 polymorphism with the FBG level was observed under the recessive model in the Caucasian population (WMD = 3.30 mg/dl, 95% CI: 0.66, 5.93, *p* = 0.01)and the between-study heterogeneity was low (I^2^ = 39.50% and *p* = 0.19). Under allelic, recessive and homozygous models, a significant correlation was observed between this SNP and circulating FBG levels in the healthy group and cross-sectional studies. The between-study heterogeneity was not observed in these subgroups (I^2^ = 0.00%). The findings of other subgroup analyses were reported in Supplementary Table S9.

### Meta-regression and cumulative meta-analysis

No significant association was observed between potential modulators (e.g. ethnicity, health status, Hardy–Weinberg equilibrium, quality score, design of the study, adjustment of confounders) and effect sizes for the association between CD36 rs1761667 polymorphism and BMI, HDL-C, LDL-C, SBP and DBP using meta-regression analyses. Ethnicity, quality score, design of the study, adjustment of confounders in the homozygote model and Hardy–Weinberg equilibrium were associated with between-study heterogeneity in the allelic model on the relation of CD36 SNP at rs1761667 on the levels of TC was determined using meta-regression (Supplementary Table S4). Meta-regression based on the mentioned modulators showed that all of them influence the association between rs1761667 polymorphism and the levels of TG in allelic and homozygote models; in addition, ethnicity, health status and Hardy–Weinberg equilibrium were associated with a difference in TG levels in the homozygote model; however, heterogeneity remained unchanged (Supplementary Table S5). Meta-regression according to ethnicity, health status and health status showed that these variables were significantly related to the difference in FBG values in the recessive and allelic models, respectively (Supplementary Table S9). These variables might be the cause of inconsistency in the relationship between SNP rs1761667 and cardiometabolic risk factors and for the sources of the heterogeneity observed in the current study.

The cumulative meta-analysis confirmed the overall estimate was not influenced by the year of study except for FBG in the allelic model (Supplementary Figures S14–S22).

### Sensitivity analysis and publication bias

The deletion of each study, individually, from the meta-analysis altered some overall association with BMI (dominant and heterozygous models), WC (dominant model), triglyceride (dominant and heterozygous models), HDL-C (recessive, homozygous, heterozygous models), LDL-C (recessive, heterozygous models), FBG (allelic, homozygous model) which are provided in Supplementary Table S10.

No evidence of publication bias was found for BMI, TC and HDL-C levels. Begg’s and Egger’s asymmetry tests suggested evidence of publication bias for the meta-analysis of serum TG and LDL-C levels in the dominant and heterozygous models. However, the results remained unchanged using the trim and fill analysis, which means it is unlikely that publication bias significantly affected imputes estimates (Tables 2, 3 and 4). There was no evidence of publication bias for studies evaluating the association between CD36 rs1761667 polymorphism and WC, SBP, DBP, and FBG concentrations by visually observing the Begg’s funnel plots (Supplementary Figure S1–S4).

## Discussion

The results of the present systematic review and meta-analysis unraveled that CD36 rs1761667 polymorphism had a significant association with cardiometabolic risk factors such as circulating TG, HDL-C, and FBG levels. No significant relationship was observed for other comparisons in the overall analyses. A previous cohort study demonstrated that high triglyceride levels were three times more common in CVD patients than elevated cholesterol levels alone^28^. The CD36 is a multifunctional receptor that both persistent up-regulation and deficiency of this protein can increase the CVD risk. Abnormally up-regulated CD36 can lead to foam cell formation, endothelial apoptosis, exacerbated inflammation, macrophage trapping, and thrombosis. On the other hand, CD36 deficiency promotes metabolic disorders, dyslipidemia (increasing triglyceride, fatty acid, apoB48, and chylomicron remnants in plasma levels) and sub-clinical inflammation, all of which are cardiometabolic risk factors^29^. Similarly, regarding higher TG levels in individuals with the G/G genotype, previous studies demonstrated this finding and also showed that improvement in plasma TG levels after a fish oil-rich dietary intervention was lower in these individuals compared with G/A and A/A individuals^12,16,17^. In addition, a study in North Indian population showed that the presence of the minor rs1761667-allele A was associated with elevating the risk of developing T2DM^9^. The results of the ethnicity subgroup showed that Caucasians with AA genotype had a higher FBG concentration than G carriers. This discrepancy in this finding might be owing to different ethnicity, sample sizes, gene-environment and gene–gene interactions, publication bias and clinical heterogeneity. Another possible reason for this contrary finding can be a varied selection of the health status in different populations. On the other hand, the majority of eligible studies had evaluated fasting glucose levels. It was better to apply other glucose indicators (glycated hemoglobin A1c) because fasting blood glucose is affected by many factors such as BMI, psychological stress, smoking habits, potassium intake, etc^30,31^. So, it seems that for a definitive interpretation of this result further studies are needed.

The subgroup analysis demonstrated that the A allele was associated with elevated TC and LDL-C levels and decreased HDL-C levels in Asians. In line with the mentioned conclusion in the present study, some previous studies reported these outcomes and suggested A allele of rs1761667 as a susceptibility factor for high serum cholesterol levels, low HDL-C and atherothrombotic stroke^11,26,32^. The deficiency of CD36 occurs rarely in Caucasians and is relatively common (3–10%) in the population of Asian and African descent^33^. As mentioned before, CD36 deficiency is closely related to the elevated prevalence of metabolic abnormalities, including hyperlipidemia, and increased fasting glucose levels. Moreover, clinical investigations have shown that the rs1761667-A allele decreases the CD36 expression and is associated with upper recognition taste thresholds for fat and decreased lipid taste perception^34,35^. The significant relationship between the CD36 SNP rs1761667 variant and lipid levels were shown in Asians also highlights the interaction between the CD36 SNP rs1761667 variant and ethnicity in modulating the plasma lipids. Bayoumy et al.^11^ noted in an Egyptian population that the minor allele frequency (MAF) of rs1761667 polymorphism was merely 0.25, Noel et al.^36^ showed that the MAF was 0.46 in a Hispanic population. Previous studies have been reported the influence of interethnic differences in allele frequencies. These variations can contribute to the differences in gene expression and eventually, disease susceptibility. Therefore, it is important to consider the population variations in the allele frequency when trying to identify an association between a polymorphism and the risk of diseases^37–39^. Further studies with different ethnicities should be performed to confirm these conclusions.

The analyses also showed a significant association between increased levels of LDL-C, FBG and A allele of CD36 rs1761667 in healthy participants. It is mentioned that the synthesis and translocation of CD36 are influenced by various stimuli. Modifications of CD36 affect cardiac function via altering the cellular uptake of fatty acids in the myocardium. The increased CD36-induced fatty acid uptake could be harmful or beneficial under various pathological conditions^40^. CD36 is decreased in pathological cardiac hypertrophy and increased in diabetic cardiomyopathy and atherosclerosis. In a healthy heart, insulin promotes CD36 transportation from the endosome to the cell membrane. Simultaneously, the forkhead box O1 (FOXO1) transcription factor promotes CD36 expression^41^. Long chain fatty acids (LCFAs) absorbed by CD36 are used for oxidation and storage as lipids in mitochondrial. Whereas in diabetes, the enhancement of insulin strongly activates the PI3K-Akt (phosphatidylinositol 3‑kinase-protein kinase B) pathway that leads to a robust CD36 expression. On the other hand, upregulated mir-320 and down-regulated mir-200b-3p accelerate the CD36 transcription and translation, uptake of LCFAs facilitates in cardiomyocytes eventually. Intracellular LCFAs either enter the mitochondria for producing energy and the by-products-reactive oxygen species (ROS) or forms triglycerides. As we know, triglycerides accumulation can result in insulin resistance. The ROS assembly and insulin resistance deteriorate cardiac function and would trigger diabetic cardiomyopathy^42^. Therefore, CD36 has different functions in diverse conditions and there is a need for more in-depth study with different health status^40^. Moreover, a significant association with BMI was detected among subjects with heart diseases in the recessive model. The mechanisms underlying how modifications of CD36 affect fat metabolism and cardiometabolic risk factors still remain to be elucidated; however, some evidence has shown that CD36 plays a role in the metabolism of LDL-C and HDL-C and contributes directly to their regulation^43,44^. A systematic review of studies that investigated the association between CD36 and the metabolic complications of obesity, reported that CD36 may be involved in obesity-related complications in humans. Moreover, CD36 deficiency might affect myocardial uptake of LCFAs, delay clearance of plasma fatty acid after an oral meal, and be associated with abnormalities in chylomicron formation^45^. The CD36 is a membrane transporter of long-chain polyunsaturated fatty acid in many tissues including skeletal muscles, adipocytes and the heart. Dysfunction of this protein might reduce the intramuscular fatty acid oxidation rate. Therefore the availability of fatty acid enhances their storage in adipocytes^46,47^. Peroxisome proliferator-activated receptor (PPAR-γ) is a nuclear receptor that regulates adipocyte differentiation and adipogenesis and CD36 is regarded as a key factor in the activation of PPAR-γ and its change might influence PPAR-γ mediated adipocyte differentiation^48^. It has been suggested that CD36 expression is reduced in circumvallate taste buds among high-fat diet-induced obese rats which leads to a decreased sensitivity to fat taste, as a result the intake of fatty foods increases as a compensatory response ^49^. Muthuswamy et al.^50^ reported that a lower CD36 expression (in AA and AG genotype at rs1761667) might be involved in reducing the release of PYY from taste bud cells. The presence of CD36 in gustatory papillae, the main LCFA receptor in taste bud cells^51^, contributes to dietary fat taste perception and fat preference^35^. More preference and increased eating of fatty foods have been expressed, which may reflect a decline of oral and gastrointestinal fatty acid sensitivity in obesity^52^. Based on the results obtained after subgroup analysis according to quality assessment, design of studies, adjustment for confounders, and Hardy–Weinberg equilibrium, high-quality cohort studies considering confounders such as age, sex, BMI, smoking, alcohol consumption and physical activity are needed to confirm the results.

The current evidence showed no significant association of this CD36 SNP with blood pressure; however, subgroup analysis based on adjustment of confounders demonstrated a significant association between decreased DBP and studies which did not consider confounding variables into account. Molecular studies suggested that CD36 contributes to the production of nitric oxide. Since reducing nitric oxide activity in the renal medulla is associated with hypertension, it is proposed that a decreased CD36 in renal cells may be related to hypertension ^53^. Furthermore, some studies in animals indicated that there is a relationship between CD36 genetic background and regulation of blood pressure^54,55^. There are some reasons which may explain these inconsistencies. The conflicting results might be due to variations in the health status and the genotyping methods, differences in ethnicity of the populations and their sex, gene–gene interactions in various populations and interactions of rs1761667 polymorphism with other variants in the CD36 gene. Moreover, some environmental factors may affect CD36 expression, as alcohol and fatty acids are recognized to modify the epigenome that includes acetylation of histones, DNA methylation, etc. ^56,57^. According to the factors mentioned, observations are still controversial and further studies are needed to arrive at a firm conclusion about the association with blood pressure. However, it is worth noting that studies on CD36 suggest that transcriptional activation, post-translational modification and localization alterations in this protein may create new approaches for the treatment of CVD^40^.

The present meta-analysis has some strengths. To the best of our knowledge, this is the first meta-analysis about the relationship between genetic locus 7q11 rs1761667 polymorphism and cardiometabolic risk factors which tried to include all related publications. Notably, several subgroup analyses and meta-regression were performed to assess the potentially different relation between rs1761667 polymorphism and cardiometabolic risk factors. There are a number of limitations that should be considered when interpreting the current study’s results. First, some information (including sex, lifestyle, other genetic variations, alcohol consumption or smoking, physical activity and so on) was unavailable in studies to perform more detailed subgroup analysis. Age is another risk factor that plays an important role in the enhancement of cardiovascular risk factors and the development of CVD. Unfortunately, conducting subgroup analysis based on age groups was not possible in the present study. In addition, further research considering other environmental and genetic factors is still needed to come up with a more apprehensive estimation of CD36 rs1761667 polymorphism with cardiometabolic risk factors. Second, the number of studies available for some outcome variables and a number of subgroups was fairly small. Furthermore, the total sample size of Asian and European studies are still limited in our research. Another problem is the fact that some of the obtained relations [BMI (dominant and heterozygous models), WC (dominant model), TG (dominant and heterozygous models), HDL-C (recessive, homozygous and heterozygous models), LDL-C (recessive and heterozygous models) and FBG (allelic and homozygous models)] were affected by the removal of one or several studies in the sensitivity analysis. In addition, we could not find any GWAS finding on rs1761667; therefore, it was not possible to compare the results obtained in this study with GWAS result. A high level of heterogeneity was also observed between the studies included in analyses for different genetic models. Thus, the findings should be interpreted with caution.

In summary, the results of the current systematic review and meta-analysis indicated that CD36 rs1761667 polymorphism was significantly associated with decreased TG and elevated HDL-C and FBG concentration in the overall analysis. Asian populations with different genotypes have different levels of lipids (TC, LDL-C and HDL-C), which may affect the susceptibility of the disease in these people. The influence of ethnicity, confounders, and health status on pooled effects should be considered when interpreting the association between rs1761667 polymorphism and elevated cardiometabolic risk factors. Furthermore, cohort studies with a large sample size which take confounders into account are needed to confirm the associations found between rs1761667 SNP and cardiovascular risk factors.

## Methods

This systematic review and meta-analysis is reported in accordance with the PRISMA (Preferred Reporting Items for Systematic Reviews and Meta-Analyses) statement^58^. The study protocol was also registered in the prospective register of systematic reviews (PROSPERO) [protocol code: CRD42021253789].

### Search strategy

Relevant articles were identified through online search of the literature in PubMed/MEDLINE, EMBASE, Scopus, ISI (Web of Science) and Google Scholar up to December 2021 without any publication date, language, and any other restrictions. The keywords used to search were: rs1761667 OR “− 31,118 G > A” OR “− 31,118 G > A” OR “− 31,118 G > A” OR “− 31,118 G > A”. The research was also updated by adding the following keywords in the all fields "GWAS", "Genome-wide association studies", "Genome wide association studies", "Genome wide association study", "Genome-wide association study", "GWA study", "whole genome association study", "WGA study", "WGAS", "Whole-genome association study", "whole genome association studies", "whole-genome association studies", "WGA", "Whole Genome Association Analysis", "Whole-Genome Association Analysis", "Genome Wide Association Analysis", "Genome-Wide Association Analysis" to did not lose any article (Supplementary Table S11). The references of the relevant study were also examined manually for any missing related literature.

### Eligibility criteria

All published studies (cross-sectional, cohort, case–control designs and baseline of controlled clinical trials) that focused on CD36 rs1761667 polymorphism and cardiometabolic biomarkers such as anthropometric indices (BMI, and WC), lipid profile markers (TC, TG, HDL-C and LDL-C), blood pressure and FBG were included in the present review. Furthermore, articles with or without deviation from the Hardy–Weinberg equilibrium (HWE) were included. If the studies were done in pregnant, lactating women, children, adolescents aged < 18 years, and also if they had no outcomes of interest, were excluded from the review. In the case of repeated publications on the same study, we selected the one which included a higher number of participants.

### Data extraction

For each eligible study, data were extracted on the author’s last name, publication year, country, participants’ characteristics (sample size, sex, age, health status and ethnicity), method of genotyping, HWE and mean ± standard deviation (SD) of desired outcomes. Two reviewers (ASA and ZY) independently screened the titles and abstracts, assessed the full text of the relevant articles, and extracted the data. Any possible disagreements or discrepancies were resolved by group discussion.

### Risk of bias assessment

Two authors independently evaluated the methodological quality of eligible studies. The quality of each study was assessed by using the Newcastle–Ottawa (NOS) Scale for case–control (eight items)^59^ and its modified version adapted for cross-sectional studies (seven items)^60^ with a maximum score of 9 and 10, respectively. The NOS was used for assessing the risk of bias in clinical trials because their baseline assessments were considered in this meta-analysis. According to the obtained NOS scores, case–control studies were classified into three levels: low quality (0–4 points), medium quality (5–6) and high quality (7–9 points) and cross sectional studies were classified into four levels: unsatisfactory (0–4 points), satisfactory (5–6 points), good (7–8 points) and very good (9–10 points) (as previously performed^61,62^). Any discrepancies were addressed by discussion to reach a consensus.

### Statistical analysis

The raw difference in means and its 95% confidence interval (CI) was calculated by five genetic comparison models: allelic model (A vs. G), dominant model (AA + GA vs. GG), recessive model (AA vs. GA + GG), homozygous model (AA vs. GG) and heterozygous model (GA vs. GG) to determine the effect size for the meta-analysis. The fixed-effect model was carried out to combine the subgroup-specific estimates when studies reported separate results across different subgroups and the pooled effect size was used for meta-analysis. A random-effects model which takes the between-study heterogeneity into account was used to derive weighted mean difference (WMD) as the summary estimate and its confidence limit. The heterogeneity between studies was evaluated using Cochran's Q test and the I-squared (I^2^) statistic (which is an estimate ranging from 0 to 100%). The heterogeneity was regarded as low, moderate, and high when the values of I^2^ were 25%, 50%, and exceeded 75%, respectively^63^. Subgroups analyses according to ethnicity, health status (heart disease, healthy and others), Hardy–Weinberg equilibrium, quality score (high or medium quality), design of the study (case–control or cross-sectional) and adjustment of confounders were performed to detect sources of between-study heterogeneity and also meta-regression analysis was used to identify potential sources of heterogeneity. Sensitivity analysis was used to assess whether the overall association depended on a specific study^64^. The presence of the publication bias was investigated by visual inspection of the funnel plots in case there were < 10 studies in each analysis, and also through statistical asymmetry tests (Begg's adjusted rank correlation and Egger`s tests) for meta-analysis of 10 or more effect sizes^65^. In the case of asymmetry, Duval and Tweedie’s trim and fill analysis was applied for more adjustment of publication bias^66^. All statistical analyses were performed by using STATA version 11.2 (StataCorp, College Station, TX). Two-tailed *p* ≤ 0.05 were considered statistically significant.

## Supplementary Information

Supplementary Information 1.

Supplementary Information 2.

## Data Availability

All data analyzed during the current study are available in Supplementary 2.

## Abbreviations

CVD: Cardiovascular disease
CD36: Cluster of differentiation 36
LCFA: Long-chain fatty acids
GWAS: Genome-wide association study
SNPs: Single-nucleotide polymorphisms
T2DM: Type 2 diabetes mellitus
BMI: Body mass index
HWE: Hardy–Weinberg equilibrium
SD: Standard deviation
WMD: Weighted mean difference
CI: Confidence interval
WC: Waist circumference
TC: Total cholesterol
TG: Triglyceride
HDL-C: High-density lipoprotein cholesterol
LDL-C: Low-density lipoprotein cholesterol
SBP: Systolic blood pressure
DBP: Diastolic blood pressure
FBG: Fasting blood glucose
MAF: Minor allele frequency
FOXO1: Forkhead box O1
ROS: Reactive oxygen species
pi3k-Akt: Phosphatidylinositol 3‑kinase-protein kinase B
PPAR-γ: Peroxisome proliferator-activated receptor
