# Supplementary material for: A systematic review and meta-analysis on the association between CD36 rs1761667 polymorphism and cardiometabolic risk factors in adults
Source: Sci Rep. 2022 Apr 8;12:5916. doi: 10.1038/s41598-022-09908-0 (PMC8993862; doi:10.1038/s41598-022-09908-0)
Supplement: Supplementary file 1 — Supplementary Information 1. [file 41598_2022_9908_MOESM1_ESM.docx]

A systematic review and meta-analysis on the association between CD36 rs1761667 polymorphism and cardiometabolic risk factors in adults

Zeinab Yazdanpanah, Hassan Mozaffari‐Khosravi, Masoud Mirzaei, Mohammad Hasan Sheikhha, Amin Salehi-Abargouei

**Legends to supplementary Tables**

**Supplementary Table S1-** Articles were excluded from the current systematic review and meta-analysis

**Supplementary Table S2-** Study quality and risk of bias assessment

**Supplementary Table S3-** The association between *CD36* rs1761667 polymorphism and body mass index based on several subgroups. All analyses were conducted using a random-effects model

**Supplementary Table S4-** The association between *CD36* rs1761667 polymorphism and total cholesterol based on several subgroups. All analyses were conducted using a random-effects model

**Supplementary Table S5-** The association between *CD36* rs1761667 polymorphism and triglyceride indices based on several subgroups. All analyses were conducted using a random-effects model

**Supplementary Table S6-** The association between *CD36* rs1761667 polymorphism and HDL cholesterol indices based on several subgroups. All analyses were conducted using a random-effects model

**Supplementary Table S7-** The association between *CD36* rs1761667 polymorphism and LDL cholesterol indices based on several subgroups. All analyses were conducted using a random-effects model

**Supplementary Table S8-** The association between *CD36* rs1761667 polymorphism and blood pressurebased on several subgroups. All analyses were conducted using a random-effects model

**Supplementary Table S9-** The association between *CD36* rs1761667 polymorphism and fasting blood glucose based on several subgroups. All analyses were conducted using a random-effects model

**Supplementary Table S10-** Sensitivity analysis

**Supplementary Table S11-** The strategy used for online database search

**Legends to supplementary Figure**

**Supplementary Figure S1-** Begg's funnel plots (with pseudo 95% CIs) depicting the effect sizes (difference in means) versus their standard errors (SEs)for studies which examined the association between genotype distribution of rs1761667 and waist circumference.

**Supplementary Figure S2-** Begg's funnel plots (with pseudo 95% CIs) depicting the effect sizes (difference in means) versus their standard errors (SEs)for studies which examined the association between genotype distribution of rs1761667 and systolic blood pressure.

**Supplementary Figure S3-** Begg's funnel plots (with pseudo 95% CIs) depicting the effect sizes (difference in means) versus their standard errors (SEs)for studies which examined the association between genotype distribution of rs1761667 and diastolic blood pressure.

**Supplementary Figure S4-** Begg's funnel plots (with pseudo 95% CIs) depicting the effect sizes (difference in means) versus their standard errors (SEs)for studies which examined the association between genotype distribution of rs1761667 and fasting blood glucose.

**Supplementary Figure S5-** Forest plot for studies which examined the association between genotype distribution of rs1761667 and body mass index (kg/m^2^) in adults

**Supplementary Figure S6-** Forest plot for studies which examined the association between genotype distribution of rs1761667 and waist circumference (cm) in adults.

**Supplementary Figure S7-** Forest plot for studies which examined the association between genotype distribution of rs1761667 and total cholesterol (mg/dl) in adults.

**Supplementary Figure S8-** Forest plot for studies which examined the association between genotype distribution of rs1761667 and triglyceride (mg/dl) in adults.

**Supplementary Figure S9-** Forest plot for studies which examined the association between genotype distribution of rs1761667 and HDL cholesterol (mg/dl) in adults.

**Supplementary Figure S10-** Forest plot for studies which examined the association between genotype distribution of rs1761667 and LDL cholesterol (mg/dl) in adults.

**Supplementary Figure S11-** Forest plot for studies which examined the association between genotype distribution of rs1761667 and systolic blood pressure (mmHg) in adults.

**Supplementary Figure S12-** Forest plot for studies which examined the association between genotype distribution of rs1761667 and diastolic blood pressure (mmHg) in adults.

**Supplementary Figure S13-** Forest plot for studies which examined the association between genotype distribution of rs1761667 and fasting blood glucose (mg/dl) in adults.

**Supplementary Figure S14-** Forest plot of the cumulative meta-analysis for studies which examined the association between genotype distribution of rs1761667 and body mass index (kg/m^2^) in adults

**Supplementary Figure S15-** Forest plot of the cumulative meta-analysis for studies which examined the association between genotype distribution of rs1761667 and waist circumference (cm) in adults.

**Supplementary Figure S16-** Forest plot of the cumulative meta-analysis for studies which examined the association between genotype distribution of rs1761667 and total cholesterol (mg/dl) in adults.

**Supplementary Figure S17-** Forest plot of the cumulative meta-analysis for studies which examined the association between genotype distribution of rs1761667 and triglyceride (mg/dl) in adults.

**Supplementary Figure S18-** Forest plot of the cumulative meta-analysis for studies which examined the association between genotype distribution of rs1761667 and HDL cholesterol (mg/dl) in adults.

**Supplementary Figure S19-** Forest plot of the cumulative meta-analysis for studies which examined the association between genotype distribution of rs1761667 and LDL cholesterol (mg/dl) in adults.

**Supplementary Figure S20-** Forest plot of the cumulative meta-analysis for studies which examined the association between genotype distribution of rs1761667 and systolic blood pressure (mmHg) in adults.

**Supplementary Figure S21-** Forest plot of the cumulative meta-analysis for studies which examined the association between genotype distribution of rs1761667 and diastolic blood pressure (mmHg) in adults.

**Supplementary Figure S22-** Forest plot of the cumulative meta-analysis for studies which examined the association between genotype distribution of rs1761667 and fasting blood glucose (mg/dl) in adults.

**Supplementary Table S1-** Articles were excluded from the current systematic review and meta-analysis

| **Reasons** | **References** |
| --- | --- |
| Reporting duplicate data | ^1,2^ |
| No data on the outcome variables | ^3-14^ |
| Conducting on pregnant women | ^15,16^ |
| Children and adolescents aged below 18 years | ^17,18^ |
| Not providing the sufficient data | ^19,20^ |

**Supplementary Table S2-** Study quality and risk of bias assessment

| **Results of quality assessment by the New Castle-Ottawa Scale for case-control studies^21^** | | | | | | | | | | | | | |
| --- | --- | --- | --- | --- | --- | --- | --- | --- | --- | --- | --- | --- | --- |
| **Author (year)** | **Selection** | | | |  | **Comparability** |  | **Exposure** | | | |  | **Overall quality**  **(total = 9)** |
|  | Case definition | Representativeness of the cases | Selection of controls | Definition of controls |  |  |  | Ascertainment of exposure | Same method for cases and controls | | Non-response rate |  |  |
| Bayoumyet al.) 2012(^22^ | * | * | - | * |  | - |  | * | * | | * |  | 6 |
| Boghdady et al. (2016)^23^ | * | * | * | * |  | ** |  | * | * | | * |  | 9 |
| Melis et al. (2017)^24^ | * | * | * | * |  | * |  | * | * | | * |  | 8 |
| Momeni-Moghaddam et al. (2019)^25^ | * | - | * | * |  | - |  | * | * | | * |  | 6 |
| Solakivi et al. (2015)^26^ | * | * | * | * |  | ** |  | * | * | | * |  | 9 |
| Yang et al. (2018)^16^ | * | - | * | * |  | - |  | * | * | | * |  | 6 |
| Yuan et al. (2015)^27^ | * | - | * | * |  | - |  | * | * | | * |  | 6 |
| Zhang et al. (2014)^28^ | * | - | * | * |  | - |  | * | * | | * |  | 6 |
| Zhang et al. (2015)^29^ | * | * | * | * |  | ** |  | * | * | | * |  | 9 |
| **Results of quality assessment by the Newcastle-Ottawa Scale adapted for cross-sectional studies^30^** | | | | | | | | | | | | | |
|  | **Selection** | | | |  | **Comparability** |  | **Outcome** | | | |  | **Overall quality**  **(total = 10)** |
|  | Representativeness  of the sample | Selected group  of users | Sample size | Diagnose |  |  |  | Ascertainment of  the method | | Statistical test | |  |  |
| Dalton et al. (2013)^31^ | * | * | - | ** |  | ** |  | ** | | * | |  | 9 |
| Dawczynski et al. (2013)^32^ | * | * | * | ** |  | - |  | ** | | * | |  | 8 |
| Fujii et al. (2019)^33^ | * | - | * | ** |  | - |  | ** | | * | |  | 7 |
| Maet al. (2004(^34^ | * | * | - | ** |  | ** |  | ** | | * | |  | 9 |
| Madden et al. (2008)^35^ | - | * | - | ** |  | - |  | ** | | * | |  | 6 |
| Mrizak et al. (2015)^36^ | * | * | - | ** |  | * |  | ** | | * | |  | 8 |
| Ramos-Lopez et al. (2013)^37^ | * | - | - | * |  | - |  | ** | | * | |  | 5 |
| Ramos-Lopez et al. (2016)^38^ | * | * | * | ** |  | ** |  | ** | | * | |  | 10 |
| Shen et al. (2017)^39^ | * | * | - | * |  | * |  | ** | | * | |  | 7 |

**Supplementary Table S3-**The association between *CD36* rs1761667 polymorphism and body mass index based on several subgroups. All analyses were conducted using a random-effects model^a^

|  | **No. of data-sets** | **No. of subjects** | **Meta-analysis** | |  | **Heterogeneity** | | | |  | **Meta-regression** | | |  |  |  |  |  |  |  |  |  |  |  |  |  |
| --- | --- | --- | --- | --- | --- | --- | --- | --- | --- | --- | --- | --- | --- | --- | --- | --- | --- | --- | --- | --- | --- | --- | --- | --- | --- | --- |
|  |  |  | **WMD^2^ (95%CI)** | **P _effect_** |  | ***Q* statistic** | **P _within_** | **I^2^ (%)** | **P _between group_** |  | **β** | **I^2^_residual (%)_** | **P-value** |  |  |  |  |  |  |  |  |  |  |  |  |  |
| **Body mass index (kg/m^2^)** | | | | | | | | | | | | | |  |  |  |  |  |  |  |  |  |  |  |  |  |
| **Allelic model (A vs. G)** | | | | | | | | | | | | | |  |  |  |  |  |  |  |  |  |  |  |  |  |
| Ethnicity | | | | | | | | | | | | | |  |  |  |  |  |  |  |  |  |  |  |  |  |
| Asian | 5 | 2334 | 0.06 (-0.21, 0.34) | 0.63 |  | 2.26 | 0.68 | 0.00 | 0.08 |  | 0.03 | 31.12 | 0.72 |  |  |  |  |  |  |  |  |  |  |  |  |  |
| Caucasian | 4 | 2110 | 0.00 (-0.83, 0.83) | 0.99 |  | 7.45 | 0.059 | 59.70 |  |  |  |  |  |  |  |  |  |  |  |  |  |  |  |  |  |  |
| Mix | 3 | 512 | 0.40 (-0.26, 1.07) | 0.23 |  | 1.38 | 0.50 | 0.00 |  |  |  |  |  |  |  |  |  |  |  |  |  |  |  |  |  |  |
| Participants’ health status | | | | | | | | | | | | | |  |  |  |  |  |  |  |  |  |  |  |  |  |
| Heart disease | 3 | 796 | 0.01 (-0.49, 0.52) | 0.95 |  | 1.21 | 0.54 | 0.00 | 0.16 |  | -0.02 | 27.81 | 0.72 |  |  |  |  |  |  |  |  |  |  |  |  |  |
| Healthy | 5 | 1328 | 0.28 (-0.12, 0.68) | 0.17 |  | 3.56 | 0.46 | 0.00 |  |  |  |  |  |  |  |  |  |  |  |  |  |  |  |  |  |  |
| Others | 4 | 2832 | -0.09 (-0.62, 0.43) | 0.72 |  | 7.60 | 0.05 | 60.50 |  |  |  |  |  |  |  |  |  |  |  |  |  |  |  |  |  |  |
| Hardy-Weinberg equilibrium | | | | | | | | | | | | | |  |  |  |  |  |  |  |  |  |  |  |  |  |
| Equilibrium | 8 | 3774 | 0.01 (-0.33, 0.37) | 0.92 |  | 12.80 | 0.07 | 45.30 | 0.77 |  | 0.05 | 34.32 | 0.58 |  |  |  |  |  |  |  |  |  |  |  |  |  |
| Disequilibrium | 1 | 348 | -0.22 (-1.26, 0.80) | 0.66 |  | - | - | - |  |  |  |  |  |  |  |  |  |  |  |  |  |  |  |  |  |  |
| NM | 4 | 834 | 0.16 (-0.45, 0.77) | 0.60 |  | 3.16 | 0.36 | 5.10 |  |  |  |  |  |  |  |  |  |  |  |  |  |  |  |  |  |  |
| Quality score | | | | | | | | | | | | | |  |  |  |  |  |  |  |  |  |  |  |  |  |
| High quality | 7 | 3396 | 0.14 (0.35, 0.63) | 0.57 |  | 12.73 | 0.04 | 52.90 | 0.47 |  | 0.01 | 31.39 | 0.85 |  |  |  |  |  |  |  |  |  |  |  |  |  |
| Medium quality | 5 | 1560 | 0.06 (-0.26, 0.39) | 0.68 |  | 2.74 | 0.60 | 0.00 |  |  |  |  |  |  |  |  |  |  |  |  |  |  |  |  |  |  |
| Design of study | | | | | | | | | | | | | |  |  |  |  |  |  |  |  |  |  |  |  |  |
| Case-control | 7 | 3162 | -0.01 (-0.43, 0.40) | 0.96 |  | 11.83 | 0.06 | 49.30 | 0.41 |  | 0.03 | 31.37 | 0.70 |  |  |  |  |  |  |  |  |  |  |  |  |  |
| Cross-sectional | 5 | 1794 | 0.02 (-0.25, 0.31) | 0.60 |  | 3.49 | 0.47 | 0.00 |  |  |  |  |  |  |  |  |  |  |  |  |  |  |  |  |  |  |
| Adjustment of confounders | | | | | | | | | | | | | |  |  |  |  |  |  |  |  |  |  |  |  |  |
| Adjusted | 6 | 2406 | 0.29 (-0.41, 0.99) | 0.41 |  | 12.53 | 0.02 | 60.10 | 0.42 |  | 0.01 | 31.39 | 0.87 |  |  |  |  |  |  |  |  |  |  |  |  |  |
| Unadjusted | 6 | 2550 | 0.04 (-0.22, 0.31) | 0.76 |  | 2.82 | 0.72 | 0.00 |  |  |  |  |  |  |  |  |  |  |  |  |  |  |  |  |  |  |
| **Dominant model (AA+GA vs. GG)** | | | | | | | | | | | | | |  |  |  |  |  |  |  |  |  |  |  |  |  |
| Ethnicity | | | | | | | | | | | | | |  |  |  |  |  |  |  |  |  |  |  |  |  |
| Asian | 5 | 1167 | 0.26 (-0.11, 0.64) | 0.17 |  | 2.34 | 0.67 | 0.00 | 0.12 |  | 0.16 | 18.46 | 0.23 |  |  |  |  |  |  |  |  |  |  |  |  |  |
| Caucasian | 4 | 1055 | -0.05 (-1.08, 0.97) | 0.91 |  | 4.71 | 0.19 | 36.30 |  |  |  |  |  |  |  |  |  |  |  |  |  |  |  |  |  |  |
| Mix | 3 | 256 | 1.05 (-0.17, 2.27) | 0.09 |  | 2.19 | 0.33 | 8.50 |  |  |  |  |  |  |  |  |  |  |  |  |  |  |  |  |  |  |
| Participants’ health status | | | | | | | | | | | | | |  |  |  |  |  |  |  |  |  |  |  |  |  |
| Heart disease | 3 | 398 | 0.70 (-0.57, 1.98) | 0.28 |  | 4.03 | 0.13 | 50.40 | 0.18 |  | 0.05 | 24.73 | 0.54 |  |  |  |  |  |  |  |  |  |  |  |  |  |
| Healthy | 5 | 664 | 0.47 (-0.10, 1.04) | 0.10 |  | 1.19 | 0.87 | 0.00 |  |  |  |  |  |  |  |  |  |  |  |  |  |  |  |  |  |  |
| Others | 4 | 1416 | -0.06 (-0.68, 0.55) | 0.84 |  | 4.76 | 0.19 | 37.00 |  |  |  |  |  |  |  |  |  |  |  |  |  |  |  |  |  |  |
| Hardy-Weinberg equilibrium | | | | | | | | | | | | | |  |  |  |  |  |  |  |  |  |  |  |  |  |
| Equilibrium | 8 | 1887 | 0.19 (-0.25, 0.64) | 0.39 |  | 9.88 | 0.19 | 29.10 | 0.68 |  | 0.20 | 18.46 | 0.16 |  |  |  |  |  |  |  |  |  |  |  |  |  |
| Disequilibrium | 1 | 174 | -0.18 (-2.09, 1.71) | 0.84 |  | - | - | - |  |  |  |  |  |  |  |  |  |  |  |  |  |  |  |  |  |  |
| NM | 4 | 417 | 0.60 (-0.43, 1.64) | 0.25 |  | 3.08 | 0.38 | 2.60 |  |  |  |  |  |  |  |  |  |  |  |  |  |  |  |  |  |  |
| Quality score | | | | | | | | | | | | | |  |  |  |  |  |  |  |  |  |  |  |  |  |
| High quality | 7 | 1698 | 0.30 (-0.42, 1.02) | 0.41 |  | 10.65 | 0.10 | 43.70 | 0.33 |  | 0.16 | 13.54 | 0.20 |  |  |  |  |  |  |  |  |  |  |  |  |  |
| Medium quality | 5 | 780 | 0.37 (-0.09, 0.84) | 0.12 |  | 1.18 | 0.77 | 0.00 |  |  |  |  |  |  |  |  |  |  |  |  |  |  |  |  |  |  |
| Design of study | | | | | | | | | | | | | |  |  |  |  |  |  |  |  |  |  |  |  |  |
| Case-control | 7 | 1581 | 0.25 (-0.37, 0.87) | 0.43 |  | 11.05 | 0.08 | 45.70 | 0.99 |  | 0.16 | 18.84 | 0.26 |  |  |  |  |  |  |  |  |  |  |  |  |  |
| Cross-sectional | 5 | 897 | 0.20 (-0.29, 0.70) | 0.42 |  | 2.35 | 0.67 | 0.00 |  |  |  |  |  |  |  |  |  |  |  |  |  |  |  |  |  |  |
| Adjustment of confounders | | | | | | | | | | | | | |  |  |  |  |  |  |  |  |  |  |  |  |  |
| Adjusted | 6 | 1203 | 0.57 (-0.50, 1.65) | 0.29 |  | 10.65 | 0.05 | 53.10 | 0.61 |  | 0.13 | 16.57 | 0.25 |  |  |  |  |  |  |  |  |  |  |  |  |  |
| Unadjusted | 6 | 1275 | 0.24 (-0.12, 0.61) | 0.18 |  | 2.50 | 0.77 | 0.00 |  |  |  |  |  |  |  |  |  |  |  |  |  |  |  |  |  |  |
| **Recessive model (AA vs. GA + GG)** | | | | | | | | | | | | | |  |  |  |  |  |  |  |  |  |  |  |  |  |
| Ethnicity | | | | | | | | | | | | | |  |  |  |  |  |  |  |  |  |  |  |  |  |
| Asian | 5 | 1167 | -0.36 (-0.91, 0.19) | 0.20 |  | 3.85 | 0.42 | 0.00 | 0.38 |  | -0.11 | 45.03 | 0.47 |  |  |  |  |  |  |  |  |  |  |  |  |  |
| Caucasian | 4 | 1055 | -0.02 (-1.48, 1.43) | 0.97 |  | 8.64 | 0.03 | 65.30 |  |  |  |  |  |  |  |  |  |  |  |  |  |  |  |  |  |  |
| Mix | 3 | 256 | 0.19 (-1.43, 1.83) | 0.81 |  | 4.28 | 0.11 | 53.30 |  |  |  |  |  |  |  |  |  |  |  |  |  |  |  |  |  |  |
| Participants’ health status | | | | | | | | | | | | | |  |  |  |  |  |  |  |  |  |  |  |  |  |
| Heart disease | 3 | 398 | -1.36 (-2.53, -0.20) | **0.02** |  | 0.04 | 0.98 | 0.00 | 0.06 |  | -.018 | 36.19 | 0.09 |  |  |  |  |  |  |  |  |  |  |  |  |  |
| Healthy | 5 | 664 | 0.21 (-0.75, 1.19) | 0.66 |  | 7.02 | 0.13 | 43.00 |  |  |  |  |  |  |  |  |  |  |  |  |  |  |  |  |  |  |
| Others | 4 | 1416 | -0.30 (-1.11, 0.50) | 0.45 |  | 6.32 | 0.09 | 52.5 |  |  |  |  |  |  |  |  |  |  |  |  |  |  |  |  |  |  |
| Hardy-Weinberg equilibrium | | | | | | | | | | | | | |  |  |  |  |  |  |  |  |  |  |  |  |  |
| Equilibrium | 8 | 1887 | -0.34 (-0.89, 0.21) | 0.23 |  | 9.86 | 0.19 | 29.00 | 0.74 |  | -0.10 | 47.32 | 0.56 |  |  |  |  |  |  |  |  |  |  |  |  |  |
| Disequilibrium | 1 | 174 | -0.98 (-3.11, 1.14) | 0.36 |  | - | - | - |  |  |  |  |  |  |  |  |  |  |  |  |  |  |  |  |  |  |
| NM | 4 | 417 | 0.10 (-1.63, 1.83) | 0.90 |  | 8.23 | 0.04 | 63.60 |  |  |  |  |  |  |  |  |  |  |  |  |  |  |  |  |  |  |
| Quality score | | | | | | | | | | | | | |  |  |  |  |  |  |  |  |  |  |  |  |  |
| High quality | 7 | 1698 | 0.03 (-0.86, 0.93) | 0.93 |  | 14.67 | 0.02 | 59.10 | 0.71 |  | -0.24 | 41.07 | 0.21 |  |  |  |  |  |  |  |  |  |  |  |  |  |
| Medium quality | 5 | 780 | -0.47 (-1.10, 0.16) | 0.14 |  | 3.90 | 0.41 | 0.00 |  |  |  |  |  |  |  |  |  |  |  |  |  |  |  |  |  |  |
| Design of study | | | | | | | | | | | | | |  |  |  |  |  |  |  |  |  |  |  |  |  |
| Case-control | 7 | 1581 | -0.50 (-1.33, 0.32) | 0.23 |  | 12.91 | 0.04 | 53.50 | 0.19 |  | -0.14 | 46.37 | 0.49 |  |  |  |  |  |  |  |  |  |  |  |  |  |
| Cross-sectional | 5 | 897 | -0.29 (-0.84, 0.25) | 0.90 |  | 4.09 | 0.39 | 2.30 |  |  |  |  |  |  |  |  |  |  |  |  |  |  |  |  |  |  |
| Adjustment of confounders | | | | | | | | | | | | | |  |  |  |  |  |  |  |  |  |  |  |  |  |
| Adjusted | 6 | 1203 | 0.21 (-0.98, 1.42) | 0.72 |  | 14.64 | 0.01 | 65.80 | 0.89 |  | -0.21 | 41.86 | 0.23 |  |  |  |  |  |  |  |  |  |  |  |  |  |
| Unadjusted | 6 | 1275 | -0.40 (-0.92, 0.12) | 0.13 |  | 4.06 | 0.54 | 0.00 |  |  |  |  |  |  |  |  |  |  |  |  |  |  |  |  |  |  |
| **Homozygous model (AA vs. GG)** | | | | | | | | | | | | | |  |  |  |  |  |  |  |  |  |  |  |  |  |
| Ethnicity | | | | | | | | | | | | | |  |  |  |  |  |  |  |  |  |  |  |  |  |
| Asian | 5 | 616 | -0.18 (-0.78, 0.40) | 0.53 |  | 2.79 | 0.59 | 0.00 | 0.15 |  | 0.01 | 37.03 | 0.94 |  |  |  |  |  |  |  |  |  |  |  |  |  |
| Caucasian | 4 | 519 | 0.14 (-1.67, 1.94) | 0.88 |  | 8.85 | 0.03 | 66.10 |  |  |  |  |  |  |  |  |  |  |  |  |  |  |  |  |  |  |
| Mix | 3 | 120 | 0.87 (-0.48, 2.23) | 0.20 |  | 1.59 | 0.45 | 0.00 |  |  |  |  |  |  |  |  |  |  |  |  |  |  |  |  |  |  |
| Participants’ health status | | | | | | | | | | | | | |  |  |  |  |  |  |  |  |  |  |  |  |  |
| Heart disease | 3 | 155 | -0.77 (-2.11, 0.55) | 0.25 |  | 1.14 | 0.56 | 0.00 | 0.14 |  | -0.12 | 29.32 | 0.33 |  |  |  |  |  |  |  |  |  |  |  |  |  |
| Healthy | 5 | 339 | 0.45 (-0.45, 1.36) | 0.32 |  | 4.62 | 0.32 | 13.30 |  |  |  |  |  |  |  |  |  |  |  |  |  |  |  |  |  |  |
| Others | 4 | 761 | -0.27 (-1.30, 0.74) | 0.59 |  | 7.28 | 0.06 | 58.80 |  |  |  |  |  |  |  |  |  |  |  |  |  |  |  |  |  |  |
| Hardy-Weinberg equilibrium | | | | | | | | | | | | | |  |  |  |  |  |  |  |  |  |  |  |  |  |
| Equilibrium | 8 | 1004 | -0.20 (-0.89, 0.47) | 0.55 |  | 11.14 | 0.13 | 37.10 | 0.51 |  | 0.05 | 37.76 | 0.80 |  |  |  |  |  |  |  |  |  |  |  |  |  |
| Disequilibrium | 1 | 51 | -1.04 (-3.68, 1.58) | 0.43 |  | - | - | - |  |  |  |  |  |  |  |  |  |  |  |  |  |  |  |  |  |  |
| NM | 4 | 200 | 0.55 (-1.04, 2.15) | 0.49 |  | 4.53 | 0.20 | 33.80 |  |  |  |  |  |  |  |  |  |  |  |  |  |  |  |  |  |  |
| Quality score | | | | | | | | | | | | | |  |  |  |  |  |  |  |  |  |  |  |  |  |
| High quality | 7 | 887 | 0.33 (-0.74, 1.41) | 0.54 |  | 13.99 | 0.03 | 57.10 | 0.96 |  | -0.11 | 35.31 | 0.57 |  |  |  |  |  |  |  |  |  |  |  |  |  |
| Medium quality | 5 | 368 | -0.22 (-0.93, 0.47) | 0.52 |  | 2.95 | 0.56 | 0.00 |  |  |  |  |  |  |  |  |  |  |  |  |  |  |  |  |  |  |
| Design of study | | | | | | | | | | | | | |  |  |  |  |  |  |  |  |  |  |  |  |  |
| Case-control | 7 | 744 | -0.29 (-1.21, 0.62) | 0.52 |  | 11.76 | 0.06 | 49.00 | 0.25 |  | -0.01 | 37.34 | 0.94 |  |  |  |  |  |  |  |  |  |  |  |  |  |
| Cross-sectional | 5 | 511 | 0.11 (-0.61, 0.84) | 0.75 |  | 3.87 | 0.42 | 0.00 |  |  |  |  |  |  |  |  |  |  |  |  |  |  |  |  |  |  |
| Adjustment of confounders | | | | | | | | | | | | | |  |  |  |  |  |  |  |  |  |  |  |  |  |
| Adjusted | 6 | 582 | 0.68 (-0.81, 2.18) | 0.37 |  | 13.98 | 0.01 | 64.20 | 0.99 |  | -0.10 | 35.29 | 0.57 |  |  |  |  |  |  |  |  |  |  |  |  |  |
| Unadjusted | 6 | 673 | -0.21 (-0.79, 0.35) | 0.45 |  | 2.95 | 0.70 | 0.00 |  |  |  |  |  |  |  |  |  |  |  |  |  |  |  |  |  |  |
| **Heterozygous model (GA vs. GG)** | | | | | | | | | | | | | |  |  |  |  |  |  |  |  |  |  |  |  |  |
| Ethnicity | | | | | | | | | | | | | |  |  |  |  |  |  |  |  |  |  |  |  |  |
| Asian | 5 | 1052 | 0.40 (-0.004, 0.80) | 0.052 |  | 3.04 | 0.55 | 0.00 | 0.20 |  | 0.21 | 19.37 | 0.12 |  |  |  |  |  |  |  |  |  |  |  |  |  |
| Caucasian | 4 | 783 | -0.08 (-0.97, 0.81) | 0.86 |  | 3.58 | 0.31 | 16.20 |  |  |  |  |  |  |  |  |  |  |  |  |  |  |  |  |  |  |
| Mix | 3 | 193 | 1.112 -0.47, 2.70) | 0.17 |  | 3.26 | 0.19 | 38.60 |  |  |  |  |  |  |  |  |  |  |  |  |  |  |  |  |  |  |
| Participants’ health status | | | | | | | | | | | | | |  |  |  |  |  |  |  |  |  |  |  |  |  |
| Heart disease | 3 | 356 | 0.99 (-0.41, 2.40) | 0.16 |  | 4.73 | 0.09 | 57.70 | 0.13 |  | 0.11 | 27.43 | 0.28 |  |  |  |  |  |  |  |  |  |  |  |  |  |
| Healthy | 5 | 539 | 0.57 (-0.06, 1.20) | 0.07 |  | 1.54 | 0.81 | 0.00 |  |  |  |  |  |  |  |  |  |  |  |  |  |  |  |  |  |  |
| Others | 4 | 1133 | 0.01 (-0.45, 0.48) | 0.95 |  | 2.81 | 0.42 | 0.00 |  |  |  |  |  |  |  |  |  |  |  |  |  |  |  |  |  |  |
| Hardy-Weinberg equilibrium | | | | | | | | | | | | | |  |  |  |  |  |  |  |  |  |  |  |  |  |
| Equilibrium | 8 | 1546 | 0.32 (-0.10, 0.74) | 0.13 |  | 8.23 | 0.31 | 15.00 | 0.74 |  | 0.10 | 21.49 | 0.28 |  |  |  |  |  |  |  |  |  |  |  |  |  |
| Disequilibrium | 1 | 158 | -0.07 (-2.00, 1.85) | 0.93 |  | - | - | - |  |  |  |  |  |  |  |  |  |  |  |  |  |  |  |  |  |  |
| NM | 4 | 324 | 0.71 (-0.61, 2.03) | 0.29 |  | 4.37 | 0.22 | 31.30 |  |  |  |  |  |  |  |  |  |  |  |  |  |  |  |  |  |  |
| Quality score | | | | | | | | | | | | | |  |  |  |  |  |  |  |  |  |  |  |  |  |
| High quality | 7 | 1356 | 0.28 (-0.43, 1.00) | 0.43 |  | 9.54 | 0.14 | 37.10 | 0.17 |  | 0.25 | 5.83 | 0.056 |  |  |  |  |  |  |  |  |  |  |  |  |  |
| Medium quality | 5 | 672 | 0.58 (0.07, 1.09) | **0.02** |  | 1.70 | 0.79 | 0.00 |  |  |  |  |  |  |  |  |  |  |  |  |  |  |  |  |  |  |
| Design of study | | | | | | | | | | | | | |  |  |  |  |  |  |  |  |  |  |  |  |  |
| Case-control | 7 | 1271 | 0.42 (-0.23, 1.09) | 0.20 |  | 11.17 | 0.08 | 46.30 | 0.69 |  | 0.22 | 20.66 | 0.14 |  |  |  |  |  |  |  |  |  |  |  |  |  |
| Cross-sectional | 5 | 757 | 0.24 (-0.29, 0.77) | 0.37 |  | 1.71 | 0.78 | 0.00 |  |  |  |  |  |  |  |  |  |  |  |  |  |  |  |  |  |  |
| Adjustment of confounders | | | | | | | | | | | | | |  |  |  |  |  |  |  |  |  |  |  |  |  |
| Adjusted | 6 | 898 | 0.51 (-0.55, 1.58) | 0.34 |  | 9.53 | 0.09 | 47.60 | 0.51 |  | 0.20 | 13.00 | 0.09 |  |  |  |  |  |  |  |  |  |  |  |  |  |
| Unadjusted | 6 | 1130 | 0.39 (-0.003, 0.79) | 0.052 |  | 3.08 | 0.68 | 0.00 |  |  |  |  |  |  |  |  |  |  |  |  |  |  |  |  |  |  |

^a^ All analysis was done using random effects model. WMD, weighted mean difference; 95% CI, 95% confidence interval; NM, not mention.

**Supplementary Table S4-**The association between *CD36* rs1761667 polymorphism and total cholesterol based on several subgroups. All analyses were conducted using a random-effects model^a^

|  | **No. of data-sets** | **No. of subjects** | **Meta-analysis** | |  | **Heterogeneity** | | | |  | **Meta-regression** | | |  |  |  |  |  |  |  |  |  |  |  |  |  |  |  |  |  |  |  |  |  |  |  |  |  |  |  |  |
| --- | --- | --- | --- | --- | --- | --- | --- | --- | --- | --- | --- | --- | --- | --- | --- | --- | --- | --- | --- | --- | --- | --- | --- | --- | --- | --- | --- | --- | --- | --- | --- | --- | --- | --- | --- | --- | --- | --- | --- | --- | --- |
|  |  |  | **WMD^2^ (95%CI)** | **P _effect_** |  | ***Q* statistic** | **P _within_** | **I^2^ (%)** | **P _between group_** |  | **β** | **I^2^_residual (%)_** | **P-value** |  |  |  |  |  |  |  |  |  |  |  |  |  |  |  |  |  |  |  |  |  |  |  |  |  |  |  |  |
| **Total cholesterol (mg/dl)** | | | | | | | | | | | | | |  |  |  |  |  |  |  |  |  |  |  |  |  |  |  |  |  |  |  |  |  |  |  |  |  |  |  |  |
| **Allelic model (A vs. G)** | | | | | | | | | | | | | |  |  |  |  |  |  |  |  |  |  |  |  |  |  |  |  |  |  |  |  |  |  |  |  |  |  |  |  |
| Ethnicity | | | | | | | | | | | | | |  |  |  |  |  |  |  |  |  |  |  |  |  |  |  |  |  |  |  |  |  |  |  |  |  |  |  |  |
| Asian | 4 | 3644 | 1.04 (0.88, 1.20) | **<0.001** |  | 2.91 | 0.40 | 0.00 | 0.97 |  | -0.38 | 71.94 | 0.65 |  |  |  |  |  |  |  |  |  |  |  |  |  |  |  |  |  |  |  |  |  |  |  |  |  |  |  |  |
| Caucasian | 2 | 2556 | 0.94 (-3.47, 5.36) | 0.67 |  | 2.09 | 0.14 | 52.10 |  |  |  |  |  |  |  |  |  |  |  |  |  |  |  |  |  |  |  |  |  |  |  |  |  |  |  |  |  |  |  |  |  |
| Mix | 5 | 1310 | -2.46 (-9.50, 4.57) | 0.49 |  | 23.18 | <0.001 | 82.70 |  |  |  |  |  |  |  |  |  |  |  |  |  |  |  |  |  |  |  |  |  |  |  |  |  |  |  |  |  |  |  |  |  |
| Participants’ health status | | | | | | | | | | | | | |  |  |  |  |  |  |  |  |  |  |  |  |  |  |  |  |  |  |  |  |  |  |  |  |  |  |  |  |
| Heart disease | 3 | 796 | -1.45 (-7.18, 4.26) | 0.61 |  | 1.80 | 0.40 | 0.00 | 0.41 |  | -0.53 | 65.55 | 0.51 |  |  |  |  |  |  |  |  |  |  |  |  |  |  |  |  |  |  |  |  |  |  |  |  |  |  |  |  |
| Healthy | 3 | 1954 | 0.41 (-1.60, 2.44) | 0.09 |  | 7.91 | 0.01 | 74.70 |  |  |  |  |  |  |  |  |  |  |  |  |  |  |  |  |  |  |  |  |  |  |  |  |  |  |  |  |  |  |  |  |  |
| Others | 5 | 4760 | -3.80 (-8.87, 1.25) | 0.14 |  | 16.74 | 0.002 | 76.10 |  |  |  |  |  |  |  |  |  |  |  |  |  |  |  |  |  |  |  |  |  |  |  |  |  |  |  |  |  |  |  |  |  |
| Hardy-Weinberg equilibrium | | | | | | | | | | | | | |  |  |  |  |  |  |  |  |  |  |  |  |  |  |  |  |  |  |  |  |  |  |  |  |  |  |  |  |
| Equilibrium | 9 | 6662 | -0.24 (-2.23, 1.74) | 0.80 |  | 19.61 | 0.01 | 59.20 | 0.03 |  | 1.04 | 54.98 | **<0.001** |  |  |  |  |  |  |  |  |  |  |  |  |  |  |  |  |  |  |  |  |  |  |  |  |  |  |  |  |
| Disequilibrium | 1 | 348 | 1.54 (-6.14, 9.24) | 0.69 |  | - | - | - |  |  |  |  |  |  |  |  |  |  |  |  |  |  |  |  |  |  |  |  |  |  |  |  |  |  |  |  |  |  |  |  |  |
| NM | 2 | 500 | 3.19 (-10.43, 16.81) | 0.64 |  | 2.10 | 0.14 | 52.40 |  |  |  |  |  |  |  |  |  |  |  |  |  |  |  |  |  |  |  |  |  |  |  |  |  |  |  |  |  |  |  |  |  |
| Quality score | | | | | | | | | | | | | |  |  |  |  |  |  |  |  |  |  |  |  |  |  |  |  |  |  |  |  |  |  |  |  |  |  |  |  |
| High quality | 6 | 5920 | 1.80 (-0.96, 4.58) | 0.20 |  | 11.84 | 0.03 | 57.80 | 0.24 |  | -1.08 | 69.47 | 0.39 |  |  |  |  |  |  |  |  |  |  |  |  |  |  |  |  |  |  |  |  |  |  |  |  |  |  |  |  |
| Medium quality | 5 | 1590 | -4.23 (-10.57, 2.10) | 0.19 |  | 15.06 | 0.005 | 73.40 |  |  |  |  |  |  |  |  |  |  |  |  |  |  |  |  |  |  |  |  |  |  |  |  |  |  |  |  |  |  |  |  |  |
| Design of study | | | | | | | | | | | | | |  |  |  |  |  |  |  |  |  |  |  |  |  |  |  |  |  |  |  |  |  |  |  |  |  |  |  |  |
| Case-control | 7 | 5410 | -2.89 (-6.90, 1.12) | 0.15 |  | 18.21 | 0.006 | 67.10 | 0.35 |  | 0.20 | 63.75 | 0.86 |  |  |  |  |  |  |  |  |  |  |  |  |  |  |  |  |  |  |  |  |  |  |  |  |  |  |  |  |
| Cross-sectional | 4 | 2100 | 2.89 (-1.09, 6.88) | 0.15 |  | 9.18 | 0.02 | 67.30 |  |  |  |  |  |  |  |  |  |  |  |  |  |  |  |  |  |  |  |  |  |  |  |  |  |  |  |  |  |  |  |  |  |
| Adjustment of confounders | | | | | | | | | | | | | |  |  |  |  |  |  |  |  |  |  |  |  |  |  |  |  |  |  |  |  |  |  |  |  |  |  |  |  |
| Adjusted | 5 | 5514 | 1.04 (0.88, 1.20) | **<0.001** |  | 3.92 | 0.41 | 0.00 | 0.87 |  | -0.59 | 67.19 | 0.61 |  |  |  |  |  |  |  |  |  |  |  |  |  |  |  |  |  |  |  |  |  |  |  |  |  |  |  |  |
| Unadjusted | 6 | 1996 | -1.67 (-7.11, 3.77) | 0.54 |  | 24.28 | <0.001 | 79.40 |  |  |  |  |  |  |  |  |  |  |  |  |  |  |  |  |  |  |  |  |  |  |  |  |  |  |  |  |  |  |  |  |  |
| **Dominant model (AA+GA vs. GG)** | | | | | | | | | | | | | |  |  |  |  |  |  |  |  |  |  |  |  |  |  |  |  |  |  |  |  |  |  |  |  |  |  |  |  |
| Ethnicity | | | | | | | | | | | | | |  |  |  |  |  |  |  |  |  |  |  |  |  |  |  |  |  |  |  |  |  |  |  |  |  |  |  |  |
| Asian | 4 | 1822 | -3.45 (-11.35, 4.44) | 0.39 |  | 6.36 | 0.09 | 52.80 | 0.005 |  | 0.42 | 49.52 | 0.66 |  |  |  |  |  |  |  |  |  |  |  |  |  |  |  |  |  |  |  |  |  |  |  |  |  |  |  |  |
| Caucasian | 2 | 1278 | 1.27 (-5.46, 8.01) | 0.71 |  | 1.51 | 0.21 | 33.70 |  |  |  |  |  |  |  |  |  |  |  |  |  |  |  |  |  |  |  |  |  |  |  |  |  |  |  |  |  |  |  |  |  |
| Mix | 5 | 655 | -3.45 (-11.35, 4.44) | 0.99 |  | 10.72 | 0.03 | 62.70 |  |  |  |  |  |  |  |  |  |  |  |  |  |  |  |  |  |  |  |  |  |  |  |  |  |  |  |  |  |  |  |  |  |
| Participants’ health status | | | | | | | | | | | | | |  |  |  |  |  |  |  |  |  |  |  |  |  |  |  |  |  |  |  |  |  |  |  |  |  |  |  |  |
| Heart disease | 3 | 398 | -7.17 (-16.16, 1.81) | 0.11 |  | 0.74 | 0.69 | 9.50 | <0.001 |  | 0.08 | 68.64 | 0.93 |  |  |  |  |  |  |  |  |  |  |  |  |  |  |  |  |  |  |  |  |  |  |  |  |  |  |  |  |
| Healthy | 3 | 977 | 3.58 (-3.82, 10.99) | 0.34 |  | 7.87 | 0.02 | 74.60 |  |  |  |  |  |  |  |  |  |  |  |  |  |  |  |  |  |  |  |  |  |  |  |  |  |  |  |  |  |  |  |  |  |
| Others | 5 | 2380 | 1.18 (-1.58, 3.94) | 0.40 |  | 4.42 | 0.35 | 0.00 |  |  |  |  |  |  |  |  |  |  |  |  |  |  |  |  |  |  |  |  |  |  |  |  |  |  |  |  |  |  |  |  |  |
| Hardy-Weinberg equilibrium | | | | | | | | | | | | | |  |  |  |  |  |  |  |  |  |  |  |  |  |  |  |  |  |  |  |  |  |  |  |  |  |  |  |  |
| Equilibrium | 9 | 3331 | 1.74 (-2.27, 5.77) | 0.39 |  | 25.46 | 0.001 | 68.60 | 0.20 |  | -0.65 | 67.08 | 0.66 |  |  |  |  |  |  |  |  |  |  |  |  |  |  |  |  |  |  |  |  |  |  |  |  |  |  |  |  |
| Disequilibrium | 1 | 174 | -2.28 (-15.17, 10.61) | 0.72 |  | - | - | - |  |  |  |  |  |  |  |  |  |  |  |  |  |  |  |  |  |  |  |  |  |  |  |  |  |  |  |  |  |  |  |  |  |
| NM | 2 | 250 | -4.74 (-12.87, 3.38) | 0.25 |  | 0.02 | 0.89 | 0.00 |  |  |  |  |  |  |  |  |  |  |  |  |  |  |  |  |  |  |  |  |  |  |  |  |  |  |  |  |  |  |  |  |  |
| Quality score | | | | | | | | | | | | | |  |  |  |  |  |  |  |  |  |  |  |  |  |  |  |  |  |  |  |  |  |  |  |  |  |  |  |  |
| High quality | 6 | 2960 | 2.05 (1.87, 2.24) | **<0.001** |  | 4.38 | 0.49 | 0.00 | 0.002 |  | -0.21 | 54.65 | 0.89 |  |  |  |  |  |  |  |  |  |  |  |  |  |  |  |  |  |  |  |  |  |  |  |  |  |  |  |  |
| Medium quality | 5 | 795 | -4.65 (-16.39, 7.08) | 0.43 |  | 15.14 | 0.004 | 73.60 |  |  |  |  |  |  |  |  |  |  |  |  |  |  |  |  |  |  |  |  |  |  |  |  |  |  |  |  |  |  |  |  |  |
| Design of study | | | | | | | | | | | | | |  |  |  |  |  |  |  |  |  |  |  |  |  |  |  |  |  |  |  |  |  |  |  |  |  |  |  |  |
| Case-control | 7 | 2705 | -1.94 (-6.67, 2.79) | 0.42 |  | 9.17 | 0.16 | 34.60 | 0.001 |  | 0.86 | 51.71 | 0.52 |  |  |  |  |  |  |  |  |  |  |  |  |  |  |  |  |  |  |  |  |  |  |  |  |  |  |  |  |
| Cross-sectional | 4 | 1050 | 3.49 (-3.29, 10.29) | 0.31 |  | 8.09 | 0.04 | 62.90 |  |  |  |  |  |  |  |  |  |  |  |  |  |  |  |  |  |  |  |  |  |  |  |  |  |  |  |  |  |  |  |  |  |
| Adjustment of confounders | | | | | | | | | | | | | |  | | |  | | |  |  |  |  |  |  |  |  |  |  |  |  |  |  |  |  |  |  |  |  |  |  |
| Adjusted | 5 | 2757 | 2.06 (1.87, 2.24) | **<0.001** |  | 1.99 | 0.73 | 0.00 | 0.01 |  | -0.45 | 57.93 | 0.76 |  |  |  |  |  |  |  |  |  |  |  |  |  |  |  |  |  |  |  |  |  |  |  |  |  |  |  |  |
| Unadjusted | 6 | 998 | -4.18 (-13.58, 5.22) | 0.38 |  | 20.96 | 0.001 | 76.10 |  |  |  |  |  |  |  |  |  |  |  |  |  |  |  |  |  |  |  |  |  |  |  |  |  |  |  |  |  |  |  |  |  |
| **Recessive model (AA vs. GA + GG)** | | | | | | | | | | | | | |  |  |  |  |  |  |  |  |  |  |  |  |  |  |  |  |  |  |  |  |  |  |  |  |  |  |  |  |
| Ethnicity | | | | | | | | | | | | | |  |  |  |  |  |  |  |  |  |  |  |  |  |  |  |  |  |  |  |  |  |  |  |  |  |  |  |  |
| Asian | 4 | 1822 | 0.26 (-2.18, 2.71) | 0.83 |  | 3.10 | 0.37 | 3.20 | 0.01 |  | -1.79 | 88.85 | 0.41 |  |  |  |  |  |  |  |  |  |  |  |  |  |  |  |  |  |  |  |  |  |  |  |  |  |  |  |  |
| Caucasian | 2 | 1278 | 1.04 (-4.19, 6.28) | 0.69 |  | 1.22 | 0.27 | 17.80 |  |  |  |  |  |  |  |  |  |  |  |  |  |  |  |  |  |  |  |  |  |  |  |  |  |  |  |  |  |  |  |  |  |
| Mix | 5 | 655 | -9.21 (-26.81, 8.39) | 0.30 |  | 79.59 | <0.001 | 95.00 |  |  |  |  |  |  |  |  |  |  |  |  |  |  |  |  |  |  |  |  |  |  |  |  |  |  |  |  |  |  |  |  |  |
| Participants’ health status | | | | | | | | | | | | | |  |  |  |  |  |  |  |  |  |  |  |  |  |  |  |  |  |  |  |  |  |  |  |  |  |  |  |  |
| Heart disease | 3 | 398 | 1.39 (-17.74, 20.54) | 0.88 |  | 5.05 | 0.08 | 60.4 | 0.38 |  | -1.58 | 89.13 | 0.44 |  |  |  |  |  |  |  |  |  |  |  |  |  |  |  |  |  |  |  |  |  |  |  |  |  |  |  |  |
| Healthy | 3 | 977 | 7.19 (-8.36, 22.75) | 0.36 |  | 48.84 | <0.001 | 95.90 |  |  |  |  |  |  |  |  |  |  |  |  |  |  |  |  |  |  |  |  |  |  |  |  |  |  |  |  |  |  |  |  |  |
| Others | 5 | 2380 | -9.64 (-20.29, 1.00) | 0.07 |  | 36.09 | <0.001 | 88.90 |  |  |  |  |  |  |  |  |  |  |  |  |  |  |  |  |  |  |  |  |  |  |  |  |  |  |  |  |  |  |  |  |  |
| Hardy-Weinberg equilibrium | | | | | | | | | | | | | |  |  |  |  |  |  |  |  |  |  |  |  |  |  |  |  |  |  |  |  |  |  |  |  |  |  |  |  |
| Equilibrium | 9 | 3331 | -3.78 (-8.08, 0.51) | 0.08 |  | 50.13 | <0.001 | 84.00 | <0.001 |  | 0.64 | 88.09 | 0.83 |  |  |  |  |  |  |  |  |  |  |  |  |  |  |  |  |  |  |  |  |  |  |  |  |  |  |  |  |
| Disequilibrium | 1 | 174 | 13.55 (-6.13, 33.24) | 0.17 |  | - | - | - |  |  |  |  |  |  |  |  |  |  |  |  |  |  |  |  |  |  |  |  |  |  |  |  |  |  |  |  |  |  |  |  |  |
| NM | 2 | 250 | 2.69 (-38.64, 44.03) | 0.89 |  | 9.32 | 0.002 | 89.30 |  |  |  |  |  |  |  |  |  |  |  |  |  |  |  |  |  |  |  |  |  |  |  |  |  |  |  |  |  |  |  |  |  |
| Quality score | | | | | | | | | | | | | |  |  |  |  |  |  |  |  |  |  |  |  |  |  |  |  |  |  |  |  |  |  |  |  |  |  |  |  |
| High quality | 6 | 2960 | 2.07 (-5.95, 10.10) | 0.61 |  | 42.69 | <0.001 | 88.30 | <0.001 |  | -1.89 | 88.91 | 0.56 |  |  |  |  |  |  |  |  |  |  |  |  |  |  |  |  |  |  |  |  |  |  |  |  |  |  |  |  |
| Medium quality | 5 | 795 | -5.17 (-20.73, 10.38) | 0.51 |  | 31.97 | <0.001 | 87.50 |  |  |  |  |  |  |  |  |  |  |  |  |  |  |  |  |  |  |  |  |  |  |  |  |  |  |  |  |  |  |  |  |  |
| Design of study | | | | | | | | | | | | | |  |  | | |  | | |  | |  | |  | |  | |  | |  | |  | |  | |  | |  | |  |
| Case-control | 7 | 2705 | -5.42 (-14.69, 3.84) | 0.25 |  | 39.38 | <0.001 | 84.80 | 0.16 |  | -0.39 | 89.06 | 0.90 |  |  |  |  |  |  |  |  |  |  |  |  |  |  |  |  |  |  |  |  |  |  |  |  |  |  |  |  |
| Cross-sectional | 4 | 1050 | 3.21 (-10.51, 16.93) | 0.64 |  | 50.60 | <0.001 | 94.10 |  |  |  |  |  |  |  |  |  |  |  |  |  |  |  |  |  |  |  |  |  |  |  |  |  |  |  |  |  |  |  |  |  |
| Adjustment of confounders | | | | | | | | | | | | | |  | | |  | | |  |  |  |  |  |  |  |  |  |  |  |  |  |  |  |  |  |  |  |  |  |  |
| Adjusted | 5 | 2757 | -0.40 (-3.79, 2.98) | 0.81 |  | 5.86 | 0.21 | 31.70 | 0.02 |  | -0.79 | 89.02 | 0.79 |  |  |  |  |  |  |  |  |  |  |  |  |  |  |  |  |  |  |  |  |  |  |  |  |  |  |  |  |
| Unadjusted | 6 | 998 | 0.02 (-15.24, 15.30) | 0.99 |  | 80.78 | <0.001 | 93.80 |  |  |  |  |  |  |  |  |  |  |  |  |  |  |  |  |  |  |  |  |  |  |  |  |  |  |  |  |  |  |  |  |  |
| **Homozygous model (AA vs. GG)** | | | | | | | | | | | | | |  |  |  |  |  |  |  |  |  |  |  |  |  |  |  |  |  |  |  |  |  |  |  |  |  |  |  |  |
| Ethnicity | | | | | | | | | | | | | |  |  |  |  |  |  |  |  |  |  |  |  |  |  |  |  |  |  |  |  |  |  |  |  |  |  |  |  |
| Asian | 4 | 201 | 1.17 (0.76, 1.58) | **<0.001** |  | 1.87 | 0.59 | 0.00 | 0.01 |  | 1.19 | 40.80 | **0.001** |  |  |  |  |  |  |  |  |  |  |  |  |  |  |  |  |  |  |  |  |  |  |  |  |  |  |  |  |
| Caucasian | 2 | 1357 | 1.96 (-7.15, 11.07) | 0.67 |  | 2.02 | 0.15 | 50.60 |  |  |  |  |  |  |  |  |  |  |  |  |  |  |  |  |  |  |  |  |  |  |  |  |  |  |  |  |  |  |  |  |  |
| Mix | 5 | 309 | -2.13 (-14.34, 10.07) | 0.73 |  | 12.83 | 0.01 | 68.80 |  |  |  |  |  |  |  |  |  |  |  |  |  |  |  |  |  |  |  |  |  |  |  |  |  |  |  |  |  |  |  |  |  |
| Participants’ health status | | | | | | | | | | | | | |  |  |  |  |  |  |  |  |  |  |  |  |  |  |  |  |  |  |  |  |  |  |  |  |  |  |  |  |
| Heart disease | 3 | 155 | -2.59 (-20.35, 15.16) | 0.77 |  | 3.32 | 0.19 | 39.80 | 0.03 |  | 0.61 | 54.81 | 0.66 |  |  |  |  |  |  |  |  |  |  |  |  |  |  |  |  |  |  |  |  |  |  |  |  |  |  |  |  |
| Healthy | 3 | 498 | 5.52 (1.60, 9.43) | **0.006** |  | 2.45 | 0.29 | 18.50 |  |  |  |  |  |  |  |  |  |  |  |  |  |  |  |  |  |  |  |  |  |  |  |  |  |  |  |  |  |  |  |  |  |
| Others | 5 | 1214 | -3.08 (-10.68, 4.51) | 0.42 |  | 7.90 | 0.09 | 49.40 |  |  |  |  |  |  |  |  |  |  |  |  |  |  |  |  |  |  |  |  |  |  |  |  |  |  |  |  |  |  |  |  |  |
| Hardy-Weinberg equilibrium | | | | | | | | | | | | | |  |  |  |  |  |  |  |  |  |  |  |  |  |  |  |  |  |  |  |  |  |  |  |  |  |  |  |  |
| Equilibrium | 9 | 1698 | 1.58 (-1.39, 4.56) | 0.29 |  | 13.44 | 0.09 | 40.50 | 0.20 |  | 2.11 | 42.63 | 0.07 |  |  |  |  |  |  |  |  |  |  |  |  |  |  |  |  |  |  |  |  |  |  |  |  |  |  |  |  |
| Disequilibrium | 1 | 51 | 10.48 (-11.57, 32.54) | 0.35 |  | - | - | - |  |  |  |  |  |  |  |  |  |  |  |  |  |  |  |  |  |  |  |  |  |  |  |  |  |  |  |  |  |  |  |  |  |
| NM | 2 | 118 | -1.75 (-34.58, 31.07) | 0.91 |  | 4.08 | 0.04 | 75.50 |  |  |  |  |  |  |  |  |  |  |  |  |  |  |  |  |  |  |  |  |  |  |  |  |  |  |  |  |  |  |  |  |  |
| Quality score | | | | | | | | | | | | | |  |  |  |  |  |  |  |  |  |  |  |  |  |  |  |  |  |  |  |  |  |  |  |  |  |  |  |  |
| High quality | 6 | 1508 | 2.18 (-1.24, 5.61) | 0.35 |  | 9.17 | 0.10 | 45.50 | 0.12 |  | 1.23 | 46.56 | **0.001** |  |  |  |  |  |  |  |  |  |  |  |  |  |  |  |  |  |  |  |  |  |  |  |  |  |  |  |  |
| Medium quality | 5 | 359 | -2.49 (-14.86, 9.87) | 0.69 |  | 9.00 | 0.06 | 55.60 |  |  |  |  |  |  |  |  |  |  |  |  |  |  |  |  |  |  |  |  |  |  |  |  |  |  |  |  |  |  |  |  |  |
| Design of study | | | | | | | | | | | | | |  |  |  |  |  |  |  |  |  |  |  |  |  |  |  |  |  |  |  |  |  |  |  |  |  |  |  |  |
| Case-control | 7 | 1336 | -2.46 (-9.31, 4.38) | 0.48 |  | 10.84 | 0.09 | 44.70 | 0.01 |  | 1.55 | 40.84 | **0.02** |  |  |  |  |  |  |  |  |  |  |  |  |  |  |  |  |  |  |  |  |  |  |  |  |  |  |  |  |
| Cross-sectional | 4 | 531 | 5.13 (1.59, 8.68) | **0.005** |  | 3.30 | 0.34 | 9.20 |  |  |  |  |  |  |  |  |  |  |  |  |  |  |  |  |  |  |  |  |  |  |  |  |  |  |  |  |  |  |  |  |  |
| Adjustment of confounders | | | | | | | | | | | | | |  | | |  | | |  |  |  |  |  |  |  |  |  |  |  |  |  |  |  |  |  |  |  |  |  |  |
| Adjusted | 5 | 1407 | 0.95 (-2.15, 4.06) | 0.54 |  | 4.66 | 0.32 | 56.70 | 0.03 |  | 1.39 | 43.60 | **0.01** |  |  |  |  |  |  |  |  |  |  |  |  |  |  |  |  |  |  |  |  |  |  |  |  |  |  |  |  |
| Unadjusted | 6 | 460 | 2.20 (-6.70, 11.10) | 0.62 |  | 11.55 | 0.04 | 14.10 |  |  |  |  |  |  |  |  |  |  |  |  |  |  |  |  |  |  |  |  |  |  |  |  |  |  |  |  |  |  |  |  |  |
| **Heterozygous model (GA vs. GG)** | | | | | | | | | | | | | |  |  |  |  |  |  |  |  |  |  |  |  |  |  |  |  |  |  |  |  |  |  |  |  |  |  |  |  |
| Ethnicity | | | | | | | | | | | | | |  |  |  |  |  |  |  |  |  |  |  |  |  |  |  |  |  |  |  |  |  |  |  |  |  |  |  |  |
| Asian | 4 | 1581 | -5.15 (-14.60, 4.29) | 0.28 |  | 8.17 | 0.04 | 63.30 | <0.001 |  | -0.48 | 77.78 | 0.75 |  |  |  |  |  |  |  |  |  |  |  |  |  |  |  |  |  |  |  |  |  |  |  |  |  |  |  |  |
| Caucasian | 2 | 922 | 0.77 (-4.93, 6.48) | 0.79 |  | 0.89 | 0.34 | 0.00 |  |  |  |  |  |  |  |  |  |  |  |  |  |  |  |  |  |  |  |  |  |  |  |  |  |  |  |  |  |  |  |  |  |
| Mix | 5 | 459 | -1.65(-18.71, 15.41) | 0.85 |  | 33.49 | <0.001 | 88.10 |  |  |  |  |  |  |  |  |  |  |  |  |  |  |  |  |  |  |  |  |  |  |  |  |  |  |  |  |  |  |  |  |  |
| Participants’ health status | | | | | | | | | | | | | |  |  |  |  |  |  |  |  |  |  |  |  |  |  |  |  |  |  |  |  |  |  |  |  |  |  |  |  |
| Heart disease | 3 | 356 | -8.62 (-17.81, 0.57) | 0.06 |  | 0.64 | 0.72 | 0.00 | <0.001 |  | -0.57 | 84.22 | 0.68 |  |  |  |  |  |  |  |  |  |  |  |  |  |  |  |  |  |  |  |  |  |  |  |  |  |  |  |  |
| Healthy | 3 | 682 | 0.67 (-14.69, 16.03) | 0.93 |  | 32.87 | <0.001 | 93.90 |  |  |  |  |  |  |  |  |  |  |  |  |  |  |  |  |  |  |  |  |  |  |  |  |  |  |  |  |  |  |  |  |  |
| Others | 5 | 1924 | 2.31 (2.14, 2.49) | <0.001 |  | 3.68 | 0.45 | 0.00 |  |  |  |  |  |  |  |  |  |  |  |  |  |  |  |  |  |  |  |  |  |  |  |  |  |  |  |  |  |  |  |  |  |
| Hardy-Weinberg equilibrium | | | | | | | | | | | | | |  |  |  |  |  |  |  |  |  |  |  |  |  |  |  |  |  |  |  |  |  |  |  |  |  |  |  |  |
| Equilibrium | 9 | 2621 | 1.36 (-3.98, 6.71) | 0.61 |  | 42.98 | <0.001 | 81.40 | <0.001 |  | -2.33 | 84.29 | 0.23 |  |  |  |  |  |  |  |  |  |  |  |  |  |  |  |  |  |  |  |  |  |  |  |  |  |  |  |  |
| Disequilibrium | 1 | 158 | -3.94 (-16.98, 9.09) | 0.55 |  | - | - | - |  |  |  |  |  |  |  |  |  |  |  |  |  |  |  |  |  |  |  |  |  |  |  |  |  |  |  |  |  |  |  |  |  |
| NM | 2 | 183 | -13.39 (-21.40, -5.38) | 0.001 |  | 0.49 | 0.48 | 0.00 |  |  |  |  |  |  |  |  |  |  |  |  |  |  |  |  |  |  |  |  |  |  |  |  |  |  |  |  |  |  |  |  |  |
| Quality score | | | | | | | | | | | | | |  |  |  |  |  |  |  |  |  |  |  |  |  |  |  |  |  |  |  |  |  |  |  |  |  |  |  |  |
| High quality | 6 | 2330 | -1.64 (-7.86, 4.57) | 0.60 |  | 16.45 | 0.006 | 69.60 | <0.001 |  | -1.42 | 78.84 | 0.52 |  |  |  |  |  |  |  |  |  |  |  |  |  |  |  |  |  |  |  |  |  |  |  |  |  |  |  |  |
| Medium quality | 5 | 632 | -4.85 (-19.17, 9.46) | 0.54 |  | 21.67 | <0.001 | 81.50 |  |  |  |  |  |  |  |  |  |  |  |  |  |  |  |  |  |  |  |  |  |  |  |  |  |  |  |  |  |  |  |  |  |
| Design of study | | | | | | | | | | | | | |  | |  | | |  | | |  | |  | |  | |  | |  | |  | |  | |  | |  | |  | |
| Case-control | 7 | 2229 | -2.27 (-7.44, 2.90) | 0.38 |  | 9.74 | 0.13 | 38.40 | <0.001 |  | -0.65 | 79.38 | 0.76 |  |  |  |  |  |  |  |  |  |  |  |  |  |  |  |  |  |  |  |  |  |  |  |  |  |  |  |  |
| Cross-sectional | 4 | 733 | 1.11 (-12.92, 15.15) | 0.87 |  | 32.91 | <0.001 | 90.90 |  |  |  |  |  |  |  |  |  |  |  |  |  |  |  |  |  |  |  |  |  |  |  |  |  |  |  |  |  |  |  |  |  |
| Adjustment of confounders | | | | | | | | | | | | | |  | | |  | | |  |  |  |  |  |  |  |  |  |  |  |  |  |  |  |  |  |  |  |  |  |  |
| Adjusted | 5 | 2186 | 2.31 (2.14, 2.49) | **<0.001** |  | 1.27 | 0.86 | 0.00 | 0.002 |  | -1.87 | 80.73 | 0.36 |  |  |  |  |  |  |  |  |  |  |  |  |  |  |  |  |  |  |  |  |  |  |  |  |  |  |  |  |
| Unadjusted | 6 | 776 | -7.02 (-21.02, 6.97) | 0.32 |  | 48.56 | <0.001 | 89.70 |  |  |  |  |  |  |  |  |  |  |  |  |  |  |  |  |  |  |  |  |  |  |  |  |  |  |  |  |  |  |  |  |  |

^a^ All analysis was done using random effects model. WMD, weighted mean difference; 95% CI, 95% confidence interval; NM, not mention.

**Supplementary Table S5-**The association between *CD36* rs1761667 polymorphism and triglyceride indices based on several subgroups. All analyses were conducted using a random-effects model^a^

|  | **No. of data-sets** | **No. of subjects** | **Meta-analysis** | |  | **Heterogeneity** | | | |  | **Meta-regression** | | |  |  |  |  |  |  |  |  |  |  |  |  |  |  |  |  |  |  |  |  |  |  |  |  |  |  |  |  |
| --- | --- | --- | --- | --- | --- | --- | --- | --- | --- | --- | --- | --- | --- | --- | --- | --- | --- | --- | --- | --- | --- | --- | --- | --- | --- | --- | --- | --- | --- | --- | --- | --- | --- | --- | --- | --- | --- | --- | --- | --- | --- |
|  |  |  | **WMD^2^ (95%CI)** | **P _effect_** |  | ***Q* statistic** | **P _within_** | **I^2^ (%)** | **P _between group_** |  | **β** | **I^2^_residual (%)_** | **P-value** |  |  |  |  |  |  |  |  |  |  |  |  |  |  |  |  |  |  |  |  |  |  |  |  |  |  |  |  |
| **Triglyceride (mg/dl)** | | | | | | | | | | | | | |  |  |  |  |  |  |  |  |  |  |  |  |  |  |  |  |  |  |  |  |  |  |  |  |  |  |  |  |
| **Allelic model (A vs. G)** | | | | | | | | | | | | | |  |  |  |  |  |  |  |  |  |  |  |  |  |  |  |  |  |  |  |  |  |  |  |  |  |  |  |  |
| Ethnicity | | | | | | | | | | | | | |  |  |  |  |  |  |  |  |  |  |  |  |  |  |  |  |  |  |  |  |  |  |  |  |  |  |  |  |
| Asian | 4 | 1916 | -5.26 (-13.30, 2.77) | 0.19 |  | 4.98 | 0.17 | 39.70 | 0.72 |  | -3.65 | 49.39 | **0.006** |  |  |  |  |  |  |  |  |  |  |  |  |  |  |  |  |  |  |  |  |  |  |  |  |  |  |  |  |
| Caucasian | 2 | 1300 | -3.31 (-7.86, 1.24) | 0.15 |  | 0.06 | 0.80 | 0.00 |  |  |  |  |  |  |  |  |  |  |  |  |  |  |  |  |  |  |  |  |  |  |  |  |  |  |  |  |  |  |  |  |  |
| Mix | 5 | 994 | -14.28 (-25.00, -3.55) | 0.009 |  | 13.29 | 0.01 | 69.90 |  |  |  |  |  |  |  |  |  |  |  |  |  |  |  |  |  |  |  |  |  |  |  |  |  |  |  |  |  |  |  |  |  |
| Participants’ health status | | | | | | | | | | | | | |  |  |  |  |  |  |  |  |  |  |  |  |  |  |  |  |  |  |  |  |  |  |  |  |  |  |  |  |
| Heart disease | 3 | 796 | -3.68 (-12.94, 5.56) | 0.43 |  | 2.19 | 0.33 | 8.80 | 0.04 |  | -3.35 | 34.91 | **0.003** |  |  |  |  |  |  |  |  |  |  |  |  |  |  |  |  |  |  |  |  |  |  |  |  |  |  |  |  |
| Healthy | 3 | 1764 | -4.26 (-6.12, -2.40) | **<0.001** |  | 0.27 | 0.87 | 0.00 |  |  |  |  |  |  |  |  |  |  |  |  |  |  |  |  |  |  |  |  |  |  |  |  |  |  |  |  |  |  |  |  |  |
| Others | 5 | 1650 | -15.96 (-26.53, -5.39) | 0.003 |  | 10.33 | 0.03 | 61.30 |  |  |  |  |  |  |  |  |  |  |  |  |  |  |  |  |  |  |  |  |  |  |  |  |  |  |  |  |  |  |  |  |  |
| Hardy-Weinberg equilibrium | | | | | | | | | | | | | |  |  |  |  |  |  |  |  |  |  |  |  |  |  |  |  |  |  |  |  |  |  |  |  |  |  |  |  |
| Equilibrium | 7 | 400 | -6.65(-11.20, -2.11) | **0.004** |  | 16.97 | 0.01 | 58.80 | 0.49 |  | -4.56 | 41.03 | **0.001** |  |  |  |  |  |  |  |  |  |  |  |  |  |  |  |  |  |  |  |  |  |  |  |  |  |  |  |  |
| Disequilibrium | 1 | 348 | -4.76 (-16.75, 7.22) | 0.43 |  | - | - | - |  |  |  |  |  |  |  |  |  |  |  |  |  |  |  |  |  |  |  |  |  |  |  |  |  |  |  |  |  |  |  |  |  |
| NM | 3 | 3462 | -12.53 (-25.06, 0.002) | 0.05 |  | 1.05 | 0.59 | 0.00 |  |  |  |  |  |  |  |  |  |  |  |  |  |  |  |  |  |  |  |  |  |  |  |  |  |  |  |  |  |  |  |  |  |
| Quality score | | | | | | | | | | | | | |  |  |  |  |  |  |  |  |  |  |  |  |  |  |  |  |  |  |  |  |  |  |  |  |  |  |  |  |
| High quality | 5 | 2404 | -5.20 (-9.15, -1.25) | **0.01** |  | 4.22 | 0.37 | 5.10 | 0.99 |  | -4.57 | 51.54 | **0.01** |  |  |  |  |  |  |  |  |  |  |  |  |  |  |  |  |  |  |  |  |  |  |  |  |  |  |  |  |
| Medium quality | 6 | 1806 | -8.79 (-16.85, -0.73) | **0.03** |  | 14.75 | 0.01 | 66.10 |  |  |  |  |  |  |  |  |  |  |  |  |  |  |  |  |  |  |  |  |  |  |  |  |  |  |  |  |  |  |  |  |  |
| Design of study | | | | | | | | | | | | | |  |  |  |  |  |  |  |  |  |  |  |  |  |  |  |  |  |  |  |  |  |  |  |  |  |  |  |  |
| Case-control | 5 | 1220 | -13.68 (-27.02, -0.33) | **0.04** |  | 12.22 | 0.01 | 67.30 | 0.04 |  | -4.20 | 55.34 | **0.03** |  |  |  |  |  |  |  |  |  |  |  |  |  |  |  |  |  |  |  |  |  |  |  |  |  |  |  |  |
| Cross-sectional | 6 | 2990 | -4.52 (-6.29, -2.75) | **<0.001** |  | 2.82 | 0.72 | 0.00 |  |  |  |  |  |  |  |  |  |  |  |  |  |  |  |  |  |  |  |  |  |  |  |  |  |  |  |  |  |  |  |  |  |
| Adjustment of confounders | | | | | | | | | | | | | |  |  |  |  |  |  |  |  |  |  |  |  |  |  |  |  |  |  |  |  |  |  |  |  |  |  |  |  |
| Adjusted | 3 | 1324 | -8.02 (-18.37, 2.33) | 0.12 |  | 3.10 | 0.21 | 35.50 | 0.76 |  | -4.09 | 48.66 | **0.01** |  |  |  |  |  |  |  |  |  |  |  |  |  |  |  |  |  |  |  |  |  |  |  |  |  |  |  |  |
| Unadjusted | 8 | 2886 | -7.93 (-13.35, -2.50) | **0.004** |  | 15.78 | 0.02 | 55.60 |  |  |  |  |  |  |  |  |  |  |  |  |  |  |  |  |  |  |  |  |  |  |  |  |  |  |  |  |  |  |  |  |  |
| **Dominant model (AA+GA vs. GG)** | | | | | | | | | | | | | |  |  |  |  |  |  |  |  |  |  |  |  |  |  |  |  |  |  |  |  |  |  |  |  |  |  |  |  |
| Ethnicity | | | | | | | | | | | | | |  |  |  |  |  |  |  |  |  |  |  |  |  |  |  |  |  |  |  |  |  |  |  |  |  |  |  |  |
| Asian | 4 | 958 | -9.79 (-22.17, 2.58) | 0.12 |  | 5.54 | 0.13 | 45.80 | 0.03 |  | -2.94 | 54.72 | 0.19 |  |  |  |  |  |  |  |  |  |  |  |  |  |  |  |  |  |  |  |  |  |  |  |  |  |  |  |  |
| Caucasian | 2 | 650 | -5.67 (-14.20, 2.84) | 0.19 |  | 0.00 | 0.94 | 0.00 |  |  |  |  |  |  |  |  |  |  |  |  |  |  |  |  |  |  |  |  |  |  |  |  |  |  |  |  |  |  |  |  |  |
| Mix | 5 | 497 | -15.98 (-39.09, 7.12) | 0.17 |  | 9.94 | 0.04 | 59.80 |  |  |  |  |  |  |  |  |  |  |  |  |  |  |  |  |  |  |  |  |  |  |  |  |  |  |  |  |  |  |  |  |  |
| Participants’ health status | | | | | | | | | | | | | |  |  |  |  |  |  |  |  |  |  |  |  |  |  |  |  |  |  |  |  |  |  |  |  |  |  |  |  |
| Heart disease | 3 | 398 | -9.35 (-22.37, 3.67) | 0.15 |  | 0.92 | 0.63 | 0.00 | 0.02 |  | -3.09 | 53.95 | 0.10 |  |  |  |  |  |  |  |  |  |  |  |  |  |  |  |  |  |  |  |  |  |  |  |  |  |  |  |  |
| Healthy | 3 | 882 | -0.27 (-6.43, 5.89) | 0.93 |  | 3.06 | 0.21 | 34.70 |  |  |  |  |  |  |  |  |  |  |  |  |  |  |  |  |  |  |  |  |  |  |  |  |  |  |  |  |  |  |  |  |  |
| Others | 5 | 825 | -23.65 (-47.06, -0.23) | 0.04 |  | 10.80 | 0.02 | 62.90 |  |  |  |  |  |  |  |  |  |  |  |  |  |  |  |  |  |  |  |  |  |  |  |  |  |  |  |  |  |  |  |  |  |
| Hardy-Weinberg equilibrium | | | | | | | | | | | | | |  |  |  |  |  |  |  |  |  |  |  |  |  |  |  |  |  |  |  |  |  |  |  |  |  |  |  |  |
| Equilibrium | 7 | 1731 | -6.64 (-14.76, 1.46) | 0.10 |  | 19.43 | 0.007 | 64.00 | 0.35 |  | -4.61 | 49.51 | 0.10 |  |  |  |  |  |  |  |  |  |  |  |  |  |  |  |  |  |  |  |  |  |  |  |  |  |  |  |  |
| Disequilibrium | 1 | 174 | -13.83 (-37.33, 9.65) | 0.24 |  | - | - | - |  |  |  |  |  |  |  |  |  |  |  |  |  |  |  |  |  |  |  |  |  |  |  |  |  |  |  |  |  |  |  |  |  |
| NM | 3 | 200 | -11.73 (-35.91, 12.44) | 0.34 |  | 0.69 | 0.70 | 0.00 |  |  |  |  |  |  |  |  |  |  |  |  |  |  |  |  |  |  |  |  |  |  |  |  |  |  |  |  |  |  |  |  |  |
| Quality score | | | | | | | | | | | | | |  |  |  |  |  |  |  |  |  |  |  |  |  |  |  |  |  |  |  |  |  |  |  |  |  |  |  |  |
| High quality | 5 | 1202 | -5.74 (-11.60, 0.12) | 0.055 |  | 1.08 | 0.89 | 0.00 | 0.05 |  | -4.26 | 54.89 | 0.13 |  |  |  |  |  |  |  |  |  |  |  |  |  |  |  |  |  |  |  |  |  |  |  |  |  |  |  |  |
| Medium quality | 6 | 903 | -13.50 (-29.28, 2.28) | 0.09 |  | 17.06 | 0.004 | 70.70 |  |  |  |  |  |  |  |  |  |  |  |  |  |  |  |  |  |  |  |  |  |  |  |  |  |  |  |  |  |  |  |  |  |
| Design of study | | | | | | | | | | | | | |  |  |  |  |  |  |  |  |  |  |  |  |  |  |  |  |  |  |  |  |  |  |  |  |  |  |  |  |
| Case-control | 5 | 610 | -25.95 (-48.25, -3.66) | **0.02** |  | 9.21 | 0.056 | 56.50 | 0.007 |  | -2.35 | 54.56 | 0.29 |  |  |  |  |  |  |  |  |  |  |  |  |  |  |  |  |  |  |  |  |  |  |  |  |  |  |  |  |
| Cross-sectional | 6 | 1495 | -0.61 (-4.51, 3.28) | 0.75 |  | 5.58 | 0.34 | 10.40 |  |  |  |  |  |  |  |  |  |  |  |  |  |  |  |  |  |  |  |  |  |  |  |  |  |  |  |  |  |  |  |  |  |
| Adjustment of confounders | | | | | | | | | | | | | |  | | |  | | |  |  |  |  |  |  |  |  |  |  |  |  |  |  |  |  |  |  |  |  |  |  |
| Adjusted | 3 | 662 | -6.53 (-15.00, 1.92) | 0.13 |  | 0.99 | 0.61 | 0.00 | 0.14 |  | -3.47 | 54.81 | 0.15 |  |  |  |  |  |  |  |  |  |  |  |  |  |  |  |  |  |  |  |  |  |  |  |  |  |  |  |  |
| Unadjusted | 8 | 1443 | -8.20 (-17.91, 1.51) | 0.09 |  | 18.89 | 0.009 | 62.90 |  |  |  |  |  |  |  |  |  |  |  |  |  |  |  |  |  |  |  |  |  |  |  |  |  |  |  |  |  |  |  |  |  |
| **Recessive model (AA vs. GA + GG)** | | | | | | | | | | | | | |  |  |  |  |  |  |  |  |  |  |  |  |  |  |  |  |  |  |  |  |  |  |  |  |  |  |  |  |
| Ethnicity | | | | | | | | | | | | | |  |  |  |  |  |  |  |  |  |  |  |  |  |  |  |  |  |  |  |  |  |  |  |  |  |  |  |  |
| Asian | 4 | 958 | -3.01 (-19.63, 13.59) | 0.72 |  | 5.15 | 0.16 | 41.70 | 0.02 |  | -7.53 | 63.41 | **0.003** |  |  |  |  |  |  |  |  |  |  |  |  |  |  |  |  |  |  |  |  |  |  |  |  |  |  |  |  |
| Caucasian | 2 | 650 | -3.40 (-10.22, 3.42) | 0.32 |  | 0.09 | 0.76 | 0.00 |  |  |  |  |  |  |  |  |  |  |  |  |  |  |  |  |  |  |  |  |  |  |  |  |  |  |  |  |  |  |  |  |  |
| Mix | 5 | 497 | -28.84(-46.76, -10.92) | 0.002 |  | 20.01 | <0.001 | 80.00 |  |  |  |  |  |  |  |  |  |  |  |  |  |  |  |  |  |  |  |  |  |  |  |  |  |  |  |  |  |  |  |  |  |
| Participants’ health status | | | | | | | | | | | | | |  |  |  |  |  |  |  |  |  |  |  |  |  |  |  |  |  |  |  |  |  |  |  |  |  |  |  |  |
| Heart disease | 3 | 398 | -1.66 (-36.71, 33.39) | 0.92 |  | 6.10 | 0.04 | 67.20 | 0.002 |  | -7.27 | 59.34 | **0.003** |  |  |  |  |  |  |  |  |  |  |  |  |  |  |  |  |  |  |  |  |  |  |  |  |  |  |  |  |
| Healthy | 3 | 882 | -8.11 (-14.74, -1.48) | 0.01 |  | 4.64 | 0.09 | 56.90 |  |  |  |  |  |  |  |  |  |  |  |  |  |  |  |  |  |  |  |  |  |  |  |  |  |  |  |  |  |  |  |  |  |
| Others | 5 | 825 | -27.75 (-43.09, -12.41) | <0.001 |  | 9.59 | 0.04 | 58.30 |  |  |  |  |  |  |  |  |  |  |  |  |  |  |  |  |  |  |  |  |  |  |  |  |  |  |  |  |  |  |  |  |  |
| Hardy-Weinberg equilibrium | | | | | | | | | | | | | |  |  |  |  |  |  |  |  |  |  |  |  |  |  |  |  |  |  |  |  |  |  |  |  |  |  |  |  |
| Equilibrium | 7 | 1731 | -12.59 (-22.08, -3.10) | **0.009** |  | 29.40 | <0.001 | 76.20 | 0.28 |  | -8.45 | 68.24 | **0.02** |  |  |  |  |  |  |  |  |  |  |  |  |  |  |  |  |  |  |  |  |  |  |  |  |  |  |  |  |
| Disequilibrium | 1 | 174 | -1.55 (-26.90, 23.78) | 0.90 |  | - | - | - |  |  |  |  |  |  |  |  |  |  |  |  |  |  |  |  |  |  |  |  |  |  |  |  |  |  |  |  |  |  |  |  |  |
| NM | 3 | 200 | -24.01 (-42.47, -5.56) | 0.01 |  | 2.11 | 0.34 | 5.30 |  |  |  |  |  |  |  |  |  |  |  |  |  |  |  |  |  |  |  |  |  |  |  |  |  |  |  |  |  |  |  |  |  |
| Quality score | | | | | | | | | | | | | |  |  |  |  |  |  |  |  |  |  |  |  |  |  |  |  |  |  |  |  |  |  |  |  |  |  |  |  |
| High quality | 5 | 1202 | -16.20 (-28.61, -3.78) | **0.01** |  | 10.07 | 0.03 | 60.30 | 0.30 |  | -8.51 | 68.75 | 0.051 |  |  |  |  |  |  |  |  |  |  |  |  |  |  |  |  |  |  |  |  |  |  |  |  |  |  |  |  |
| Medium quality | 6 | 903 | -12.03 (-28.58, 4.52) | 0.15 |  | 21.19 | 0.001 | 76.40 |  |  |  |  |  |  |  |  |  |  |  |  |  |  |  |  |  |  |  |  |  |  |  |  |  |  |  |  |  |  |  |  |  |
| Design of study | | | | | | | | | | | | | |  |  | | |  | | |  | |  | |  | |  | |  | |  | |  | |  | |  | |  | |  |
| Case-control | 5 | 610 | -15.90 (-45.47, 13.66) | 0.29 |  | 19.80 | 0.001 | 79.80 | 0.056 |  | -8.27 | 72.94 | **0.04** |  |  |  |  |  |  |  |  |  |  |  |  |  |  |  |  |  |  |  |  |  |  |  |  |  |  |  |  |
| Cross-sectional | 6 | 1495 | -14.54 (-22.74, -6.35) | **<0.001** |  | 8.86 | 0.11 | 43.60 |  |  |  |  |  |  |  |  |  |  |  |  |  |  |  |  |  |  |  |  |  |  |  |  |  |  |  |  |  |  |  |  |  |
| Adjustment of confounders | | | | | | | | | | | | | |  | | |  | | |  |  |  |  |  |  |  |  |  |  |  |  |  |  |  |  |  |  |  |  |  |  |
| Adjusted | 3 | 662 | -18.72 (-41.89, 4.44) | 0.11 |  | 6.75 | 0.03 | 70.40 | 0.11 |  | -8.10 | 66.45 | **0.03** |  |  |  |  |  |  |  |  |  |  |  |  |  |  |  |  |  |  |  |  |  |  |  |  |  |  |  |  |
| Unadjusted | 8 | 1443 | -14.57 (-25.80, -3.33) | **0.01** |  | 23.05 | 0.002 | 69.60 |  |  |  |  |  |  |  |  |  |  |  |  |  |  |  |  |  |  |  |  |  |  |  |  |  |  |  |  |  |  |  |  |  |
| **Homozygous model (AA vs. GG)** | | | | | | | | | | | | | |  |  |  |  |  |  |  |  |  |  |  |  |  |  |  |  |  |  |  |  |  |  |  |  |  |  |  |  |
| Ethnicity | | | | | | | | | | | | | |  |  |  |  |  |  |  |  |  |  |  |  |  |  |  |  |  |  |  |  |  |  |  |  |  |  |  |  |
| Asian | 4 | 506 | -8.87 (-27.54, 9.80) | 0.35 |  | 5.23 | 0.15 | 42.70 | 0.76 |  | -8.64 | 61.85 | **0.02** |  |  |  |  |  |  |  |  |  |  |  |  |  |  |  |  |  |  |  |  |  |  |  |  |  |  |  |  |
| Caucasian | 2 | 326 | -6.92 (-16.53, 2.68) | 0.15 |  | 0.04 | 0.84 | 0.00 |  |  |  |  |  |  |  |  |  |  |  |  |  |  |  |  |  |  |  |  |  |  |  |  |  |  |  |  |  |  |  |  |  |
| Mix | 5 | 229 | -38.05 (-71.02, -5.07) | 0.02 |  | 18.31 | 0.001 | 78.20 |  |  |  |  |  |  |  |  |  |  |  |  |  |  |  |  |  |  |  |  |  |  |  |  |  |  |  |  |  |  |  |  |  |
| Participants’ health status | | | | | | | | | | | | | |  |  |  |  |  |  |  |  |  |  |  |  |  |  |  |  |  |  |  |  |  |  |  |  |  |  |  |  |
| Heart disease | 3 | 454 | -6.48 (-45.97, 33.00) | 0.74 |  | 4.70 | 0.09 | 57.40 | 0.02 |  | -5.74 | 52.89 | **0.02** |  |  |  |  |  |  |  |  |  |  |  |  |  |  |  |  |  |  |  |  |  |  |  |  |  |  |  |  |
| Healthy | 3 | 155 | -5.46 (-8.92, -2.00) | **0.002** |  | 0.14 | 0.93 | 0.00 |  |  |  |  |  |  |  |  |  |  |  |  |  |  |  |  |  |  |  |  |  |  |  |  |  |  |  |  |  |  |  |  |  |
| Others | 5 | 452 | -38.74 (-66.81, -10.67) | 0.007 |  | 11.77 | 0.01 | 66.00 |  |  |  |  |  |  |  |  |  |  |  |  |  |  |  |  |  |  |  |  |  |  |  |  |  |  |  |  |  |  |  |  |  |
| Hardy-Weinberg equilibrium | | | | | | | | | | | | | |  |  |  |  |  |  |  |  |  |  |  |  |  |  |  |  |  |  |  |  |  |  |  |  |  |  |  |  |
| Equilibrium | 7 | 915 | -11.85 (-23.02, -0.68) | **0.03** |  | 21.58 | 0.003 | 67.60 | 0.38 |  | -8.73 | 59.88 | **0.04** |  |  |  |  |  |  |  |  |  |  |  |  |  |  |  |  |  |  |  |  |  |  |  |  |  |  |  |  |
| Disequilibrium | 1 | 51 | -12.46 (-44.56, 19.62) | 0.44 |  | - | - | - |  |  |  |  |  |  |  |  |  |  |  |  |  |  |  |  |  |  |  |  |  |  |  |  |  |  |  |  |  |  |  |  |  |
| NM | 3 | 95 | -24.91 (-51.83, 2.00) | 0.07 |  | 1.79 | 0.40 | 0.00 |  |  |  |  |  |  |  |  |  |  |  |  |  |  |  |  |  |  |  |  |  |  |  |  |  |  |  |  |  |  |  |  |  |
| Quality score | | | | | | | | | | | | | |  |  |  |  |  |  |  |  |  |  |  |  |  |  |  |  |  |  |  |  |  |  |  |  |  |  |  |  |
| High quality | 5 | 645 | -12.56 (-21.92, -3.19) | **0.009** |  | 4.73 | 0.31 | 15.40 | 0.19 |  | -10.12 | 62.93 | 0.08 |  |  |  |  |  |  |  |  |  |  |  |  |  |  |  |  |  |  |  |  |  |  |  |  |  |  |  |  |
| Medium quality | 6 | 416 | -17.30 (-39.56, 4.95) | 0.12 |  | 17.73 | 0.003 | 71.80 |  |  |  |  |  |  |  |  |  |  |  |  |  |  |  |  |  |  |  |  |  |  |  |  |  |  |  |  |  |  |  |  |  |
| Design of study | | | | | | | | | | | | | |  |  |  |  |  |  |  |  |  |  |  |  |  |  |  |  |  |  |  |  |  |  |  |  |  |  |  |  |
| Case-control | 5 | 248 | -34.06 (-77.31, 9.18) | 0.12 |  | 17.62 | 0.001 | 77.30 | 0.12 |  | -8.65 | 60.69 | 0.11 |  |  |  |  |  |  |  |  |  |  |  |  |  |  |  |  |  |  |  |  |  |  |  |  |  |  |  |  |
| Cross-sectional | 6 | 813 | -6.32 (-9.64, -3.00) | **<0.001** |  | 4.09 | 0.53 | 0.00 |  |  |  |  |  |  |  |  |  |  |  |  |  |  |  |  |  |  |  |  |  |  |  |  |  |  |  |  |  |  |  |  |  |
| Adjustment of confounders | | | | | | | | | | | | | |  | | |  | | |  |  |  |  |  |  |  |  |  |  |  |  |  |  |  |  |  |  |  |  |  |  |
| Adjusted | 3 | 319 | -13.94 (-23.82, -4.06) | 0.12 |  | 3.93 | 0.14 | 49.10 | 0.56 |  | -8.79 | 60.60 | 0.07 |  |  |  |  |  |  |  |  |  |  |  |  |  |  |  |  |  |  |  |  |  |  |  |  |  |  |  |  |
| Unadjusted | 8 | 742 | -14.91 (-28.78, -1.03) | **0.03** |  | 19.86 | 0.006 | 64.70 |  |  |  |  |  |  |  |  |  |  |  |  |  |  |  |  |  |  |  |  |  |  |  |  |  |  |  |  |  |  |  |  |  |
| **Heterozygous model (GA vs. GG)** | | | | | | | | | | | | | |  |  |  |  |  |  |  |  |  |  |  |  |  |  |  |  |  |  |  |  |  |  |  |  |  |  |  |  |
| Ethnicity | | | | | | | | | | | | | |  |  |  |  |  |  |  |  |  |  |  |  |  |  |  |  |  |  |  |  |  |  |  |  |  |  |  |  |
| Asian | 4 | 865 | -10.20 (-23.03, 2.63) | 0.11 |  | 5.50 | 0.13 | 45.50 | <0.001 |  | -1.53 | 58.68 | 0.50 |  |  |  |  |  |  |  |  |  |  |  |  |  |  |  |  |  |  |  |  |  |  |  |  |  |  |  |  |
| Caucasian | 2 | 466 | -4.96 (-13.90, 3.98) | 0.27 |  | 0.00 | 0.97 | 0.00 |  |  |  |  |  |  |  |  |  |  |  |  |  |  |  |  |  |  |  |  |  |  |  |  |  |  |  |  |  |  |  |  |  |
| Mix | 5 | 347 | -6.44 (-28.01, 15.11) | 0.55 |  | 5.50 | 0.08 | 51.80 |  |  |  |  |  |  |  |  |  |  |  |  |  |  |  |  |  |  |  |  |  |  |  |  |  |  |  |  |  |  |  |  |  |
| Participants’ health status | | | | | | | | | | | | | |  |  |  |  |  |  |  |  |  |  |  |  |  |  |  |  |  |  |  |  |  |  |  |  |  |  |  |  |
| Heart disease | 3 | 356 | -11.20 ( -24.58, 2.17) | 0.10 |  | 0.34 | 0.84 | 0.00 | 0.001 |  | -2.49 | 68.62 | 0.24 |  |  |  |  |  |  |  |  |  |  |  |  |  |  |  |  |  |  |  |  |  |  |  |  |  |  |  |  |
| Healthy | 3 | 616 | 1.98 (-9.85, 13.82) | 0.74 |  | 7.73 | 0.02 | 47.10 |  |  |  |  |  |  |  |  |  |  |  |  |  |  |  |  |  |  |  |  |  |  |  |  |  |  |  |  |  |  |  |  |  |
| Others | 5 | 706 | -17.00 (-39.25, 5.23) | 0.13 |  | 9.05 | 0.06 | 55.80 |  |  |  |  |  |  |  |  |  |  |  |  |  |  |  |  |  |  |  |  |  |  |  |  |  |  |  |  |  |  |  |  |  |
| Hardy-Weinberg equilibrium | | | | | | | | | | | | | |  |  |  |  |  |  |  |  |  |  |  |  |  |  |  |  |  |  |  |  |  |  |  |  |  |  |  |  |
| Equilibrium | 7 | 1371 | -6.39 (-16.61, 3.81) | 0.22 |  | 28.22 | <0.001 | 75.20 | 0.23 |  | -3.93 | 67.43 | 0.21 |  |  |  |  |  |  |  |  |  |  |  |  |  |  |  |  |  |  |  |  |  |  |  |  |  |  |  |  |
| Disequilibrium | 1 | 158 | -14.01 (-37.80, 9.77) | 0.24 |  | - | - | - |  |  |  |  |  |  |  |  |  |  |  |  |  |  |  |  |  |  |  |  |  |  |  |  |  |  |  |  |  |  |  |  |  |
| NM | 3 | 149 | -6.32 (-31.73, 19.09) | 0.62 |  | 0.64 | 0.72 | 0.00 |  |  |  |  |  |  |  |  |  |  |  |  |  |  |  |  |  |  |  |  |  |  |  |  |  |  |  |  |  |  |  |  |  |
| Quality score | | | | | | | | | | | | | |  |  |  |  |  |  |  |  |  |  |  |  |  |  |  |  |  |  |  |  |  |  |  |  |  |  |  |  |
| High quality | 5 | 968 | -4.12 (-10.35, 2.09) | 0.19 |  | 0.76 | 0.94 | 0.00 | 0.003 |  | -3.71 | 62.97 | 0.22 |  |  |  |  |  |  |  |  |  |  |  |  |  |  |  |  |  |  |  |  |  |  |  |  |  |  |  |  |
| Medium quality | 6 | 710 | -12.92 (-30.96, 5.12) | 0.16 |  | 21.15 | 0.001 | 76.40 |  |  |  |  |  |  |  |  |  |  |  |  |  |  |  |  |  |  |  |  |  |  |  |  |  |  |  |  |  |  |  |  |  |
| Design of study | | | | | | | | | | | | | |  | |  | | |  | | |  | |  | |  | |  | |  | |  | |  | |  | |  | |  | |
| Case-control | 5 | 521 | -22.35 (-40.44, -4.25) | **0.01** |  | 6.13 | 0.19 | 34.70 | <0.001 |  | -1.50 | 64.72 | 0.57 |  |  |  |  |  |  |  |  |  |  |  |  |  |  |  |  |  |  |  |  |  |  |  |  |  |  |  |  |
| Cross-sectional | 6 | 1157 | 0.80 (-7.24, 8.84) | 0.84 |  | 12.17 | 0.03 | 58.90 |  |  |  |  |  |  |  |  |  |  |  |  |  |  |  |  |  |  |  |  |  |  |  |  |  |  |  |  |  |  |  |  |  |
| Adjustment of confounders | | | | | | | | | | | | | |  | | |  | | |  |  |  |  |  |  |  |  |  |  |  |  |  |  |  |  |  |  |  |  |  |  |
| Adjusted | 3 | 478 | -5.25 (-14.13, 3.62) | 0.24 |  | 0.59 | 0.74 | 0.00 | 0.02 |  | -3.00 | 65.29 | 0.26 |  |  |  |  |  |  |  |  |  |  |  |  |  |  |  |  |  |  |  |  |  |  |  |  |  |  |  |  |
| Unadjusted | 8 | 1200 | 7.31 (-19.03, 4.40) | 0.22 |  | 25.27 | 0.001 | 72.30 |  |  |  |  |  |  |  |  |  |  |  |  |  |  |  |  |  |  |  |  |  |  |  |  |  |  |  |  |  |  |  |  |  |

^a^ All analysis was done using random effects model. WMD, weighted mean difference; 95% CI, 95% confidence interval; NM, not mention.

**Supplementary Table S6-**The association between *CD36* rs1761667 polymorphism and HDL cholesterol indices based on several subgroups. All analyses were conducted using a random-effects model^a^

|  | **No. of data-sets** | **No. of subjects** | **Meta-analysis** | |  | **Heterogeneity** | | | |  | **Meta-regression** | | |  |  |  |  |  |  |  |  |  |  |  |  |  |  |  |  |  |  |  |  |  |  |  |  |  |  |  |  |  |  |  |  |  |  |  |  |  |  |  |  |  |
| --- | --- | --- | --- | --- | --- | --- | --- | --- | --- | --- | --- | --- | --- | --- | --- | --- | --- | --- | --- | --- | --- | --- | --- | --- | --- | --- | --- | --- | --- | --- | --- | --- | --- | --- | --- | --- | --- | --- | --- | --- | --- | --- | --- | --- | --- | --- | --- | --- | --- | --- | --- | --- | --- | --- |
|  |  |  | **WMD^2^ (95%CI)** | **P _effect_** |  | ***Q* statistic** | **P _within_** | **I^2^ (%)** | **P _between group_** |  | **β** | **I^2^_residual (%)_** | **P-value** |  |  |  |  |  |  |  |  |  |  |  |  |  |  |  |  |  |  |  |  |  |  |  |  |  |  |  |  |  |  |  |  |  |  |  |  |  |  |  |  |  |
| **HDL cholesterol (mg/dl)** | | | | | | | | | | | | | |  |  |  |  |  |  |  |  |  |  |  |  |  |  |  |  |  |  |  |  |  |  |  |  |  |  |  |  |  |  |  |  |  |  |  |  |  |  |  |  |  |
| **Allelic model (A vs. G)** | | | | | | | | | | | | | |  |  |  |  |  |  |  |  |  |  |  |  |  |  |  |  |  |  |  |  |  |  |  |  |  |  |  |  |  |  |  |  |  |  |  |  |  |  |  |  |  |
| Ethnicity | | | | | | | | | | | | | |  |  |  |  |  |  |  |  |  |  |  |  |  |  |  |  |  |  |  |  |  |  |  |  |  |  |  |  |  |  |  |  |  |  |  |  |  |  |  |  |  |
| Asian | 5 | 4634 | -0.09 (-0.18, -0.01) | **0.02** |  | 3.00 | 0.55 | 0.00 | 0.13 |  | 0.29 | 72.27 | 0.24 |  |  |  |  |  |  |  |  |  |  |  |  |  |  |  |  |  |  |  |  |  |  |  |  |  |  |  |  |  |  |  |  |  |  |  |  |  |  |  |  |  |
| Caucasian | 2 | 1300 | 0.68 (-2.23, 3.60) | 0.64 |  | 3.47 | 0.06 | 71.20 |  |  |  |  |  |  |  |  |  |  |  |  |  |  |  |  |  |  |  |  |  |  |  |  |  |  |  |  |  |  |  |  |  |  |  |  |  |  |  |  |  |  |  |  |  |  |
| Mix | 5 | 994 | 0.87 (-1.90, 3.65) | 0.53 |  | 21.16 | <0.001 | 86.30 |  |  |  |  |  |  |  |  |  |  |  |  |  |  |  |  |  |  |  |  |  |  |  |  |  |  |  |  |  |  |  |  |  |  |  |  |  |  |  |  |  |  |  |  |  |  |
| Participants’ health status | | | | | | | | | | | | | |  |  |  |  |  |  |  |  |  |  |  |  |  |  |  |  |  |  |  |  |  |  |  |  |  |  |  |  |  |  |  |  |  |  |  |  |  |  |  |  |  |
| Heart disease | 3 | 796 | 0.67 (-0.97, 2.32) | 0.42 |  | 0.25 | 0.88 | 0.00 | 0.11 |  | 0.29 | 72.41 | 0.19 |  |  |  |  |  |  |  |  |  |  |  |  |  |  |  |  |  |  |  |  |  |  |  |  |  |  |  |  |  |  |  |  |  |  |  |  |  |  |  |  |  |
| Healthy | 3 | 1764 | -0.10 (-2.43, 2.23) | 0.93 |  | 13.47 | 0.001 | 85.20 |  |  |  |  |  |  |  |  |  |  |  |  |  |  |  |  |  |  |  |  |  |  |  |  |  |  |  |  |  |  |  |  |  |  |  |  |  |  |  |  |  |  |  |  |  |  |
| Others | 6 | 4368 | 1.22 (-0.44, 2.87) | 0.15 |  | 21.53 | 0.001 | 76.80 |  |  |  |  |  |  |  |  |  |  |  |  |  |  |  |  |  |  |  |  |  |  |  |  |  |  |  |  |  |  |  |  |  |  |  |  |  |  |  |  |  |  |  |  |  |  |
| Hardy-Weinberg equilibrium | | | | | | | | | | | | | |  |  |  |  |  |  |  |  |  |  |  |  |  |  |  |  |  |  |  |  |  |  |  |  |  |  |  |  |  |  |  |  |  |  |  |  |  |  |  |  |  |
| Equilibrium | 9 | 6180 | 0.84 (-0.17, 1.86) | 0.10 |  | 38.72 | <0.001 | 79.30 | 0.60 |  | 0.25 | 70.31 | 0.47 |  |  |  |  |  |  |  |  |  |  |  |  |  |  |  |  |  |  |  |  |  |  |  |  |  |  |  |  |  |  |  |  |  |  |  |  |  |  |  |  |  |
| Disequilibrium | 1 | 348 | 0.78 (-1.37, 2.94) | 0.47 |  | - | - | - |  |  |  |  |  |  |  |  |  |  |  |  |  |  |  |  |  |  |  |  |  |  |  |  |  |  |  |  |  |  |  |  |  |  |  |  |  |  |  |  |  |  |  |  |  |  |
| NM | 3 | 400 | -0.79 (-3.02, 1.43) | 0.48 |  | 0.71 | 0.70 | 0.00 |  |  |  |  |  |  |  |  |  |  |  |  |  |  |  |  |  |  |  |  |  |  |  |  |  |  |  |  |  |  |  |  |  |  |  |  |  |  |  |  |  |  |  |  |  |  |
| Quality score | | | | | | | | | | | | | |  |  |  |  |  |  |  |  |  |  |  |  |  |  |  |  |  |  |  |  |  |  |  |  |  |  |  |  |  |  |  |  |  |  |  |  |  |  |  |  |  |
| High quality | 6 | 5122 | 0.66 (-0.50, 1.84) | 0.26 |  | 10.04 | 0.07 | 50.20 | 0.60 |  | 0.29 | 72.12 | 0.34 |  |  |  |  |  |  |  |  |  |  |  |  |  |  |  |  |  |  |  |  |  |  |  |  |  |  |  |  |  |  |  |  |  |  |  |  |  |  |  |  |  |
| Medium quality | 6 | 1806 | 0.57 (-1.35, 2.50) | 0.56 |  | 29.34 | <0.001 | 83.00 |  |  |  |  |  |  |  |  |  |  |  |  |  |  |  |  |  |  |  |  |  |  |  |  |  |  |  |  |  |  |  |  |  |  |  |  |  |  |  |  |  |  |  |  |  |  |
| Design of study | | | | | | | | | | | | | |  | | |  | | | |  | | | |  | | |  | | |  | | |  | | |  | | |  | | |  | | |  | | |  | | |  | | |
| Case-control | 6 | 3938 | 1.12 (-0.41, 2.66) | 0.15 |  | 19.29 | 0.002 | 74.10 | 0.13 |  | 0.35 | 72.10 | 0.30 |  |  |  |  |  |  |  |  |  |  |  |  |  |  |  |  |  |  |  |  |  |  |  |  |  |  |  |  |  |  |  |  |  |  |  |  |  |  |  |  |  |
| Cross-sectional | 6 | 2990 | 0.18 (-1.45, 1.83) | 0.82 |  | 18.11 | 0.003 | 72.40 |  |  |  |  |  |  |  |  |  |  |  |  |  |  |  |  |  |  |  |  |  |  |  |  |  |  |  |  |  |  |  |  |  |  |  |  |  |  |  |  |  |  |  |  |  |  |
| Adjustment of confounders | | | | | | | | | | | | | |  |  |  |  |  |  |  |  |  |  |  |  |  |  |  |  |  |  |  |  |  |  |  |  |  |  |  |  |  |  |  |  |  |  |  |  |  |  |  |  |  |
| Adjusted | 4 | 4042 | 1.11 (-0.61, 2.83) | 0.20 |  | 9.45 | 0.02 | 68.20 | 0.64 |  | 0.28 | 71.66 | 0.41 |  |  |  |  |  |  |  |  |  |  |  |  |  |  |  |  |  |  |  |  |  |  |  |  |  |  |  |  |  |  |  |  |  |  |  |  |  |  |  |  |  |
| Unadjusted | 8 | 2886 | 0.37 (-1.18, 1.93) | 0.63 |  | 29.98 | <0.001 | 76.70 |  |  |  |  |  |  |  |  |  |  |  |  |  |  |  |  |  |  |  |  |  |  |  |  |  |  |  |  |  |  |  |  |  |  |  |  |  |  |  |  |  |  |  |  |  |  |
| **Dominant model (AA+GA vs. GG)** | | | | | | | | | | | | | |  |  |  |  |  |  |  |  |  |  |  |  |  |  |  |  |  |  |  |  |  |  |  |  |  |  |  |  |  |  |  |  |  |  |  |  |  |  |  |  |  |
| Ethnicity | | | | | | | | | | | | | |  |  |  |  |  |  |  |  |  |  |  |  |  |  |  |  |  |  |  |  |  |  |  |  |  |  |  |  |  |  |  |  |  |  |  |  |  |  |  |  |  |
| Asian | 5 | 2317 | -0.29 (-0.43, -0.14) | **<0.001** |  | 0.58 | 0.96 | 0.00 | 0.02 |  | 0.19 | 58.64 | 0.64 |  |  |  |  |  |  |  |  |  |  |  |  |  |  |  |  |  |  |  |  |  |  |  |  |  |  |  |  |  |  |  |  |  |  |  |  |  |  |  |  |  |
| Caucasian | 2 | 650 | 1.46 (-1.95, 4.88) | 0.65 |  | 1.68 | 0.19 | 40.40 |  |  |  |  |  |  |  |  |  |  |  |  |  |  |  |  |  |  |  |  |  |  |  |  |  |  |  |  |  |  |  |  |  |  |  |  |  |  |  |  |  |  |  |  |  |  |
| Mix | 5 | 497 | 1.20 (-4.02, 6.43) | 0.40 |  | 18.41 | 0.001 | 78.30 |  |  |  |  |  |  |  |  |  |  |  |  |  |  |  |  |  |  |  |  |  |  |  |  |  |  |  |  |  |  |  |  |  |  |  |  |  |  |  |  |  |  |  |  |  |  |
| Participants’ health status | | | | | | | | | | | | | |  |  |  |  |  |  |  |  |  |  |  |  |  |  |  |  |  |  |  |  |  |  |  |  |  |  |  |  |  |  |  |  |  |  |  |  |  |  |  |  |  |
| Heart disease | 3 | 398 | -0.10 (-2.58, 2.37) | 0.93 |  | 0.35 | 0.83 | 0.00 | 0.15 |  | 0.24 | 61.85 | 0.48 |  |  |  |  |  |  |  |  |  |  |  |  |  |  |  |  |  |  |  |  |  |  |  |  |  |  |  |  |  |  |  |  |  |  |  |  |  |  |  |  |  |
| Healthy | 3 | 882 | -0.60 (-4.99, 3.78) | 0.78 |  | 14.42 | 0.001 | 86.10 |  |  |  |  |  |  |  |  |  |  |  |  |  |  |  |  |  |  |  |  |  |  |  |  |  |  |  |  |  |  |  |  |  |  |  |  |  |  |  |  |  |  |  |  |  |  |
| Others | 6 | 2184 | 1.10 (-0.85, 3.05) | 0.26 |  | 9.83 | 0.08 | 49.10 |  |  |  |  |  |  |  |  |  |  |  |  |  |  |  |  |  |  |  |  |  |  |  |  |  |  |  |  |  |  |  |  |  |  |  |  |  |  |  |  |  |  |  |  |  |  |
| Hardy-Weinberg equilibrium | | | | | | | | | | | | | |  |  |  |  |  |  |  |  |  |  |  |  |  |  |  |  |  |  |  |  |  |  |  |  |  |  |  |  |  |  |  |  |  |  |  |  |  |  |  |  |  |
| Equilibrium | 9 | 3090 | 0.53 (-1.11, 2.18) | 0.52 |  | 28.22 | <0.001 | 71.70 | 0.60 |  | 0.15 | 59.36 | 0.77 |  |  |  |  |  |  |  |  |  |  |  |  |  |  |  |  |  |  |  |  |  |  |  |  |  |  |  |  |  |  |  |  |  |  |  |  |  |  |  |  |  |
| Disequilibrium | 1 | 174 | 1.06 (-1.84, 3.97) | 0.47 |  | - | - | - |  |  |  |  |  |  |  |  |  |  |  |  |  |  |  |  |  |  |  |  |  |  |  |  |  |  |  |  |  |  |  |  |  |  |  |  |  |  |  |  |  |  |  |  |  |  |
| NM | 3 | 200 | -1.11 (-5.14, 2.92) | 0.58 |  | 0.05 | 0.97 | 0.00 |  |  |  |  |  |  |  |  |  |  |  |  |  |  |  |  |  |  |  |  |  |  |  |  |  |  |  |  |  |  |  |  |  |  |  |  |  |  |  |  |  |  |  |  |  |  |
| Quality score | | | | | | | | | | | | | |  |  |  |  |  |  |  |  |  |  |  |  |  |  |  |  |  |  |  |  |  |  |  |  |  |  |  |  |  |  |  |  |  |  |  |  |  |  |  |  |  |
| High quality | 6 | 2561 | 0.85 (-0.94, 2.66) | 0.35 |  | 10.04 | 0.07 | 50.20 | 0.05 |  | 0.05 | 58.81 | 0.91 |  |  |  |  |  |  |  |  |  |  |  |  |  |  |  |  |  |  |  |  |  |  |  |  |  |  |  |  |  |  |  |  |  |  |  |  |  |  |  |  |  |
| Medium quality | 6 | 903 | -0.21 (-2.79, 2.36) | 0.87 |  | 14.46 | 0.01 | 65.40 |  |  |  |  |  |  |  |  |  |  |  |  |  |  |  |  |  |  |  |  |  |  |  |  |  |  |  |  |  |  |  |  |  |  |  |  |  |  |  |  |  |  |  |  |  |  |
| Design of study | | | | | | | | | | | | | |  |  |  |  |  |  |  |  |  |  |  |  |  |  |  |  |  |  |  |  |  |  |  |  |  |  |  |  |  |  |  |  |  |  |  |  |  |  |  |  |  |
| Case-control | 6 | 1969 | -0.23 (-0.68, 0.22) | 0.31 |  | 5.10 | 0.40 | 1.90 | 0.25 |  | 0.16 | 59.90 | 0.75 |  |  |  |  |  |  |  |  |  |  |  |  |  |  |  |  |  |  |  |  |  |  |  |  |  |  |  |  |  |  |  |  |  |  |  |  |  |  |  |  |  |
| Cross-sectional | 6 | 1495 | 0.43 (-2.58, 3.45) | 0.77 |  | 21.91 | 0.001 | 77.20 |  |  |  |  |  |  |  |  |  |  |  |  |  |  |  |  |  |  |  |  |  |  |  |  |  |  |  |  |  |  |  |  |  |  |  |  |  |  |  |  |  |  |  |  |  |  |
| Adjustment of confounders | | | | | | | | | | | | | |  | | | |  | | | |  |  |  |  |  |  |  |  |  |  |  |  |  |  |  |  |  |  |  |  |  |  |  |  |  |  |  |  |  |  |  |  |  |
| Adjusted | 4 | 2021 | 1.62 (-1.39, 4.65) | 0.29 |  | 9.97 | 0.01 | 69.90 | 0.10 |  | 0.04 | 59.09 | 0.92 |  |  |  |  |  |  |  |  |  |  |  |  |  |  |  |  |  |  |  |  |  |  |  |  |  |  |  |  |  |  |  |  |  |  |  |  |  |  |  |  |  |
| Unadjusted | 8 | 1443 | -0.28 (-2.27, 1.71) | 0.78 |  | 15.69 | 0.02 | 55.40 |  |  |  |  |  |  |  |  |  |  |  |  |  |  |  |  |  |  |  |  |  |  |  |  |  |  |  |  |  |  |  |  |  |  |  |  |  |  |  |  |  |  |  |  |  |  |
| **Recessive model (AA vs. GA + GG)** | | | | | | | | | | | | | |  |  |  |  |  |  |  |  |  |  |  |  |  |  |  |  |  |  |  |  |  |  |  |  |  |  |  |  |  |  |  |  |  |  |  |  |  |  |  |  |  |
| Ethnicity | | | | | | | | | | | | | |  |  |  |  |  |  |  |  |  |  |  |  |  |  |  |  |  |  |  |  |  |  |  |  |  |  |  |  |  |  |  |  |  |  |  |  |  |  |  |  |  |
| Asian | 5 | 2317 | 0.29 (-0.29, 0.89) | 0.32 |  | 4.08 | 0.39 | 1.90 | 0.55 |  | 0.73 | 61.97 | 0.09 |  |  |  |  |  |  |  |  |  |  |  |  |  |  |  |  |  |  |  |  |  |  |  |  |  |  |  |  |  |  |  |  |  |  |  |  |  |  |  |  |  |
| Caucasian | 2 | 650 | 0.75 (-3.38, 4.90) | 0.72 |  | 3.04 | 0.08 | 67.10 |  |  |  |  |  |  |  |  |  |  |  |  |  |  |  |  |  |  |  |  |  |  |  |  |  |  |  |  |  |  |  |  |  |  |  |  |  |  |  |  |  |  |  |  |  |  |
| Mix | 5 | 497 | 2.25 (-2.11, 6.62) | 0.31 |  | 20.74 | <0.001 | 80.70 |  |  |  |  |  |  |  |  |  |  |  |  |  |  |  |  |  |  |  |  |  |  |  |  |  |  |  |  |  |  |  |  |  |  |  |  |  |  |  |  |  |  |  |  |  |  |
| Participants’ health status | | | | | | | | | | | | | |  |  |  |  |  |  |  |  |  |  |  |  |  |  |  |  |  |  |  |  |  |  |  |  |  |  |  |  |  |  |  |  |  |  |  |  |  |  |  |  |  |
| Heart disease | 3 | 398 | 3.52 (-0.82, 7.87) | 0.11 |  | 0.39 | 0.82 | 0.00 | 0.26 |  | 0.65 | 62.12 | 0.09 |  |  |  |  |  |  |  |  |  |  |  |  |  |  |  |  |  |  |  |  |  |  |  |  |  |  |  |  |  |  |  |  |  |  |  |  |  |  |  |  |  |
| Healthy | 3 | 882 | 0.29 (-1.89, 2.48) | 0.79 |  | 5.17 | 0.07 | 61.30 |  |  |  |  |  |  |  |  |  |  |  |  |  |  |  |  |  |  |  |  |  |  |  |  |  |  |  |  |  |  |  |  |  |  |  |  |  |  |  |  |  |  |  |  |  |  |
| Others | 4 | 2184 | 2.40 (-0.83, 5.64) | 0.14 |  | 20.80 | 0.001 | 76.00 |  |  |  |  |  |  |  |  |  |  |  |  |  |  |  |  |  |  |  |  |  |  |  |  |  |  |  |  |  |  |  |  |  |  |  |  |  |  |  |  |  |  |  |  |  |  |
| Hardy-Weinberg equilibrium | | | | | | | | | | | | | |  |  |  |  |  |  |  |  |  |  |  |  |  |  |  |  |  |  |  |  |  |  |  |  |  |  |  |  |  |  |  |  |  |  |  |  |  |  |  |  |  |
| Equilibrium | 9 | 3090 | 1.62 (0.23, 3.01) | **0.02** |  | 25.44 | 0.001 | 68.60 | 0.54 |  | 0.65 | 58.94 | 0.30 |  |  |  |  |  |  |  |  |  |  |  |  |  |  |  |  |  |  |  |  |  |  |  |  |  |  |  |  |  |  |  |  |  |  |  |  |  |  |  |  |  |
| Disequilibrium | 1 | 174 | 2.58 (-3.96, 9.13) | 0.43 |  | - | - | - |  |  |  |  |  |  |  |  |  |  |  |  |  |  |  |  |  |  |  |  |  |  |  |  |  |  |  |  |  |  |  |  |  |  |  |  |  |  |  |  |  |  |  |  |  |  |
| NM | 3 | 200 | -1.20 (-5.23, 2.81) | 0.55 |  | 2.20 | 0.33 | 9.00 |  |  |  |  |  |  |  |  |  |  |  |  |  |  |  |  |  |  |  |  |  |  |  |  |  |  |  |  |  |  |  |  |  |  |  |  |  |  |  |  |  |  |  |  |  |  |
| Quality score | | | | | | | | | | | | | |  |  |  |  |  |  |  |  |  |  |  |  |  |  |  |  |  |  |  |  |  |  |  |  |  |  |  |  |  |  |  |  |  |  |  |  |  |  |  |  |  |
| High quality | 6 | 2561 | 0.87 (-0.50, 2.25) | 0.21 |  | 6.45 | 0.26 | 22.50 | 0.74 |  | 1.06 | 61.99 | 0.08 |  |  |  |  |  |  |  |  |  |  |  |  |  |  |  |  |  |  |  |  |  |  |  |  |  |  |  |  |  |  |  |  |  |  |  |  |  |  |  |  |  |
| Medium quality | 6 | 903 | 2.24 (-1.11, 5.60) | 0.19 |  | 22.47 | <0.001 | 77.70 |  |  |  |  |  |  |  |  |  |  |  |  |  |  |  |  |  |  |  |  |  |  |  |  |  |  |  |  |  |  |  |  |  |  |  |  |  |  |  |  |  |  |  |  |  |  |
| Design of study | | | | | | | | | | | | | |  |  | | | |  | | | |  | | |  | | |  | | |  | | |  | | |  | | |  | | |  | | |  | | |  | | |  |  |
| Case-control | 6 | 1969 | 3.53 (-0.07, 7.14) | 0.055 |  | 21.22 | 0.001 | 76.40 | 0.57 |  | 0.72 | 63.03 | 0.23 |  |  |  |  |  |  |  |  |  |  |  |  |  |  |  |  |  |  |  |  |  |  |  |  |  |  |  |  |  |  |  |  |  |  |  |  |  |  |  |  |  |
| Cross-sectional | 6 | 1495 | 0.38 (-1.25, 2.02) | 0.64 |  | 7.50 | 0.18 | 33.30 |  |  |  |  |  |  |  |  |  |  |  |  |  |  |  |  |  |  |  |  |  |  |  |  |  |  |  |  |  |  |  |  |  |  |  |  |  |  |  |  |  |  |  |  |  |  |
| Adjustment of confounders | | | | | | | | | | | | | |  | | | |  | | | |  |  |  |  |  |  |  |  |  |  |  |  |  |  |  |  |  |  |  |  |  |  |  |  |  |  |  |  |  |  |  |  |  |
| Adjusted | 4 | 2021 | 1.30 ( -0.62, 3.23) | 0.18 |  | 5.18 | 0.15 | 42.10 | 0.75 |  | 0.92 | 62.01 | 0.10 |  |  |  |  |  |  |  |  |  |  |  |  |  |  |  |  |  |  |  |  |  |  |  |  |  |  |  |  |  |  |  |  |  |  |  |  |  |  |  |  |  |
| Unadjusted | 8 | 1443 | 1.61 (-1.11, 4.34) | 0.24 |  | 23.75 | 0.001 | 70.50 |  |  |  |  |  |  |  |  |  |  |  |  |  |  |  |  |  |  |  |  |  |  |  |  |  |  |  |  |  |  |  |  |  |  |  |  |  |  |  |  |  |  |  |  |  |  |
| **Homozygous model (AA vs. GG)** | | | | | | | | | | | | | |  |  |  |  |  |  |  |  |  |  |  |  |  |  |  |  |  |  |  |  |  |  |  |  |  |  |  |  |  |  |  |  |  |  |  |  |  |  |  |  |  |
| Ethnicity | | | | | | | | | | | | | |  |  |  |  |  |  |  |  |  |  |  |  |  |  |  |  |  |  |  |  |  |  |  |  |  |  |  |  |  |  |  |  |  |  |  |  |  |  |  |  |  |
| Asian | 5 | 1234 | 0.01 (-0.20, 0.22) | 0.92 |  | 3.45 | 0.48 | 0.00 | 0.06 |  | 0.89 | 70.07 | 0.17 |  |  |  |  |  |  |  |  |  |  |  |  |  |  |  |  |  |  |  |  |  |  |  |  |  |  |  |  |  |  |  |  |  |  |  |  |  |  |  |  |  |
| Caucasian | 2 | 326 | 1.46 (-4.28, 7.21) | 0.61 |  | 3.32 | 0.06 | 69.90 |  |  |  |  |  |  |  |  |  |  |  |  |  |  |  |  |  |  |  |  |  |  |  |  |  |  |  |  |  |  |  |  |  |  |  |  |  |  |  |  |  |  |  |  |  |  |
| Mix | 5 | 229 | 2.93 (-4.26, 10.13) | 0.42 |  | 24.68 | <0.001 | 83.80 |  |  |  |  |  |  |  |  |  |  |  |  |  |  |  |  |  |  |  |  |  |  |  |  |  |  |  |  |  |  |  |  |  |  |  |  |  |  |  |  |  |  |  |  |  |  |
| Participants’ health status | | | | | | | | | | | | | |  |  |  |  |  |  |  |  |  |  |  |  |  |  |  |  |  |  |  |  |  |  |  |  |  |  |  |  |  |  |  |  |  |  |  |  |  |  |  |  |  |
| Heart disease | 3 | 155 | 2.81 (-1.92, 7.56) | 0.24 |  | 0.30 | 0.86 | 0.00 | 0.09 |  | 0.84 | 70.10 | 0.13 |  |  |  |  |  |  |  |  |  |  |  |  |  |  |  |  |  |  |  |  |  |  |  |  |  |  |  |  |  |  |  |  |  |  |  |  |  |  |  |  |  |
| Healthy | 3 | 454 | -0.29 (-5.22, 4.63) | 0.90 |  | 13.70 | 0.001 | 85.40 |  |  |  |  |  |  |  |  |  |  |  |  |  |  |  |  |  |  |  |  |  |  |  |  |  |  |  |  |  |  |  |  |  |  |  |  |  |  |  |  |  |  |  |  |  |  |
| Others | 6 | 1180 | 3.47 (-0.49, 7.44) | 0.08 |  | 18.10 | 0.003 | 72.40 |  |  |  |  |  |  |  |  |  |  |  |  |  |  |  |  |  |  |  |  |  |  |  |  |  |  |  |  |  |  |  |  |  |  |  |  |  |  |  |  |  |  |  |  |  |  |
| Hardy-Weinberg equilibrium | | | | | | | | | | | | | |  |  |  |  |  |  |  |  |  |  |  |  |  |  |  |  |  |  |  |  |  |  |  |  |  |  |  |  |  |  |  |  |  |  |  |  |  |  |  |  |  |
| Equilibrium | 9 | 1643 | 2.20 (-0.18, 4.58) | 0.07 |  | 35.19 | <0.001 | 77.30 | 0.52 |  | 0.76 | 67.64 | 0.36 |  |  |  |  |  |  |  |  |  |  |  |  |  |  |  |  |  |  |  |  |  |  |  |  |  |  |  |  |  |  |  |  |  |  |  |  |  |  |  |  |  |
| Disequilibrium | 1 | 51 | 3.19 (-3.58, 9.98) | 0.35 |  | - | - | - |  |  |  |  |  |  |  |  |  |  |  |  |  |  |  |  |  |  |  |  |  |  |  |  |  |  |  |  |  |  |  |  |  |  |  |  |  |  |  |  |  |  |  |  |  |  |
| NM | 3 | 95 | -1.60 (-6.33, 3.12) | 0.50 |  | 0.59 | 0.74 | 0.00 |  |  |  |  |  |  |  |  |  |  |  |  |  |  |  |  |  |  |  |  |  |  |  |  |  |  |  |  |  |  |  |  |  |  |  |  |  |  |  |  |  |  |  |  |  |  |
| Quality score | | | | | | | | | | | | | |  |  |  |  |  |  |  |  |  |  |  |  |  |  |  |  |  |  |  |  |  |  |  |  |  |  |  |  |  |  |  |  |  |  |  |  |  |  |  |  |  |
| High quality | 6 | 1373 | 1.83 (-0.78, 4.45) | 0.17 |  | 10.70 | 0.058 | 79.20 | 0.15 |  | 1.12 | 70.08 | 0.20 |  |  |  |  |  |  |  |  |  |  |  |  |  |  |  |  |  |  |  |  |  |  |  |  |  |  |  |  |  |  |  |  |  |  |  |  |  |  |  |  |  |
| Medium quality | 6 | 416 | 2.04 (-2.71, 6.79) | 0.40 |  | 24.07 | <0.001 | 53.30 |  |  |  |  |  |  |  |  |  |  |  |  |  |  |  |  |  |  |  |  |  |  |  |  |  |  |  |  |  |  |  |  |  |  |  |  |  |  |  |  |  |  |  |  |  |  |
| Design of study | | | | | | | | | | | | | |  |  |  |  |  |  |  |  |  |  |  |  |  |  |  |  |  |  |  |  |  |  |  |  |  |  |  |  |  |  |  |  |  |  |  |  |  |  |  |  |  |
| Case-control | 6 | 976 | 3.51 (-0.56, 7.59) | 0.09 |  | 15.02 | 0.01 | 66.70 | 0.19 |  | 0.88 | 70.08 | 0.30 |  |  |  |  |  |  |  |  |  |  |  |  |  |  |  |  |  |  |  |  |  |  |  |  |  |  |  |  |  |  |  |  |  |  |  |  |  |  |  |  |  |
| Cross-sectional | 6 | 813 | 0.72 (-2.96, 4.42) | 0.70 |  | 20.12 | 0.001 | 75.20 |  |  |  |  |  |  |  |  |  |  |  |  |  |  |  |  |  |  |  |  |  |  |  |  |  |  |  |  |  |  |  |  |  |  |  |  |  |  |  |  |  |  |  |  |  |  |
| Adjustment of confounders | | | | | | | | | | | | | |  | | | |  | | | |  |  |  |  |  |  |  |  |  |  |  |  |  |  |  |  |  |  |  |  |  |  |  |  |  |  |  |  |  |  |  |  |  |
| Adjusted | 4 | 1047 | 2.76 (-1.00, 6.53) | 0.15 |  | 10.11 | 0.01 | 70.30 | 0.20 |  | 0.95 | 70.09 | 0.23 |  |  |  |  |  |  |  |  |  |  |  |  |  |  |  |  |  |  |  |  |  |  |  |  |  |  |  |  |  |  |  |  |  |  |  |  |  |  |  |  |  |
| Unadjusted | 8 | 742 | 1.43 (-2.23, 5.10) | 0.44 |  | 25.10 | 0.001 | 72.10 |  |  |  |  |  |  |  |  |  |  |  |  |  |  |  |  |  |  |  |  |  |  |  |  |  |  |  |  |  |  |  |  |  |  |  |  |  |  |  |  |  |  |  |  |  |  |
| **Heterozygous model (GA vs. GG)** | | | | | | | | | | | | | |  |  |  |  |  |  |  |  |  |  |  |  |  |  |  |  |  |  |  |  |  |  |  |  |  |  |  |  |  |  |  |  |  |  |  |  |  |  |  |  |  |
| Ethnicity | | | | | | | | | | | | | |  |  |  |  |  |  |  |  |  |  |  |  |  |  |  |  |  |  |  |  |  |  |  |  |  |  |  |  |  |  |  |  |  |  |  |  |  |  |  |  |  |
| Asian | 5 | 2039 | -0.38 (-0.52, -0.23) | **<0.001** |  | 0.20 | 0.99 | 0.00 | 0.01 |  | -0.12 | 48.06 | 0.75 |  |  |  |  |  |  |  |  |  |  |  |  |  |  |  |  |  |  |  |  |  |  |  |  |  |  |  |  |  |  |  |  |  |  |  |  |  |  |  |  |  |
| Caucasian | 2 | 466 | 1.45 (-1.03, 3.93) | 0.25 |  | 0.63 | 0.42 | 0.00 |  |  |  |  |  |  |  |  |  |  |  |  |  |  |  |  |  |  |  |  |  |  |  |  |  |  |  |  |  |  |  |  |  |  |  |  |  |  |  |  |  |  |  |  |  |  |
| Mix | 5 | 347 | 0.50 (-4.25, 5.27) | 0.83 |  | 14.68 | 0.005 | 72.70 |  |  |  |  |  |  |  |  |  |  |  |  |  |  |  |  |  |  |  |  |  |  |  |  |  |  |  |  |  |  |  |  |  |  |  |  |  |  |  |  |  |  |  |  |  |  |
| Participants’ health status | | | | | | | | | | | | | |  |  |  |  |  |  |  |  |  |  |  |  |  |  |  |  |  |  |  |  |  |  |  |  |  |  |  |  |  |  |  |  |  |  |  |  |  |  |  |  |  |
| Heart disease | 3 | 356 | -0.59 (-3.11, 1.91) | 0.64 |  | 0.43 | 0.80 | 0.00 | 0.055 |  | 0.009 | 56.80 | 0.97 |  |  |  |  |  |  |  |  |  |  |  |  |  |  |  |  |  |  |  |  |  |  |  |  |  |  |  |  |  |  |  |  |  |  |  |  |  |  |  |  |  |
| Healthy | 3 | 616 | -0.86 (-5.06, 3.34) | 0.68 |  | 12.11 | 0.002 | 83.50 |  |  |  |  |  |  |  |  |  |  |  |  |  |  |  |  |  |  |  |  |  |  |  |  |  |  |  |  |  |  |  |  |  |  |  |  |  |  |  |  |  |  |  |  |  |  |
| Others | 6 | 1880 | 0.07 (-1.16, 1.31) | 0.90 |  | 6.12 | 0.29 | 18.30 |  |  |  |  |  |  |  |  |  |  |  |  |  |  |  |  |  |  |  |  |  |  |  |  |  |  |  |  |  |  |  |  |  |  |  |  |  |  |  |  |  |  |  |  |  |  |
| Hardy-Weinberg equilibrium | | | | | | | | | | | | | |  |  |  |  |  |  |  |  |  |  |  |  |  |  |  |  |  |  |  |  |  |  |  |  |  |  |  |  |  |  |  |  |  |  |  |  |  |  |  |  |  |
| Equilibrium | 9 | 2545 | -0.15 (-1.72, 1.41) | 0.84 |  | 24.02 | 0.002 | 66.70 | 0.72 |  | -0.08 | 53.09 | 0.87 |  |  |  |  |  |  |  |  |  |  |  |  |  |  |  |  |  |  |  |  |  |  |  |  |  |  |  |  |  |  |  |  |  |  |  |  |  |  |  |  |  |
| Disequilibrium | 1 | 158 | 0.78 (-2.15, 3.72) | 0.59 |  | - | - | - |  |  |  |  |  |  |  |  |  |  |  |  |  |  |  |  |  |  |  |  |  |  |  |  |  |  |  |  |  |  |  |  |  |  |  |  |  |  |  |  |  |  |  |  |  |  |
| NM | 3 | 149 | -0.74 (-4.97, 3.49) | 0.73 |  | 0.44 | 0.80 | 0.00 |  |  |  |  |  |  |  |  |  |  |  |  |  |  |  |  |  |  |  |  |  |  |  |  |  |  |  |  |  |  |  |  |  |  |  |  |  |  |  |  |  |  |  |  |  |  |
| Quality score | | | | | | | | | | | | | |  |  |  |  |  |  |  |  |  |  |  |  |  |  |  |  |  |  |  |  |  |  |  |  |  |  |  |  |  |  |  |  |  |  |  |  |  |  |  |  |  |
| High quality | 6 | 2142 | 0.27 (-1.09, 1.65) | 0.69 |  | 6.94 | 0.054 | 27.90 | 0.01 |  | -0.34 | 49.33 | 0.46 |  |  |  |  |  |  |  |  |  |  |  |  |  |  |  |  |  |  |  |  |  |  |  |  |  |  |  |  |  |  |  |  |  |  |  |  |  |  |  |  |  |
| Medium quality | 6 | 710 | -0.98 (-3.25, 1.27) | 0.39 |  | 10.89 | 0.22 | 54.10 |  |  |  |  |  |  |  |  |  |  |  |  |  |  |  |  |  |  |  |  |  |  |  |  |  |  |  |  |  |  |  |  |  |  |  |  |  |  |  |  |  |  |  |  |  |  |
| Design of study | | | | | | | | | | | | | |  | |  | | | |  | | | |  | | |  | | |  | | |  | | |  | | |  | | |  | | |  | | |  | | |  | | |  |
| Case-control | 6 | 1695 | -0.37 (-0.52, -0.23) | **<0.001** |  | 2.73 | 0.74 | 0.00 | 0.10 |  | -0.15 | 51.76 | 0.74 |  |  |  |  |  |  |  |  |  |  |  |  |  |  |  |  |  |  |  |  |  |  |  |  |  |  |  |  |  |  |  |  |  |  |  |  |  |  |  |  |  |
| Cross-sectional | 6 | 1157 | 0.18 (-2.76, 3.13) | 0.90 |  | 19.05 | 0.002 | 73.80 |  |  |  |  |  |  |  |  |  |  |  |  |  |  |  |  |  |  |  |  |  |  |  |  |  |  |  |  |  |  |  |  |  |  |  |  |  |  |  |  |  |  |  |  |  |  |
| Adjustment of confounders | | | | | | | | | | | | | |  | | | |  | | | |  |  |  |  |  |  |  |  |  |  |  |  |  |  |  |  |  |  |  |  |  |  |  |  |  |  |  |  |  |  |  |  |  |
| Adjusted | 4 | 1613 | 0.90 (-1.63, 3.43) | 0.48 |  | 6.82 | 0.07 | 45.10 | 0.02 |  | -0.28 | 49.89 | 0.50 |  |  |  |  |  |  |  |  |  |  |  |  |  |  |  |  |  |  |  |  |  |  |  |  |  |  |  |  |  |  |  |  |  |  |  |  |  |  |  |  |  |
| Unadjusted | 8 | 1239 | -0.86 (-2.68, 0.95) | 0.35 |  | 12.75 | 0.07 | 56.00 |  |  |  |  |  |  |  |  |  |  |  |  |  |  |  |  |  |  |  |  |  |  |  |  |  |  |  |  |  |  |  |  |  |  |  |  |  |  |  |  |  |  |  |  |  |  |

^a^ All analysis was done using random effects model. WMD, weighted mean difference; 95% CI, 95% confidence interval; NM, not mention.

**Supplementary Table S7-**The association between *CD36* rs1761667 polymorphism and LDL cholesterol indices based on several subgroups. All analyses were conducted using a random-effects model^a^

|  | **No. of data-sets** | **No. of subjects** | **Meta-analysis** | |  | **Heterogeneity** | | | |  | **Meta-regression** | | |  |  |  |  |  |  |  |  |  |  |  |  |  |  |  |  |  |  |  |  |  |  |  |  |  |  |  |  |  |  |  |  |  |  |  |  |  |  |  |  |  |
| --- | --- | --- | --- | --- | --- | --- | --- | --- | --- | --- | --- | --- | --- | --- | --- | --- | --- | --- | --- | --- | --- | --- | --- | --- | --- | --- | --- | --- | --- | --- | --- | --- | --- | --- | --- | --- | --- | --- | --- | --- | --- | --- | --- | --- | --- | --- | --- | --- | --- | --- | --- | --- | --- | --- |
|  |  |  | **WMD^2^ (95%CI)** | **P _effect_** |  | ***Q* statistic** | **P _within_** | **I^2^ (%)** | **P _between group_** |  | **β** | **I^2^_residual (%)_** | **P-value** |  |  |  |  |  |  |  |  |  |  |  |  |  |  |  |  |  |  |  |  |  |  |  |  |  |  |  |  |  |  |  |  |  |  |  |  |  |  |  |  |  |
| **LDL cholesterol (mg/dl)** | | | | | | | | | | | | | |  |  |  |  |  |  |  |  |  |  |  |  |  |  |  |  |  |  |  |  |  |  |  |  |  |  |  |  |  |  |  |  |  |  |  |  |  |  |  |  |  |
| **Allelic model (A vs. G)** | | | | | | | | | | | | | |  |  |  |  |  |  |  |  |  |  |  |  |  |  |  |  |  |  |  |  |  |  |  |  |  |  |  |  |  |  |  |  |  |  |  |  |  |  |  |  |  |
| Ethnicity | | | | | | | | | | | | | |  |  |  |  |  |  |  |  |  |  |  |  |  |  |  |  |  |  |  |  |  |  |  |  |  |  |  |  |  |  |  |  |  |  |  |  |  |  |  |  |  |
| Asian | 4 | 3644 | 1.32 (1.18, 1.45) | **<0.001** |  | 2.37 | 0.49 | 0.00 | <0.001 |  | -0.74 | 78.72 | 0.54 |  |  |  |  |  |  |  |  |  |  |  |  |  |  |  |  |  |  |  |  |  |  |  |  |  |  |  |  |  |  |  |  |  |  |  |  |  |  |  |  |  |
| Caucasian | 1 | 216 | 5.03 (-14.54, 4.47) | 0.29 |  | - | - | - |  |  |  |  |  |  |  |  |  |  |  |  |  |  |  |  |  |  |  |  |  |  |  |  |  |  |  |  |  |  |  |  |  |  |  |  |  |  |  |  |  |  |  |  |  |  |
| Mix | 5 | 1310 | -2.24 (-11.11, 6.62) | 0.61 |  | 34.91 | <0.001 | 88.50 |  |  |  |  |  |  |  |  |  |  |  |  |  |  |  |  |  |  |  |  |  |  |  |  |  |  |  |  |  |  |  |  |  |  |  |  |  |  |  |  |  |  |  |  |  |  |
| Participants’ health status | | | | | | | | | | | | | |  |  |  |  |  |  |  |  |  |  |  |  |  |  |  |  |  |  |  |  |  |  |  |  |  |  |  |  |  |  |  |  |  |  |  |  |  |  |  |  |  |
| Heart disease | 3 | 796 | -1.13 (-5.95, 3.68) | 0.64 |  | 1.43 | 0.48 | 0.00 | <0.001 |  | -1.04 | 84.88 | 0.40 |  |  |  |  |  |  |  |  |  |  |  |  |  |  |  |  |  |  |  |  |  |  |  |  |  |  |  |  |  |  |  |  |  |  |  |  |  |  |  |  |  |
| Healthy | 3 | 1086 | 5.43 (0.16, 10.70) | **0.04** |  | 6.64 | 0.03 | 69.90 |  |  |  |  |  |  |  |  |  |  |  |  |  |  |  |  |  |  |  |  |  |  |  |  |  |  |  |  |  |  |  |  |  |  |  |  |  |  |  |  |  |  |  |  |  |  |
| Others | 4 | 3288 | -7.20 (-17.79, 3.38) | 0.18 |  | 20.43 | <0.001 | 85.30 |  |  |  |  |  |  |  |  |  |  |  |  |  |  |  |  |  |  |  |  |  |  |  |  |  |  |  |  |  |  |  |  |  |  |  |  |  |  |  |  |  |  |  |  |  |  |
| Hardy-Weinberg equilibrium | | | | | | | | | | | | | |  |  |  |  |  |  |  |  |  |  |  |  |  |  |  |  |  |  |  |  |  |  |  |  |  |  |  |  |  |  |  |  |  |  |  |  |  |  |  |  |  |
| Equilibrium | 7 | 4106 | -2.13 (-7.05, 2.77) | 0.39 |  | 45.03 | <0.001 | 86.70 | 0.27 |  | -0.21 | 81.29 | 0.88 |  |  |  |  |  |  |  |  |  |  |  |  |  |  |  |  |  |  |  |  |  |  |  |  |  |  |  |  |  |  |  |  |  |  |  |  |  |  |  |  |  |
| Disequilibrium | 1 | 348 | 2.01 (-4.65, 8.67) | 0.55 |  | - | - | - |  |  |  |  |  |  |  |  |  |  |  |  |  |  |  |  |  |  |  |  |  |  |  |  |  |  |  |  |  |  |  |  |  |  |  |  |  |  |  |  |  |  |  |  |  |  |
| NM | 3 | 716 | 0.73 (-9.48, 10.94) | 0.88 |  | 8.27 | 0.01 | 75.80 |  |  |  |  |  |  |  |  |  |  |  |  |  |  |  |  |  |  |  |  |  |  |  |  |  |  |  |  |  |  |  |  |  |  |  |  |  |  |  |  |  |  |  |  |  |  |
| Quality score | | | | | | | | | | | | | |  |  |  |  |  |  |  |  |  |  |  |  |  |  |  |  |  |  |  |  |  |  |  |  |  |  |  |  |  |  |  |  |  |  |  |  |  |  |  |  |  |
| High quality | 4 | 3364 | -0.62 (-4.40, 3.14) | 0.57 |  | 11.27 | 0.01 | 73.40 | 0.01 |  | -1.28 | 82.47 | 0.44 |  |  |  |  |  |  |  |  |  |  |  |  |  |  |  |  |  |  |  |  |  |  |  |  |  |  |  |  |  |  |  |  |  |  |  |  |  |  |  |  |  |
| Medium quality | 6 | 1806 | -3.53 (-11.50, 4.43) | 0.38 |  | 38.64 | <0.001 | 87.10 |  |  |  |  |  |  |  |  |  |  |  |  |  |  |  |  |  |  |  |  |  |  |  |  |  |  |  |  |  |  |  |  |  |  |  |  |  |  |  |  |  |  |  |  |  |  |
| Design of study | | | | | | | | | | | | | |  | | |  | | | |  | | | |  | | |  | | |  | | |  | | |  | | |  | | |  | | |  | | |  | | |  | | |
| Case-control | 6 | 3938 | -3.98 (-9.72, 1.76) | 0.17 |  | 20.72 | 0.001 | 75.90 | <0.001 |  | -0.36 | 80.23 | 0.84 |  |  |  |  |  |  |  |  |  |  |  |  |  |  |  |  |  |  |  |  |  |  |  |  |  |  |  |  |  |  |  |  |  |  |  |  |  |  |  |  |  |
| Cross-sectional | 4 | 1232 | 3.15 (-2.87, 9.17) | 0.30 |  | 11.87 | 0.008 | 74.70 |  |  |  |  |  |  |  |  |  |  |  |  |  |  |  |  |  |  |  |  |  |  |  |  |  |  |  |  |  |  |  |  |  |  |  |  |  |  |  |  |  |  |  |  |  |  |
| Adjustment of confounders | | | | | | | | | | | | | |  |  |  |  |  |  |  |  |  |  |  |  |  |  |  |  |  |  |  |  |  |  |  |  |  |  |  |  |  |  |  |  |  |  |  |  |  |  |  |  |  |
| Adjusted | 3 | 2958 | -0.66 (-5.80, 4.47) | 0.79 |  | 2.87 | 0.23 | 30.40 | 0.001 |  | -0.83 | 81.44 | 0.60 |  |  |  |  |  |  |  |  |  |  |  |  |  |  |  |  |  |  |  |  |  |  |  |  |  |  |  |  |  |  |  |  |  |  |  |  |  |  |  |  |  |
| Unadjusted | 7 | 2212 | -1.32 (-7.63, 4.99) | 0.68 |  | 41.47 | <0.001 | 85.50 |  |  |  |  |  |  |  |  |  |  |  |  |  |  |  |  |  |  |  |  |  |  |  |  |  |  |  |  |  |  |  |  |  |  |  |  |  |  |  |  |  |  |  |  |  |  |
| **Dominant model (AA+GA vs. GG)** | | | | | | | | | | | | | |  |  |  |  |  |  |  |  |  |  |  |  |  |  |  |  |  |  |  |  |  |  |  |  |  |  |  |  |  |  |  |  |  |  |  |  |  |  |  |  |  |
| Ethnicity | | | | | | | | | | | | | |  |  |  |  |  |  |  |  |  |  |  |  |  |  |  |  |  |  |  |  |  |  |  |  |  |  |  |  |  |  |  |  |  |  |  |  |  |  |  |  |  |
| Asian | 4 | 1822 | 0.47 (-3.41, 4.36) | 0.80 |  | 3.76 | 0.28 | 20.20 | 0.46 |  | -0.25 | 62.27 | 0.80 |  |  |  |  |  |  |  |  |  |  |  |  |  |  |  |  |  |  |  |  |  |  |  |  |  |  |  |  |  |  |  |  |  |  |  |  |  |  |  |  |  |
| Caucasian | 1 | 108 | -8.20 (-25.18, 8.76) | 0.34 |  | - | - | - |  |  |  |  |  |  |  |  |  |  |  |  |  |  |  |  |  |  |  |  |  |  |  |  |  |  |  |  |  |  |  |  |  |  |  |  |  |  |  |  |  |  |  |  |  |  |
| Mix | 5 | 655 | -3.47 (-13.31, 6.37) | 0.49 |  | 10.24 | 0.03 | 60.90 |  |  |  |  |  |  |  |  |  |  |  |  |  |  |  |  |  |  |  |  |  |  |  |  |  |  |  |  |  |  |  |  |  |  |  |  |  |  |  |  |  |  |  |  |  |  |
| Participants’ health status | | | | | | | | | | | | | |  |  |  |  |  |  |  |  |  |  |  |  |  |  |  |  |  |  |  |  |  |  |  |  |  |  |  |  |  |  |  |  |  |  |  |  |  |  |  |  |  |
| Heart disease | 3 | 398 | -3.75 (-11.63, 4.13) | 0.35 |  | 1.05 | 0.59 | 0.00 | 0.32 |  | -0.28 | 38.54 | 0.76 |  |  |  |  |  |  |  |  |  |  |  |  |  |  |  |  |  |  |  |  |  |  |  |  |  |  |  |  |  |  |  |  |  |  |  |  |  |  |  |  |  |
| Healthy | 3 | 543 | 0.30 (-5.74, 6.36) | 0.92 |  | 3.07 | 0.21 | 34.90 |  |  |  |  |  |  |  |  |  |  |  |  |  |  |  |  |  |  |  |  |  |  |  |  |  |  |  |  |  |  |  |  |  |  |  |  |  |  |  |  |  |  |  |  |  |  |
| Others | 4 | 1644 | -7.15 (-20.42, 6.12) | 0.29 |  | 9.19 | 0.02 | 67.40 |  |  |  |  |  |  |  |  |  |  |  |  |  |  |  |  |  |  |  |  |  |  |  |  |  |  |  |  |  |  |  |  |  |  |  |  |  |  |  |  |  |  |  |  |  |  |
| Hardy-Weinberg equilibrium | | | | | | | | | | | | | |  |  |  |  |  |  |  |  |  |  |  |  |  |  |  |  |  |  |  |  |  |  |  |  |  |  |  |  |  |  |  |  |  |  |  |  |  |  |  |  |  |
| Equilibrium | 7 | 2053 | 0.06 (-4.06, 4.20) | 0.97 |  | 12.11 | 0.06 | 50.50 | 0.33 |  | -0.56 | 50.47 | 0.65 |  |  |  |  |  |  |  |  |  |  |  |  |  |  |  |  |  |  |  |  |  |  |  |  |  |  |  |  |  |  |  |  |  |  |  |  |  |  |  |  |  |
| Disequilibrium | 1 | 174 | -1.08 (-12.17, 10.01) | 0.84 |  | - | - | - |  |  |  |  |  |  |  |  |  |  |  |  |  |  |  |  |  |  |  |  |  |  |  |  |  |  |  |  |  |  |  |  |  |  |  |  |  |  |  |  |  |  |  |  |  |  |
| NM | 3 | 358 | -3.26 (-11.15, 4.63) | 0.41 |  | 1.01 | 0.60 | 0.00 |  |  |  |  |  |  |  |  |  |  |  |  |  |  |  |  |  |  |  |  |  |  |  |  |  |  |  |  |  |  |  |  |  |  |  |  |  |  |  |  |  |  |  |  |  |  |
| Quality score | | | | | | | | | | | | | |  |  |  |  |  |  |  |  |  |  |  |  |  |  |  |  |  |  |  |  |  |  |  |  |  |  |  |  |  |  |  |  |  |  |  |  |  |  |  |  |  |
| High quality | 4 | 1682 | 2.23 (2.07, 2.40) | **<0.001** |  | 2.22 | 0.52 | 0.00 | 0.35 |  | -0.95 | 54.28 | 0.49 |  |  |  |  |  |  |  |  |  |  |  |  |  |  |  |  |  |  |  |  |  |  |  |  |  |  |  |  |  |  |  |  |  |  |  |  |  |  |  |  |  |
| Medium quality | 6 | 903 | -4.91 (-12.70, 2.88) | 0.21 |  | 12.46 | 0.02 | 59.90 |  |  |  |  |  |  |  |  |  |  |  |  |  |  |  |  |  |  |  |  |  |  |  |  |  |  |  |  |  |  |  |  |  |  |  |  |  |  |  |  |  |  |  |  |  |  |
| Design of study | | | | | | | | | | | | | |  |  |  |  |  |  |  |  |  |  |  |  |  |  |  |  |  |  |  |  |  |  |  |  |  |  |  |  |  |  |  |  |  |  |  |  |  |  |  |  |  |
| Case-control | 6 | 1969 | -3.35 (-10.69, 3.97) | 0.36 |  | 11.65 | 0.04 | 57.10 | 0.80 |  | -0.14 | 49.20 | 0.91 |  |  |  |  |  |  |  |  |  |  |  |  |  |  |  |  |  |  |  |  |  |  |  |  |  |  |  |  |  |  |  |  |  |  |  |  |  |  |  |  |  |
| Cross-sectional | 4 | 616 | 0.22 (-5.18, 5.63) | 0.93 |  | 3.83 | 0.28 | 21.70 |  |  |  |  |  |  |  |  |  |  |  |  |  |  |  |  |  |  |  |  |  |  |  |  |  |  |  |  |  |  |  |  |  |  |  |  |  |  |  |  |  |  |  |  |  |  |
| Adjustment of confounders | | | | | | | | | | | | | |  | | | |  | | | |  |  |  |  |  |  |  |  |  |  |  |  |  |  |  |  |  |  |  |  |  |  |  |  |  |  |  |  |  |  |  |  |  |
| Adjusted | 3 | 1432 | 2.23 (2.07, 2.40) | **<0.001** |  | 0.93 | 0.62 | 0.00 | 0.21 |  | -0.95 | 56.95 | 0.46 |  |  |  |  |  |  |  |  |  |  |  |  |  |  |  |  |  |  |  |  |  |  |  |  |  |  |  |  |  |  |  |  |  |  |  |  |  |  |  |  |  |
| Unadjusted | 7 | 1106 | -4.08 (-10.39, 2.23) | 0.20 |  | 13.06 | 0.04 | 54.10 |  |  |  |  |  |  |  |  |  |  |  |  |  |  |  |  |  |  |  |  |  |  |  |  |  |  |  |  |  |  |  |  |  |  |  |  |  |  |  |  |  |  |  |  |  |  |
| **Recessive model (AA vs. GA + GG)** | | | | | | | | | | | | | |  |  |  |  |  |  |  |  |  |  |  |  |  |  |  |  |  |  |  |  |  |  |  |  |  |  |  |  |  |  |  |  |  |  |  |  |  |  |  |  |  |
| Ethnicity | | | | | | | | | | | | | |  |  |  |  |  |  |  |  |  |  |  |  |  |  |  |  |  |  |  |  |  |  |  |  |  |  |  |  |  |  |  |  |  |  |  |  |  |  |  |  |  |
| Asian | 4 | 1822 | 1.19 (-3.60, 5.99) | 0.62 |  | 3.58 | 0.31 | 16.20 | <0.001 |  | -2.17 | 92.92 | 0.45 |  |  |  |  |  |  |  |  |  |  |  |  |  |  |  |  |  |  |  |  |  |  |  |  |  |  |  |  |  |  |  |  |  |  |  |  |  |  |  |  |  |
| Caucasian | 1 | 108 | -4.86 (-19.94, 10.21) | 0.52 |  | - | - | - |  |  |  |  |  |  |  |  |  |  |  |  |  |  |  |  |  |  |  |  |  |  |  |  |  |  |  |  |  |  |  |  |  |  |  |  |  |  |  |  |  |  |  |  |  |  |
| Mix | 5 | 655 | -7.85 (-28.39, 12.68) | 0.45 |  | 98.17 | <0.001 | 95.90 |  |  |  |  |  |  |  |  |  |  |  |  |  |  |  |  |  |  |  |  |  |  |  |  |  |  |  |  |  |  |  |  |  |  |  |  |  |  |  |  |  |  |  |  |  |  |
| Participants’ health status | | | | | | | | | | | | | |  |  |  |  |  |  |  |  |  |  |  |  |  |  |  |  |  |  |  |  |  |  |  |  |  |  |  |  |  |  |  |  |  |  |  |  |  |  |  |  |  |
| Heart disease | 3 | 398 | -3.36 (-24.38, 17.65) | 0.75 |  | 7.50 | 0.02 | 73.30 | <0.001 |  | -2.38 | 93.60 | 0.41 |  |  |  |  |  |  |  |  |  |  |  |  |  |  |  |  |  |  |  |  |  |  |  |  |  |  |  |  |  |  |  |  |  |  |  |  |  |  |  |  |  |
| Healthy | 3 | 543 | 12.77 (1.99, 23.55) | **0.02** |  | 12.70 | 0.002 | 84.30 |  |  |  |  |  |  |  |  |  |  |  |  |  |  |  |  |  |  |  |  |  |  |  |  |  |  |  |  |  |  |  |  |  |  |  |  |  |  |  |  |  |  |  |  |  |  |
| Others | 4 | 1644 | -15.48 (-38.16, 7.19) | 0.18 |  | 48.17 | <0.001 | 93.80 |  |  |  |  |  |  |  |  |  |  |  |  |  |  |  |  |  |  |  |  |  |  |  |  |  |  |  |  |  |  |  |  |  |  |  |  |  |  |  |  |  |  |  |  |  |  |
| Hardy-Weinberg equilibrium | | | | | | | | | | | | | |  |  |  |  |  |  |  |  |  |  |  |  |  |  |  |  |  |  |  |  |  |  |  |  |  |  |  |  |  |  |  |  |  |  |  |  |  |  |  |  |  |
| Equilibrium | 7 | 2053 | -6.12 (-16.56, 4.31) | 0.25 |  | 89.11 | <0.001 | 93.30 | <0.001 |  | -0.26 | 92.48 | 0.93 |  |  |  |  |  |  |  |  |  |  |  |  |  |  |  |  |  |  |  |  |  |  |  |  |  |  |  |  |  |  |  |  |  |  |  |  |  |  |  |  |  |
| Disequilibrium | 1 | 174 | 13.97 (-3.27, 31.21) | 0.11 |  | - | - | - |  |  |  |  |  |  |  |  |  |  |  |  |  |  |  |  |  |  |  |  |  |  |  |  |  |  |  |  |  |  |  |  |  |  |  |  |  |  |  |  |  |  |  |  |  |  |
| NM | 3 | 358 | 0.21 (-26.98, 27.41) | 0.98 |  | 23.34 | <0.001 | 91.40 |  |  |  |  |  |  |  |  |  |  |  |  |  |  |  |  |  |  |  |  |  |  |  |  |  |  |  |  |  |  |  |  |  |  |  |  |  |  |  |  |  |  |  |  |  |  |
| Quality score | | | | | | | | | | | | | |  |  |  |  |  |  |  |  |  |  |  |  |  |  |  |  |  |  |  |  |  |  |  |  |  |  |  |  |  |  |  |  |  |  |  |  |  |  |  |  |  |
| High quality | 4 | 1682 | -0.46 (-16.31, 15.38) | 0.95 |  | 49.36 | <0.001 | 93.90 | <0.001 |  | -2.40 | 93.33 | 0.55 |  |  |  |  |  |  |  |  |  |  |  |  |  |  |  |  |  |  |  |  |  |  |  |  |  |  |  |  |  |  |  |  |  |  |  |  |  |  |  |  |  |
| Medium quality | 6 | 903 | -5.27 (-24.83, 14.28) | 0.59 |  | 74.65 | <0.001 | 93.30 |  |  |  |  |  |  |  |  |  |  |  |  |  |  |  |  |  |  |  |  |  |  |  |  |  |  |  |  |  |  |  |  |  |  |  |  |  |  |  |  |  |  |  |  |  |  |
| Design of study | | | | | | | | | | | | | |  |  | | | |  | | | |  | | |  | | |  | | |  | | |  | | |  | | |  | | |  | | |  | | |  | | |  |  |
| Case-control | 6 | 1969 | -10.17 (-26.66, 6.31) | 0.22 |  | 52.93 | <0.001 | 90.60 | <0.001 |  | -0.58 | 93.11 | 0.89 |  |  |  |  |  |  |  |  |  |  |  |  |  |  |  |  |  |  |  |  |  |  |  |  |  |  |  |  |  |  |  |  |  |  |  |  |  |  |  |  |  |
| Cross-sectional | 4 | 616 | 7.09 (-4.94, 19.13) | 0.24 |  | 23.49 | <0.001 | 87.20 |  |  |  |  |  |  |  |  |  |  |  |  |  |  |  |  |  |  |  |  |  |  |  |  |  |  |  |  |  |  |  |  |  |  |  |  |  |  |  |  |  |  |  |  |  |  |
| Adjustment of confounders | | | | | | | | | | | | | |  | | | |  | | | |  |  |  |  |  |  |  |  |  |  |  |  |  |  |  |  |  |  |  |  |  |  |  |  |  |  |  |  |  |  |  |  |  |
| Adjusted | 3 | 1432 | -8.62 (-22.99, 5.74) | 0.24 |  | 6.93 | 0.03 | 71.10 | <0.001 |  | -1.33 | 93.17 | 0.72 |  |  |  |  |  |  |  |  |  |  |  |  |  |  |  |  |  |  |  |  |  |  |  |  |  |  |  |  |  |  |  |  |  |  |  |  |  |  |  |  |  |
| Unadjusted | 7 | 1106 | -0.72 (-15.75, 14.29) | 0.92 |  | 90.91 | <0.001 | 93.40 |  |  |  |  |  |  |  |  |  |  |  |  |  |  |  |  |  |  |  |  |  |  |  |  |  |  |  |  |  |  |  |  |  |  |  |  |  |  |  |  |  |  |  |  |  |  |
| **Homozygous model (AA vs. GG)** | | | | | | | | | | | | | |  |  |  |  |  |  |  |  |  |  |  |  |  |  |  |  |  |  |  |  |  |  |  |  |  |  |  |  |  |  |  |  |  |  |  |  |  |  |  |  |  |
| Ethnicity | | | | | | | | | | | | | |  |  |  |  |  |  |  |  |  |  |  |  |  |  |  |  |  |  |  |  |  |  |  |  |  |  |  |  |  |  |  |  |  |  |  |  |  |  |  |  |  |
| Asian | 4 | 929 | 1.92 (1.57, 2.28) | **<0.001** |  | 2.48 | 0.47 | 0.00 | 0.003 |  | -3.31 | 79.30 | 0.31 |  |  |  |  |  |  |  |  |  |  |  |  |  |  |  |  |  |  |  |  |  |  |  |  |  |  |  |  |  |  |  |  |  |  |  |  |  |  |  |  |  |
| Caucasian | 1 | 57 | -9.67 (-29.67, 10.33) | 0.34 |  | - | - | - |  |  |  |  |  |  |  |  |  |  |  |  |  |  |  |  |  |  |  |  |  |  |  |  |  |  |  |  |  |  |  |  |  |  |  |  |  |  |  |  |  |  |  |  |  |  |
| Mix | 5 | 309 | -10.86 (-30.90, 9.18) | 0.28 |  | 37.19 | <0.001 | 89.20 |  |  |  |  |  |  |  |  |  |  |  |  |  |  |  |  |  |  |  |  |  |  |  |  |  |  |  |  |  |  |  |  |  |  |  |  |  |  |  |  |  |  |  |  |  |  |
| Participants’ health status | | | | | | | | | | | | | |  |  |  |  |  |  |  |  |  |  |  |  |  |  |  |  |  |  |  |  |  |  |  |  |  |  |  |  |  |  |  |  |  |  |  |  |  |  |  |  |  |
| Heart disease | 3 | 155 | -1.73 (-16.86, 13.39) | 0.82 |  | 2.85 | 0.24 | 29.90 | <0.001 |  | -3.60 | 83.36 | 0.27 |  |  |  |  |  |  |  |  |  |  |  |  |  |  |  |  |  |  |  |  |  |  |  |  |  |  |  |  |  |  |  |  |  |  |  |  |  |  |  |  |  |
| Healthy | 3 | 286 | 9.10 (0.29, 17.91) | **0.04** |  | 2.40 | 0.11 | 54.60 |  |  |  |  |  |  |  |  |  |  |  |  |  |  |  |  |  |  |  |  |  |  |  |  |  |  |  |  |  |  |  |  |  |  |  |  |  |  |  |  |  |  |  |  |  |  |
| Others | 4 | 854 | -20.24 (-46.63, 6.14) | 0.13 |  | 26.92 | <0.001 | 88.90 |  |  |  |  |  |  |  |  |  |  |  |  |  |  |  |  |  |  |  |  |  |  |  |  |  |  |  |  |  |  |  |  |  |  |  |  |  |  |  |  |  |  |  |  |  |  |
| Hardy-Weinberg equilibrium | | | | | | | | | | | | | |  |  |  |  |  |  |  |  |  |  |  |  |  |  |  |  |  |  |  |  |  |  |  |  |  |  |  |  |  |  |  |  |  |  |  |  |  |  |  |  |  |
| Equilibrium | 7 | 1069 | -5.25 (-15.13, 4.62) | 0.29 |  | 43.78 | <0.001 | 86.30 | 0.35 |  | -1.30 | 80.21 | 0.72 |  |  |  |  |  |  |  |  |  |  |  |  |  |  |  |  |  |  |  |  |  |  |  |  |  |  |  |  |  |  |  |  |  |  |  |  |  |  |  |  |  |
| Disequilibrium | 1 | 51 | -0.12 (-19.09, 18.84) | 0.22 |  | - | - | - |  |  |  |  |  |  |  |  |  |  |  |  |  |  |  |  |  |  |  |  |  |  |  |  |  |  |  |  |  |  |  |  |  |  |  |  |  |  |  |  |  |  |  |  |  |  |
| NM | 3 | 175 | 11.82 (-7.41, 31.06) | 0.99 |  | 6.07 | 0.04 | 67.00 |  |  |  |  |  |  |  |  |  |  |  |  |  |  |  |  |  |  |  |  |  |  |  |  |  |  |  |  |  |  |  |  |  |  |  |  |  |  |  |  |  |  |  |  |  |  |
| Quality score | | | | | | | | | | | | | |  |  |  |  |  |  |  |  |  |  |  |  |  |  |  |  |  |  |  |  |  |  |  |  |  |  |  |  |  |  |  |  |  |  |  |  |  |  |  |  |  |
| High quality | 4 | 879 | 2.17 (-7.87, 12.22) | 0.67 |  | 7.80 | 0.05 | 61.50 | 0.01 |  | -4.13 | 81.28 | 0.35 |  |  |  |  |  |  |  |  |  |  |  |  |  |  |  |  |  |  |  |  |  |  |  |  |  |  |  |  |  |  |  |  |  |  |  |  |  |  |  |  |  |
| Medium quality | 6 | 416 | -9.51 (-27.73, 8.70) | 0.30 |  | 38.09 | <0.001 | 86.90 |  |  |  |  |  |  |  |  |  |  |  |  |  |  |  |  |  |  |  |  |  |  |  |  |  |  |  |  |  |  |  |  |  |  |  |  |  |  |  |  |  |  |  |  |  |  |
| Design of study | | | | | | | | | | | | | |  |  |  |  |  |  |  |  |  |  |  |  |  |  |  |  |  |  |  |  |  |  |  |  |  |  |  |  |  |  |  |  |  |  |  |  |  |  |  |  |  |
| Case-control | 6 | 976 | -11.11 (-26.98, 4.76) | 0.17 |  | 27.97 | <0.001 | 82.10 | <0.001 |  | -2.50 | 80.11 | 0.62 |  |  |  |  |  |  |  |  |  |  |  |  |  |  |  |  |  |  |  |  |  |  |  |  |  |  |  |  |  |  |  |  |  |  |  |  |  |  |  |  |  |
| Cross-sectional | 4 | 319 | 5.28 (-5.37, 15.94) | 0.33 |  | 8.43 | 0.03 | 64.40 |  |  |  |  |  |  |  |  |  |  |  |  |  |  |  |  |  |  |  |  |  |  |  |  |  |  |  |  |  |  |  |  |  |  |  |  |  |  |  |  |  |  |  |  |  |  |
| Adjustment of confounders | | | | | | | | | | | | | |  | | | |  | | | |  |  |  |  |  |  |  |  |  |  |  |  |  |  |  |  |  |  |  |  |  |  |  |  |  |  |  |  |  |  |  |  |  |
| Adjusted | 3 | 778 | -2.97 (-14.80, 8.86) | 0.62 |  | 2.98 | 0.22 | 32.80 | 0.002 |  | -3.21 | 80.69 | 0.45 |  |  |  |  |  |  |  |  |  |  |  |  |  |  |  |  |  |  |  |  |  |  |  |  |  |  |  |  |  |  |  |  |  |  |  |  |  |  |  |  |  |
| Unadjusted | 7 | 517 | -4.66 (-18.42, 9.09) | 0.50 |  | 39.40 | <0.001 | 84.80 |  |  |  |  |  |  |  |  |  |  |  |  |  |  |  |  |  |  |  |  |  |  |  |  |  |  |  |  |  |  |  |  |  |  |  |  |  |  |  |  |  |  |  |  |  |  |
| **Heterozygous model (GA vs. GG)** | | | | | | | | | | | | | |  |  |  |  |  |  |  |  |  |  |  |  |  |  |  |  |  |  |  |  |  |  |  |  |  |  |  |  |  |  |  |  |  |  |  |  |  |  |  |  |  |
| Ethnicity | | | | | | | | | | | | | |  |  |  |  |  |  |  |  |  |  |  |  |  |  |  |  |  |  |  |  |  |  |  |  |  |  |  |  |  |  |  |  |  |  |  |  |  |  |  |  |  |
| Asian | 4 | 1581 | -0.65 (-5.86, 4.55) | 0.80 |  | 4.54 | 0.20 | 33.90 | <0.001 |  | -1.94 | 83.45 | 0.07 |  |  |  |  |  |  |  |  |  |  |  |  |  |  |  |  |  |  |  |  |  |  |  |  |  |  |  |  |  |  |  |  |  |  |  |  |  |  |  |  |  |
| Caucasian | 1 | 78 | -7.35 (-25.07, 10.37) | 0.41 |  | - | - | - |  |  |  |  |  |  |  |  |  |  |  |  |  |  |  |  |  |  |  |  |  |  |  |  |  |  |  |  |  |  |  |  |  |  |  |  |  |  |  |  |  |  |  |  |  |  |
| Mix | 5 | 459 | -6.48 (-14.85, 1.88) | 0.12 |  | 7.66 | 0.10 | 47.80 |  |  |  |  |  |  |  |  |  |  |  |  |  |  |  |  |  |  |  |  |  |  |  |  |  |  |  |  |  |  |  |  |  |  |  |  |  |  |  |  |  |  |  |  |  |  |
| Participants’ health status | | | | | | | | | | | | | |  |  |  |  |  |  |  |  |  |  |  |  |  |  |  |  |  |  |  |  |  |  |  |  |  |  |  |  |  |  |  |  |  |  |  |  |  |  |  |  |  |
| Heart disease | 3 | 356 | -4.31 (-12.43, 3.80) | 0.29 |  | 2.03 | 0.36 | 1.60 | <0.001 |  | -1.58 | 61.69 | 0.20 |  |  |  |  |  |  |  |  |  |  |  |  |  |  |  |  |  |  |  |  |  |  |  |  |  |  |  |  |  |  |  |  |  |  |  |  |  |  |  |  |  |
| Healthy | 3 | 372 | -6.68 (-13.87, 0.49) | 0.06 |  | 3.84 | 0.14 | 47.90 |  |  |  |  |  |  |  |  |  |  |  |  |  |  |  |  |  |  |  |  |  |  |  |  |  |  |  |  |  |  |  |  |  |  |  |  |  |  |  |  |  |  |  |  |  |  |
| Others | 4 | 1390 | -1.66 (-10.22, 6.90) | 0.70 |  | 4.50 | 0.21 | 33.30 |  |  |  |  |  |  |  |  |  |  |  |  |  |  |  |  |  |  |  |  |  |  |  |  |  |  |  |  |  |  |  |  |  |  |  |  |  |  |  |  |  |  |  |  |  |  |
| Hardy-Weinberg equilibrium | | | | | | | | | | | | | |  |  |  |  |  |  |  |  |  |  |  |  |  |  |  |  |  |  |  |  |  |  |  |  |  |  |  |  |  |  |  |  |  |  |  |  |  |  |  |  |  |
| Equilibrium | 7 | 1699 | -2.24 (-7.06, 2.57) | 0.36 |  | 15.09 | 0.02 | 60.20 | 0.01 |  | -2.34 | 72.37 | 0.08 |  |  |  |  |  |  |  |  |  |  |  |  |  |  |  |  |  |  |  |  |  |  |  |  |  |  |  |  |  |  |  |  |  |  |  |  |  |  |  |  |  |
| Disequilibrium | 1 | 158 | -2.75 (-13.96, 8.44) | 0.62 |  | - | - | - |  |  |  |  |  |  |  |  |  |  |  |  |  |  |  |  |  |  |  |  |  |  |  |  |  |  |  |  |  |  |  |  |  |  |  |  |  |  |  |  |  |  |  |  |  |  |
| NM | 3 | 261 | -6.38 (-19.07, 6.30) | 0.32 |  | 3.73 | 0.15 | 46.40 |  |  |  |  |  |  |  |  |  |  |  |  |  |  |  |  |  |  |  |  |  |  |  |  |  |  |  |  |  |  |  |  |  |  |  |  |  |  |  |  |  |  |  |  |  |  |
| Quality score | | | | | | | | | | | | | |  |  |  |  |  |  |  |  |  |  |  |  |  |  |  |  |  |  |  |  |  |  |  |  |  |  |  |  |  |  |  |  |  |  |  |  |  |  |  |  |  |
| High quality | 4 | 1408 | -3.98 (-8.77, 0.80) | 0.69 |  | 10.93 | 0.01 | 72.50 | <0.001 |  | -2.53 | 77.25 | 0.10 |  |  |  |  |  |  |  |  |  |  |  |  |  |  |  |  |  |  |  |  |  |  |  |  |  |  |  |  |  |  |  |  |  |  |  |  |  |  |  |  |  |
| Medium quality | 6 | 710 | -4.00 (-7.33, -0.67) | **0.01** |  | 2.96 | 0.70 | 0.00 |  |  |  |  |  |  |  |  |  |  |  |  |  |  |  |  |  |  |  |  |  |  |  |  |  |  |  |  |  |  |  |  |  |  |  |  |  |  |  |  |  |  |  |  |  |  |
| Design of study | | | | | | | | | | | | | |  | |  | | | |  | | | |  | | |  | | |  | | |  | | |  | | |  | | |  | | |  | | |  | | |  | | |  |
| Case-control | 6 | 1695 | -1.97 (-8.36, 4.41) | 0.54 |  | 8.88 | 0.11 | 43.70 | <0.001 |  | -2.80 | 77.16 | 0.08 |  |  |  |  |  |  |  |  |  |  |  |  |  |  |  |  |  |  |  |  |  |  |  |  |  |  |  |  |  |  |  |  |  |  |  |  |  |  |  |  |  |
| Cross-sectional | 4 | 423 | -5.93 (-11.42,-0.44) | **0.03** |  | 3.85 | 0.27 | 22.00 |  |  |  |  |  |  |  |  |  |  |  |  |  |  |  |  |  |  |  |  |  |  |  |  |  |  |  |  |  |  |  |  |  |  |  |  |  |  |  |  |  |  |  |  |  |  |
| Adjustment of confounders | | | | | | | | | | | | | |  | | | |  | | | |  |  |  |  |  |  |  |  |  |  |  |  |  |  |  |  |  |  |  |  |  |  |  |  |  |  |  |  |  |  |  |  |  |
| Adjusted | 3 | 1264 | 2.33 (2.17, 2.48) | **<0.001** |  | 0.91 | 0.63 | 0.00 | <0.001 |  | -2.67 | 78.92 | 0.06 |  |  |  |  |  |  |  |  |  |  |  |  |  |  |  |  |  |  |  |  |  |  |  |  |  |  |  |  |  |  |  |  |  |  |  |  |  |  |  |  |  |
| Unadjusted | 7 | 854 | -5.14 (-8.43, -1.84) | 0.002 |  | 6.11 | 0.41 | 1.70 |  |  |  |  |  |  |  |  |  |  |  |  |  |  |  |  |  |  |  |  |  |  |  |  |  |  |  |  |  |  |  |  |  |  |  |  |  |  |  |  |  |  |  |  |  |  |

^a^ All analysis was done using random effects model. WMD, weighted mean difference; 95% CI, 95% confidence interval; NM, not mention.

**Supplementary Table S8-** The association between *CD36* rs1761667 polymorphism and blood pressure based on several subgroups. All analyses were conducted using a random-effects model ^a^

|  | **No. of data-sets** | **No. of subjects** | **Meta-analysis** | |  | **Heterogeneity** | | | |  | **Meta-regression** | | |  |  |  |  |  |  |  |  |  |  |  |  |  |  |  |  |  |  |  |  |  |  |  |  |  |  |  |  |  |  |  |  |  |  |  |  |  |  |  |  |  |
| --- | --- | --- | --- | --- | --- | --- | --- | --- | --- | --- | --- | --- | --- | --- | --- | --- | --- | --- | --- | --- | --- | --- | --- | --- | --- | --- | --- | --- | --- | --- | --- | --- | --- | --- | --- | --- | --- | --- | --- | --- | --- | --- | --- | --- | --- | --- | --- | --- | --- | --- | --- | --- | --- | --- |
|  |  |  | **WMD^2^ (95%CI)** | **P _effect_** |  | ***Q* statistic** | **P _within_** | **I^2^ (%)** | **P _between group_** |  | **β** | **I^2^_residual (%)_** | **P-value** |  |  |  |  |  |  |  |  |  |  |  |  |  |  |  |  |  |  |  |  |  |  |  |  |  |  |  |  |  |  |  |  |  |  |  |  |  |  |  |  |  |
| **Systolic Blood pressure (mmHg)** | | | | | | | | | | | | | |  |  |  |  |  |  |  |  |  |  |  |  |  |  |  |  |  |  |  |  |  |  |  |  |  |  |  |  |  |  |  |  |  |  |  |  |  |  |  |  |  |
| **Allelic model (A vs. G)** | | | | | | | | | | | | | |  |  |  |  |  |  |  |  |  |  |  |  |  |  |  |  |  |  |  |  |  |  |  |  |  |  |  |  |  |  |  |  |  |  |  |  |  |  |  |  |  |
| Ethnicity | | | | | | | | | | | | | |  |  |  |  |  |  |  |  |  |  |  |  |  |  |  |  |  |  |  |  |  |  |  |  |  |  |  |  |  |  |  |  |  |  |  |  |  |  |  |  |  |
| Asian | 2 | 1468 | -0.78 (-2.67, 1.10) | 0.41 |  | 0.91 | 0.34 | 28.40 | <0.001 |  | -1.39 | 87.38 | 0.12 |  |  |  |  |  |  |  |  |  |  |  |  |  |  |  |  |  |  |  |  |  |  |  |  |  |  |  |  |  |  |  |  |  |  |  |  |  |  |  |  |  |
| Caucasian | 2 | 2556 | 0.05 (-1.28, 1.40) | 0.93 |  | 1.40 | 0.23 | 0.00 |  |  |  |  |  |  |  |  |  |  |  |  |  |  |  |  |  |  |  |  |  |  |  |  |  |  |  |  |  |  |  |  |  |  |  |  |  |  |  |  |  |  |  |  |  |  |
| Mix | 1 | 200 | -8.75 (-11.47, -6.02) | <0.001 |  | - | - | - |  |  |  |  |  |  |  |  |  |  |  |  |  |  |  |  |  |  |  |  |  |  |  |  |  |  |  |  |  |  |  |  |  |  |  |  |  |  |  |  |  |  |  |  |  |  |
| Quality score | | | | | | | | | | | | | |  |  |  |  |  |  |  |  |  |  |  |  |  |  |  |  |  |  |  |  |  |  |  |  |  |  |  |  |  |  |  |  |  |  |  |  |  |  |  |  |  |
| High quality | 3 | 3546 | -0.28 (-1.60, 1.03) | 0.67 |  | 2.89 | 0.23 | 30.70 | <0.001 |  | -1.70 | 87.09 | 0.17 |  |  |  |  |  |  |  |  |  |  |  |  |  |  |  |  |  |  |  |  |  |  |  |  |  |  |  |  |  |  |  |  |  |  |  |  |  |  |  |  |  |
| Medium quality | 2 | 678 | -4.35 (-12.94, 4.24) | 0.32 |  | 21.54 | <0.001 | 95.40 |  |  |  |  |  |  |  |  |  |  |  |  |  |  |  |  |  |  |  |  |  |  |  |  |  |  |  |  |  |  |  |  |  |  |  |  |  |  |  |  |  |  |  |  |  |  |
| Design of study | | | | | | | | | | | | | |  |  |  |  |  |  |  |  |  |  |  |  |  |  |  |  |  |  |  |  |  |  |  |  |  |  |  |  |  |  |  |  |  |  |  |  |  |  |  |  |  |
| Case-control | 3 | 2150 | -3.08 (-8.04, 1.88) | 0.22 |  | 28.09 | <0.001 | 92.90 | 0.01 |  | -1.02 | 90.14 | 0.45 |  |  |  |  |  |  |  |  |  |  |  |  |  |  |  |  |  |  |  |  |  |  |  |  |  |  |  |  |  |  |  |  |  |  |  |  |  |  |  |  |  |
| Cross-sectional | 2 | 2074 | -0.27 (-2.66, 2.12) | 0.82 |  | 2.31 | 0.12 | 56.80 |  |  |  |  |  |  |  |  |  |  |  |  |  |  |  |  |  |  |  |  |  |  |  |  |  |  |  |  |  |  |  |  |  |  |  |  |  |  |  |  |  |  |  |  |  |  |
| Adjustment of confounders | | | | | | | | | | | | | |  |  |  |  |  |  |  |  |  |  |  |  |  |  |  |  |  |  |  |  |  |  |  |  |  |  |  |  |  |  |  |  |  |  |  |  |  |  |  |  |  |
| Adjusted | 2 | 2556 | 0.05 (-1.28, 1.40) | 0.93 |  | 1.40 | 0.23 | 28.40 | <0.001 |  | -1.48 | 87.04 | 0.18 |  |  |  |  |  |  |  |  |  |  |  |  |  |  |  |  |  |  |  |  |  |  |  |  |  |  |  |  |  |  |  |  |  |  |  |  |  |  |  |  |  |
| Unadjusted | 3 | 1668 | -3.51 (-8.81, 1.78) | 0.19 |  | 23.07 | <0.001 | 91.30 |  |  |  |  |  |  |  |  |  |  |  |  |  |  |  |  |  |  |  |  |  |  |  |  |  |  |  |  |  |  |  |  |  |  |  |  |  |  |  |  |  |  |  |  |  |  |
| **Dominant model (AA+GA vs. GG)** | | | | | | | | | | | | | |  |  |  |  |  |  |  |  |  |  |  |  |  |  |  |  |  |  |  |  |  |  |  |  |  |  |  |  |  |  |  |  |  |  |  |  |  |  |  |  |  |
| Ethnicity | | | | | | | | | | | | | |  |  |  |  |  |  |  |  |  |  |  |  |  |  |  |  |  |  |  |  |  |  |  |  |  |  |  |  |  |  |  |  |  |  |  |  |  |  |  |  |  |
| Asian | 2 | 734 | 0.12 (-2.46, 2.70) | 0.92 |  | 0.51 | 0.47 | 0.00 | <0.001 |  | -3.66 | 87.95 | 0.12 |  |  |  |  |  |  |  |  |  |  |  |  |  |  |  |  |  |  |  |  |  |  |  |  |  |  |  |  |  |  |  |  |  |  |  |  |  |  |  |  |  |
| Caucasian | 2 | 1278 | -1.08 (-3.08, 0.90) | 0.28 |  | 0.04 | 0.85 | 0.00 |  |  |  |  |  |  |  |  |  |  |  |  |  |  |  |  |  |  |  |  |  |  |  |  |  |  |  |  |  |  |  |  |  |  |  |  |  |  |  |  |  |  |  |  |  |  |
| Mix | 1 | 100 | -23.94 (-31.19, -16.69) | <0.001 |  | - | - | - |  |  |  |  |  |  |  |  |  |  |  |  |  |  |  |  |  |  |  |  |  |  |  |  |  |  |  |  |  |  |  |  |  |  |  |  |  |  |  |  |  |  |  |  |  |  |
| Quality score | | | | | | | | | | | | | |  |  |  |  |  |  |  |  |  |  |  |  |  |  |  |  |  |  |  |  |  |  |  |  |  |  |  |  |  |  |  |  |  |  |  |  |  |  |  |  |  |
| High quality | 3 | 1773 | -1.01 (-2.76, 0.72) | 0.25 |  | 0.06 | <0.001 | 97.20 | 0.10 |  | -4.09 | 89.21 | 0.22 |  |  |  |  |  |  |  |  |  |  |  |  |  |  |  |  |  |  |  |  |  |  |  |  |  |  |  |  |  |  |  |  |  |  |  |  |  |  |  |  |  |
| Medium quality | 2 | 339 | -11.22 (-35.75, 13.30) | 0.37 |  | 36.29 | 0.97 | 0.00 |  |  |  |  |  |  |  |  |  |  |  |  |  |  |  |  |  |  |  |  |  |  |  |  |  |  |  |  |  |  |  |  |  |  |  |  |  |  |  |  |  |  |  |  |  |  |
| Design of study | | | | | | | | | | | | | |  | |  | | | |  | | | |  | | |  | | |  | | |  | | | |  | | |  | | |  | | |  | | |  | | |  | |  |
| Case-control | 3 | 1075 | -7.44 (-18.08, 3.19) | 0.17 |  | 37.80 | <0.001 | 94.70 | 0.27 |  | -2.32 | 90.17 | 0.51 |  |  |  |  |  |  |  |  |  |  |  |  |  |  |  |  |  |  |  |  |  |  |  |  |  |  |  |  |  |  |  |  |  |  |  |  |  |  |  |  |  |
| Cross-sectional | 2 | 1037 | -0.87 (-3.00, 1.26) | 0.42 |  | 0.00 | 0.95 | 0.00 |  |  |  |  |  |  |  |  |  |  |  |  |  |  |  |  |  |  |  |  |  |  |  |  |  |  |  |  |  |  |  |  |  |  |  |  |  |  |  |  |  |  |  |  |  |  |
| Adjustment of confounders | | | | | | | | | | | | | |  | | | |  | | | | |  |  |  |  |  |  |  |  |  |  |  |  |  |  |  |  |  |  |  |  |  |  |  |  |  |  |  |  |  |  |  |  |
| Adjusted | 2 | 1278 | -1.08 (-3.08,0.90) | 0.28 |  | 0.04 | <0.001 | 94.70 | 0.34 |  | -3.27 | 89.51 | 0.27 |  |  |  |  |  |  |  |  |  |  |  |  |  |  |  |  |  |  |  |  |  |  |  |  |  |  |  |  |  |  |  |  |  |  |  |  |  |  |  |  |  |
| Unadjusted | 3 | 834 | -7.35 (-18.70, 3.98) | 0.20 |  | 38.06 | 0.85 | 0.00 |  |  |  |  |  |  |  |  |  |  |  |  |  |  |  |  |  |  |  |  |  |  |  |  |  |  |  |  |  |  |  |  |  |  |  |  |  |  |  |  |  |  |  |  |  |  |
| **Recessive model (AA vs. GA + GG)** | | | | | | | | | | | | | |  |  |  |  |  |  |  |  |  |  |  |  |  |  |  |  |  |  |  |  |  |  |  |  |  |  |  |  |  |  |  |  |  |  |  |  |  |  |  |  |  |
| Ethnicity | | | | | | | | | | | | | |  |  |  |  |  |  |  |  |  |  |  |  |  |  |  |  |  |  |  |  |  |  |  |  |  |  |  |  |  |  |  |  |  |  |  |  |  |  |  |  |  |
| Asian | 2 | 734 | -4.40 ( -9.69, 0.89) | 0.10 |  | 1.83 | 0.10 | 45.40 | <0.001 |  | -2.86 | 95.83 | 0.13 |  |  |  |  |  |  |  |  |  |  |  |  |  |  |  |  |  |  |  |  |  |  |  |  |  |  |  |  |  |  |  |  |  |  |  |  |  |  |  |  |  |
| Caucasian | 2 | 1278 | 0.96 (-2.03, 3.96) | 0.52 |  | 2.66 | 0.17 | 62.40 |  |  |  |  |  |  |  |  |  |  |  |  |  |  |  |  |  |  |  |  |  |  |  |  |  |  |  |  |  |  |  |  |  |  |  |  |  |  |  |  |  |  |  |  |  |  |
| Mix | 1 | 100 | -16.33 (-18.83, -13.83) | <0.001 |  | - | - | - |  |  |  |  |  |  |  |  |  |  |  |  |  |  |  |  |  |  |  |  |  |  |  |  |  |  |  |  |  |  |  |  |  |  |  |  |  |  |  |  |  |  |  |  |  |  |
| Quality score | | | | | | | | | | | | | |  |  |  |  |  |  |  |  |  |  |  |  |  |  |  |  |  |  |  |  |  |  |  |  |  |  |  |  |  |  |  |  |  |  |  |  |  |  |  |  |  |
| High quality | 3 | 1773 | -0.98 (-5.23, 3.26) | 0.64 |  | 9.20 | 0.01 | 78.30 | <0.001 |  | -3.91 | 95.09 | 0.12 |  |  |  |  |  |  |  |  |  |  |  |  |  |  |  |  |  |  |  |  |  |  |  |  |  |  |  |  |  |  |  |  |  |  |  |  |  |  |  |  |  |
| Medium quality | 2 | 339 | -9.37 (-23.29, 4.55) | 0.18 |  | 27.71 | <0.001 | 96.40 |  |  |  |  |  |  |  |  |  |  |  |  |  |  |  |  |  |  |  |  |  |  |  |  |  |  |  |  |  |  |  |  |  |  |  |  |  |  |  |  |  |  |  |  |  |  |
| Design of study | | | | | | | | | | | | | |  | |  | | | |  | | | |  | | |  | | |  | | |  | | | |  | | |  | | |  | | |  | | |  | | |  | |  |
| Case-control | 3 | 1075 | -6.43 (-17.59, 4.72) | 0.25 |  | 75.00 | <0.001 | 97.30 | <0.001 |  | -2.63 | 97.43 | 0.35 |  |  |  |  |  |  |  |  |  |  |  |  |  |  |  |  |  |  |  |  |  |  |  |  |  |  |  |  |  |  |  |  |  |  |  |  |  |  |  |  |  |
| Cross-sectional | 2 | 1037 | -2.13 (-11.90, 7.63) | 0.66 |  | 8.19 | 0.004 | 87.80 |  |  |  |  |  |  |  |  |  |  |  |  |  |  |  |  |  |  |  |  |  |  |  |  |  |  |  |  |  |  |  |  |  |  |  |  |  |  |  |  |  |  |  |  |  |  |
| Adjustment of confounders | | | | | | | | | | | | | |  | | | |  | | | | |  |  |  |  |  |  |  |  |  |  |  |  |  |  |  |  |  |  |  |  |  |  |  |  |  |  |  |  |  |  |  |  |
| Adjusted | 2 | 1278 | 0.96 (-2.03, 3.96) | 0.52 |  | 2.66 | 0.10 | 62.40 | <0.001 |  | -3.65 | 95.05 | 0.10 |  |  |  |  |  |  |  |  |  |  |  |  |  |  |  |  |  |  |  |  |  |  |  |  |  |  |  |  |  |  |  |  |  |  |  |  |  |  |  |  |  |
| Unadjusted | 3 | 834 | -8.85 (-18.62, 0.90) | 0.07 |  | 30.26 | <0.001 | 93.40 |  |  |  |  |  |  |  |  |  |  |  |  |  |  |  |  |  |  |  |  |  |  |  |  |  |  |  |  |  |  |  |  |  |  |  |  |  |  |  |  |  |  |  |  |  |  |
| **Homozygous model (AA vs. GG)** | | | | | | | | | | | | | |  |  |  |  |  |  |  |  |  |  |  |  |  |  |  |  |  |  |  |  |  |  |  |  |  |  |  |  |  |  |  |  |  |  |  |  |  |  |  |  |  |
| Ethnicity | | | | | | | | | | | | | |  |  |  |  |  |  |  |  |  |  |  |  |  |  |  |  |  |  |  |  |  |  |  |  |  |  |  |  |  |  |  |  |  |  |  |  |  |  |  |  |  |
| Asian | 2 | 391 | -3.91 (-10.06, 2.23) | 0.21 |  | 2.15 | 0.14 | 53.40 | <0.001 |  | -5.83 | 94.75 | 0.10 |  |  |  |  |  |  |  |  |  |  |  |  |  |  |  |  |  |  |  |  |  |  |  |  |  |  |  |  |  |  |  |  |  |  |  |  |  |  |  |  |  |
| Caucasian | 2 | 629 | -0.08 (-2.50, 2.34) | 0.94 |  | 1.05 | 0.30 | 4.40 |  |  |  |  |  |  |  |  |  |  |  |  |  |  |  |  |  |  |  |  |  |  |  |  |  |  |  |  |  |  |  |  |  |  |  |  |  |  |  |  |  |  |  |  |  |  |
| Mix | 1 | 30 | -35.0 (-42.18, -27.81) | <0.001 |  | - | - | - |  |  |  |  |  |  |  |  |  |  |  |  |  |  |  |  |  |  |  |  |  |  |  |  |  |  |  |  |  |  |  |  |  |  |  |  |  |  |  |  |  |  |  |  |  |  |
| Quality score | | | | | | | | | | | | | |  |  |  |  |  |  |  |  |  |  |  |  |  |  |  |  |  |  |  |  |  |  |  |  |  |  |  |  |  |  |  |  |  |  |  |  |  |  |  |  |  |
| High quality | 3 | 934 | -1.74 (-5.64, 2.16) | 0.38 |  | 5.23 | 0.07 | 61.70 | <0.001 |  | -7.11 | 94.55 | 0.14 |  |  |  |  |  |  |  |  |  |  |  |  |  |  |  |  |  |  |  |  |  |  |  |  |  |  |  |  |  |  |  |  |  |  |  |  |  |  |  |  |  |
| Medium quality | 2 | 116 | -17.95 (-51.18, 15.27) | 0.29 |  | 55.74 | <0.001 | 98.20 |  |  |  |  |  |  |  |  |  |  |  |  |  |  |  |  |  |  |  |  |  |  |  |  |  |  |  |  |  |  |  |  |  |  |  |  |  |  |  |  |  |  |  |  |  |  |
| Design of study | | | | | | | | | | | | | |  | |  |  |  |  |  |  |  |  |  |  |  |  |  |  |  |  |  |  |  |  |  |  |  |  |  |  |  |  |  |  |  |  |  |  |  |  |  |  |  |
| Case-control | 3 | 476 | -12.29 (-30.19, 5.61) | 0.17 |  | 71.82 | <0.001 | 97.20 | 0.004 |  | -4.48 | 95.63 | 0.40 |  |  |  |  |  |  |  |  |  |  |  |  |  |  |  |  |  |  |  |  |  |  |  |  |  |  |  |  |  |  |  |  |  |  |  |  |  |  |  |  |  |
| Cross-sectional | 2 | 574 | -2.67 (-10.84, 5.49) | 0.52 |  | 5.06 | 0.02 | 80.30 |  |  |  |  |  |  |  |  |  |  |  |  |  |  |  |  |  |  |  |  |  |  |  |  |  |  |  |  |  |  |  |  |  |  |  |  |  |  |  |  |  |  |  |  |  |  |
| Adjustment of confounders | | | | | | | | | | | | | |  | | | |  | | | | |  |  |  |  |  |  |  |  |  |  |  |  |  |  |  |  |  |  |  |  |  |  |  |  |  |  |  |  |  |  |  |  |
| Adjusted | 2 | 629 | -0.08 (-2.50, 2.34) | 0.94 |  | 1.05 | 0.30 | 4.40 | <0.001 |  | -6.13 | 94.46 | 0.16 |  |  |  |  |  |  |  |  |  |  |  |  |  |  |  |  |  |  |  |  |  |  |  |  |  |  |  |  |  |  |  |  |  |  |  |  |  |  |  |  |  |
| Unadjusted | 3 | 421 | -14.38 (-33.93, 5.16) | 0.14 |  | 57.63 | <0.001 | 96.50 |  |  |  |  |  |  |  |  |  |  |  |  |  |  |  |  |  |  |  |  |  |  |  |  |  |  |  |  |  |  |  |  |  |  |  |  |  |  |  |  |  |  |  |  |  |  |
| **Heterozygous model (GA vs. GG)** | | | | | | | | | | | | | |  |  |  |  |  |  |  |  |  |  |  |  |  |  |  |  |  |  |  |  |  |  |  |  |  |  |  |  |  |  |  |  |  |  |  |  |  |  |  |  |  |
| Ethnicity | | | | | | | | | | | | | |  |  |  |  |  |  |  |  |  |  |  |  |  |  |  |  |  |  |  |  |  |  |  |  |  |  |  |  |  |  |  |  |  |  |  |  |  |  |  |  |  |
| Asian | 2 | 672 | 0.96 (-1.73, 3.67) | 0.48 |  | 0.12 | 0.73 | 0.00 | <0.001 |  | -3.00 | 82.48 | 0.12 |  |  |  |  |  |  |  |  |  |  |  |  |  |  |  |  |  |  |  |  |  |  |  |  |  |  |  |  |  |  |  |  |  |  |  |  |  |  |  |  |  |
| Caucasian | 2 | 922 | -1.65 (-3.73, 0.43) | 0.12 |  | 0.14 | 0.70 | 0.00 |  |  |  |  |  |  |  |  |  |  |  |  |  |  |  |  |  |  |  |  |  |  |  |  |  |  |  |  |  |  |  |  |  |  |  |  |  |  |  |  |  |  |  |  |  |  |
| Mix | 1 | 75 | -20.00 (-27.20, -12.79) | **<0.001** |  | - | - | - |  |  |  |  |  |  |  |  |  |  |  |  |  |  |  |  |  |  |  |  |  |  |  |  |  |  |  |  |  |  |  |  |  |  |  |  |  |  |  |  |  |  |  |  |  |  |
| Quality score | | | | | | | | | | | | | |  |  |  |  |  |  |  |  |  |  |  |  |  |  |  |  |  |  |  |  |  |  |  |  |  |  |  |  |  |  |  |  |  |  |  |  |  |  |  |  |  |
| High quality | 3 | 1380 | -1.15 (-2.98, 0.67) | 0.21 |  | 1.09 | 0.58 | 0.00 | 0.27 |  | -3.23 | 85.58 | 0.24 |  |  |  |  |  |  |  |  |  |  |  |  |  |  |  |  |  |  |  |  |  |  |  |  |  |  |  |  |  |  |  |  |  |  |  |  |  |  |  |  |  |
| Medium quality | 2 | 289 | -9.05 (-30.06, 11.95) | 0.39 |  | 26.49 | <0.001 | 96.20 |  |  |  |  |  |  |  |  |  |  |  |  |  |  |  |  |  |  |  |  |  |  |  |  |  |  |  |  |  |  |  |  |  |  |  |  |  |  |  |  |  |  |  |  |  |  |
| Design of study | | | | | | | | | | | | | |  | | |  | | | |  | | | |  | | |  | | |  | | | |  | | |  | | |  | | |  | | |  | | |  | | |  | |
| Case-control | 3 | 823 | -5.95 (-15.28, 3.37) | 0.21 |  | 27.29 | <0.001 | 92.70 | 0.52 |  | -1.86 | 86.62 | 0.52 |  |  |  |  |  |  |  |  |  |  |  |  |  |  |  |  |  |  |  |  |  |  |  |  |  |  |  |  |  |  |  |  |  |  |  |  |  |  |  |  |  |
| Cross-sectional | 2 | 843 | -1.10(-3.45, 1.24) | 0.35 |  | 1.09 | 0.29 | 7.80 |  |  |  |  |  |  |  |  |  |  |  |  |  |  |  |  |  |  |  |  |  |  |  |  |  |  |  |  |  |  |  |  |  |  |  |  |  |  |  |  |  |  |  |  |  |  |
| Adjustment of confounders | | | | | | | | | | | | | |  | | | |  | | | | |  |  |  |  |  |  |  |  |  |  |  |  |  |  |  |  |  |  |  |  |  |  |  |  |  |  |  |  |  |  |  |  |
| Adjusted | 2 | 922 | -1.65 (-3.73, 0.43) | 0.12 |  | 0.14 | 0.70 | 93.00 | 0.98 |  | -2.51 | 86.32 | 0.32 |  |  |  |  |  |  |  |  |  |  |  |  |  |  |  |  |  |  |  |  |  |  |  |  |  |  |  |  |  |  |  |  |  |  |  |  |  |  |  |  |  |
| Unadjusted | 3 | 747 | -5.48 (-15.65, 4.69) | 0.29 |  | 28.65 | <0.001 | 0.00 |  |  |  |  |  |  |  |  |  |  |  |  |  |  |  |  |  |  |  |  |  |  |  |  |  |  |  |  |  |  |  |  |  |  |  |  |  |  |  |  |  |  |  |  |  |  |
| **Diastolic Blood pressure (mmHg)** | | | | | | | | | | | | | |  |  | | | |  | | |  | | | |  | | |  | | |  | |  | |  | | |  | | |  | | |  | | |  | | |  |  |  |  |
| **Allelic model (A vs. G)** | | | | | | | | | | | | | |  |  |  |  |  |  |  |  |  |  |  |  |  |  |  |  |  |  |  |  |  |  |  |  |  |  |  |  |  |  |  |  |  |  |  |  |  |  |  |  |  |
| Ethnicity | | | | | | | | | | | | | |  |  |  |  |  |  |  |  |  |  |  |  |  |  |  |  |  |  |  |  |  |  |  |  |  |  |  |  |  |  |  |  |  |  |  |  |  |  |  |  |  |
| Asian | 3 | 1692 | -0.60 (-1.81, 0.60) | 0.32 |  | 1.16 | 0.55 | 0.00 | 0.29 |  | -0.08 | 0.00 | 0.66 |  |  |  |  |  |  |  |  |  |  |  |  |  |  |  |  |  |  |  |  |  |  |  |  |  |  |  |  |  |  |  |  |  |  |  |  |  |  |  |  |  |
| Caucasian | 2 | 2556 | 0.11 (-0.70, 0.92) | 0.79 |  | 1.30 | 0.25 | 22.90 |  |  |  |  |  |  |  |  |  |  |  |  |  |  |  |  |  |  |  |  |  |  |  |  |  |  |  |  |  |  |  |  |  |  |  |  |  |  |  |  |  |  |  |  |  |  |
| Mix | 1 | 200 | -2.08 (-5.31, 1.14) | 0.20 |  | - | - | - |  |  |  |  |  |  |  |  |  |  |  |  |  |  |  |  |  |  |  |  |  |  |  |  |  |  |  |  |  |  |  |  |  |  |  |  |  |  |  |  |  |  |  |  |  |  |
| Quality score | | | | | | | | | | | | | |  |  |  |  |  |  |  |  |  |  |  |  |  |  |  |  |  |  |  |  |  |  |  |  |  |  |  |  |  |  |  |  |  |  |  |  |  |  |  |  |  |
| High quality | 3 | 3546 | -0.16 (-1.10, 0.76) | 0.72 |  | 3.45 | 0.17 | 42.10 | 0.64 |  | -0.15 | 0.00 | 0.56 |  |  |  |  |  |  |  |  |  |  |  |  |  |  |  |  |  |  |  |  |  |  |  |  |  |  |  |  |  |  |  |  |  |  |  |  |  |  |  |  |  |
| Medium quality | 3 | 902 | -0.44 (-1.85, 0.96) | 0.53 |  | 1.22 | 0.54 | 0.00 |  |  |  |  |  |  |  |  |  |  |  |  |  |  |  |  |  |  |  |  |  |  |  |  |  |  |  |  |  |  |  |  |  |  |  |  |  |  |  |  |  |  |  |  |  |  |
| Design of study | | | | | | | | | | | | | |  |  |  |  |  |  |  |  |  |  |  |  |  |  |  |  |  |  |  |  |  |  |  |  |  |  |  |  |  |  |  |  |  |  |  |  |  |  |  |  |  |
| Case-control | 4 | 2374 | -0.35 (-1.17, 0.47) | 0.40 |  | 1.25 | 0.74 | 0.00 | 0.46 |  | -0.07 | 1.31 | 0.75 |  |  |  |  |  |  |  |  |  |  |  |  |  |  |  |  |  |  |  |  |  |  |  |  |  |  |  |  |  |  |  |  |  |  |  |  |  |  |  |  |  |
| Cross-sectional | 2 | 2074 | -0.27 (-2.15, 1.60) | 0.77 |  | 3.11 | 0.07 | 67.90 |  |  |  |  |  |  |  |  |  |  |  |  |  |  |  |  |  |  |  |  |  |  |  |  |  |  |  |  |  |  |  |  |  |  |  |  |  |  |  |  |  |  |  |  |  |  |
| Adjustment of confounders | | | | | | | | | | | | | |  |  |  |  |  |  |  |  |  |  |  |  |  |  |  |  |  |  |  |  |  |  |  |  |  |  |  |  |  |  |  |  |  |  |  |  |  |  |  |  |  |
| Adjusted | 2 | 2556 | 0.11 (-0.70, 0.92) | 0.79 |  | 1.30 | 0.25 | 22.90 | 0.18 |  | -0.2 | 0.00 | 0.40 |  |  |  |  |  |  |  |  |  |  |  |  |  |  |  |  |  |  |  |  |  |  |  |  |  |  |  |  |  |  |  |  |  |  |  |  |  |  |  |  |  |
| Unadjusted | 4 | 1892 | -0.78 (-1.92, 0.34) | 0.17 |  | 1.86 | 0.60 | 0.00 |  |  |  |  |  |  |  |  |  |  |  |  |  |  |  |  |  |  |  |  |  |  |  |  |  |  |  |  |  |  |  |  |  |  |  |  |  |  |  |  |  |  |  |  |  |  |
| **Dominant model (AA+GA vs. GG)** | | | | | | | | | | | | | |  |  |  |  |  |  |  |  |  |  |  |  |  |  |  |  |  |  |  |  |  |  |  |  |  |  |  |  |  |  |  |  |  |  |  |  |  |  |  |  |  |
| Ethnicity | | | | | | | | | | | | | |  |  |  |  |  |  |  |  |  |  |  |  |  |  |  |  |  |  |  |  |  |  |  |  |  |  |  |  |  |  |  |  |  |  |  |  |  |  |  |  |  |
| Asian | 3 | 846 | -0.39 (-2.22, 1.42) | 0.66 |  | 0.50 | 0.78 | 0.00 | 0.92 |  | -0.05 | 0.00 | 0.87 |  |  |  |  |  |  |  |  |  |  |  |  |  |  |  |  |  |  |  |  |  |  |  |  |  |  |  |  |  |  |  |  |  |  |  |  |  |  |  |  |  |
| Caucasian | 2 | 1278 | 0.005 (-1.30, 1.31) | 0.99 |  | 0.22 | 0.63 | 0.00 |  |  |  |  |  |  |  |  |  |  |  |  |  |  |  |  |  |  |  |  |  |  |  |  |  |  |  |  |  |  |  |  |  |  |  |  |  |  |  |  |  |  |  |  |  |  |
| Mix | 1 | 100 | -1.31 (-14.67, 12.03) | 0.84 |  | - | - | - |  |  |  |  |  |  |  |  |  |  |  |  |  |  |  |  |  |  |  |  |  |  |  |  |  |  |  |  |  |  |  |  |  |  |  |  |  |  |  |  |  |  |  |  |  |  |
| Quality score | | | | | | | | | | | | | |  |  |  |  |  |  |  |  |  |  |  |  |  |  |  |  |  |  |  |  |  |  |  |  |  |  |  |  |  |  |  |  |  |  |  |  |  |  |  |  |  |
| High quality | 3 | 1773 | -0.19 (-1.35, 0.95) | 0.73 |  | 0.62 | 0.73 | 0.00 | 0.80 |  | -0.07 | 0.00 | 0.86 |  |  |  |  |  |  |  |  |  |  |  |  |  |  |  |  |  |  |  |  |  |  |  |  |  |  |  |  |  |  |  |  |  |  |  |  |  |  |  |  |  |
| Medium quality | 3 | 451 | 0.17 (-2.53, 2.88) | 0.90 |  | 0.19 | 0.90 | 0.00 |  |  |  |  |  |  |  |  |  |  |  |  |  |  |  |  |  |  |  |  |  |  |  |  |  |  |  |  |  |  |  |  |  |  |  |  |  |  |  |  |  |  |  |  |  |  |
| Design of study | | | | | | | | | | | | | |  | |  | | | |  | | | |  | | |  | | |  | | |  | | | |  | | |  | | |  | | |  | | |  | | |  | |  |
| Case-control | 4 | 1187 | -0.14 (-1.61, 1.33) | 0.85 |  | 0.27 | 0.96 | 0.00 | 0.99 |  | -0.08 | 0.00 | 0.81 |  |  |  |  |  |  |  |  |  |  |  |  |  |  |  |  |  |  |  |  |  |  |  |  |  |  |  |  |  |  |  |  |  |  |  |  |  |  |  |  |  |
| Cross-sectional | 2 | 1037 | -0.13 (-1.67, 1.39) | 0.86 |  | 0.61 | 0.43 | 0.00 |  |  |  |  |  |  |  |  |  |  |  |  |  |  |  |  |  |  |  |  |  |  |  |  |  |  |  |  |  |  |  |  |  |  |  |  |  |  |  |  |  |  |  |  |  |  |
| Adjustment of confounders | | | | | | | | | | | | | |  | | | |  | | | | |  |  |  |  |  |  |  |  |  |  |  |  |  |  |  |  |  |  |  |  |  |  |  |  |  |  |  |  |  |  |  |  |
| Adjusted | 2 | 1278 | 0.005 (-1.30, 1.31) | 0.99 |  | 0.22 | 0.63 | 0.00 | 0.71 |  | -0.13 | 0.00 | 0.72 |  |  |  |  |  |  |  |  |  |  |  |  |  |  |  |  |  |  |  |  |  |  |  |  |  |  |  |  |  |  |  |  |  |  |  |  |  |  |  |  |  |
| Unadjusted | 4 | 946 | -0.41 (-2.22, 1.39) | 0.65 |  | 0.52 | 0.91 | 0.00 |  |  |  |  |  |  |  |  |  |  |  |  |  |  |  |  |  |  |  |  |  |  |  |  |  |  |  |  |  |  |  |  |  |  |  |  |  |  |  |  |  |  |  |  |  |  |
| **Recessive model (AA vs. GA + GG)** | | | | | | | | | | | | | |  |  |  |  |  |  |  |  |  |  |  |  |  |  |  |  |  |  |  |  |  |  |  |  |  |  |  |  |  |  |  |  |  |  |  |  |  |  |  |  |  |
| Ethnicity | | | | | | | | | | | | | |  |  |  |  |  |  |  |  |  |  |  |  |  |  |  |  |  |  |  |  |  |  |  |  |  |  |  |  |  |  |  |  |  |  |  |  |  |  |  |  |  |
| Asian | 3 | 846 | -2.23 (-5.13, 0.65) | 0.13 |  | 2.39 | 0.30 | 16.40 | 0.07 |  | -0.47 | 48.47 | 0.41 |  |  |  |  |  |  |  |  |  |  |  |  |  |  |  |  |  |  |  |  |  |  |  |  |  |  |  |  |  |  |  |  |  |  |  |  |  |  |  |  |  |
| Caucasian | 2 | 1278 | 0.24 (-1.25, 1.74) | 0.75 |  | 1.90 | 0.16 | 47.20 |  |  |  |  |  |  |  |  |  |  |  |  |  |  |  |  |  |  |  |  |  |  |  |  |  |  |  |  |  |  |  |  |  |  |  |  |  |  |  |  |  |  |  |  |  |  |
| Mix | 1 | 100 | -5.00 (-11.32, 1.32) | 0.12 |  | - | - | - |  |  |  |  |  |  |  |  |  |  |  |  |  |  |  |  |  |  |  |  |  |  |  |  |  |  |  |  |  |  |  |  |  |  |  |  |  |  |  |  |  |  |  |  |  |  |
| Quality score | | | | | | | | | | | | | |  |  |  |  |  |  |  |  |  |  |  |  |  |  |  |  |  |  |  |  |  |  |  |  |  |  |  |  |  |  |  |  |  |  |  |  |  |  |  |  |  |
| High quality | 3 | 1773 | -0.65 (-2.90, 1.58) | 0.56 |  | 6.89 | 0.03 | 71.00 | 0.30 |  | -0.70 | 45.79 | 0.33 |  |  |  |  |  |  |  |  |  |  |  |  |  |  |  |  |  |  |  |  |  |  |  |  |  |  |  |  |  |  |  |  |  |  |  |  |  |  |  |  |  |
| Medium quality | 3 | 451 | -1.68 (-4.59, 1.21) | 0.25 |  | 1.59 | 0.45 | 0.00 |  |  |  |  |  |  |  |  |  |  |  |  |  |  |  |  |  |  |  |  |  |  |  |  |  |  |  |  |  |  |  |  |  |  |  |  |  |  |  |  |  |  |  |  |  |  |
| Design of study | | | | | | | | | | | | | |  | |  | | | |  | | | |  | | |  | | |  | | |  | | | |  | | |  | | |  | | |  | | |  | | |  | |  |
| Case-control | 4 | 1187 | -0.78 (-2.15, 0.58) | 0.26 |  | 2.06 | 0.55 | 0.00 | 0.26 |  | -0.58 | 48.87 | 0.41 |  |  |  |  |  |  |  |  |  |  |  |  |  |  |  |  |  |  |  |  |  |  |  |  |  |  |  |  |  |  |  |  |  |  |  |  |  |  |  |  |  |
| Cross-sectional | 2 | 1037 | -1.56 (-7.25, 4.13) | 0.59 |  | 6.22 | 0.01 | 83.90 |  |  |  |  |  |  |  |  |  |  |  |  |  |  |  |  |  |  |  |  |  |  |  |  |  |  |  |  |  |  |  |  |  |  |  |  |  |  |  |  |  |  |  |  |  |  |
| Adjustment of confounders | | | | | | | | | | | | | |  | | | |  | | | | |  |  |  |  |  |  |  |  |  |  |  |  |  |  |  |  |  |  |  |  |  |  |  |  |  |  |  |  |  |  |  |  |
| Adjusted | 2 | 1278 | 0.24 (-1.25, 1.74) | 0.75 |  | 1.90 | 0.16 | 47.20 | 0.03 |  | -0.82 | 41.41 | 0.20 |  |  |  |  |  |  |  |  |  |  |  |  |  |  |  |  |  |  |  |  |  |  |  |  |  |  |  |  |  |  |  |  |  |  |  |  |  |  |  |  |  |
| Unadjusted | 4 | 946 | -2.66 (-5.08, -0.25) | **0.03** |  | 3.00 | 0.39 | 0.10 |  |  |  |  |  |  |  |  |  |  |  |  |  |  |  |  |  |  |  |  |  |  |  |  |  |  |  |  |  |  |  |  |  |  |  |  |  |  |  |  |  |  |  |  |  |  |
| **Homozygous model (AA vs. GG)** | | | | | | | | | | | | | |  |  |  |  |  |  |  |  |  |  |  |  |  |  |  |  |  |  |  |  |  |  |  |  |  |  |  |  |  |  |  |  |  |  |  |  |  |  |  |  |  |
| Ethnicity | | | | | | | | | | | | | |  |  |  |  |  |  |  |  |  |  |  |  |  |  |  |  |  |  |  |  |  |  |  |  |  |  |  |  |  |  |  |  |  |  |  |  |  |  |  |  |  |
| Asian | 3 | 454 | -2.40 (-5.42, 0.62) | 0.12 |  | 2.10 | 0.35 | 4.80 | 0.26 |  | -0.23 | 19.00 | 0.66 |  |  |  |  |  |  |  |  |  |  |  |  |  |  |  |  |  |  |  |  |  |  |  |  |  |  |  |  |  |  |  |  |  |  |  |  |  |  |  |  |  |
| Caucasian | 2 | 629 | 0.13 (-1.43, 1.69) | 0.86 |  | 1.09 | 0.29 | 8.00 |  |  |  |  |  |  |  |  |  |  |  |  |  |  |  |  |  |  |  |  |  |  |  |  |  |  |  |  |  |  |  |  |  |  |  |  |  |  |  |  |  |  |  |  |  |  |
| Mix | 1 | 30 | -5.00 (-19.40, 9.40) | 0.49 |  | - | - | - |  |  |  |  |  |  |  |  |  |  |  |  |  |  |  |  |  |  |  |  |  |  |  |  |  |  |  |  |  |  |  |  |  |  |  |  |  |  |  |  |  |  |  |  |  |  |
| Quality score | | | | | | | | | | | | | |  |  |  |  |  |  |  |  |  |  |  |  |  |  |  |  |  |  |  |  |  |  |  |  |  |  |  |  |  |  |  |  |  |  |  |  |  |  |  |  |  |
| High quality | 3 | 934 | -0.85 (-3.41, 1.69) | 0.51 |  | 5.44 | 0.06 | 63.20 | 0.81 |  | -0.47 | 14.06 | 0.50 |  |  |  |  |  |  |  |  |  |  |  |  |  |  |  |  |  |  |  |  |  |  |  |  |  |  |  |  |  |  |  |  |  |  |  |  |  |  |  |  |  |
| Medium quality | 3 | 179 | -0.85 (-4.60, 2.90) | 0.65 |  | 0.37 | 0.80 | 0.00 |  |  |  |  |  |  |  |  |  |  |  |  |  |  |  |  |  |  |  |  |  |  |  |  |  |  |  |  |  |  |  |  |  |  |  |  |  |  |  |  |  |  |  |  |  |  |
| Design of study | | | | | | | | | | | | | |  | |  |  |  |  |  |  |  |  |  |  |  |  |  |  |  |  |  |  |  |  |  |  |  |  |  |  |  |  |  |  |  |  |  |  |  |  |  |  |  |
| Case-control | 4 | 539 | -0.65 (-2.44, 1.12) | 0.47 |  | 0.39 | 0.94 | 0.00 | 0.71 |  | -0.44 | 17.26 | 0.55 |  |  |  |  |  |  |  |  |  |  |  |  |  |  |  |  |  |  |  |  |  |  |  |  |  |  |  |  |  |  |  |  |  |  |  |  |  |  |  |  |  |
| Cross-sectional | 2 | 574 | -1.61 (-7.36, 4.12) | 0.58 |  | 5.35 | 0.02 | 81.30 |  |  |  |  |  |  |  |  |  |  |  |  |  |  |  |  |  |  |  |  |  |  |  |  |  |  |  |  |  |  |  |  |  |  |  |  |  |  |  |  |  |  |  |  |  |  |
| Adjustment of confounders | | | | | | | | | | | | | |  | | | |  | | | | |  |  |  |  |  |  |  |  |  |  |  |  |  |  |  |  |  |  |  |  |  |  |  |  |  |  |  |  |  |  |  |  |
| Adjusted | 2 | 629 | 0.13 (-1.43, 1.69) | 0.86 |  | 1.09 | 0.29 | 8.00 | 0.10 |  | -0.67 | 0.46 | 0.30 |  |  |  |  |  |  |  |  |  |  |  |  |  |  |  |  |  |  |  |  |  |  |  |  |  |  |  |  |  |  |  |  |  |  |  |  |  |  |  |  |  |
| Unadjusted | 4 | 484 | -2.52 (-5.40, 0.35) | 0.08 |  | 2.22 | 0.52 | 0.00 |  |  |  |  |  |  |  |  |  |  |  |  |  |  |  |  |  |  |  |  |  |  |  |  |  |  |  |  |  |  |  |  |  |  |  |  |  |  |  |  |  |  |  |  |  |  |
| **Heterozygous model (GA vs. GG)** | | | | | | | | | | | | | |  |  |  |  |  |  |  |  |  |  |  |  |  |  |  |  |  |  |  |  |  |  |  |  |  |  |  |  |  |  |  |  |  |  |  |  |  |  |  |  |  |
| Ethnicity | | | | | | | | | | | | | |  |  |  |  |  |  |  |  |  |  |  |  |  |  |  |  |  |  |  |  |  |  |  |  |  |  |  |  |  |  |  |  |  |  |  |  |  |  |  |  |  |
| Asian | 3 | 762 | 0.10 (-1.79, 2.00) | 0.91 |  | 0.31 | 0.85 | 0.00 | 0.99 |  | -0.01 | 0.00 | 0.97 |  |  |  |  |  |  |  |  |  |  |  |  |  |  |  |  |  |  |  |  |  |  |  |  |  |  |  |  |  |  |  |  |  |  |  |  |  |  |  |  |  |
| Caucasian | 2 | 922 | -0.05 (-1.43, 1.32) | 0.93 |  | 0.00 | 0.94 | 0.00 |  |  |  |  |  |  |  |  |  |  |  |  |  |  |  |  |  |  |  |  |  |  |  |  |  |  |  |  |  |  |  |  |  |  |  |  |  |  |  |  |  |  |  |  |  |  |
| Mix | 1 | 75 | 0.00 (-13.35, 13.35) | 1.00 |  | - | - | - |  |  |  |  |  |  |  |  |  |  |  |  |  |  |  |  |  |  |  |  |  |  |  |  |  |  |  |  |  |  |  |  |  |  |  |  |  |  |  |  |  |  |  |  |  |  |
| Quality score | | | | | | | | | | | | | |  |  |  |  |  |  |  |  |  |  |  |  |  |  |  |  |  |  |  |  |  |  |  |  |  |  |  |  |  |  |  |  |  |  |  |  |  |  |  |  |  |
| High quality | 3 | 1380 | -0.06 (-1.27, 1.14) | 0.91 |  | 0.01 | 0.99 | 0.00 | 0.78 |  | 0.03 | 0.00 | 0.93 |  |  |  |  |  |  |  |  |  |  |  |  |  |  |  |  |  |  |  |  |  |  |  |  |  |  |  |  |  |  |  |  |  |  |  |  |  |  |  |  |  |
| Medium quality | 3 | 379 | 0.35 (-2.43, 3.13) | 0.80 |  | 0.26 | 0.88 | 0.00 |  |  |  |  |  |  |  |  |  |  |  |  |  |  |  |  |  |  |  |  |  |  |  |  |  |  |  |  |  |  |  |  |  |  |  |  |  |  |  |  |  |  |  |  |  |  |
| Design of study | | | | | | | | | | | | | |  | | |  | | | |  | | | |  | | |  | | |  | | | |  | | |  | | |  | | |  | | |  | | |  | | |  | |
| Case-control | 4 | 913 | 0.03 (-1.51, 1.58) | 0.96 |  | 0.33 | 0.95 | 0.00 | 0.94 |  | -0.007 | 0.00 | 0.98 |  |  |  |  |  |  |  |  |  |  |  |  |  |  |  |  |  |  |  |  |  |  |  |  |  |  |  |  |  |  |  |  |  |  |  |  |  |  |  |  |  |
| Cross-sectional | 2 | 846 | -0.04 (-1.63, 1.56) | 0.96 |  | 0.00 | 0.95 | 0.00 |  |  |  |  |  |  |  |  |  |  |  |  |  |  |  |  |  |  |  |  |  |  |  |  |  |  |  |  |  |  |  |  |  |  |  |  |  |  |  |  |  |  |  |  |  |  |
| Adjustment of confounders | | | | | | | | | | | | | |  | | | |  | | | | |  |  |  |  |  |  |  |  |  |  |  |  |  |  |  |  |  |  |  |  |  |  |  |  |  |  |  |  |  |  |  |  |
| Adjusted | 2 | 922 | -0.05 (-1.43, 1.32) | 0.93 |  | 0.00 | 0.94 | 0.00 | 0.89 |  | 0.01 | 0.00 | 0.96 |  |  |  |  |  |  |  |  |  |  |  |  |  |  |  |  |  |  |  |  |  |  |  |  |  |  |  |  |  |  |  |  |  |  |  |  |  |  |  |  |  |
| Unadjusted | 4 | 837 | 0.10 (-1.77, 1.98) | 0.91 |  | 0.31 | 0.95 | 0.00 |  |  |  |  |  |  |  |  |  |  |  |  |  |  |  |  |  |  |  |  |  |  |  |  |  |  |  |  |  |  |  |  |  |  |  |  |  |  |  |  |  |  |  |  |  |  |

^a^ All analysis was done using random effects model. WMD, weighted mean difference; 95% CI, 95% confidence interval; NM, not mention.

**Supplementary Table S9-**The association between *CD36* rs1761667 polymorphism and fasting blood glucose based on several subgroups. All analyses were conducted using a random-effects model^a^

|  | **No. of data-sets** | **No. of subjects** | **Meta-analysis** | |  | **Heterogeneity** | | | |  | **Meta-regression** | | |  |  |  |  |  |  |  |  |  |  |  |  |  |  |  |  |  |  |  |  |  |  |  |  |  |  |  |  |
| --- | --- | --- | --- | --- | --- | --- | --- | --- | --- | --- | --- | --- | --- | --- | --- | --- | --- | --- | --- | --- | --- | --- | --- | --- | --- | --- | --- | --- | --- | --- | --- | --- | --- | --- | --- | --- | --- | --- | --- | --- | --- |
|  |  |  | **WMD^2^ (95%CI)** | **P _effect_** |  | ***Q* statistic** | **P _within_** | **I^2^ (%)** | **P _between group_** |  | **β** | **I^2^_residual (%)_** | **P-value** |  |  |  |  |  |  |  |  |  |  |  |  |  |  |  |  |  |  |  |  |  |  |  |  |  |  |  |  |
| **Fasting blood glucose (mg/dl)** | | | | | | | | | | | | | |  |  |  |  |  |  |  |  |  |  |  |  |  |  |  |  |  |  |  |  |  |  |  |  |  |  |  |  |
| **Allelic model (A vs. G)** | | | | | | | | | | | | | |  |  |  |  |  |  |  |  |  |  |  |  |  |  |  |  |  |  |  |  |  |  |  |  |  |  |  |  |
| Ethnicity | | | | | | | | | | | | | |  |  |  |  |  |  |  |  |  |  |  |  |  |  |  |  |  |  |  |  |  |  |  |  |  |  |  |  |
| Asian | 3 | 1692 | 0.36 (-1.31, 2.04) | 0.66 |  | 1.66 | 0.43 | 0.00 | 0.17 |  | 1.41 | 71.38 | 0.06 |  |  |  |  |  |  |  |  |  |  |  |  |  |  |  |  |  |  |  |  |  |  |  |  |  |  |  |  |
| Caucasian | 3 | 2772 | 3.26 (-0.46, 7.00) | 0.08 |  | 18.62 | <0.001 | 89.30 |  |  |  |  |  |  |  |  |  |  |  |  |  |  |  |  |  |  |  |  |  |  |  |  |  |  |  |  |  |  |  |  |  |
| Mix | 1 | 146 | 4.76 (-9.56, 19.10) | 0.51 |  | - | - | - |  |  |  |  |  |  |  |  |  |  |  |  |  |  |  |  |  |  |  |  |  |  |  |  |  |  |  |  |  |  |  |  |  |
| Participants’ health status | | | | | | | | | | | | | |  |  |  |  |  |  |  |  |  |  |  |  |  |  |  |  |  |  |  |  |  |  |  |  |  |  |  |  |
| Heart disease | 1 | 478 | -1.12 (-3.98, 1.72) | 0.43 |  | - | - | - | 0.003 |  | 1.05 | 65.07 | **0.04** |  |  |  |  |  |  |  |  |  |  |  |  |  |  |  |  |  |  |  |  |  |  |  |  |  |  |  |  |
| Healthy | 2 | 1300 | 1.16 (0.13, 2.20) | **0.02** |  | 0.51 | 0.47 | 0.00 |  |  |  |  |  |  |  |  |  |  |  |  |  |  |  |  |  |  |  |  |  |  |  |  |  |  |  |  |  |  |  |  |  |
| Others | 4 | 2832 | 3.79 (-0.48, 8.07) | 0.08 |  | 11.64 | 0.009 | 74.20 |  |  |  |  |  |  |  |  |  |  |  |  |  |  |  |  |  |  |  |  |  |  |  |  |  |  |  |  |  |  |  |  |  |
| Hardy-Weinberg equilibrium | | | | | | | | | | | | | |  |  |  |  |  |  |  |  |  |  |  |  |  |  |  |  |  |  |  |  |  |  |  |  |  |  |  |  |
| Equilibrium | 5 | 3916 | 2.19 (-0.45, 4.85) | 0.10 |  | 21.37 | 0.001 | 76.60 | 0.30 |  | 0.91 | 74.50 | 0.26 |  |  |  |  |  |  |  |  |  |  |  |  |  |  |  |  |  |  |  |  |  |  |  |  |  |  |  |  |
| Disequilibrium | 1 | 478 | -0.79 (-4.17, 2.58) | 0.64 |  | - | - | - |  |  |  |  |  |  |  |  |  |  |  |  |  |  |  |  |  |  |  |  |  |  |  |  |  |  |  |  |  |  |  |  |  |
| NM | 1 | 216 | 2.48 (-1.25, 6.21) | 0.19 |  | - | - | - |  |  |  |  |  |  |  |  |  |  |  |  |  |  |  |  |  |  |  |  |  |  |  |  |  |  |  |  |  |  |  |  |  |
| Quality score | | | | | | | | | | | | | |  |  |  |  |  |  |  |  |  |  |  |  |  |  |  |  |  |  |  |  |  |  |  |  |  |  |  |  |
| High quality | 4 | 3692 | 2.83 (-0.14, 5.81) | 0.06 |  | 19.45 | <0.001 | 84.60 | 0.16 |  | 1.13 | 78.74 | 0.26 |  |  |  |  |  |  |  |  |  |  |  |  |  |  |  |  |  |  |  |  |  |  |  |  |  |  |  |  |
| Medium quality | 3 | 918 | 0.37 (-2.28, 3.03) | 0.78 |  | 2.36 | 0.30 | 15.40 |  |  |  |  |  |  |  |  |  |  |  |  |  |  |  |  |  |  |  |  |  |  |  |  |  |  |  |  |  |  |  |  |  |
| Design of study | | | | | | | | | | | | | |  |  |  |  |  |  |  |  |  |  |  |  |  |  |  |  |  |  |  |  |  |  |  |  |  |  |  |  |
| Case-control | 3 | 2174 | 2.74 (-3.97, 9.46) | 0.42 |  | 16.76 | <0.001 | 88.10 | 0.01 |  | 1.08 | 79.35 | 0.20 |  |  |  |  |  |  |  |  |  |  |  |  |  |  |  |  |  |  |  |  |  |  |  |  |  |  |  |  |
| Cross-sectional | 4 | 2436 | 1.17 (0.25, 2.10) | **0.01** |  | 0.76 | 0.86 | 0.00 |  |  |  |  |  |  |  |  |  |  |  |  |  |  |  |  |  |  |  |  |  |  |  |  |  |  |  |  |  |  |  |  |  |
| Adjustment of confounders | | | | | | | | | | | | | |  |  |  |  |  |  |  |  |  |  |  |  |  |  |  |  |  |  |  |  |  |  |  |  |  |  |  |  |
| Adjusted | 3 | 2702 | 3.70 (-1.04, 8.45) | 0.12 |  | 18.71 | <0.001 | 89.30 | 0.12 |  | 0.96 | 79.63 | 0.28 |  |  |  |  |  |  |  |  |  |  |  |  |  |  |  |  |  |  |  |  |  |  |  |  |  |  |  |  |
| Unadjusted | 4 | 1908 | 0.72 (-0.80, 2.25) | 0.35 |  | 2.69 | 0.44 | 0.00 |  |  |  |  |  |  |  |  |  |  |  |  |  |  |  |  |  |  |  |  |  |  |  |  |  |  |  |  |  |  |  |  |  |
| **Dominant model (AA+GA vs. GG)** | | | | | | | | | | | | | |  |  |  |  |  |  |  |  |  |  |  |  |  |  |  |  |  |  |  |  |  |  |  |  |  |  |  |  |
| Ethnicity | | | | | | | | | | | | | |  |  |  |  |  |  |  |  |  |  |  |  |  |  |  |  |  |  |  |  |  |  |  |  |  |  |  |  |
| Asian | 3 | 846 | 0.70 (-1.57, 2.98) | 0.54 |  | 1.16 | 0.55 | 0.00 | 0.04 |  | 2.44 | 86.65 | 0.07 |  |  |  |  |  |  |  |  |  |  |  |  |  |  |  |  |  |  |  |  |  |  |  |  |  |  |  |  |
| Caucasian | 3 | 1386 | 5.20 (-2.91, 13.33) | 0.20 |  | 42.36 | <0.001 | 95.30 |  |  |  |  |  |  |  |  |  |  |  |  |  |  |  |  |  |  |  |  |  |  |  |  |  |  |  |  |  |  |  |  |  |
| Mix | 1 | 73 | 10.29 (-6.58, 27.17) | 0.23 |  | - | - | - |  |  |  |  |  |  |  |  |  |  |  |  |  |  |  |  |  |  |  |  |  |  |  |  |  |  |  |  |  |  |  |  |  |
| Participants’ health status | | | | | | | | | | | | | |  |  |  |  |  |  |  |  |  |  |  |  |  |  |  |  |  |  |  |  |  |  |  |  |  |  |  |  |
| Heart disease | 1 | 239 | -1.27 (-5.69, 3.14) | 0.57 |  | - | - | - | <0.001 |  | 1.82 | 84.82 | 0.06 |  |  |  |  |  |  |  |  |  |  |  |  |  |  |  |  |  |  |  |  |  |  |  |  |  |  |  |  |
| Healthy | 2 | 650 | 0.91 (-0.85, 2.68) | 0.31 |  | 0.47 | 0.49 | 0.00 |  |  |  |  |  |  |  |  |  |  |  |  |  |  |  |  |  |  |  |  |  |  |  |  |  |  |  |  |  |  |  |  |  |
| Others | 4 | 1416 | 7.18 (-1.35, 15.73) | 0.09 |  | 28.37 | <0.001 | 89.40 |  |  |  |  |  |  |  |  |  |  |  |  |  |  |  |  |  |  |  |  |  |  |  |  |  |  |  |  |  |  |  |  |  |
| Hardy-Weinberg equilibrium | | | | | | | | | | | | | |  |  |  |  |  |  |  |  |  |  |  |  |  |  |  |  |  |  |  |  |  |  |  |  |  |  |  |  |
| Equilibrium | 5 | 2023 | 4.14 (-1.18, 9.47) | 0.12 |  | 47.18 | <0.001 | 89.40 | 0.32 |  | 1.49 | 87.29 | 0.30 |  |  |  |  |  |  |  |  |  |  |  |  |  |  |  |  |  |  |  |  |  |  |  |  |  |  |  |  |
| Disequilibrium | 1 | 174 | -1.52 (-7.75, 4.70) | 0.63 |  | - | - | - |  |  |  |  |  |  |  |  |  |  |  |  |  |  |  |  |  |  |  |  |  |  |  |  |  |  |  |  |  |  |  |  |  |
| NM | 1 | 108 | 2.86 (-2.99, 8.73) | 0.33 |  | - | - | - |  |  |  |  |  |  |  |  |  |  |  |  |  |  |  |  |  |  |  |  |  |  |  |  |  |  |  |  |  |  |  |  |  |
| Quality score | | | | | | | | | | | | | |  |  |  |  |  |  |  |  |  |  |  |  |  |  |  |  |  |  |  |  |  |  |  |  |  |  |  |  |
| High quality | 4 | 1846 | 5.14 (-1.10, 11.39) | 0.10 |  | 45.85 | <0.001 | 93.50 | 0.09 |  | 1.80 | 90.24 | 0.25 |  |  |  |  |  |  |  |  |  |  |  |  |  |  |  |  |  |  |  |  |  |  |  |  |  |  |  |  |
| Medium quality | 3 | 459 | 0.31 (-3.19, 3.82) | 0.86 |  | 1.40 | 0.49 | 0.00 |  |  |  |  |  |  |  |  |  |  |  |  |  |  |  |  |  |  |  |  |  |  |  |  |  |  |  |  |  |  |  |  |  |
| Design of study | | | | | | | | | | | | | |  |  | | |  | | |  | |  | |  | |  | |  | |  | |  | |  | |  | |  | |  |
| Case-control | 3 | 1087 | 5.56 (-6.15, 17.27) | 0.35 |  | 24.18 | <0.001 | 91.70 | <0.001 |  | 1.95 | 89.27 | 0.28 |  |  |  |  |  |  |  |  |  |  |  |  |  |  |  |  |  |  |  |  |  |  |  |  |  |  |  |  |
| Cross-sectional | 4 | 1218 | 1.12 (-0.34, 2.59) | 0.13 |  | 1.69 | 0.64 | 0.00 |  |  |  |  |  |  |  |  |  |  |  |  |  |  |  |  |  |  |  |  |  |  |  |  |  |  |  |  |  |  |  |  |  |
| Adjustment of confounders | | | | | | | | | | | | | |  | | |  | | |  |  |  |  |  |  |  |  |  |  |  |  |  |  |  |  |  |  |  |  |  |  |
| Adjusted | 3 | 1351 | 6.98 (-2.67, 16.64) | 0.15 |  | 42.71 | <0.001 | 95.30 | 0.01 |  | 1.63 | 89.85 | 0.31 |  |  |  |  |  |  |  |  |  |  |  |  |  |  |  |  |  |  |  |  |  |  |  |  |  |  |  |  |
| Unadjusted | 4 | 954 | 0.98 (-1.13, 3.11) | 0.36 |  | 1.62 | 0.65 | 0.00 |  |  |  |  |  |  |  |  |  |  |  |  |  |  |  |  |  |  |  |  |  |  |  |  |  |  |  |  |  |  |  |  |  |
| **Recessive model (AA vs. GA + GG)** | | | | | | | | | | | | | |  |  |  |  |  |  |  |  |  |  |  |  |  |  |  |  |  |  |  |  |  |  |  |  |  |  |  |  |
| Ethnicity | | | | | | | | | | | | | |  |  |  |  |  |  |  |  |  |  |  |  |  |  |  |  |  |  |  |  |  |  |  |  |  |  |  |  |
| Asian | 3 | 846 | 0.15 (-3.61, 3.92) | 0.93 |  | 1.22 | 0.54 | 0.00 | 0.47 |  | 1.46 | 0.00 | **0.04** |  |  |  |  |  |  |  |  |  |  |  |  |  |  |  |  |  |  |  |  |  |  |  |  |  |  |  |  |
| Caucasian | 3 | 1386 | 3.30 (0.66, 5.93) | **0.01** |  | 3.31 | 0.19 | 39.50 |  |  |  |  |  |  |  |  |  |  |  |  |  |  |  |  |  |  |  |  |  |  |  |  |  |  |  |  |  |  |  |  |  |
| Mix | 1 | 73 | 4.80 (-22.19, 31.80) | 0.72 |  | - | - | - |  |  |  |  |  |  |  |  |  |  |  |  |  |  |  |  |  |  |  |  |  |  |  |  |  |  |  |  |  |  |  |  |  |
| Participants’ health status | | | | | | | | | | | | | |  |  |  |  |  |  |  |  |  |  |  |  |  |  |  |  |  |  |  |  |  |  |  |  |  |  |  |  |
| Heart disease | 1 | 239 | -3.30 (-10.52, 3.90) | 0.36 |  | - | - | - | 0.15 |  | 1.12 | 0.00 | **0.01** |  |  |  |  |  |  |  |  |  |  |  |  |  |  |  |  |  |  |  |  |  |  |  |  |  |  |  |  |
| Healthy | 2 | 650 | 2.06 (0.42, 3.70) | **0.01** |  | 0.14 | 0.70 | 0.00 |  |  |  |  |  |  |  |  |  |  |  |  |  |  |  |  |  |  |  |  |  |  |  |  |  |  |  |  |  |  |  |  |  |
| Others | 4 | 1416 | 3.98 (1.14, 6.82) | 0.006 |  | 2.14 | 0.54 | 0.00 |  |  |  |  |  |  |  |  |  |  |  |  |  |  |  |  |  |  |  |  |  |  |  |  |  |  |  |  |  |  |  |  |  |
| Hardy-Weinberg equilibrium | | | | | | | | | | | | | |  |  |  |  |  |  |  |  |  |  |  |  |  |  |  |  |  |  |  |  |  |  |  |  |  |  |  |  |
| Equilibrium | 5 | 2023 | 2.48 (0.40, 4.56) | **0.01** |  | 6.07 | 0.30 | 17.60 | 0.67 |  | 1.61 | 13.09 | 0.10 |  |  |  |  |  |  |  |  |  |  |  |  |  |  |  |  |  |  |  |  |  |  |  |  |  |  |  |  |
| Disequilibrium | 1 | 174 | -1.77 (-11.44, 7.89) | 0.71 |  | - | - | - |  |  |  |  |  |  |  |  |  |  |  |  |  |  |  |  |  |  |  |  |  |  |  |  |  |  |  |  |  |  |  |  |  |
| NM | 1 | 108 | 3.50 (-4.21, 11.21) | 0.37 |  | - | - | - |  |  |  |  |  |  |  |  |  |  |  |  |  |  |  |  |  |  |  |  |  |  |  |  |  |  |  |  |  |  |  |  |  |
| Quality score | | | | | | | | | | | | | |  |  |  |  |  |  |  |  |  |  |  |  |  |  |  |  |  |  |  |  |  |  |  |  |  |  |  |  |
| High quality | 4 | 1846 | 2.71 (0.85, 4.57) | **0.004** |  | 3.53 | 0.31 | 14.90 | 0.34 |  | 1.51 | 24.76 | 0.19 |  |  |  |  |  |  |  |  |  |  |  |  |  |  |  |  |  |  |  |  |  |  |  |  |  |  |  |  |
| Medium quality | 3 | 459 | -0.09 (-5.29, 5.11) | 0.93 |  | 1.61 | 0.44 | 0.00 |  |  |  |  |  |  |  |  |  |  |  |  |  |  |  |  |  |  |  |  |  |  |  |  |  |  |  |  |  |  |  |  |  |
| Design of study | | | | | | | | | | | | | |  |  | | |  | | |  | |  | |  | |  | |  | |  | |  | |  | |  | |  | |  |
| Case-control | 3 | 1087 | 1.92 (-5.59, 9.43) | 0.61 |  | 4.81 | 0.09 | 58.50 | 0.32 |  | 1.26 | 22.45 | 0.16 |  |  |  |  |  |  |  |  |  |  |  |  |  |  |  |  |  |  |  |  |  |  |  |  |  |  |  |  |
| Cross-sectional | 4 | 1218 | 2.00 (0.46, 3.54) | **0.01** |  | 0.24 | 0.97 | 0.00 |  |  |  |  |  |  |  |  |  |  |  |  |  |  |  |  |  |  |  |  |  |  |  |  |  |  |  |  |  |  |  |  |  |
| Adjustment of confounders | | | | | | | | | | | | | |  | | |  | | |  |  |  |  |  |  |  |  |  |  |  |  |  |  |  |  |  |  |  |  |  |  |
| Adjusted | 3 | 1351 | 3.34 (0.42, 6.27) | 0.02 |  | 3.28 | 0.19 | 39.10 | 0.33 |  | 1.18 | 31.73 | 0.25 |  |  |  |  |  |  |  |  |  |  |  |  |  |  |  |  |  |  |  |  |  |  |  |  |  |  |  |  |
| Unadjusted | 4 | 954 | 0.80 (-2.58, 4.18) | 0.64 |  | 1.80 | 0.61 | 0.00 |  |  |  |  |  |  |  |  |  |  |  |  |  |  |  |  |  |  |  |  |  |  |  |  |  |  |  |  |  |  |  |  |  |
| **Homozygous model (AA vs. GG)** | | | | | | | | | | | | | |  |  |  |  |  |  |  |  |  |  |  |  |  |  |  |  |  |  |  |  |  |  |  |  |  |  |  |  |
| Ethnicity | | | | | | | | | | | | | |  |  |  |  |  |  |  |  |  |  |  |  |  |  |  |  |  |  |  |  |  |  |  |  |  |  |  |  |
| Asian | 3 | 454 | 0.53 (-3.43, 4.49) | 0.79 |  | 1.67 | 0.43 | 0.00 | 0.15 |  | 3.00 | 78.22 | 0.07 |  |  |  |  |  |  |  |  |  |  |  |  |  |  |  |  |  |  |  |  |  |  |  |  |  |  |  |  |
| Caucasian | 3 | 686 | 6.80 (-1.81, 15.42) | 0.12 |  | 24.98 | <0.001 | 92.00 |  |  |  |  |  |  |  |  |  |  |  |  |  |  |  |  |  |  |  |  |  |  |  |  |  |  |  |  |  |  |  |  |  |
| Mix | 1 | 33 | 12.10 (-15.30, 39.50) | 0.38 |  | - | - | - |  |  |  |  |  |  |  |  |  |  |  |  |  |  |  |  |  |  |  |  |  |  |  |  |  |  |  |  |  |  |  |  |  |
| Participants’ health status | | | | | | | | | | | | | |  |  |  |  |  |  |  |  |  |  |  |  |  |  |  |  |  |  |  |  |  |  |  |  |  |  |  |  |
| Heart disease | 1 | 86 | -3.90 (-11.79, 3.97) | 0.33 |  | - | - | - | <0.001 |  | 2.20 | 73.18 | 0.06 |  |  |  |  |  |  |  |  |  |  |  |  |  |  |  |  |  |  |  |  |  |  |  |  |  |  |  |  |
| Healthy | 2 | 326 | 2.15 (0.04, 4.26) | **0.04** |  | 0.33 | 0.56 | 0.00 |  |  |  |  |  |  |  |  |  |  |  |  |  |  |  |  |  |  |  |  |  |  |  |  |  |  |  |  |  |  |  |  |  |
| Others | 4 | 761 | 8.07 (-1.32, 17.47) | 0.09 |  | 13.88 | 0.003 | 78.40 |  |  |  |  |  |  |  |  |  |  |  |  |  |  |  |  |  |  |  |  |  |  |  |  |  |  |  |  |  |  |  |  |  |
| Hardy-Weinberg equilibrium | | | | | | | | | | | | | |  |  |  |  |  |  |  |  |  |  |  |  |  |  |  |  |  |  |  |  |  |  |  |  |  |  |  |  |
| Equilibrium | 5 | 1065 | 4.30 (-1.95, 10.56) | 0.17 |  | 29.51 | <0.001 | 83.10 | 0.46 |  | 1.85 | 79.64 | 0.32 |  |  |  |  |  |  |  |  |  |  |  |  |  |  |  |  |  |  |  |  |  |  |  |  |  |  |  |  |
| Disequilibrium | 1 | 51 | -2.82 (-13.77, 8.11) | 0.61 |  | - | - | - |  |  |  |  |  |  |  |  |  |  |  |  |  |  |  |  |  |  |  |  |  |  |  |  |  |  |  |  |  |  |  |  |  |
| NM | 1 | 57 | 4.68 (-4.17, 13.53) | 0.30 |  | - | - | - |  |  |  |  |  |  |  |  |  |  |  |  |  |  |  |  |  |  |  |  |  |  |  |  |  |  |  |  |  |  |  |  |  |
| Quality score | | | | | | | | | | | | | |  |  |  |  |  |  |  |  |  |  |  |  |  |  |  |  |  |  |  |  |  |  |  |  |  |  |  |  |
| High quality | 4 | 967 | 6.12 (-0.85, 13.10) | 0.08 |  | 26.40 | <0.001 | 88.60 | 0.16 |  | 2.23 | 82.69 | 0.32 |  |  |  |  |  |  |  |  |  |  |  |  |  |  |  |  |  |  |  |  |  |  |  |  |  |  |  |  |
| Medium quality | 3 | 206 | 0.08 (-5.99, 6.16) | 0.97 |  | 2.10 | 0.35 | 4.90 |  |  |  |  |  |  |  |  |  |  |  |  |  |  |  |  |  |  |  |  |  |  |  |  |  |  |  |  |  |  |  |  |  |
| Design of study | | | | | | | | | | | | | |  |  |  |  |  |  |  |  |  |  |  |  |  |  |  |  |  |  |  |  |  |  |  |  |  |  |  |  |
| Case-control | 3 | 509 | 5.21 (-9.86, 20.28) | 0.49 |  | 15.03 | 0.001 | 86.70 | <0.001 |  | 2.21 | 84.48 | 0.25 |  |  |  |  |  |  |  |  |  |  |  |  |  |  |  |  |  |  |  |  |  |  |  |  |  |  |  |  |
| Cross-sectional | 4 | 664 | 2.17 (0.25, 4.08) | **0.02** |  | 0.84 | 0.83 | 0.00 |  |  |  |  |  |  |  |  |  |  |  |  |  |  |  |  |  |  |  |  |  |  |  |  |  |  |  |  |  |  |  |  |  |
| Adjustment of confounders | | | | | | | | | | | | | |  | | |  | | |  |  |  |  |  |  |  |  |  |  |  |  |  |  |  |  |  |  |  |  |  |  |
| Adjusted | 3 | 662 | 8.11 (-2.25, 18.48) | 0.12 |  | 25.26 | <0.001 | 92.10 | 0.09 |  | 1.87 | 83.78 | 0.35 |  |  |  |  |  |  |  |  |  |  |  |  |  |  |  |  |  |  |  |  |  |  |  |  |  |  |  |  |
| Unadjusted | 4 | 511 | 1.22 (-2.39, 4.84) | 0.50 |  | 2.37 | 0.50 | 0.00 |  |  |  |  |  |  |  |  |  |  |  |  |  |  |  |  |  |  |  |  |  |  |  |  |  |  |  |  |  |  |  |  |  |
| **Heterozygous model (GA vs. GG)** | | | | | | | | | | | | | |  |  |  |  |  |  |  |  |  |  |  |  |  |  |  |  |  |  |  |  |  |  |  |  |  |  |  |  |
| Ethnicity | | | | | | | | | | | | | |  |  |  |  |  |  |  |  |  |  |  |  |  |  |  |  |  |  |  |  |  |  |  |  |  |  |  |  |
| Asian | 3 | 762 | 0.69 (-1.70, 3.08) | 0.57 |  | 0.74 | 0.69 | 0.00 | 0.18 |  | 2.06 | 83.93 | 0.11 |  |  |  |  |  |  |  |  |  |  |  |  |  |  |  |  |  |  |  |  |  |  |  |  |  |  |  |  |
| Caucasian | 3 | 1000 | 4.30 (-3.49, 12.10) | 0.27 |  | 35.85 | <0.001 | 94.00 |  |  |  |  |  |  |  |  |  |  |  |  |  |  |  |  |  |  |  |  |  |  |  |  |  |  |  |  |  |  |  |  |  |
| Mix | 1 | 51 | 9.30 (-8.41, 27.01) | 0.30 |  | - | - | - |  |  |  |  |  |  |  |  |  |  |  |  |  |  |  |  |  |  |  |  |  |  |  |  |  |  |  |  |  |  |  |  |  |
| Participants’ health status | | | | | | | | | | | | | |  |  |  |  |  |  |  |  |  |  |  |  |  |  |  |  |  |  |  |  |  |  |  |  |  |  |  |  |
| Heart disease | 1 | 214 | -0.84 (-5.33, 3.64) | 0.71 |  | - | - | - | <0.001 |  | 1.57 | 81.50 | 0.08 |  |  |  |  |  |  |  |  |  |  |  |  |  |  |  |  |  |  |  |  |  |  |  |  |  |  |  |  |
| Healthy | 2 | 466 | 0.19 (-1.65, 2.04) | 0.83 |  | 0.36 | <0.001 | 85.90 |  |  |  |  |  |  |  |  |  |  |  |  |  |  |  |  |  |  |  |  |  |  |  |  |  |  |  |  |  |  |  |  |  |
| Others | 4 | 1133 | 6.59 (-1.41, 14.61) | 0.10 |  | 21.23 | 0.55 | 0.00 |  |  |  |  |  |  |  |  |  |  |  |  |  |  |  |  |  |  |  |  |  |  |  |  |  |  |  |  |  |  |  |  |  |
| Hardy-Weinberg equilibrium | | | | | | | | | | | | | |  |  |  |  |  |  |  |  |  |  |  |  |  |  |  |  |  |  |  |  |  |  |  |  |  |  |  |  |
| Equilibrium | 5 | 1577 | 3.89 (-1.24, 9.03) | 0.13 |  | 37.82 | <0.001 | 86.80 | 0.44 |  | 1.25 | 83.91 | 0.36 |  |  |  |  |  |  |  |  |  |  |  |  |  |  |  |  |  |  |  |  |  |  |  |  |  |  |  |  |
| Disequilibrium | 1 | 158 | -1.35 (-7.62, 4.91( | 0.67 |  | - | - | - |  |  |  |  |  |  |  |  |  |  |  |  |  |  |  |  |  |  |  |  |  |  |  |  |  |  |  |  |  |  |  |  |  |
| NM | 1 | 78 | 1.80 (-3.76, 7.36) | 0.52 |  | - | - | - |  |  |  |  |  |  |  |  |  |  |  |  |  |  |  |  |  |  |  |  |  |  |  |  |  |  |  |  |  |  |  |  |  |
| Quality score | | | | | | | | | | | | | |  |  |  |  |  |  |  |  |  |  |  |  |  |  |  |  |  |  |  |  |  |  |  |  |  |  |  |  |
| High quality | 4 | 1431 | 4.46 (-1.58, 10.51) | 0.14 |  | 37.54 | <0.001 | 92.00 | 0.18 |  | 1.62 | 86.24 | 0.33 |  |  |  |  |  |  |  |  |  |  |  |  |  |  |  |  |  |  |  |  |  |  |  |  |  |  |  |  |
| Medium quality | 3 | 382 | 0.27 (-3.20, 3.74) | 0.87 |  | 0.68 | 0.71 | 0.00 |  |  |  |  |  |  |  |  |  |  |  |  |  |  |  |  |  |  |  |  |  |  |  |  |  |  |  |  |  |  |  |  |  |
| Design of study | | | | | | | | | | | | | |  | |  | | |  | | |  | |  | |  | |  | |  | |  | |  | |  | |  | |  | |
| Case-control | 3 | 838 | 5.36 ( -5.33, 16.05) | 0.32 |  | 18.31 | <0.001 | 89.10 | <0.001 |  | 1.44 | 87.34 | 0.32 |  |  |  |  |  |  |  |  |  |  |  |  |  |  |  |  |  |  |  |  |  |  |  |  |  |  |  |  |
| Cross-sectional | 4 | 975 | 0.58 (-0.96, 2.12) | 0.46 |  | 1.67 | 0.64 | 0.00 |  |  |  |  |  |  |  |  |  |  |  |  |  |  |  |  |  |  |  |  |  |  |  |  |  |  |  |  |  |  |  |  |  |
| Adjustment of confounders | | | | | | | | | | | | | |  | | |  | | |  |  |  |  |  |  |  |  |  |  |  |  |  |  |  |  |  |  |  |  |  |  |
| Adjusted | 3 | 973 | 6.09 (-3.46, 15.65) | 0.21 |  | 36.06 | <0.001 | 94.50 | 0.08 |  | 1.35 | 86.68 | 0.36 |  |  |  |  |  |  |  |  |  |  |  |  |  |  |  |  |  |  |  |  |  |  |  |  |  |  |  |  |
| Unadjusted | 4 | 840 | 0.86 (-1.33, 3.06) | 0.44 |  | 0.87 | 0.83 | 0.00 |  |  |  |  |  |  |  |  |  |  |  |  |  |  |  |  |  |  |  |  |  |  |  |  |  |  |  |  |  |  |  |  |  |

^a^ All analysis was done using random effects model. WMD, weighted mean difference; 95% CI, 95% confidence interval; NM, not mention.

**Supplementary Table S10-**Sensitivity analysis

|  | **Author** | **Year** | **WMDa (95%CI)** |
| --- | --- | --- | --- |
| **Body mass index (kg/m2)** | | | |
| Dominant model (AA+GA vs. GG) | Solakivi et al. [20] | 2015 | 0.35 (0.01, 0.70) |
| Heterozygous model (GA vs. GG) | Solakivi et al. [20] | 2015 | 0.47 (0.10, 0.84) |
| **Waist circumference (cm)** | | | |
| Dominant model (AA+GA vs. GG) | Ma et al.[28] | 2004 | -3.10 (-5.72, -0.43) |
| **Triglyceride (mg/dl)** | | | |
| Dominant model (AA+GA vs. GG) | Ramoset al.[31] | 2013 | -9.57 (-17.04, -2.10) |
| Heterozygous model (GA vs. GG) | Ramoset al.[31] | 2013 | -7.32 (-13.77, -.87) |
| **HDL cholesterol (mg/dl)** | | | |
| Recessive model (AA vs. GA + GG) | Bayoumyet al. [16]  Ma et al.[28]  Ramoset al. [32]  Yuan [21]  Zhang [23]  Momeni-Moghaddam [19] | 2016  2004  2016  2015  2015  2019 | 0.32 (-0.41, 1.07)  1.17 (-.186, 2.52)  1.30 (-.005, 2.60)  1.22 (-0.03, 2.49)  2.01(-0.10, 4.13)  1.24 (-0.06, 2.54) |
| Homozygous model (AA vs. GG) | Ramoset al.[31] | 2013 | 2.65 (0.24, 5.06) |
| Heterozygous model (GA vs. GG) | Ramoset al.[31] | 2013 | -.368 (-0.51, -0.22) |
| **LDL cholesterol (mg/dl)** | | | |
| Recessive model (AA vs. GA + GG) | Bayoumyet al. [16] | 2016 | 2.23 (2.06, 2.40) |
| Heterozygous model (GA vs. GG) | Zhang [23] | 2015 | -4.70 (-7.81, -1.60) |
| **Fasting blood glucose (mg/dl)** | | | |
| Allelic model (A vs. G) | Solakivi et al. [20]  Momeni-Moghaddam [19] | 2015  2019 | 0.96 (0.08, 1.84)  2.75 (1.35, 5.15) |
| Homozygous model (AA vs. GG) | Momeni-Moghaddam [19] | 2019 | 5.81 (8.06, 11.56) |

^a^WMD, weighted mean difference; 95% CI, 95% confidence interval

**Supplementary Table S11-** The strategy used for online database search

| 1 | "rs1761667", "-31118 G>A", "-31118 G > A", "-31118 G> A", "31118 G >A" |
| --- | --- |
| 2 | ("rs1761667", "-31118 G>A", "-31118 G > A", "-31118 G> A", "31118 G >A") AND ("GWAS" OR "Genome-wide association studies" OR "GWASs" OR "Genome wide association studies" OR "Genome wide association study" OR "Genome-wide association study" OR "GWA study" OR "whole genome association study" OR "WGA study" OR "WGAS" OR "Whole-genome association study" OR "whole genome association studies" OR "whole-genome association studies" OR "WGA" OR "Whole Genome Association Analysis" OR "Whole-Genome Association Analysis" OR "Genome Wide Association Analysis" OR "Genome-Wide Association Analysis") |

| **0**  **1**  **2**  **3**  **-10**  **-5**  **0**  **5**   1. **Allelic model (A vs. G)**   **SE of differences in mean**  **Difference in mean** | **0**  **2**  **4**  **6**  **-10**  **-5**  **0**  **5**  **10**  **B) Dominant model (AA+GA vs. GG)**  **SE of differences in mean**  **Difference in mean** |
| --- | --- |
| **0**  **2**  **4**  **6**  **-10**  **0**  **10**  **C) Recessive model (AA vs. GA + GG)**  **SE of differences in mean**  **Difference in mean** | **0**  **2**  **4**  **6**  **8**  **-20**  **-10**  **0**  **10**  **D) Homozygous model (AA vs. GG)**  **SE of differences in mean**  **Difference in mean** |
| **0**  **2**  **4**  **6**  **-10**  **-5**  **0**  **5**  **10**  **E) Heterozygous model (GA vs. GG)**  **SE of differences in mean**  **Difference in mean** |  |

**Supplementary Figure S1-** Begg's funnel plots (with pseudo 95% CIs) depicting the effect sizes (difference in means) versus their standard errors (SEs)for studies which examined the association between genotype distribution of rs1761667 and waist circumference.

| **0**  **0.5**  **1**  **1.5**  **-10**  **-5**  **0**  **5**  **SE of differences in mean**  **Difference in mean**   1. **Allelic model (A vs. G)** | **B) Dominant model (AA+GA vs. GG)**  **0**  **1**  **2**  **3**  **4**  **-30**  **-20**  **-10**  **0**  **10**  **SE of differences in mean**  **Difference in mean** |
| --- | --- |
| **D) Homozygous model (AA vs. GG)**  **0**  **1**  **2**  **3**  **4**  **-40**  **-20**  **0**  **SE of differences in mean**  **Difference in mean**    **C) Recessive model (AA vs. GA + GG)**  **Difference in mean**  **0**  **1**  **2**  **3**  **4**  **-20**  **-10**  **0**  **SE of differences in mean** |  |
|  |  |

**Supplementary Figure S2-** Begg's funnel plots (with pseudo 95% CIs) depicting the effect sizes (difference in means) versus their standard errors (SEs)for studies which examined the association between genotype distribution of rs1761667 and systolic blood pressure.

**E) Heterozygous model (GA vs. GG)**

**0**

**1**

**2**

**3**

**4**

**-20**

**-10**

**0**

**10**

**SE of differences in mean**

**Difference in mean**

| **0**  **0.5**  **1**  **1.5**  **2**  **-4**  **-2**  **0**  **2**  **4**   1. **Allelic model (A vs. G)**   **SE of differences in mean**  **Difference in mean** | **0**  **2**  **4**  **6**  **8**  **-20**  **-10**  **0**  **10**  **20**  **B) Dominant model (AA+GA vs. GG)**  **Difference in mean** |
| --- | --- |
| **0**  **1**  **2**  **3**  **-10**  **-5**  **0**  **5**  **C) Recessive model (AA vs. GA + GG)**  **Difference in mean** | **0**  **2**  **4**  **6**  **8**  **-20**  **-10**  **0**  **10**  **20**  **D) Homozygous model (AA vs. GG)**  **Difference in mean** |
|  |  |

**Supplementary Figure S3-** Begg's funnel plots (with pseudo 95% CIs) depicting the effect sizes (difference in means) versus their standard errors (SEs)for studies which examined the association between genotype distribution of rs1761667 and diastolic blood pressure.

**0**

**2**

**4**

**6**

**8**

**-20**

**-10**

**0**

**10**

**20**

**E) Heterozygous model (GA vs. GG)**

**Difference in mean**

| **0**  **5**  **10**  **-20**  **0**  **20**   1. **Allelic model (A vs. G)**   **Difference in mean** | **0**  **5**  **10**  **15**  **-40**  **-20**  **0**  **20**  **40**  **B) Dominant model (AA+GA vs. GG)**  **Difference in mean** |
| --- | --- |
| **0**  **5**  **10**  **15**  **20**  **-40**  **-20**  **0**  **20**  **40**  **C) Recessive model (AA vs. GA + GG)**  **Difference in mean**    | **0**  **5**  **10**  **15**  **20**  **-40**  **-20**  **0**  **20**  **40**  **D) Homozygous model (AA vs. GG)**  **Difference in mean**    |
| **0**  **5**  **10**  **15**  **20**  **-40**  **-20**  **0**  **20**  **40**  **E) Heterozygous model (GA vs. GG)**  **Difference in mean**    |  |

**Supplementary Figure S4-** Begg's funnel plots (with pseudo 95% CIs) depicting the effect sizes (difference in means) versus their standard errors (SEs)for studies which examined the association between genotype distribution of rs1761667 and fasting blood glucose.

**Overall (I-squared = 31.2%, p = 0.14)**

Momeni-Moghaddam, M (2019)

Fujii, R (2019)

Ramos-Lopez, O (2016)

Melis, M (2017)

Madden, J (2008)

Dalton, M (2013)

Yuan, Y (2015)

Boghdady,A (2016)

Shen, Y. C (2017)

Yang, Y. (2018)

Zhang, Y (2014)

**0.03 (-0.25, 0.31)**

-0.39 (-1.29, 0.50)

-0.01 (-0.47, 0.45)

1.07 (-0.24, 2.38)

1.59 (-0.29, 3.47)

-0.35 (-1.42, 0.72)

0.55 (-0.70, 1.80)

-0.05 (-0.83, 0.73)

-0.64 (-1.11, -0.16)

0.32 (-1.07, 1.72)

0.11 (-0.83, 1.05)

0.34 (-0.21, 0.89)

0.18 (-0.51, 0.86)

**100.00**

7.43

16.41

4.00

2.08

5.62

4.34

9.07

15.95

3.59

6.93

13.82

10.75

-3.47

0

3.47

**Study (year)**

**WMD (95% CI)**

**%Weight**

1. **Allelic model (A vs. G)**

**Overall (I-squared = 17.9%, p = 0.26)**

Momeni-Moghaddam, M (2019)

Yang, Y. (2018)

Dalton, M (2013)

Zhang, Y (2014)

Melis, M (2017)

Shen, Y. C (2017)

Solakivi, T (2015)

Boghdady,A (2016)

Yuan, Y (2015)

Ramos-Lopez, O (2016)

Madden, J (2008)

Fujii, R (2019)

**0.23 (-0.15, 0.62)**

-0.37 (-1.87, 1.13)

0.47 (-0.25, 1.18)

1.29 (-0.59, 3.16)

0.75 (-0.29, 1.80)

0.68 (-2.05, 3.40)

0.27 (-1.44, 1.97)

-0.84 (-1.73, 0.04)

2.52 (0.06, 4.97)

0.32 (-0.77, 1.41)

1.10 (-1.01, 3.21)

-0.10 (-1.91, 1.70)

0.05 (-0.54, 0.64)

**100.00**

5.76

18.06

3.82

10.48

1.88

4.57

13.44

2.31

9.88

3.07

4.12

22.60

0

-4.97

4.97

**Study (year)**

**WMD (95% CI)**

**%Weight**

**B) Dominant model (AA+GA vs. GG)**

**Overall (I-squared = 41.2%, p = 0.06)**

Solakivi, T (2015)

Momeni-Moghaddam, M (2019)

Yang, Y. (2018)

Fujii, R (2019)

Melis, M (2017)

Yuan, Y (2015)

Madden, J (2008)

Zhang, Y (2014)

Boghdady,A (2016)

Shen, Y. C (2017)

Dalton, M (2013)

Ramos-Lopez, O (2016)

**-0.30 (-0.85, 0.26)**

-0.88 (-1.60, -0.15)

-1.30 (-3.14, 0.53)

0.30 (-0.74, 1.35)

-0.24 (-1.20, 0.71)

4.55 (0.93, 8.16)

-0.63 (-1.88, 0.62)

-0.78 (-2.47, 0.91)

-1.32 (-3.16, 0.52)

-1.61 (-4.25, 1.04)

0.01 (-1.30, 1.32)

-0.08 (-2.07, 1.91)

1.81 (-0.22, 3.84)

**100.00**

16.30

6.47

12.49

13.53

2.11

10.49

7.25

6.44

3.64

9.96

5.74

5.57

0

-8.16

8.16

**Study (year)**

**WMD (95% CI)**

**%Weight**

**C) Recessive model (AA vs. GA + GG)**

**Overall (I-squared = 35.0%, p = 0.11)**

Fujii, R (2019)

Melis, M (2017)

Dalton, M (2013)

Madden, J (2008)

Solakivi, T (2015)

Yuan, Y (2015)

Yang, Y. (2018)

Shen, Y. C (2017)

Ramos-Lopez, O (2016)

Boghdady,A (2016)

Momeni-Moghaddam, M (2019)

Zhang, Y (2014)

**-0.11 (-0.72, 0.51)**

-0.20 (-1.19, 0.79)

4.25 (0.25, 8.25)

0.80 (-1.50, 3.10)

-0.64 (-2.78, 1.50)

-1.30 (-2.31, -0.29)

-0.30 (-1.67, 1.07)

0.43 (-0.70, 1.56)

0.20 (-1.65, 2.05)

2.20 (-0.31, 4.71)

0.70 (-2.59, 3.99)

-1.44 (-3.60, 0.71)

-0.75 (-2.74, 1.24)

**100.00**

15.73

2.18

5.64

6.32

15.44

11.52

14.06

7.80

4.92

3.10

6.24

7.06

0

-8.25

8.25

**Study (year)**

**WMD (95% CI)**

**%Weight**

**D) Homozygous model (AA vs. GG)**

**Overall (I-squared = 15.7%, p = 0.29)**

Fujii, R (2019)

Melis, M (2017)

Dalton, M (2013)

Madden, J (2008)

Solakivi, T (2015)

Yuan, Y (2015)

Yang, Y. (2018)

Shen, Y. C (2017)

Ramos-Lopez, O (2016)

Boghdady,A (2016)

Momeni-Moghaddam, M (2019)

Zhang, Y (2014)

**0.36 (-0.04, 0.76)**

0.10 (-0.53, 0.73)

-0.45 (-3.27, 2.38)

1.51 (-0.54, 3.56)

0.21 (-1.70, 2.12)

-0.60 (-1.53, 0.33)

0.60 (-0.62, 1.82)

0.63 (-0.17, 1.43)

0.30 (-1.51, 2.11)

0.50 (-1.75, 2.75)

3.00 (0.52, 5.48)

-0.19 (-1.72, 1.33)

0.98 (-0.07, 2.03)

**100.00**

23.20

1.92

3.53

4.03

13.82

8.98

17.08

4.46

2.98

2.47

6.06

11.47

0

-5.48

5.48

**Study (year)**

**WMD (95% CI)**

**%Weight**

**E) Heterozygous model (GA vs. GG)**

**Supplementary Figure S5-** Forest plot for studies which examined the association between genotype distribution of rs1761667 and body mass index (kg/m^2^) in adults.

**Overall (I-squared = 52.3%, p = 0.078)**

Boghdady,A (2016)

Fujii, R (2019)

Bayoumy, N. M (2012)

Ma, X (2004)

7.70 (-2.72, 18.12)

-2.43 (-4.83, -0.02)

0.03 (-1.74, 1.81)

-5.58 (-11.92, 0.76)

0.59 (-1.91, 3.09)

**100.00**

3.60

27.67

33.35

8.55

26.84

0

-18.1

18.10

**Study (year)**

**WMD (95% CI)**

**%Weight**

**B) Dominant model (AA+GA vs. GG)**

**Overall (I-squared = 66.3%, p = 0.018)**

Boghdady, A (2016)

Solakivi, T (2015)

Fujii, R (2019)

Bayoumy, N. M (2012)

Ma, X (2004)

**-2.33 (-4.71, 0.06)**

-2.89 (-12.54, 6.76)

-2.60 (-4.67, -0.54)

-0.34 (-3.69, 3.01)

-6.27 (-9.23, -3.30)

-0.09 (-2.37, 2.19)

**100.00**

5.17

26.82

20.20

22.12

25.70

0

-12.5

12.5

**Study (year)**

**WMD (95% CI)**

**%Weight**

**C) Recessive model (AA vs. GA + GG)**

**Overall (I-squared = 65.7%, p = 0.02)**

Boghdady,A (2016)

Solakivi, T (2015)

Fujii, R (2019)

Bayoumy, N. M (2012)

Ma, X (2004)

**-2.25 (-5.49, 0.98)**

3.83 (-9.01, 16.67)

-3.80 (-6.58, -1.02)

-0.30 (-3.73, 3.13)

-10.00 (-16.57, -3.43)

0.40 (-2.50, 3.30)

**100.00**

5.38

27.84

25.13

14.32

27.33

0

-16.7

16.7

**Study (year)**

**WMD (95% CI)**

**%Weight**

**D) Homozygous model (AA vs. GG)**

**Overall (I-squared = 57.7%, p = 0.051)**

Ma, X (2004)

Fujii, R (2019)

Boghdady,A (2016)

Solakivi, T (2015)

Bayoumy, N. M (2012)

**-0.92 (-2.20, 0.35)**

0.16 (-1.33, 1.65)

-0.04 (-1.46, 1.39)

1.57 (-3.99, 7.13)

-1.85 (-3.19, -0.52)

-3.00 (-5.23, -0.77)

**100.00**

25.05

25.82

4.63

26.89

17.61

0

-7.13

7.13

**Study (year)**

**WMD (95% CI)**

**%Weight**

1. **Allelic model (A vs. G)**

**Overall (I-squared = 32.4%, p = 0.20)**

Boghdady, A (2016)

Solakivi, T (2015)

Fujii, R (2019)

Bayoumy, N. M (2012)

Ma, X (2004)

**-0.30 (-2.04, 1.45)**

8.73 (-1.95, 19.41)

-1.70 (-4.22, 0.82)

0.10 (-1.78, 1.98)

-4.00 (-10.42, 2.42)

0.70 (-1.98, 3.38)

**100.00**

2.58

27.70

37.28

6.67

25.77

0

-19.4

19.4

**Study (year)**

**WMD (95% CI)**

**%Weight**

**E) Heterozygous model (GA vs. GG)**

**Supplementary Figure S6-** Forest plot for studies which examined the association between genotype distribution of rs1761667 and waist circumference (cm) in adults.

**Overall (I-squared = 64.6%, p = 0.002)**

Mrizak, I (2015)

Bayoumy, N. M (2012)

Yuan, Y (2015)

Ma, X (2004)

Ramos-Lopez, O (2013)

Boghdady, A (2016)

Ramos-Lopez, O (2016)

Zhang, Y (2014)

Momeni-Moghaddam, M (2019)

Zhang, Y (2015)

Solakivi, T (2015)

**0.42 (-1.61, 2.44)**

7.58 (3.03, 12.13)

-15.83 (-24.87, -6.80)

-6.39 (-20.19, 7.42)

3.42 (-1.33, 8.16)

0.84 (-0.83, 2.51)

-7.82 (-28.14, 12.50)

-6.51 (-20.90, 7.89)

-6.23 (-16.91, 4.44)

1.51 (-5.69, 8.70)

1.05 (0.89, 1.21)

-1.11 (-5.02, 2.80)

**100.00**

11.14

4.20

1.99

10.61

21.66

0.96

1.84

3.15

6.04

25.36

13.06

0

-28.1

28.1

**Study (year)**

**WMD (95% CI)**

**%Weight**

1. **Allelic model (A vs. G)**

**Overall (I-squared = 65.6%, p = 0.001)**

Mrizak, I (2015)

Bayoumy, N. M (2012)

Yuan Y (2015)

Ma, X (2004)

Ramos-Lopez, O (2013)

Boghdady, A (2016)

Ramos-Lopez, O (2016)

Zhang, Y (2014)

Momeni-Moghaddam, M (2019)

Zhang, Y (2015)

Solakivi, T (2015)

**0.67 (-2.97, 4.30)**

-4.60 (-13.05, 3.86)

-19.21 (-54.88, 16.46)

-13.31 (-35.24, 8.62)

5.08 (-3.29, 13.45)

8.05 (5.21, 10.90)

-6.59 (-36.18, 23.01)

-1.06 (-32.99, 30.86)

-12.81 (-28.64, 3.02)

-4.17 (-15.92, 7.58)

2.06 (1.88, 2.25)

-1.83 (-9.00, 5.35)

**100.00**

10.76

1.00

2.48

10.88

22.25

1.42

1.23

4.37

6.98

25.76

12.85

0

-54.9

54.9

**Study (year)**

**WMD (95% CI)**

**%Weight**

**B) Dominant model (AA+GA vs. GG)**

**Overall (I-squared = 89.1%, p = 0.000)**

Mrizak, I (2015)

Solakivi, T (2015)

Momeni-Moghaddam, M (2019)

Boghdady,A (2016)

Ramos-Lopez, O (2016)

Bayoumy, N. M (2012)

Zhang, Y (2015)

Ramos-Lopez, O (2013)

Yuan Y (2015)

Ma, X (2004)

Zhang, Y (2014)

**-0.83 (-5.62, 3.95)**

21.92 (14.83, 29.00)

-1.23 (-7.34, 4.87)

16.33 (-2.18, 34.83)

-20.45 (-46.71, 5.81)

-14.45 (-33.67, 4.77)

-35.67 (-47.66, -23.68)

-0.07 (-0.47, 0.34)

-3.80 (-5.88, -1.72)

-0.08 (-21.31, 21.16)

4.19 (-3.26, 11.63)

2.71 (-16.03, 21.45)

**100.00**

12.36

13.29

4.80

2.78

4.54

8.21

16.94

16.44

3.91

12.02

4.71

0

-47.7

47.7

**Study (year)**

**WMD (95% CI)**

**%Weight**

**C) Recessive model (AA vs. GA + GG)**

**Overall (I-squared = 83.3%, p = 0.000)**

Ramos-Lopez, O (2016)

Yuan Y (2015)

Bayoumy, N. M (2012)

Momeni-Moghaddam, M (2019)

Mrizak, I (2015)

Ramos-Lopez, O (2013)

Ma, X (2004)

Boghdady, A (2016)

Solakivi, T (2015)

Zhang, Y (2015)

Zhang, Y (2014)

**-1.37 (-6.53, 3.79)**

4.40 (-28.76, 37.56)

-15.47 (-39.10, 8.16)

-10.00 (-45.76, 25.76)

-6.73 (-18.57, 5.10)

-14.14 (-22.42, -5.86)

11.25 (8.28, 14.22)

4.00 (-4.81, 12.81)

-2.40 (-34.24, 29.44)

-1.56 (-9.06, 5.94)

2.32 (2.14, 2.50)

-13.92 (-30.36, 2.52)

**100.00**

2.15

3.80

1.88

9.40

12.57

17.49

12.06

2.30

18.56

0

45.8

**Study (year)**

**WMD (95% CI)**

**%Weight**

**E) Heterozygous model (GA vs. GG)**

**Overall (I-squared = 51.4%, p = 0.024)**

Momeni-Moghaddam, M (2019)

Zhang, Y (2014)

Boghdady,A (2016)

Mrizak, I (2015)

Ramos-Lopez, O (2013)

Ma, X (2004)

Yuan Y (2015)

Solakivi, T (2015)

Ramos-Lopez, O (2016)

Bayoumy, N. M (2012)

Zhang, Y (2015)

**2.18 (-1.25, 5.61)**

11.52 (-9.04, 32.07)

-5.40 (-26.78, 15.98)

-22.30 (-53.97, 9.37)

11.90 (2.02, 21.78)

4.00 (1.02, 6.98)

7.00 (-2.80, 16.80)

-8.50 (-34.39, 17.39)

-2.33 (-10.65, 5.99)

-11.00 (-44.57, 22.57)

-45.00 (-81.40, -8.60)

1.18 (0.77, 1.59)

**100.00**

2.57

2.39

1.13

8.90

27.14

9.01

1.67

11.35

1.01

0.87

33.94

0

-81.4

81.4

**Study (year)**

**WMD (95% CI)**

**%Weight**

**D) Homozygous model (AA vs. GG)**

**Supplementary Figure S7-** Forest plot for studies which examined the association between genotype distribution of rs1761667 and total cholesterol (mg/dl) in adults.

**Overall (I-squared = 47.3%, p = 0.041)**

Yuan Y (2015)

Madden, J (2008)

Zhang, Y (2014)

Ma, X (2004)

Bayoumy, N. M (2012)

Ramos-Lopez, O (2016)

Fujii, R (2019)

Ramos-Lopez, O (2013)

Momeni-Moghaddam, M (2019)

Boghdady, A (2016)

Dawczynski, Ch (2013)

**-7.11 (-11.06, -3.16)**

-36.59 (-67.30, -5.87)

-5.62 (-24.48, 13.23)

3.98 (-16.08, 24.04)

-3.17 (-7.86, 1.53)

-22.92 (-34.31, -11.52)

-15.20 (-33.51, 3.11)

-5.21 (-11.70, 1.28)

-4.46 (-6.50, -2.42)

-3.17 (-12.65, 6.31)

-22.22 (-50.75, 6.32)

-15.78 (-36.53, 4.97)

**100.00**

1.57

3.81

3.42

20.32

8.46

4.00

16.12

26.49

10.80

1.80

3.22

0

-67.3

67.3

**Study (year)**

**WMD (95% CI)**

**%Weight**

1. **Allelic model (A vs. G)**

**Overall (I-squared = 54.5%, p = 0.015)**

Yuan, Y (2015)

Madden, J (2008)

Zhang, Y (2014)

Ma, X (2004)

Bayoumy, N. M (2012)

Ramos-Lopez, O (2016)

Fujii, R (2019)

Ramos-Lopez, O (2013)

Momeni-Moghaddam, M (2019)

Boghdady, A (2016)

Dawczynski, Ch (2013)

**-7.26 (-14.64, 0.13)**

-58.45 (-103.22, -13.68)

-6.72 (-38.84, 25.40)

-3.65 (-28.07, 20.76)

-5.60 (-14.44, 3.24)

-60.53 (-105.05, -16.00)

-11.44 (-44.75, 21.88)

-4.88 (-13.13, 3.38)

2.58 (-1.01, 6.17)

-10.02 (-25.92, 5.87)

-35.94 (-98.06, 26.18)

-8.82 (-54.38, 36.75)

**100.00**

2.47

4.41

6.80

19.21

2.49

4.15

19.91

24.94

11.90

1.34

2.39

0

-105

105

**Study (year)**

**WMD (95% CI)**

**%Weight**

**B) Dominant model (AA+GA vs. GG)**

**Overall (I-squared = 69.1%, p = 0.000)**

Yuan, Y (2015)

Madden, J (2008)

Zhang, Y (2014)

Ma, X (2004)

Bayoumy, N. M (2012)

Ramos-Lopez, O (2016)

Fujii, R (2019)

Ramos-Lopez, O (2013)

Momeni-Moghaddam, M (2019)

Boghdady, A (2016)

Dawczynski, Ch (2013)

**-14.55 (-22.74, -6.36)**

-26.63 (-83.35, 30.10)

-7.73 (-36.37, 20.91)

36.16 (-14.19, 86.50)

-3.14 (-10.17, 3.88)

-43.33 (-59.51, -27.16)

-28.32 (-52.52, -4.13)

-11.95 (-24.18, 0.29)

-11.32 (-13.84, -8.80)

3.81 (-15.04, 22.65)

-39.22 (-77.81, -0.62)

-31.66 (-60.23, -3.09)

**100.00**

1.89

5.84

2.34

17.76

11.37

7.34

14.03

20.04

9.82

3.69

5.86

0

-86.5

86.5

**Study (year)**

**WMD (95% CI)**

**%Weight**

**C) Recessive model (AA vs. GA + GG)**

**Overall (I-squared = 58.5%, p = 0.007)**

Fujii, R (2019)

Zhang, Y (2014)

Yuan, Y (2015)

Ramos-Lopez, O (2016)

Ramos-Lopez, O (2013)

Dawczynski, Ch (2013)

Momeni-Moghaddam, M (2019)

Ma, X (2004)

Boghdady, A (2016)

Bayoumy, N. M (2012)

Madden, J (2008)

**-13.94 (-23.82, -4.07)**

-13.29 (-26.00, -0.58)

31.00 (-20.30, 82.30)

-58.45 (-121.59, 4.69)

-29.50 (-64.72, 5.72)

-5.25 (-8.96, -1.54)

-29.76 (-78.43, 18.91)

-4.06 (-26.42, 18.30)

-6.66 (-16.61, 3.29)

-61.60 (-127.76, 4.56)

-90.00 (-135.92, -44.08)

-10.62 (-47.65, 26.41)

**100.00**

17.84

3.23

2.23

6.00

24.46

3.54

11.02

20.15

2.05

3.91

5.55

%

0

-136

136

**Study (year)**

**WMD (95% CI)**

**%Weight**

**D) Homozygous model (AA vs. GG)**

**Overall (I-squared = 67.4%, p = 0.001)**

Boghdady, A (2016)

Ramos-Lopez, O (2013)

Fujii, R (2019)

Momeni-Moghaddam, M (2019)

Ma, X (2004)

Ramos-Lopez, O (2016)

Dawczynski, Ch (2013)

Yuan, Y (2015)

Bayoumy, N. M (2012)

Zhang, Y (2014)

Madden, J (2008)

**-6.22 (-15.41, 2.96)**

-29.10 (-92.62, 34.42)

8.75 (5.10, 12.40)

-3.24 (-12.13, 5.65)

-11.00 (-27.27, 5.27)

-5.00 (-14.27, 4.27)

-1.50 (-37.09, 34.09)

2.53 (-44.28, 49.34)

-58.45 (-105.62, -11.28)

-50.00 (-94.59, -5.41)

-8.85 (-34.17, 16.47)

-4.42 (-38.84, 30.00)

**100.00**

1.91

20.89

17.96

12.87

17.71

5.09

3.27

3.23

3.55

8.18

5.36

0

-106

106

**Study (year)**

**WMD (95% CI)**

**%Weight**

**E) Heterozygous model (GA vs. GG)**

**Supplementary Figure S8-** Forest plot for studies which examined the association between genotype distribution of rs1761667 and triglyceride(mg/dl) in adults.

**Overall (I-squared = 72.2%, p = 0.000)**

Zhang, Y (2014)

Madden, J (2008)

Fujii, R (2019)

Ma, X (2004)

Ramos-Lopez, O (2013)

Dawczynski, Ch (2013)

Ramos-Lopez, O (2016)

Yuan, Y (2015)

Zhang, Y (2015)

Momeni-Moghaddam, M (2019)

Boghdady, A (2016)

Bayoumy, N. M (2012)

**0.58 (-0.29, 1.46)**

-0.42 (-4.97, 4.14)

-1.03 (-3.77, 1.70)

0.23 (-1.74, 2.20)

1.97 (0.39, 3.56)

-1.30 (-2.05, -0.55)

-2.34 (-8.62, 3.95)

3.55 (-0.77, 7.86)

2.75 (-1.25, 6.75)

-0.10 (-0.19, -0.01)

0.83 (-1.07, 2.73)

0.88 (-3.99, 5.76)

2.88 (1.43, 4.32)

**100.00**

3.08

6.60

9.59

11.59

16.45

1.75

3.37

3.80

18.68

9.94

2.74

12.40

0

-8.62

8.62

**Study (year)**

**WMD (95% CI)**

**%Weight**

1. **Allelic model (A vs. G)**

**Overall (I-squared = 61.1%, p = 0.003)**

Bayoumy, N. M (2012)

Ramos-Lopez, O (2013)

Zhang, Y (2014)

Ma, X (2004)

Fujii, R (2019)

Madden, J (2008)

Yuan, Y (2015)

Ramos-Lopez, O (2016)

Boghdady, A (2016)

Zhang, Y (2015)

Momeni-Moghaddam, M (2019)

Dawczynski, Ch (2013)

**0.21 (-1.16, 1.59)**

4.58 (0.05, 9.10)

-3.42 (-5.11, -1.73)

-1.01 (-6.63, 4.61)

2.72 (0.03, 5.42)

0.06 (-2.49, 2.60)

-0.95 (-5.82, 3.91)

1.12 (-4.16, 6.40)

7.42 (0.69, 14.14)

-2.09 (-11.60, 7.41)

-0.29 (-0.44, -0.15)

0.32 (-2.56, 3.20)

-0.58 (-11.63, 10.46)

**100.00**

6.40

15.86

4.65

11.57

12.16

5.78

5.12

3.48

1.90

20.78

10.87

1.44

0

-14.1

14.1

**Study (year)**

**WMD (95% CI)**

**%Weight**

**B) Dominant model (AA+GA vs. GG)**

**Overall (I-squared = 62.1%, p = 0.002)**

Momeni-Moghaddam, M (2019)

Boghdady, A (2016)

Zhang, Y (2014)

Yuan, Y (2015)

Zhang, Y (2015)

Madden, J (2008)

Ramos-Lopez, O (2016)

Ramos-Lopez, O (2013)

Dawczynski, Ch (2013)

Bayoumy, N. M (2012)

Ma, X (2004)

Fujii, R (2019)

**1.36 (0.08, 2.64)**

3.69 (-1.62, 9.00)

5.42 (-5.02, 15.85)

0.68 (-10.37, 11.72)

6.82 (-1.81, 15.46)

0.20 (0.02, 0.39)

-1.68 (-5.74, 2.38)

3.72 (-3.48, 10.93)

-0.40 (-1.40, 0.60)

-5.19 (-15.28, 4.90)

6.20 (3.30, 9.10)

2.59 (0.03, 5.16)

1.08 (-3.17, 5.33)

**100.00**

4.75

1.42

1.28

2.03

25.91

7.19

2.81

22.48

1.51

11.16

12.74

6.72

0

-15.8

15.8

**Study (year)**

**WMD (95% CI)**

**%Weight**

**C) Recessive model (AA vs. GA + GG)**

**Overall (I-squared = 55.0%, p = 0.011)**

Yuan, Y (2015)

Madden, J (2008)

Boghdady,A (2016)

Zhang, Y (2015)

Ramos-Lopez, O (2013)

Fujii, R (2019)

Ma, X (2004)

Dawczynski, Ch (2013)

Bayoumy, N. M (2012)

Zhang, Y (2014)

Ramos-Lopez, O (2016)

Momeni-Moghaddam, M (2019)

**-0.31 (-1.62, 1.00)**

-1.16 (-6.38, 4.06)

-0.38 (-5.55, 4.79)

-3.40 (-12.97, 6.17)

-0.38 (-0.52, -0.24)

-3.75 (-5.45, -2.05)

-0.13 (-2.83, 2.57)

2.00 (-0.83, 4.83)

1.35 (-10.22, 12.92)

3.00 (-1.48, 7.48)

-1.16 (-7.06, 4.74)

6.60 (-0.47, 13.67)

-0.21 (-3.11, 2.69)

**100.00**

4.96

5.04

1.74

23.19

16.75

11.73

11.15

1.22

6.26

4.08

3.00

10.88

0

-13.7

13.7

**Study (year)**

**WMD (95% CI)**

**%Weight**

**E) Heterozygous model (GA vs. GG)**

**Overall (I-squared = 70.1%, p = 0.000)**

Ma, X (2004)

Madden, J (2008)

Zhang, Y (2015)

Zhang, Y (2014)

Boghdady, A (2016)

Fujii, R (2019)

Yuan, Y (2015)

Dawczynski, Ch (2013)

Ramos-Lopez, O (2013)

Ramos-Lopez, O (2016)

Momeni-Moghaddam, M (2019)

Bayoumy, N. M (2012)

**1.64 (-0.42, 3.70)**

4.00 (0.75, 7.25)

-1.93 (-7.42, 3.56)

0.00 (-0.21, 0.21)

0.00 (-11.27, 11.27)

2.80 (-10.34, 15.94)

1.03 (-3.38, 5.44)

6.19 (-2.93, 15.31)

-4.18 (-17.33, 8.97)

-3.00 (-4.80, -1.20)

8.90 (0.34, 17.46)

3.54 (-2.16, 9.24)

9.00 (3.83, 14.17)

**100.00**

13.10

8.15

19.38

2.84

2.18

10.26

4.04

2.18

16.91

4.45

7.81

8.73

0

-17.5

17.5

**Study (year)**

**WMD (95% CI)**

**%Weight**

**D) Homozygous model (AA vs. GG)**

**Supplementary Figure S9-** Forest plot for studies which examined the association between genotype distribution of rs1761667 and HDL cholesterol (mg/dl) in adults.

**Overall (I-squared = 83.9%, p = 0.000)**

Ramos-Lopez, O (2013)

Zhang, Y (2014)

Boghdady,A (2016)

Yuan, Y (2015)

Zhang, Y (2015)

Mrizak, I (2015)

Madden, J (2008)

Momeni-Moghaddam, M (2019)

Ramos-Lopez, O (2016)

Bayoumy, N. M (2012)

**-0.63 (-4.40, 3.15)**

7.46 (4.95, 9.98)

-4.95 (-14.37, 4.46)

-4.13 (-16.69, 8.42)

-2.85 (-12.93, 7.22)

1.33 (1.19, 1.46)

8.49 (3.64, 13.33)

-5.03 (-14.54, 4.48)

1.31 (-4.97, 7.58)

-8.47 (-21.56, 4.63)

-21.88 (-32.71, -11.04)

**100.00**

15.73

8.24

5.89

7.67

16.91

13.23

8.16

11.53

5.57

7.07

0

-32.7

32.7

**%Weight**

**Study (year)**

**WMD (95% CI)**

1. **Allelic model (A vs. G)**

**Overall (I-squared = 42.1%, p = 0.077)**

Zhang, Y (2014)

Momeni-Moghaddam, M (2019)

Mrizak, I (2015)

Bayoumy, N. M (2012)

Madden, J (2008)

Zhang, Y (2015)

Ramos-Lopez, O (2013)

Ramos-Lopez, O (2016)

Yuan, Y (2015)

Boghdady,A (2016)

**-0.36 (-3.80, 3.07)**

-7.70 (-22.00, 6.59)

-3.73 (-14.09, 6.63)

-3.36 (-13.01, 6.30)

-36.84 (-64.34, -9.34)

-8.21 (-25.18, 8.76)

2.24 (2.07, 2.41)

3.39 (-0.47, 7.25)

-10.06 (-37.00, 16.87)

-3.69 (-18.34, 10.96)

6.54 (-16.66, 29.74)

**100.00**

5.01

8.52

9.49

1.50

3.70

37.74

25.59

1.56

4.81

2.07

0

-64.3

64.3

**%Weight**

**Study (year)**

**WMD (95% CI)**

**B) Dominant model (AA+GA vs. GG)**

**Overall (I-squared = 93.5%, p = 0.000)**

Bayoumy, N. M (2012)

Zhang, Y (2014)

Madden, J (2008)

Momeni-Moghaddam, M (2019)

Yuan, Y (2015)

Zhang, Y (2015)

Ramos-Lopez, O (2016)

Ramos-Lopez, O (2013)

Boghdady,A (2016)

Mrizak, I (2015)

**-2.69 (-11.53, 6.15)**

-46.67 (-60.46, -32.87)

-5.78 (-25.29, 13.73)

-4.86 (-19.94, 10.21)

14.37 (-1.24, 29.98)

-3.35 (-20.89, 14.19)

0.68 (0.33, 1.03)

-13.53 (-30.24, 3.18)

13.58 (9.58, 17.58)

-21.80 (-43.41, -0.19)

23.11 (16.36, 29.85)

**100.00**

9.96

8.02

9.52

9.33

8.67

13.15

8.95

12.81

7.37

12.22

0

-60.5

60.5

**%Weight**

**Study (year)**

**WMD (95% CI)**

**C) Recessive model (AA vs. GA + GG)**

**Overall (I-squared = 82.6%, p = 0.000)**

Ramos-Lopez, O (2013)

Madden, J (2008)

Boghdady,A (2016)

Momeni-Moghaddam, M (2019)

Yuan, Y (2015)

Mrizak, I (2015)

Bayoumy, N. M (2012)

Zhang, Y (2014)

Zhang, Y (2015)

Ramos-Lopez, O (2016)

**-2.07 (-9.79, 5.64)**

11.50 (6.75, 16.25)

-9.67 (-29.67, 10.33)

-12.80 (-41.42, 15.82)

10.09 (-7.42, 27.60)

-5.03 (-24.60, 14.54)

13.73 (3.20, 24.26)

-70.00 (-98.49, -41.51)

-10.06 (-31.86, 11.74)

1.93 (1.57, 2.29)

-18.00 (-45.90, 9.90)

**100.00**

17.53

8.30

5.24

9.54

8.50

13.91

5.27

7.51

18.76

5.43

0

-98.5

98.5

**%Weight**

**Study (year)**

**WMD (95% CI)**

**D) Homozygous model (AA vs. GG)**

**Overall (I-squared = 67.5%, p = 0.001)**

Boghdady,A (2016)

Ramos-Lopez, O (2013)

Zhang, Y (2015)

Ramos-Lopez, O (2016)

Bayoumy, N. M (2012)

Momeni-Moghaddam, M (2019)

Madden, J (2008)

Zhang, Y (2014)

Mrizak, I (2015)

Yuan, Y (2015)

**-3.99 (-8.78, 0.81)**

11.70 (-11.89, 35.29)

-3.00 (-6.84, 0.84)

2.33 (2.17, 2.49)

-5.70 (-34.13, 22.73)

-25.00 (-52.69, 2.69)

-5.98 (-16.44, 4.47)

-7.35 (-25.07, 10.37)

-7.35 (-22.08, 7.38)

-13.24 (-22.88, -3.60)

-3.09 (-18.97, 12.79)

**100.00**

3.55

21.87

25.44

2.56

2.68

11.51

5.68

7.47

12.53

6.71

0

-52.7

52.7

**E) Heterozygous model (GA vs. GG)**

**%Weight**

**Study (year)**

**WMD (95% CI)**

**Supplementary Figure S10-** Forest plot for studies which examined the association between genotype distribution of rs1761667 and LDL cholesterol (mg/dl) in adults.

**Overall (I-squared = 89.2%, p = 0.000)**13.35

Momeni-Moghaddam, M (2019)

Bayoumy, N. M (2012)6.44

Solakivi, T (2015)

Ma, X (2004)

Fujii, R (2019)

**-2.01 (-4.93, 0.90)**

0.02 (-2.49, 2.52)

-8.75 (-11.47, -6.03)

-0.70 (-2.43, 1.03)

0.68 (-0.82, 2.17)

-1.85 (-4.73, 1.04)

**100.00**

19.55

19.05

21.16

21.56

18.68

0Solakivi, T (2015)

-11.5-45.8

11.5**-0.70 (-2.76, 1.36)**

**Study (year)**

**WMD (95% CI)**

1. **Allelic model (A vs. G)**

**%Weight**Solakivi, T (2015)

**Overall (I-squared = 89.7%, p = 0.000)**

Momeni-Moghaddam, M (2019)

Bayoumy, N. M (2012)

Solakivi, T (2015)

Ma, X (2004)

Fujii, R (2019)

**-4.17 (-9.23, 0.89)**

1.09 (-2.62, 4.80)

-23.95 (-31.20, -16.70)

-1.30 (-4.33, 1.72)

-0.92 (-3.57, 1.73)

-0.79 (-4.39, 2.82)

**100.00**

20.56

15.67

21.35

21.73

20.69

0

-31.2

31.2

**%Weight**

**Study (year)**

**WMD (95% CI)**

**B) Dominant model (AA+GA vs. GG)**

**Overall (I-squared = 96.8%, p = 0.000)**

Ma, X (2004)

Fujii, R (2019)

Momeni-Moghaddam, M (2019)

Solakivi, T (2015)

Bayoumy, N. M (2012)

**-4.83 (-12.96, 3.29)**

2.41 (0.02, 4.80)

-7.61 (-14.04, -1.18)

-2.12 (-6.79, 2.54)

-0.66 (-3.45, 2.14)

-16.33 (-18.84, -13.83)

**100.00**

20.65

18.57

19.66

20.51

20.61

0

-18.8

18.8

**%Weight**

**Study (year)**

**WMD (95% CI)**

**C) Recessive model (AA vs. GA + GG)**

**Overall (I-squared = 95.3%, p = 0.000)**

Fujii, R (2019)

Solakivi, T (2015)

Ma, X (2004)

Momeni-Moghaddam, M (2019)

Bayoumy, N. M (2012)

**-8.43 (-18.09, 1.24)**

-7.40 (-14.01, -0.79)

-1.50 (-5.12, 2.12)

1.00 (-2.14, 4.14)

-1.09 (-6.35, 4.16)

-35.00 (-42.19, -27.81)

**100.00**

19.38

20.69

20.84

20.04

19.06

0

-42.2

42.2

**%Weight**

**Study (year)**

**WMD (95% CI)**

**D) Homozygous model (AA vs. GG)**

**Overall (I-squared = 86.1%, p = 0.000)**

Bayoumy, N. M (2012)

Momeni-Moghaddam, M (2019)

Fujii, R (2019)

Solakivi, T (2015)

Ma, X (2004)

**-3.30 (-7.84, 1.23)**

-20.00 (-27.20, -12.80)

1.44 (-2.41, 5.29)

0.50 (-3.30, 4.30)

-1.20 (-4.37, 1.97)

-2.00 (-4.77, 0.77)

**100.00**

15.01

20.57

20.65

21.61

22.16

0

-27.2

27.2

**E) Heterozygous model (GA vs. GG)**

**%Weight**

**Study (year)**

**WMD (95% CI)**

**Supplementary Figure S11-** Forest plot for studies which examined the association between genotype distribution of rs1761667 and systolic Blood pressure (mmHg) in adults.

**Overall (I-squared = 0.0%, p = 0.430)**

Yuan Y (2015)

Ma, X (2004)

Solakivi, T (2015)

Momeni-Moghaddam, M (2019)

Bayoumy, N. M (2012)

Fujii, R (2019)

**-0.15 (-0.75, 0.46)**

-0.15 (-3.86, 3.55)

0.53 (-0.49, 1.55)

-0.30 (-1.32, 0.71)

-0.04 (-1.77, 1.69)

-2.08 (-5.31, 1.15)

-1.42 (-3.33, 0.49)

**100.00**

2.68

35.52

35.85

12.34

3.53

10.09

0

-5.31

5.31

**%Weight**

**Study (year)**

**WMD (95% CI)**

1. **Allelic model (A vs. G)**

**Overall (I-squared = 47.7%, p = 0.089)**

Momeni-Moghaddam, M (2019)

Fujii, R (2019)

Ma, X (2004)

Bayoumy, N. M (2012)

Yuan Y (2015)

Solakivi, T (2015)

**-0.85 (-2.56, 0.85)**

-1.32 (-5.17, 2.52)

-4.86 (-9.20, -0.52)

1.00 (-0.53, 2.53)

-5.00 (-11.32, 1.32)

0.54 (-5.65, 6.74)

-0.53 (-2.08, 1.02)

**100.00**

13.41

11.30

31.44

6.22

6.44

31.19

0

-11.3

11.3

**%Weight**

**Study (year)**

**WMD (95% CI)**

**C) Recessive model (AA vs. GA + GG)**

**Overall (I-squared = 0.0%, p = 0.972)**

Momeni-Moghaddam, M (2019)

Solakivi, T (2015)

Bayoumy, N. M (2012)

Yuan Y (2015)

Fujii, R (2019)

Ma, X (2004)

**-0.14 (-1.20, 0.92)**

0.54 (-2.65, 3.74)

-0.27 (-2.03, 1.48)

-1.32 (-14.67, 12.04)

-0.69 (-6.27, 4.89)

-0.88 (-3.30, 1.54)

0.36 (-1.62, 2.34)

**100.00**

11.06

36.64

0.63

3.62

19.27

28.78

0

-14.7

14.7

**%Weight**

**Study (year)**

**WMD (95% CI)**

**B) Dominant model (AA+GA vs. GG)**

**Overall (I-squared = 14.8%, p = 0.319)**

Ma, X (2004)

Momeni-Moghaddam, M (2019)

Fujii, R (2019)

Bayoumy, N. M (2012)

Solakivi, T (2015)

Yuan Y (2015)

**-0.57 (-2.14, 1.00)**

1.00 (-1.22, 3.22)

-0.78 (-5.43, 3.87)

-4.90 (-9.38, -0.42)

-5.00 (-19.40, 9.40)

-0.60 (-2.63, 1.43)

0.00 (-7.11, 7.11)

100.00

34.18

10.33

11.05

1.18

38.59

4.67

0

-19.4

19.4

**%Weight**

**Study (year)**

**WMD (95% CI)**

**D) Homozygous model (AA vs. GG)**

**Overall (I-squared = 0.0%, p = 0.997)**

Momeni-Moghaddam, M (2019)

Fujii, R (2019)

Bayoumy, N. M (2012)

Solakivi, T (2015)

Yuan Y (2015)

Ma, X (2004)

**0.00 (-1.11, 1.11)**

0.76 (-2.47, 3.99)

-0.10 (-2.64, 2.44)

0.00 (-13.35, 13.35)

-0.10 (-1.96, 1.76)

-1.00 (-7.04, 5.04)

0.00 (-2.06, 2.06)

**100.00**

11.85

19.19

0.69

35.66

3.40

29.21

0

-13.4

13.4

**E) Heterozygous model (GA vs. GG)**

**%Weight**

**Study (year)**

**WMD (95% CI)**

**Supplementary Figure S12-** Forest plot for studies which examined the association between genotype distribution of rs1761667 and diastolic blood pressure (mmHg) in adults.

**Overall (I-squared = 74.7%, p = 0.001)**

Fujii, R (2019)

Yuan, Y (2015)

Madden, J (2008)

Ramos-Lopez, O (2016)

Solakivi, T (2015)

Ma, X (2004)

Momeni-Moghaddam, M (2019)

**2.07 (-0.11, 4.25)**

1.13 (-0.95, 3.22)

3.66 (-18.00, 25.31)

2.48 (-1.25, 6.22)

4.77 (-9.57, 19.10)

6.32 (4.18, 8.45)

1.06 (-0.02, 2.14)

-1.13 (-3.98, 1.73)

**100.00**

20.55

0.98

14.53

2.12

20.36

23.82

17.65

0

-25.3

25.3

**%Weight**

**Study (year)**

**WMD (95% CI)**

1. **Allelic model (A vs. G)**

**Overall (I-squared = 88.0%, p = 0.000)**

Ma, X (2004)

Yuan, Y (2015)

Solakivi, T (2015)

Fujii, R (2019)

Ramos-Lopez, O (2016)

Madden, J (2008)

Momeni-Moghaddam, M (2019)

**3.64 (-0.89, 8.18)**

0.72 (-1.13, 2.58)

6.64 (-23.14, 36.42)

11.86 (9.06, 14.66)

1.38 (-1.29, 4.05)

10.29 (-6.59, 27.17)

2.87 (-3.00, 8.73)

-1.27 (-5.70, 3.15)

**100.00**

20.33

2.09

19.48

19.62

5.37

15.57

17.54

0

-36.4

36.4

**%Weight**

**Study (year)**

**WMD (95% CI)**

**B) Dominant model (AA+GA vs. GG)**

**Overall (I-squared = 0.4%, p = 0.420)**

Madden, J (2008)

Ma, X (2004)

Solakivi, T (2015)

Yuan, Y (2015)

Momeni-Moghaddam, M (2019)

Ramos-Lopez, O (2016)

Fujii, R (2019)

**2.34 (0.92, 3.75)**

3.50 (-4.21, 11.22)

2.00 (0.32, 3.68)

5.77 (2.04, 9.50)

1.48 (-31.97, 34.93)

-3.31 (-10.52, 3.91)

4.81 (-22.20, 31.81)

1.46 (-3.00, 5.92)

**100.00**

3.34

68.22

14.20

0.18

3.82

0.27

9.96

0

-34.9

34.9

**%Weight**

**Study (year)**

**WMD (95% CI)**

**C) Recessive model (AA vs. GA + GG)**

**Overall (I-squared = 80.3%, p = 0.000)**

Yuan Y (2015)

Fujii, R (2019)

Ramos-Lopez, O (2016)

Madden, J (2008)

Solakivi, T (2015)

Ma, X (2004)

Momeni-Moghaddam, M (2019)

**4.29 (-1.03, 9.60)**

5.40 (-30.69, 41.49)

1.98 (-2.64, 6.60)

12.10 (-15.30, 39.50)

4.68 (-4.18, 13.54)

13.50 (9.55, 17.45)

2.00 (-0.17, 4.17)

-3.91 (-11.79, 3.98)

**100.00**

1.99

20.33

3.25

14.41

21.21

23.10

15.72

0

-41.5

41.5

**%Weight**

**Study (year)**

**WMD (95% CI)**

**D) Homozygous model (AA vs. GG)**

**Overall (I-squared = 85.0%, p = 0.000)**

Ramos-Lopez, O (2016)

Momeni-Moghaddam, M (2019)

Fujii, R (2019)

Ma, X (2004)

Madden, J (2008)

Yuan Y (2015)

Solakivi, T (2015)

**3.08 (-1.18, 7.34)**

9.30 (-8.41, 27.01)

-0.84 (-5.33, 3.65)

1.26 (-1.58, 4.10)

0.00 (-1.96, 1.96)

1.80 (-3.76, 7.36)

7.20 (-27.04, 41.44)

10.98 (7.95, 14.01)

**100.00**

4.57

17.58

19.92

20.89

15.92

1.44

19.68

0

-41.4

41.4

**E) Heterozygous model (GA vs. GG)**

**%Weight**

**Study (year)**

**WMD (95% CI)**

**Supplementary Figure S13-** Forest plot for studies which examined the association between genotype distribution of rs1761667 and fasting blood glucose (mg/dl) in adults.

Madden, J (2008)

Dalton, M (2013)

Zhang, Y (2014)

Solakivi, T (2015)

Yuan, Y (2015)

Boghdady, A (2016)

Ramos-Lopez, O (2016)

Shen, Y. C (2017)

Melis, M (2017)

Yang, Y. (2018)

Fujii, R (2019)

Momeni-Moghaddam, M (2019)

-0.01 (-0.13, 0.11)

0.00 (-0.08, 0.08)

-0.02 (-0.11, 0.07)

-0.03 (-0.13, 0.08)

-0.03 (-0.11, 0.05)

0.09 (-0.31, 0.50)

0.02 (-0.09, 0.13)

0.02 (-0.08, 0.12)

0.01 (-0.06, 0.09)

-0.00 (-0.09, 0.08)

0.01 (-0.13, 0.14)

-0.01 (-0.08, 0.06)

0

-.499

.499

**Study (year)**

**WMD (95% CI)**

1. **Allelic model (A vs. G)**

Madden, J (2008)

Dalton, M (2013)

Zhang, Y (2014)

Solakivi, T (2015)

Yuan, Y (2015)

Boghdady, A (2016)

Ramos-Lopez, O (2016)

Shen, Y. C (2017)

Melis, M (2017)

Yang, Y. (2018)

Fujii, R (2019)

Momeni-Moghaddam, M (2019)

0.11 (-0.21, 0.43)

0.09 (-0.05, 0.22)

0.05 (-0.12, 0.23)

0.01 (-0.17, 0.18)

0.05 (-0.10, 0.20)

0.76 (0.02, 1.51)

0.11 (-0.14, 0.35)

0.07 (-0.09, 0.22)

0.06 (-0.05, 0.17)

0.07 (-0.07, 0.21)

0.30 (-0.41, 1.01)

0.06 (-0.06, 0.18)

0

-1.51

1.51

**Study (year)**

**WMD (95% CI)**

**B) Dominant model (AA+GA vs. GG)**

Madden, J (2008)

Dalton, M (2013)

Zhang, Y (2014)

Solakivi, T (2015)

Yuan, Y (2015)

Boghdady, A (2016)

Ramos-Lopez, O (2016)

Shen, Y. C (2017)

Melis, M (2017)

Yang, Y. (2018)

Fujii, R (2019)

Momeni-Moghaddam, M (2019)

-0.16 (-0.40, 0.09)

-0.11 (-0.23, 0.01)

-0.11 (-0.28, 0.06)

-0.09 (-0.26, 0.08)

-0.13 (-0.27, 0.02)

-0.47 (-1.24, 0.29)

-0.04 (-0.35, 0.28)

-0.03 (-0.26, 0.21)

-0.05 (-0.21, 0.11)

-0.11 (-0.25, 0.03)

-0.14 (-0.44, 0.17)

-0.13 (-0.24, -0.02)

-1.24

1.24

0

**Study (year)**

**WMD (95% CI)**

**C) Recessive model (AA vs. GA + GG)**

Madden, J (2008)

Dalton, M (2013)

Zhang, Y (2014)

Solakivi, T (2015)

Yuan, Y (2015)

Boghdady, A (2016)

Ramos-Lopez, O (2016)

Shen, Y. C (2017)

Melis, M (2017)

Yang, Y (2018)

Fujii, R (2019)

Momeni-Moghaddam, M (2019)

-0.06 (-0.34, 0.21)

-0.09 (-0.23, 0.04)

-0.12 (-0.29, 0.05)

-0.09 (-0.29, 0.11)

-0.14 (-0.28, 0.00)

0.20 (-0.75, 1.16)

0.01 (-0.24, 0.27)

0.02 (-0.20, 0.25)

-0.02 (-0.18, 0.15)

-0.11 (-0.25, 0.03)

-0.03 (-0.35, 0.29)

-0.11 (-0.24, 0.02)

0

-1.16

1.16

**Study (year)**

**WMD (95% CI)**

**D) Homozygous model (AA vs. GG)**

Madden, J (2008)

Dalton, M (2013)

Zhang, Y (2014)

Solakivi, T (2015)

Yuan, Y (2015)

Boghdady, A (2016)

Ramos-Lopez, O (2016)

Shen, Y. C (2017)

Melis, M (2017)

Yang, Y. (2018)

Fujii, R (2019)

Momeni-Moghaddam, M (2019)

0.20 (-0.20, 0.61)

0.13 (-0.01, 0.27)

0.09 (-0.09, 0.28)

0.04 (-0.14, 0.21)

0.10 (-0.06, 0.27)

0.95 (0.17, 1.72)

0.16 (-0.14, 0.46)

0.11 (-0.10, 0.31)

0.09 (-0.03, 0.21)

0.12 (-0.03, 0.26)

0.41 (-0.48, 1.29)

0.11 (-0.02, 0.23)

0

-1.72

1.72

**E) Heterozygous model (GA vs. GG)**

**Study (year)**

**WMD (95% CI)**

**Supplementary Figure S14- Forest** plot of the cumulative meta-analysis for studies which examined the association between genotype distribution of rs1761667 and body mass index (kg/m^2^) in adults

Ma, X (2004)

Bayoumy, N. M (2012)

Solakivi, T (2015)

Boghdady, A (2016)

Fujii, R (2019)

-0.05 (-0.20, 0.10)

-0.38 (-0.67, -0.10)

-0.08 (-0.20, 0.04)

-0.16 (-0.64, 0.33)

-0.10 (-0.37, 0.17)

0

-0.667

0.667

**Study (year)**

**WMD (95% CI)**

1. **Allelic model (A vs. G)**

Ma, X (2004)

Bayoumy, N. M (2012)

Solakivi, T (2015)

Boghdady, A (2016)

Fujii, R (2019)

0.03 (-0.17, 0.23)

-0.70 (-1.61, 0.20)

-0.04 (-0.22, 0.14)

-0.04 (-1.29, 1.21)

0.01 (-0.51, 0.52)

0

-1.61

1.61

**Study (year)**

**WMD (95% CI)**

**B) Dominant model (AA+GA vs. GG)**

Ma, X (2004)

Bayoumy, N. M (2012)

Solakivi, T (2015)

Boghdady,A (2016)

Fujii, R (2019)

-0.23 (-0.59, 0.12)

-0.83 (-1.30, -0.37)

-0.20 (-0.43, 0.02)

-0.60 (-1.19, -0.00)

-0.36 (-0.91, 0.19)

0

-1.3

1.3

**Study (year)**

**WMD (95% CI)**

**C) Recessive model (AA vs. GA + GG)**

Ma, X (2004)

Bayoumy, N. M (2012)

Solakivi, T (2015)

Boghdady,A (2016)

Fujii, R (2019)

-0.16 (-0.61, 0.28)

-1.63 (-2.68, -0.57)

-0.18 (-0.50, 0.13)

-0.66 (-2.53, 1.21)

-0.39 (-1.32, 0.55)

0

-2.68

2.68

**Study (year)**

**WMD (95% CI)**

**D) Homozygous model (AA vs. GG)**

Ma, X (2004)

Bayoumy, N. M (2012)

Solakivi, T (2015)

Boghdady, A (2016)

Fujii, R (2019)

0.04 (-0.14, 0.22)

-0.50 (-1.41, 0.41)

-0.01 (-0.17, 0.14)

0.10 (-1.02, 1.21)

0.06 (-0.40, 0.53)

0

-1.41

1.41

**Study (year)**

**WMD (95% CI)**

**E) Heterozygous model (GA vs. GG)**

**Supplementary Figure S15-** Forest plot of the cumulative meta-analysis for studies which examined the association between genotype distribution of rs1761667 and waist circumference (cm) in adults.

Ma, X (2004)

Bayoumy, N. M (2012)

Ramos-Lopez, O (2013)

Zhang, Y (2014)

Solakivi, T (2015)

Yuan Y (2015)

Mrizak, I (2015)

Zhang, Y (2015)

Boghdady,A (2016)

Ramos-Lopez, O (2016)

Momeni-Moghaddam, M (2019)

-0.17 (-0.57, 0.22)

-0.49 (-0.78, -0.20)

-0.03 (-0.16, 0.09)

-0.11 (-0.26, 0.04)

-0.11 (-0.28, 0.06)

-0.11 (-0.24, 0.02)

-0.06 (-0.20, 0.09)

0.01 (-0.19, 0.22)

-0.36 (-0.68, -0.03)

-0.16 (-0.45, 0.13)

0.02 (-0.17, 0.20)

0

-.778

.778

**Study (year)**

**WMD (95% CI)**

1. **Allelic model (A vs. G)**

Ma, X (2004)

Bayoumy, N. M (2012)

Ramos-Lopez, O (2013)

Zhang, Y (2014)

Solakivi, T (2015)

Yuan Y (2015)

Mrizak, I (2015)

Zhang, Y (2015)

Boghdady, A (2016)

Ramos-Lopez, O (2016)

Momeni-Moghaddam, M (2019)

0.00 (-0.32, 0.33)

-0.58 (-1.49, 0.32)

-0.00 (-0.27, 0.27)

-0.04 (-0.19, 0.11)

0.01 (-0.12, 0.13)

-0.06 (-0.20, 0.08)

-0.07 (-0.20, 0.05)

0.10 (-0.35, 0.56)

-0.31 (-0.87, 0.26)

0.07 (-0.12, 0.25)

0.08 (-0.34, 0.51)

0

-1.49

1.49

**Study (year)**

**WMD (95% CI)**

**B) Dominant model (AA+GA vs. GG)**

Ma, X (2004)

Bayoumy, N. M (2012)

Ramos-Lopez, O (2013)

Zhang, Y (2014)

Solakivi, T (2015)

Yuan Y (2015)

Mrizak, I (2015)

Zhang, Y (2015)

Boghdady, A (2016)

Ramos-Lopez, O (2016)

Momeni-Moghaddam, M (2019)

-0.49 (-1.42, 0.43)

-1.22 (-1.70, -0.73)

-0.12 (-0.46, 0.22)

-0.27 (-0.60, 0.06)

-0.32 (-0.69, 0.05)

-0.23 (-0.52, 0.06)

-0.07 (-0.44, 0.30)

-0.10 (-0.37, 0.17)

-0.87 (-1.66, -0.08)

-0.45 (-1.10, 0.20)

-0.05 (-0.31, 0.21)

0

-1.7

1.7

**Study (year)**

**WMD (95% CI)**

**C) Recessive model (AA vs. GA + GG)**

Ma, X (2004)

Bayoumy, N. M (2012)

Ramos-Lopez, O (2013)

Zhang, Y (2014)

Solakivi, T (2015)

Yuan Y (2015)

Mrizak, I (2015)

Zhang, Y (2015)

Boghdady, A (2016)

Ramos-Lopez, O (2016)

Momeni-Moghaddam, M (2019)

-0.62 (-1.72, 0.49)

-1.63 (-2.68, -0.58)

-0.01 (-0.28, 0.26)

-0.20 (-0.52, 0.13)

-0.23 (-0.60, 0.15)

-0.17 (-0.45, 0.11)

-0.08 (-0.36, 0.19)

0.06 (-0.23, 0.34)

-1.13 (-2.07, -0.18)

-0.48 (-1.21, 0.25)

0.09 (-0.18, 0.35)

0

-2.68

2.68

**Study (year)**

**WMD (95% CI)**

**D) Homozygous model (AA vs. GG)**

Ma, X (2004)

Bayoumy, N. M (2012)

Ramos-Lopez, O (2013)

Zhang, Y (2014)

Solakivi, T (2015)

Yuan Y (2015)

Mrizak, I (2015)

Zhang, Y (2015)

Boghdady, A (2016)

Ramos-Lopez, O (2016)

Momeni-Moghaddam, M (2019)

0.07 (-0.14, 0.27)

-0.33 (-1.24, 0.58)

-0.00 (-0.38, 0.37)

-0.02 (-0.15, 0.10)

0.01 (-0.12, 0.15)

-0.05 (-0.17, 0.08)

-0.19 (-0.40, 0.03)

0.15 (-0.45, 0.75)

-0.16 (-0.73, 0.42)

0.07 (-0.12, 0.27)

0.12 (-0.44, 0.68)

0

-1.24

1.24

**E) Heterozygous model (GA vs. GG)**

**Study (year)**

**WMD (95% CI)**

**Supplementary Figure S16-** Forest plot of the cumulative meta-analysis for studies which examined the association between genotype distribution of rs1761667 and total cholesterol (mg/dl) in adults.

Ma, X (2004)

Madden, J (2008)

Bayoumy, N. M (2012)

Ramos-Lopez, O (2013)

Dawczynski, Ch (2013)

Zhang, Y (2014)

Yuan Y (2015)

Boghdady,A (2016)

Ramos-Lopez, O (2016)

Fujii, R (2019)

Momeni-Moghaddam, M (2019)

-0.22 (-0.41, -0.04)

-0.19 (-0.34, -0.04)

-0.57 (-0.86, -0.28)

-0.22 (-0.34, -0.09)

-0.22 (-0.34, -0.10)

-0.16 (-0.29, -0.04)

-0.18 (-0.30, -0.06)

-0.48 (-0.72, -0.25)

-0.20 (-0.33, -0.06)

-0.31 (-0.62, -0.00)

-0.20 (-0.31, -0.10)

0

-.858

.858

**Study (year)**

**WMD (95% CI)**

1. **Allelic model (A vs. G)**

Ma, X (2004)

Madden, J (2008)

Bayoumy, N. M (2012)

Ramos-Lopez, O (2013)

Dawczynski, Ch (2013)

Zhang, Y (2014)

Yuan, Y (2015)

Boghdady,A (2016)

Ramos-Lopez, O (2016)

Fujii, R (2019)

Momeni-Moghaddam, M (2019)

-0.30 (-0.61, 0.01)

-0.24 (-0.49, 0.01)

-1.53 (-2.45, -0.61)

-0.18 (-0.37, 0.01)

-0.18 (-0.36, -0.00)

-0.19 (-0.37, -0.00)

-0.24 (-0.42, -0.05)

-0.98 (-1.98, 0.01)

-0.22 (-0.44, -0.01)

-0.61 (-1.38, 0.16)

-0.17 (-0.33, -0.02)

0

-2.45

2.45

**Study (year)**

**WMD (95% CI)**

**B) Dominant model (AA+GA vs. GG)**

Ma, X (2004)

Madden, J (2008)

Bayoumy, N. M (2012)

Ramos-Lopez, O (2013)

Dawczynski, Ch (2013)

Zhang, Y (2014)

Yuan, Y (2015)

Boghdady, A (2016)

Ramos-Lopez, O (2016)

Fujii, R (2019)

Momeni-Moghaddam, M (2019)

-0.47 (-0.93, -0.01)

-0.39 (-0.75, -0.03)

-1.16 (-1.65, -0.68)

-0.41 (-0.75, -0.06)

-0.43 (-0.75, -0.10)

-0.31 (-0.63, 0.01)

-0.30 (-0.58, -0.02)

-0.94 (-1.51, -0.36)

-0.40 (-0.72, -0.09)

-0.65 (-1.28, -0.03)

-0.38 (-0.69, -0.08)

0

-1.65

1.65

**Study (year)**

**WMD (95% CI)**

**C) Recessive model (AA vs. GA + GG)**

Ma, X (2004)

Madden, J (2008)

Bayoumy, N. M (2012)

Ramos-Lopez, O (2013)

Dawczynski, Ch (2013)

Zhang, Y (2014)

Yuan Y (2015)

Boghdady,A (2016)

Ramos-Lopez, O (2016)

Fujii, R (2019)

Momeni-Moghaddam, M (2019)

-0.66 (-1.23, -0.09)

-0.51 (-0.95, -0.06)

-2.40 (-3.55, -1.25)

-0.41 (-0.70, -0.12)

-0.42 (-0.69, -0.14)

-0.39 (-0.78, -0.01)

-0.39 (-0.73, -0.06)

-1.60 (-3.11, -0.09)

-0.51 (-0.90, -0.12)

-1.09 (-2.26, 0.09)

-0.37 (-0.62, -0.12)

0

-3.55

3.55

**Study (year)**

**WMD (95% CI)**

**D) Homozygous model (AA vs. GG)**

Ma, X (2004)

Madden, J (2008)

Bayoumy, N. M (2012)

Ramos-Lopez, O (2013)

Dawczynski, Ch (2013)

Zhang, Y (2014)

Yuan Y (2015)

Boghdady,A (2016)

Ramos-Lopez, O (2016)

Fujii, R (2019)

Momeni-Moghaddam, M (2019)

-0.24 (-0.53, 0.06)

-0.18 (-0.42, 0.05)

-1.39 (-2.32, -0.45)

-0.11 (-0.40, 0.17)

-0.10 (-0.38, 0.17)

-0.14 (-0.31, 0.02)

-0.20 (-0.37, -0.02)

-0.85 (-1.82, 0.12)

-0.16 (-0.36, 0.04)

-0.50 (-1.21, 0.20)

-0.11 (-0.35, 0.13)

0

-2.32

2.32

**E) Heterozygous model (GA vs. GG)**

**Study (year)**

**WMD (95% CI)**

**Supplementary Figure S17-** Forest plot of the cumulative meta-analysis for studies which examined the association between genotype distribution of rs1761667 and triglyceride (mg/dl) in adults.

Ma, X (2004)

Madden, J (2008)

Bayoumy, N. M (2012)

Ramos-Lopez, O (2013)

Dawczynski, Ch (2013)

Zhang, Y (2014)

Yuan, Y (2015)

Zhang, Y (2015)

Boghdady, A (2016)

Ramos-Lopez, O (2016)

Fujii, R (2019)

Momeni-Moghaddam, M (2019)

0.17 (-0.01, 0.35)

0.12 (-0.04, 0.29)

0.52 (0.24, 0.81)

0.07 (-0.08, 0.22)

0.05 (-0.09, 0.20)

0.12 (-0.02, 0.25)

0.12 (0.01, 0.24)

0.03 (-0.09, 0.16)

0.32 (-0.12, 0.76)

0.14 (-0.01, 0.29)

0.20 (-0.14, 0.54)

0.04 (-0.07, 0.15)

0

-.811

.811

**Study (year)**

**WMD (95% CI)**

1. **Allelic model (A vs. G)**

Ma, X (2004)

Madden, J (2008)

Bayoumy, N. M (2012)

Ramos-Lopez, O (2013)

Dawczynski, Ch (2013)

Zhang, Y (2014)

Yuan, Y (2015)

Zhang, Y (2015)

Boghdady, A (2016)

Ramos-Lopez, O (2016)

Fujii, R (2019)

Momeni-Moghaddam, M (2019)

0.11 (-0.10, 0.33)

0.08 (-0.09, 0.26)

0.82 (-0.08, 1.73)

-0.02 (-0.29, 0.26)

-0.02 (-0.28, 0.24)

0.09 (-0.07, 0.25)

0.09 (-0.05, 0.22)

-0.06 (-0.27, 0.15)

0.29 (-0.69, 1.26)

0.12 (-0.07, 0.31)

0.09 (-0.31, 0.49)

-0.05 (-0.25, 0.14)

0

-1.73

1.73

**Study (year)**

**WMD (95% CI)**

**B) Dominant model (AA+GA vs. GG)**

Ma, X (2004)

Madden, J (2008)

Bayoumy, N. M (2012)

Ramos-Lopez, O (2013)

Dawczynski, Ch (2013)

Zhang, Y (2014)

Yuan, Y (2015)

Zhang, Y (2015)

Boghdady, A (2016)

Ramos-Lopez, O (2016)

Fujii, R (2019)

Momeni-Moghaddam, M (2019)

0.46 (-0.00, 0.92)

0.33 (-0.07, 0.73)

1.25 (0.76, 1.73)

0.25 (0.00, 0.50)

0.21 (-0.03, 0.45)

0.29 (-0.02, 0.59)

0.31 (0.04, 0.58)

0.20 (0.01, 0.39)

0.91 (0.15, 1.68)

0.32 (-0.02, 0.65)

0.59 (-0.21, 1.39)

0.21 (0.03, 0.39)

0

-1.73

1.73

**Study (year)**

**WMD (95% CI)**

**C) Recessive model (AA vs. GA + GG)**

Ma, X (2004)

Madden, J (2008)

Bayoumy, N. M (2012)

Ramos-Lopez, O (2013)

Dawczynski, Ch (2013)

Zhang, Y (2014)

Yuan, Y (2015)

Zhang, Y (2015)

Boghdady, A (2016)

Ramos-Lopez, O (2016)

Fujii, R (2019)

Momeni-Moghaddam, M (2019)

0.29 (-0.03, 0.62)

0.21 (-0.10, 0.51)

1.33 (0.31, 2.35)

0.14 (-0.18, 0.47)

0.11 (-0.19, 0.42)

0.22 (-0.03, 0.48)

0.24 (0.02, 0.46)

0.08 (-0.16, 0.32)

0.75 (-0.35, 1.86)

0.25 (-0.03, 0.54)

0.43 (-0.28, 1.13)

0.10 (-0.12, 0.33)

0

-2.35

2.35

**Study (year)**

**WMD (95% CI)**

**D) Homozygous model (AA vs. GG)**

Ma, X (2004)

Madden, J (2008)

Bayoumy, N. M (2012)

Ramos-Lopez, O (2013)

Dawczynski, Ch (2013)

Zhang, Y (2014)

Yuan Y (2015)

Zhang, Y (2015)

Boghdady,A (2016)

Ramos-Lopez, O (2016)

Fujii, R (2019)

Momeni-Moghaddam, M (2019)

0.07 (-0.12, 0.27)

0.06 (-0.09, 0.20)

0.74 (-0.18, 1.65)

-0.07 (-0.34, 0.21)

-0.06 (-0.32, 0.20)

0.06 (-0.08, 0.20)

0.05 (-0.07, 0.17)

-0.10 (-0.32, 0.12)

0.19 (-0.83, 1.20)

0.08 (-0.08, 0.25)

0.04 (-0.35, 0.44)

-0.09 (-0.30, 0.11)

0

-1.65

1.65

**E) Heterozygous model (GA vs. GG)**

**Study (year)**

**WMD (95% CI)**

**Supplementary Figure S18-** Forest plot of the cumulative meta-analysis for studies which examined the association between genotype distribution of rs1761667 and HDL cholesterol (mg/dl) in adults.

Madden, J (2008)

Bayoumy, N. M (2012)

Ramos-Lopez, O (2013)

Zhang, Y (2014)

Yuan, Y (2015)

Mrizak, I (2015)

Zhang, Y (2015)

Boghdady, A (2016)

Ramos-Lopez, O (2016)

Momeni-Moghaddam, M (2019)

-0.29 (-0.58, 0.00)

-0.56 (-0.85, -0.27)

-0.04 (-0.30, 0.23)

-0.24 (-0.41, -0.07)

-0.21 (-0.36, -0.06)

-0.12 (-0.36, 0.11)

0.05 (-0.28, 0.38)

-0.37 (-0.79, 0.05)

-0.27 (-0.49, -0.06)

0.05 (-0.25, 0.35)

0

-.85

.85

**Study (year)**

**WMD (95% CI)**

1. **Allelic model (A vs. G)**

Madden, J (2008)

Bayoumy, N. M (2012)

Ramos-Lopez, O (2013)

Zhang, Y (2014)

Yuan, Y (2015)

Mrizak, I (2015)

Zhang, Y (2015)

Boghdady, A (2016)

Ramos-Lopez, O (2016)

Momeni-Moghaddam, M (2019)

-0.26 (-0.78, 0.26)

-0.93 (-1.84, -0.02)

-0.10 (-0.28, 0.08)

-0.23 (-0.47, 0.01)

-0.19 (-0.40, 0.01)

-0.18 (-0.35, -0.00)

0.03 (-0.64, 0.69)

-0.33 (-1.44, 0.79)

-0.24 (-0.59, 0.11)

0.01 (-0.60, 0.62)

0

-1.84

1.84

**Study (year)**

**WMD (95% CI)**

**B) Dominant model (AA+GA vs. GG)**

Madden, J (2008)

Bayoumy, N. M (2012)

Ramos-Lopez, O (2013)

Zhang, Y (2014)

Yuan, Y (2015)

Mrizak, I (2015)

Zhang, Y (2015)

Boghdady, A (2016)

Ramos-Lopez, O (2016)

Momeni-Moghaddam, M (2019)

-0.73 (-1.53, 0.08)

-1.33 (-1.82, -0.85)

-0.07 (-0.69, 0.54)

-0.54 (-1.03, -0.05)

-0.46 (-0.88, -0.04)

-0.24 (-0.88, 0.40)

-0.01 (-0.46, 0.45)

-1.11 (-1.68, -0.53)

-0.63 (-1.20, -0.05)

0.04 (-0.37, 0.45)

0

-1.82

1.82

**Study (year)**

**WMD (95% CI)**

**C) Recessive model (AA vs. GA + GG)**

Madden, J (2008)

Bayoumy, N. M (2012)

Ramos-Lopez, O (2013)

Zhang, Y (2014)

Yuan, Y (2015)

Mrizak, I (2015)

Zhang, Y (2015)

Boghdady, A (2016)

Ramos-Lopez, O (2016)

Momeni-Moghaddam, M (2019)

-0.97 (-2.18, 0.25)

-2.47 (-3.63, -1.32)

-0.22 (-0.74, 0.30)

-0.67 (-1.28, -0.05)

-0.54 (-1.03, -0.05)

-0.38 (-0.91, 0.15)

-0.07 (-0.61, 0.47)

-1.42 (-3.43, 0.59)

-0.81 (-1.61, -0.01)

-0.02 (-0.51, 0.47)

-3.63

3.63

0

**Study (year)**

**WMD (95% CI)**

**D) Homozygous model (AA vs. GG)**

Madden, J (2008)

Bayoumy, N. M (2012)

Ramos-Lopez, O (2013)

Zhang, Y (2014)

Yuan, Y (2015)

Mrizak, I (2015)

Zhang, Y (2015)

Boghdady, A (2016)

Ramos-Lopez, O (2016)

Momeni-Moghaddam, M (2019)

-0.14 (-0.65, 0.37)

-0.68 (-1.60, 0.23)

-0.25 (-0.43, -0.08)

-0.16 (-0.41, 0.08)

-0.14 (-0.36, 0.07)

-0.24 (-0.46, -0.03)

0.00 (-0.82, 0.83)

-0.11 (-1.16, 0.93)

-0.14 (-0.48, 0.20)

-0.01 (-0.76, 0.74)

0

-1.6

1.6

**E) Heterozygous model (GA vs. GG)**

**Study (year)**

**WMD (95% CI)**

**Supplementary Figure S19-** Forest plot of the cumulative meta-analysis for studies which examined the association between genotype distribution of rs1761667 and LDL cholesterol (mg/dl) in adults.

Ma, X (2004)

Bayoumy, N. M (2012)

Solakivi, T (2015)

Fujii, R (2019)

Momeni-Moghaddam, M (2019)

-0.29 (-0.69, 0.11)

-0.91 (-1.21, -0.62)

-0.20 (-0.45, 0.04)

-0.49 (-1.30, 0.31)

-0.16 (-0.36, 0.04)

0

-1.3

1.3

**Study (year)**

**WMD (95% CI)**

1. **Allelic model (A vs. G)**

Ma, X (2004)

Bayoumy, N. M (2012)

Solakivi, T (2015)

Fujii, R (2019)

Momeni-Moghaddam, M (2019)

-0.57 (-1.16, 0.03)

-2.62 (-3.59, -1.65)

-0.31 (-0.67, 0.05)

-1.29 (-3.82, 1.25)

-0.21 (-0.50, 0.09)

0

-3.82

3.82

**Study (year)**

**WMD (95% CI)**

**B) Dominant model (AA+GA vs. GG)**

-3.82

Ma, X (2004)

Bayoumy, N. M (2012)

Solakivi, T (2015)

Fujii, R (2019)

Momeni-Moghaddam, M (2019)

-0.73 (-1.82, 0.35)

-2.11 (-2.65, -1.57)

-0.52 (-1.11, 0.07)

-1.23 (-2.92, 0.47)

-0.44 (-0.93, 0.05)

0

-2.92

2.92

**Study (year)**

**WMD (95% CI)**

**C) Recessive model (AA vs. GA + GG)**

Ma, X (2004)

Bayoumy, N. M (2012)

Solakivi, T (2015)

Fujii, R (2019)

Momeni-Moghaddam, M (2019)

-1.52 (-2.79, -0.24)

-7.32 (-9.47, -5.18)

-0.69 (-1.38, 0.00)

-3.76 (-10.57, 3.05)

-0.50 (-1.06, 0.06)

0

-10.6

10.6

**D) Homozygous model (AA vs. GG)**

**Study (year)**

**WMD (95% CI)**

Ma, X (2004)

Bayoumy, N. M (2012)

Solakivi, T (2015)

Fujii, R (2019)

Momeni-Moghaddam, M (2019)

-0.65 (-1.31, 0.02)

-2.83 (-3.85, -1.82)

-0.36 (-0.76, 0.04)

-1.36 (-4.16, 1.44)

-0.24 (-0.56, 0.09)

0

-4.16

4.16

**E) Heterozygous model (GA vs. GG)**

**Study (year)**

**WMD (95% CI)**

**Supplementary Figure S20-** Forest plot of the cumulative meta-analysis for studies which examined the association between genotype distribution of rs1761667 and systolic blood pressure (mmHg) in adults.

Ma, X (2004)

Bayoumy, N. M (2012)

Solakivi, T (2015)

Yuan, Y (2015)

Fujii, R (2019)

Momeni-Moghaddam, M (2019)

-0.04 (-0.18, 0.10)

-0.18 (-0.46, 0.11)

-0.03 (-0.12, 0.05)

-0.03 (-0.10, 0.04)

-0.12 (-0.24, 0.01)

-0.02 (-0.08, 0.04)

0

-.46

.46

1. **Allelic model (A vs. G)**

**Study (year)**

**WMD (95% CI)**

Ma, X (2004)

Bayoumy, N. M (2012)

Solakivi, T (2015)

Yuan, Y (2015)

Fujii, R (2019)

Momeni-Moghaddam, M (2019)

-0.02 (-0.15, 0.11)

-0.11 (-1.01, 0.79)

-0.02 (-0.13, 0.08)

-0.03 (-0.13, 0.08)

-0.07 (-0.24, 0.11)

-0.02 (-0.11, 0.08)

0

-1.01

1.01

**B) Dominant model (AA+GA vs. GG)**

**Study (year)**

**WMD (95% CI)**

-1.01

Ma, X (2004)

Bayoumy, N. M (2012)

Solakivi, T (2015)

Yuan, Y (2015)

Fujii, R (2019)

Momeni-Moghaddam, M (2019)

-0.19 (-0.57, 0.20)

-0.43 (-0.89, 0.03)

-0.12 (-0.34, 0.10)

-0.09 (-0.28, 0.10)

-0.38 (-0.65, -0.11)

-0.09 (-0.25, 0.07)

0

-.887

.887

**Study (year)**

**WMD (95% CI)**

**C) Recessive model (AA vs. GA + GG)**

Ma, X (2004)

Bayoumy, N. M (2012)

Solakivi, T (2015)

Yuan, Y (2015)

Fujii, R (2019)

Momeni-Moghaddam, M (2019)

-0.13 (-0.51, 0.25)

-0.33 (-1.30, 0.63)

-0.08 (-0.29, 0.12)

-0.06 (-0.23, 0.10)

-0.35 (-0.68, -0.03)

-0.06 (-0.19, 0.08)

0

-1.3

1.3

**D) Homozygous model (AA vs. GG)**

**Study (year)**

**WMD (95% CI)**

Ma, X (2004)

Bayoumy, N. M (2012)

Solakivi, T (2015)

Yuan, Y (2015)

Fujii, R (2019)

Momeni-Moghaddam, M (2019)

-0.00 (-0.14, 0.14)

0.00 (-0.91, 0.91)

-0.01 (-0.12, 0.11)

-0.01 (-0.12, 0.10)

-0.01 (-0.19, 0.18)

-0.00 (-0.10, 0.10)

0

-.907

.907

**E) Heterozygous model (GA vs. GG)**

**Study (year)**

**WMD (95% CI)**

**Supplementary Figure S21-** Forest plot of the cumulative meta-analysis for studies which examined the association between genotype distribution of rs1761667 and diastolic blood pressure (mmHg) in adults.

Ma, X (2004)

Madden, J (2008)

Solakivi, T (2015)

Yuan Y (2015)

Ramos-Lopez, O (2016)

Fujii, R (2019)

Momeni-Moghaddam, M (2019)

0.10 (0.01, 0.19)

0.11 (0.02, 0.19)

0.17 (0.06, 0.27)

0.16 (0.06, 0.25)

0.11 (0.02, 0.19)

0.07 (-0.07, 0.22)

0.12 (0.01, 0.22)

0

-.273

.273

1. **Allelic model (A vs. G)**

**Study (year)**

**WMD (95% CI)**

Ma, X (2004)

Madden, J (2008)

Solakivi, T (2015)

Yuan Y (2015)

Ramos-Lopez, O (2016)

Fujii, R (2019)

Momeni-Moghaddam, M (2019)

0.09 (-0.05, 0.22)

0.10 (-0.03, 0.22)

0.24 (-0.01, 0.49)

0.22 (-0.00, 0.44)

0.10 (-0.02, 0.23)

0.09 (-0.09, 0.27)

0.17 (-0.03, 0.38)

0

-.488

.488

**B) Dominant model (AA+GA vs. GG)**

**Study (year)**

**WMD (95% CI)**

Ma, X (2004)

Madden, J (2008)

Solakivi, T (2015)

Yuan, Y (2015)

Ramos-Lopez, O (2016)

Fujii, R (2019)

Momeni-Moghaddam, M (2019)

0.19 (0.03, 0.36)

0.20 (0.05, 0.35)

0.23 (0.12, 0.34)

0.22 (0.11, 0.32)

0.19 (0.05, 0.34)

0.10 (-0.24, 0.43)

0.19 (0.09, 0.29)

0

-.431

.431

**Study (year)**

**WMD (95% CI)**

**C) Recessive model (AA vs. GA + GG)**

Ma, X (2004)

Madden, J (2008)

Solakivi, T (2015)

Yuan Y (2015)

Ramos-Lopez, O (2016)

Fujii, R (2019)

Momeni-Moghaddam, M (2019)

0.19 (-0.01, 0.39)

0.20 (0.02, 0.39)

0.34 (0.08, 0.59)

0.31 (0.07, 0.54)

0.20 (0.02, 0.38)

0.13 (-0.21, 0.47)

0.23 (-0.03, 0.48)

0

-.593

.593

**D) Homozygous model (AA vs. GG)**

**Study (year)**

**WMD (95% CI)**

Ma, X (2004)

Madden, J (2008)

Solakivi, T (2015)

Yuan Y (2015)

Ramos-Lopez, O (2016)

Fujii, R (2019)

Momeni-Moghaddam, M (2019)

0.05 (-0.09, 0.19)

0.06 (-0.08, 0.19)

0.21 (-0.05, 0.48)

0.20 (-0.04, 0.43)

0.06 (-0.07, 0.20)

0.08 (-0.10, 0.27)

0.16 (-0.06, 0.37)

0

-.482

.482

**E) Heterozygous model (GA vs. GG)**

**Study (year)**

**WMD (95% CI)**

**Supplementary Figure S22-** Forest plot of the cumulative meta-analysis for studies which examined the association between genotype distribution of rs1761667 and fasting blood glucose (mg/dl) in adults.

**References**

1 Daoudi, H. *et al.* Oral Fat Sensing and CD36 Gene Polymorphism in Algerian Lean and Obese Teenagers. *Nutrients* **7**, 9096-9104, doi:10.3390/nu7115455 (2015).

2 Karmous, I. *et al.* Orosensory detection of bitter in fat-taster healthy and obese participants: Genetic polymorphism of CD36 and TAS2R38. *Clinical nutrition (Edinburgh, Scotland)* **37**, 313-320, doi:10.1016/j.clnu.2017.06.004 (2018).

3 Banerjee, M., Gautam, S., Saxena, M., Bid, H. K. & Agrawal, C. G. Association of CD36 gene variants rs1761667 (G &gt; A) and rs1527483 (C &gt; T) with Type 2 diabetes in North Indian population. *International Journal of Diabetes Mellitus* **2**, 179-183, doi:https://doi.org/10.1016/j.ijdm.2010.08.002 (2010).

4 Barbarossa, I. T. *et al.* Variant in a common odorant-binding protein gene is associated with bitter sensitivity in people. *Behavioural brain research* **329**, 200-204, doi:https://doi.org/10.1016/j.bbr.2017.05.015 (2017).

5 Delgado-Lista, J. *et al.* Top single nucleotide polymorphisms affecting carbohydrate metabolism in metabolic syndrome: from the LIPGENE study. *The Journal of Clinical Endocrinology & Metabolism* **99**, E384-E389, doi: https://doi.org/10.1210/jc.2013-3165 (2014).

6 Enciso-Ramírez, M. *et al.* CD36 gene polymorphism -31118 G &gt; A (rs1761667) is associated with overweight and obesity but not with fat preferences in Mexican children. *International Journal for Vitamin and Nutrition Research*, 1-9, doi: https://doi.org/10.1024/0300-9831/a000656 (2020).

7 Gautam, S., Agrawal, C. G. & Banerjee, M. CD36 gene variants in early prediction of type 2 diabetes mellitus. *Genetic testing and molecular biomarkers* **19**, 144-149, doi:https://doi.org/10.1089/gtmb.2014.0265 (2015).

8 Ghosh, A. *et al.* Platelet CD36 surface expression levels affect functional responses to oxidized LDL and are associated with inheritance of specific genetic polymorphisms. *Blood* **117**, 6355-6366, doi:https://doi.org/10.1182/blood-2011-02-338582 (2011).

9 Choi, J.-H. Genetic variation in CD36 is associated with dietary intake in Korean males. *British Journal of Nutrition* **125**, 1321-1330, doi:DOI: https://doi.org/10.1017/S0007114520003748 (2021).

10 Connor, T. *et al.* CD36 polymorphisms and the age of disease onset in patients with pathogenic variants within the mutation cluster region of APC. *Hereditary cancer in clinical practice* **19**, 1-9, doi:https://doi.org/10.1186/s13053-021-00183-0 (2021).

11 Diószegi, J. *et al.* Association of single nucleotide polymorphisms with taste and food preferences of the Hungarian general and Roma populations. *Appetite* **164**, 105270, doi:https://doi.org/10.1016/j.appet.2021.105270 (2021).

12 Karthi, M. *et al.* Single nucleotide polymorphism in CD36: Correlation to peptide YY levels in obese and non-obese adults. *Clinical Nutrition* **40**, 2707-2715, doi:https://doi.org/10.1016/j.clnu.2021.02.044 (2021).

13 Lee, D.-H. *et al.* Association between rs1761667 CD36 polymorphism and risk of stroke in Korean patients with type 2 diabetes. *Chinese Medical Journal* **134**, 2385, doi:https://doi.org/10.1097/CM9.0000000000001501 (2021).

14 Graham, C. A.-M. *et al.* The associations between bitter and fat taste sensitivity, and dietary fat intake: Are they impacted by genetic predisposition? *Chemical Senses* **46**, 1-11, doi:https://doi.org/10.1093/chemse/bjab029 (2021).

15 Fakhry, M. M. *et al.* A possible novel co-relation of locus 7q11 rs1761667 polymorphism with the severity of preeclampsia in Egyptian pregnant women. *Meta Gene* **24**, doi:https://doi.org/10.1016/j.mgene.2020.100650 (2020).

16 Yang, Y., Luo, B. R., Hu, M., Zhao, D. M. & Jing, W. J. Association of CD36 gene single nucleotide polymorphism with gestational diabetes mellitus in Chinese Han population. *Clinical and Experimental Obstetrics & Gynecology* **45**, 266-271, doi:https://doi.org/10.12891/ceog3844.2018 (2018).

17 Pioltine, M. B. *et al.* Genetic Variation in CD36 Is Associated with Decreased Fat and Sugar Intake in Obese Children and Adolescents. *Journal of nutrigenetics and nutrigenomics* **9**, 300-305, doi:https://doi.org/10.1159/000455915 (2016).

18 Sayed, A. *et al.* CD36 AA genotype is associated with decreased lipid taste perception in young obese, but not lean, children. *International Journal of Obesity* **39**, 920-924, doi: https://doi.org/10.1038/ijo.2015.20 (2015).

19 Salim, S., Kartawidjajaputra, F. & Suwanto, A. Association of FTO rs9939609 and CD36 rs1761667 with Visceral Obesity. *Journal of nutritional science and vitaminology* **66**, S329-S335, doi:https://doi.org/10.3177/jnsv.66.S329 (2020).

20 ZANG, J. *et al.* Relationship between CD36 single nucleotide polymorphisms and atherosclerotic cerebral infarction in elderly patients. *Journal of Shandong University (Health Sciences)* **51**, 99-104, doi:https://doi.org/10.6040/j.issn.1671-7554.2013.05.022 (2013).

21 Wells, G. *et al.* (2011).

22 Bayoumy, N. M., El-Shabrawi, M. M. & Hassan, H. H. Association of cluster of differentiation 36 gene variant rs1761667 (G>A) with metabolic syndrome in Egyptian adults. *Saudi Medical Journal* **33**, 489-494 (2012).

23 Boghdady, A. *et al.* Association between rs1761667 polymorphism of CD36 gene and risk of coronary atherosclerosis in Egyptian population. *Cardiovascular diagnosis and therapy* **6**, 120-130, doi: https://doi.org/10.21037/cdt.2015.12.15 (2016).

24 Melis, M. *et al.* Polymorphism rs1761667 in the CD36 Gene Is Associated to Changes in Fatty Acid Metabolism and Circulating Endocannabinoid Levels Distinctively in Normal Weight and Obese Subjects. *Frontiers in physiology* **8**, 1006, doi: https://doi.org/10.3389/fphys.2017.01006 (2017).

25 Momeni-Moghaddam, M. A. & Asadikaram, G. CD36 gene polymorphism rs1761667 (G > A) is associated with hypertension and coronary artery disease in an Iranian population. **19**, 140, doi: https://doi.org/10.1186/s12872-019-1111-6 (2019).

26 Solakivi, T., Kunnas, T. & Nikkari, S. T. Contribution of fatty acid transporter (CD36) genetic variant rs1761667 to body mass index, the TAMRISK study. *Scandinavian journal of clinical and laboratory investigation* **75**, 254-258, doi:https://doi.org/10.3109/00365513.2014.1003596 (2015).

27 Yuan, Y. *et al.* Relationship between the polymorphism of rs17154181 and rs1761667 sites of CD36 gene and macroangiopathy in patients with type 2 diabetes mellitus in Guangxi province. *Chinese general practice* **18**, 2421-2425 (2015).

28 Zhang, Y. *et al.* Associations between CD36 gene polymorphisms and susceptibility to coronary artery heart disease. *Brazilian Journal of Medical and Biological Research* **47**, 895-903, doi: https://doi.org/10.1590/1414-431x20143825 (2014).

29 Zhang, Y. *et al.* CD36 genotype associated with ischemic stroke in Chinese Han. *International journal of clinical and experimental medicine* **8**, 16149-16157 (2015).

30 Herzog, R. *et al.* Newcastle-Ottawa Scale adapted for cross-sectional studies. *Available from https://eje.bioscientifica.com/supplemental/journals/eje/176/3/R137.xml/supplementary_figure_1.pdf* (2013).

31 Dalton, M. *Biopsychological investigation of hedonic processes in individuals susceptible to overeating: role of liking and wanting in trait binge eating*. (University of Leeds, 2013).

32 Dawczynski, C. *et al.* Randomized placebo-controlled intervention with n-3 LC-PUFA-supplemented yoghurt: effects on circulating eicosanoids and cardiovascular risk factors. *Clinical nutrition* **32**, 686-696, doi:https://doi.org/10.1016/j.clnu.2012.12.010 (2013).

33 Fujii, R. *et al.* Cluster of differentiation 36 gene polymorphism (rs1761667) is associated with dietary MUFA intake and hypertension in a Japanese population. *The British journal of nutrition* **121**, 1215-1222, doi: https://doi.org/10.1017/s0007114519000679 (2019).

34 Ma, X. *et al.* A common haplotype at the CD36 locus is associated with high free fatty acid levels and increased cardiovascular risk in Caucasians. *Human molecular genetics* **13**, 2197-2205, doi:https://doi.org/10.1093/hmg/ddh233 (2004).

35 Madden, J. *et al.* Polymorphisms in the CD36 gene modulate the ability of fish oil supplements to lower fasting plasma triacyl glycerol and raise HDL cholesterol concentrations in healthy middle-aged men. *Prostaglandins, leukotrienes and essential fatty acids* **78**, 327-335, doi:https://doi.org/10.1016/j.plefa.2008.04.003 (2008).

36 Mrizak, I. *et al.* The A allele of cluster of differentiation 36 (CD36) SNP 1761667 associates with decreased lipid taste perception in obese Tunisian women. *The British journal of nutrition* **113**, 1330-1337, doi: https://doi.org/10.1017/s0007114515000343 (2015).

37 Ramos-Arellano, L. E. *et al.* CD36 haplotypes are associated with lipid profile in normal-weight subjects. *Lipids in Health and Disease* **12**, doi: https://doi.org/10.1186/1476-511x-12-167 (2013).

38 Ramos-Lopez, O. *et al.* CD36 genetic variation, fat intake and liver fibrosis in chronic hepatitis C virus infection. *Journal of food science* **8**, 1067-1074, doi: https://doi.org/10.4254/wjh.v8.i25.1067 (2016).

39 Shen, Y. C., Kennedy, O. B. & Methven, L. The effect of genotypical and phenotypical variation in taste sensitivity on liking of ice cream and dietary fat intak. *Food Quality and Preference* **55**, 79-90, doi:https://doi.org/10.1016/j.foodqual.2016.08.010 (2017).
